# A gene-rich fraction analysis of the *Passiflora edulis* genome reveals highly conserved microsyntenic regions with two related Malpighiales species

**DOI:** 10.1038/s41598-018-31330-8

**Published:** 2018-08-29

**Authors:** Carla Freitas Munhoz, Zirlane Portugal Costa, Luiz Augusto Cauz-Santos, Alina Carmen Egoávil Reátegui, Nathalie Rodde, Stéphane Cauet, Marcelo Carnier Dornelas, Philippe Leroy, Alessandro de Mello Varani, Hélène Bergès, Maria Lucia Carneiro Vieira

**Affiliations:** 1 Departamento de Genética, Escola Superior de Agricultura “Luiz de Queiroz”, Universidade de São Paulo, 13418-900 Piracicaba, Brazil; 2 Institut National de la Recherche Agronomique (INRA), Centre National de Ressources Génomique Végétales, 31326 Castanet-Tolosan, France; 3 Departamento de Biologia Vegetal, Instituto de Biologia, Universidade Estadual de Campinas, 13083-862 Campinas, Brazil; 4 INRA, UCA, UMR 1095, GDEC, 63000 Clermont-Ferrand, France; 5 Departamento de Tecnologia, Faculdade de Ciências Agrárias e Veterinárias, Universidade Estadual Paulista, 14884-900 Jaboticabal, Brazil

## Abstract

*Passiflora edulis* is the most widely cultivated species of passionflowers, cropped mainly for industrialized juice production and fresh fruit consumption. Despite its commercial importance, little is known about the genome structure of *P. edulis*. To fill in this gap in our knowledge, a genomic library was built, and now completely sequenced over 100 large-inserts. Sequencing data were assembled from long sequence reads, and structural sequence annotation resulted in the prediction of about 1,900 genes, providing data for subsequent functional analysis. The richness of repetitive elements was also evaluated. Microsyntenic regions of *P. edulis* common to *Populus trichocarpa* and *Manihot esculenta*, two related Malpighiales species with available fully sequenced genomes were examined. Overall, gene order was well conserved, with some disruptions of collinearity identified as rearrangements, such as inversion and translocation events. The microsynteny level observed between the *P. edulis* sequences and the compared genomes is surprising, given the long divergence time that separates them from the common ancestor. *P. edulis* gene-rich segments are more compact than those of the other two species, even though its genome is much larger. This study provides a first accurate gene set for *P. edulis*, opening the way for new studies on the evolutionary issues in Malpighiales genomes.

## Introduction

The Passifloraceae family belongs to the Malpighiales order and is a member of the Rosids clade, according to classical and molecular phylogenetic analysis. The family consists of 700 species, classified in 16 genera. The majority of species belong to the genus *Passiflora* (~530 species), popularly known as passion fruits^1^. This genus is widely distributed in tropical and subtropical regions of the Neotropics. Approximately 150 species are native to Brazil, which is acknowledged to be an important centre of diversity^2^.

Among the American tropical species of *Passiflora*, 60 fruit-bearing species are marketed for human consumption. Moreover, several species and hybrids have been produced for ornamental purposes (see www.passiflora.it;)^3^, and pharmacologists have found that passion fruit vines contain bioactive compounds that are used in traditional folk medicines as anxiolytics and antispasmodics^4^. *Passiflora edulis* is the major species of passionflowers grown for fresh fruit consumption and juice production in climates ranging from cool subtropical (purple variety) to warm tropical (yellow variety). Species grown particularly in Brazil include *P. edulis* (sour passion fruit) and *P. alata* (sweet passion fruit). Because of the quality of its fruit and yield for processing into commercial juices, *P. edulis* is grown in 90% of the commercial orchards. The most recent agricultural production survey showed that 58,089 hectares were planted with passion fruits, yielding 838,444 tons per year^5^.

*P. edulis* is a diploid (*2n* = 18)^6^, self-incompatible species^7,8^, with perfect, insect-pollinated flowers. Over the last two decades, our research group has carried out studies for estimating the genetic parameters of experimental populations^9^, as well as constructing genetic maps^10,11^ and mapping quantitative loci associated with the response to *Xanthomonas axonopodis* infection^12^. Munhoz and co-workers were able to determine which gene expression patterns were significantly modulated during the *P. edulis*-*X. axonopodis* interaction^13^.

Despite its commercial success, little is known about the genome structure of *P. edulis*. The genome size has been estimated at ~1,230 Mb (1 C DNA content = 1.258 pg by flow cytometric analysis)^14^. To fill in this gap in our knowledge, a large-insert genomic BAC (Bacterial Artificial Chromosomes) library was built and denoted Ped-B-Flav (https://cnrgv.toulouse.inra.fr/library/genomic_resource/Ped-B-Flav). It contains 83,000 clones, which are kept at the National Centre for Plant Genomic Resources (CNRGV: cnrgv.toulouse.inra.fr) at INRA in Toulouse, France. In addition, previous studies provided initial insights into the *P. edulis* genome using BAC-end sequence (BES) data as a major resource^15^, and described the structural organization of the plant’s chloroplast genome, which differs from that of various Malpighiales species due to rearrangement events^16^.

Although based on small-sized sequences, BAC-end sequences can be mapped to intervals of sequenced related genomes^17^ in order to identify collinear microsyntenic regions as a preliminary step towards selecting clones for full sequencing, which can be done with high accuracy using the single-molecule real-time (SMRT) sequencing (Pacific Biosciences). This method produces long, unbiased sequences that, in turn, facilitate subsequent assembly^18^, a critical step in plants due to the high proportion of repetitive sequences throughout their genomes^19^.

Most of the projects aimed at obtaining a draft or a complete plant genome were performed using large-insert based sequencing methods^20,21^ to allow estimation of the number of genes, and abundance of transposable elements and microsatellites. In the functional part of the genome in particular, the annotation of large-inserts can provide an arsenal of biological information to facilitate comparison against databases and, in addition, to determine the distribution of BAC inserts relative to related genomes in order to examine the degree of synteny between them and gain insights into evolutionary relationships^22,23^.

In this scenario, the *P*. *edulis* genome is continuing to be studied based on the large-insert BAC library and using the SMRT sequencing platform to completely sequence over 100 inserts of BAC clones. These clones were pre-selected based on BES microsynteny results and probes homologous to transcripts from a subtractive library of *P. edulis* in response to *Xanthomonas axonopodis* infection, which allowed us to obtain a gene-rich fraction of this genome. The repetitive content, predicted genes, and coding sequences were annotated. Also, microsyntenic regions of *P. edulis* common to *Populus trichocarpa* (Salicaceae, 485 Mb^24^) and *Manihot esculenta* (Euphorbiaceae, 742 Mb^25^), two related Malpighiales species with available fully sequenced and well-annotated genomes, were identified.

## Material and Methods

### BAC Selection and DNA Preparation

BAC clones were selected from the findings of Santos *et al*.^15^, which provides an initial overview of the *P. edulis* genome using BAC-end sequence (BES) data as a major resource. The results of comparative mapping between *P. edulis’* BES and the reference genomes of *Arabidopsis thaliana*, *Populus trichocarpa* and *Vitis vinifera* were also used to choose BAC clones for sequencing. In addition, based on BES functional annotation results, the BAC-inserts with coding sequences (CDS) in one or both BESs were also selected.

A second selection procedure was performed after screening the genomic library using the probes homologous to *P. edulis* transcripts described in^13^. Briefly, the authors used suppression subtractive hybridization to construct two cDNA libraries enriched for transcripts induced and repressed by *Xanthomonas axonopodis*, respectively, 24 h after inoculation with a highly virulent bacterial strain.

The homologous probes were prepared via PCR, using as a template the genomic DNA from ‘IAPAR-123’, the accession used to construct the Ped-B-Flav BAC library. Specific primers were used to generate a single amplicon (200 to 600 bp in size) for each probe gene sequence. The ‘DecaLabel DNA Labeling Kit’ (Fermentas) was used for radiolabeling the probes. The amplification products were then purified with ‘Illustra ProbeQuant^TM^ G-50 Micro Columns’ (GE Healthcare). The library was previously gridded onto macroarrays in which 41,472 clones were double-spotted on each 22 × 22 cm nylon membrane. These membranes were submerged in a bath of SSC (Saline-Sodium Citrate) solution (6×, 17 min., 50 °C); incubated overnight (68 °C) in hybridization buffer [6× SSC, 5× Denhardt’s Solution, 0.5% (w/v) SDS (Sodium Dodecyl Sulfate)]; hybridized with denatured probes (10 min, 95 °C; 1 min., cooled on ice); and washed twice in buffer 1 [2× SSC, 0.1% (w/v) SDS] (15 min., 50 °C) and buffer 2 [0.5× SSC, 0.1% (w/v) SDS] (30 min., 50 °C). Next, the hybridized membranes were placed in a film cassette for 24 h.; radioactive signals were detected using a PhosphorImager^TM^ and Storm 820 scanner (Amersham Biosciences) and analyzed using HDFR3 software, to identify the positive clones. Each positive clone was individually validated by PCR.

In order to estimate insert sizes, the preserved cultures were scraped and a positive single colony of each BAC grown in a 96-well plate (overnight, 37 °C) containing 1200 µL of LB medium with chloramphenicol (12.5 µg/mL) and glycerol (6%). DNAs were then isolated using a NucleoSpin® 96 Flash (Macherey-Nagel) BAC DNA purification kit, digested with 5 U of FastDigest™ *Not*I enzyme (Fermentas) and size-fractioned by PFGE (6 V.cm^−1^, 5 to 15 s switch time, 16 h run time, 12.5 °C) in a Chef Mapper XA Chiller System 220 V (BioRad), followed by ethidium bromide staining and visualization. The insert sizes were determined by comparison with PFGE (pulsed-field gel electrophoresis) standard size markers.

To prepare the DNA for sequencing, 1 μl of the above cultures was allowed to regrow in 20 mL of LB medium (plus 12.5 µg/mL chloramphenicol at 37 °C overnight) under shaking (250 rpm). The cultures were then mixed in pools, at a maximum of 20 clones per pool. DNA extraction was performed using the Nucleobond Xtra Midi Plus kit (Macherey-Nagel) according to the manufacturer’s instructions.

### DNA Sequencing and Assembly From Long Sequence Reads

Approximately 5 µg of each pool was used for the construction of a SMRT library based on the standard Pacific Biosciences (San Francisco, CA, USA) preparation protocol for 10-kb libraries. Each pool was sequenced in one SMRT Cell using P6 polymerase in combination with C4 chemistry, following the manufacturer’s standard operating procedures and using the PacBio RS II long-read sequencer.

Reads were assembled by a hierarchical genome assembly process (HGAP workflow)^26^, and using the v2.2.0 SMRT® analysis software suite for HGAP implementation. Reads were first aligned by the PacBio long-read aligner or BLASR^27^ against the complete genome of *Escherichia coli*, strain K12, substrain DH10B (GenBank: CP000948.1). The *E. coli* reads, as well as low quality reads (minimum read length of 500 bp and minimum read quality of 0.80) were removed from the data set. Filtered reads were then preassembled to yield long, highly accurate sequences. To perform this step, the smallest and the longest reads were separated from each other to correct errors by mapping single-pass reads to the longest reads (seed reads), which represent the longest portion of the read length distribution. Next, sequences were filtered against vector (BAC) sequences, and the Celera assembler used to assemble data and obtain draft assemblies. The last step was performed in order to significantly reduce the remaining indels and base substitution errors in the draft assembly. The Quiver algorithm was used for this purpose. This quality-aware consensus algorithm uses rich quality scores (Quality Value/QV scores) and QV is a per-base estimate of base accuracy. QV scores over 20 are from very good data with only 1% error probability. Finally, Quiver polishes the assembly for final consensus^26^.

Once the refined assembly was obtained, each BAC-insert sequence was individualized by matching the end sequences to the pool of assembled sequences using BLAST. Read coverage was assessed by aligning the raw reads on the assembled sequences with BLASR.

### Identification and Annotation of Repetitive Sequences

Eukaryotic genomes contain a substantial portion of repetitive elements which are organized into three main classes: dispersed repeats (mostly transposable elements and retrotransposed genes), local repeats (tandem repeats and simple sequence repeats or microsatellites) and segmental duplications (duplicated genomic fragments)^28^. It is highly recommended to identify and mask repetitive regions before gene prediction. Otherwise, unmasked repeats can produce spurious BLAST alignments, resulting in false evidence for gene annotations^29^.

The v2.2 REPET package was used for *de novo* detection and annotation of transposable elements (TEs). The annotation process starts with self-alignment of the sequences by all-by-all comparison. Matching clusters are then identified based on the same cluster sequences in a given family. A consensus for each family is created, and each consensus is classified according to the structures and domains present. The last step entails annotating TE copies^30,31^.

The resulting elements were then compared with sequences deposited in the Viridiplantae section of the Repbase repeat database^32^. They were classified by PASTEC, a tool for classifying TEs by searching for structural features and similarities^33^ and implementing the hierarchical classification system proposed by^34^. Repeat masking was subsequently performed with RepeatMasker Open-3.0^35^ using the library generated by the REPET and Repbase Viridiplantae dataset^32^.

MISA^36^ was used to search for microsatellites based on microsatellite sequences with at least 10 nucleotides in the repeat for mono-, 5 for di -, and 3 for tri-, tetra-, penta- or hexanucleotides. Composite microsatellites were also identified. They are formed by multiple, adjacent, repetitive motifs. Hence, a microsatellite is considered composite if it has a maximum interruption of 10 bp between motifs^37,38^.

### Gene Prediction and Functional Annotation

Evidence-driven gene prediction was performed based on gene models of *Arabidopsis thaliana* and *Theobroma cacao* and using the following software: Augustus^39^, GlimmerHMM^40^, GeneMark.hmm^41^, and SNAP^42^*. Ab initio* gene finding was performed with the BRAKER pipeline^43^. Protein homology detection and potential intron resolution were detected by Exonerate software^44^ against the annotated genomes of *Populus trichocarpa*, *Salix purpurea*, *Ricinus communis* and *Manihot esculenta*, downloaded from the Phytozome website^45^. These species are among the plant genomes with the highest number of top hits for *P. edulis*^15^.

Additionally, a *P. edulis* RNA-seq library (see details below) was used to support gene model predictions. PASA^46^ was used to produce alignment assemblies based on overlapping transcript alignments from *P. edulis* RNA-seq data. The results were combined by EVidence Modeler software^47^, and PASA was used to update the EVidence Modeler consensus predictions, adding UTR annotations and models for alternatively spliced isoforms. Exon-intron boundaries were manually examined using GenomeView^48^ and adjusted where necessary.

RNA-seq reads (2 × 100 bp; Illumina HiSeq2000) were trimmed based on quality (Phred quality score >20). Contaminants, remaining adapters, and sequences (<50 bp) were removed using SeqyClean v1.9.9^49^. Total RNA-seq assembly was implemented by Trinity^50^. In brief, RNA-seq reads were derived from three libraries (each replicated three times) of shoot apexes of juvenile, vegetative and reproductive adult plants of *P. edulis*, constructed with the aim of performing comparisons of these three developmental stages (Dornelas M.C. *et al*., unpublished data).

Functional annotation of the predicted gene sequences was performed using Blast2GO v3.2 tools^51^ for assigning ontological terms in accordance with BLASTX results (e-value cut-off of 1 × 10^−6^). In addition, protein signature recognition was performed using the InterProScan tool^52^.

### Microsynteny Analysis

The 20 *P. edulis* BAC-inserts with the highest number of annotated genes were used for the identification of potential microsyntenic regions between *P. edulis* and *Populus trichocarpa* (Salicaceae), and *P. edulis* and *Manihot esculenta* (Euphorbiaceae), two related Malpighiales species with entirely sequenced and well-annotated genomes. *P. edulis* coding sequences were compared with these two genome sequences, available in the Phytozome database^45^ using BLASTN.

Based on the phylogenetic relationships among the Malpighiales species, we chose *P. trichocarpa* because it is the closest species to *P. edulis*. Taxonomically speaking, Passifloraceae appears as a sister group to Salicaceae. On the other hand, *M. esculenta* is the most distant species from *P. edulis* among those Malpighiales with fully sequenced and well-annotated genomes.

To consider two genes as orthologs, the alignment had to show an e-value < 10^−10^ and coverage >50%. After identifying the orthologs, microsyntenic regions were defined. These are regions with more than four pairs of orthologous genes. All gene positions in the microsyntenic regions were recorded to construct comparative graphs. The analysis was carried out on JBrowse, (Phytozome v12.1 platform)^45^ to search for genes exhibiting each *P. edulis* microsyntenic region and in the *P. trichocarpa* and *M. esculenta* genome. The initial and final positions of the orthologous genes and chromosome identification were used as a basis for constructing comparative graphs. Using the GenomeView browser^48^, each of the microsyntenic regions was visualized and confirmed. Finally, comparative graphs were constructed using a graphics application.

## Results

### BAC Selection, Sequencing and Assembly

A total of 66 BAC inserts were selected for complete sequencing based on our previous BAC-end sequencing results^15^, and 46 were selected using probes homologous to transcripts of *P. edulis*^53^ (Supplementary Table S1). Thus, in total, 112 BAC inserts from the *P. edulis* genomic library were sequenced. The sequencing process resulted in 571,565 high quality reads, ranging from 500 to 46,831 bp in length. Sequences were between 24,316 and 142,456 bp in length, corresponding to their respective band sizes resolved by PFGE. The high quality of the long reads (QV > 47) and high coverage of the contigs (on average 278×) are indications of the reliability of our data (Supplementary Table S2), leading to the conclusion that all inserts were completely sequenced and assembled. The assembly, gene models, and genome browser are available at https://genomevolution.org/coge/GenomeInfo.pl?gid=52053.

The sequencing method was of sufficient quality to provide a single contig per insert, with only two exceptions; in the assembly process, insert sequences Pe101K14 and Pe141H13 had overlapping regions that resulted in a single contig of 172,337 bp; similarly, Pe20N3 and Pe64C12 resulted in a single contig of 114,997 bp. In addition, of the 112 BAC insert sequences, three corresponded to organelle DNA, and therefore these sequences were not included. Thus, 107 sequences were subjected to annotation, totaling 10,401,671 bp (10.4 Mb) corresponding to approximately 1.0% of the *P. edulis* genome. GC content across this genome fraction was 41.09%, and in the CDS 46.49%.

### Gene Representativeness, Structure and Functional Annotation

Structural sequence annotation resulted in the prediction of 1,883 genes ranging from 153 to 24,687 bp in length, with an average of 2,448 bp. These gene sequences represented 44% of the total sequenced nucleotides, corresponding to 4,608,830 bp. Intergenic regions covered from 0 (overlapped genes) to 92,497 bp, with a mean length of 3,184 bp. Between 3 and 36 predicted genes were identified per sequenced insert, with an average of 17.6 predicted genes per insert (Table 1, Supplementary Table S3). Taking into account the estimated size of the *P. edulis* genome (~1,230 Mb), the high number of genes identified herein (1,833) endorses the efficiency of the strategy used for selecting BAC-inserts that were supposedly gene-rich.

**Table 1:** Gene content in a gene-rich fraction of the *Pasiflora edulis* genome and structural annotation.

BAC code	No. of predicted genes	Intronless genes	Exons per gene	Gene length variation (bp)	Average gene length (bp)	Intergenic spacer length variation (bp)	Average intergenic spacer length (bp)	CDS length (bp)	Average CDS length (bp)
Pe101K14 + 141H13	36^*^	17	2–17	264–11,778	2,720	33–6,312	2,070	264–6,576	1,187
Pe185D11	36^*^	12	2–12	201–4,778	1,548	16–9,730	1,802	201–1,725	689
Pe164B18	29^*^	9	2–19	243–16,279	2,313	42–7,449	1,316	243–11,409	1,393
Pe214H11	29^*^	4	2–39	799–13,956	3,857	194–5,728	1,134	174–4,572	1,636
Pe164D9	28^*^	13	2–11	198–5,817	1,868	114–5,844	1,600	156–2,202	1,066
Pe186E19	28^*^	4	2–14	770–7,450	2,651	11–13,501	1,559	210–2,307	1,098
Pe43L2	27^*^	3	2–18	339–10,097	2,718	162–2,768	973	279–3,123	1,145
Pe86F9	27	13	2–5	201–20,501	1,622	147–12,507	2,776	201–1,740	607
Pe164K17	26^*^	4	2–13	436–9,502	3,037	11–7,775	1,761	204–5,334	1,310
Pe215I8	26^*^	5	2–18	312–8,238	3,007	230–13,338	2,168	180–3,501	1,253
Pe75K15	25	14	2–5	186–4,193	857	10–11,721	2,951	186–2,100	591
Pe84I14	25^*^	6	2–12	345–8,118	3,014	69–4,352	936	198–4,275	1,295
Pe84M23	25	5	2–13	305–8,652	2,753	52–5,197	998	177–3,018	1,168
Pe93M2	25^*^	5	2–16	399–7,069	2,274	135–11,933	2,170	192–2,961	1,109
Pe171P13	25^*^	8	2–20	461–9,727	2,759	158–15,960	2,392	330–4,035	1,193
Pe207D11	25^*^	12	2–17	213–6,756	1,896	5–20,551	2,838	213–2,730	897
Pe93N7	24^*^	5	2–11	921–8,889	3,120	18–7,588	1,421	387–5,085	1,486
Pe108C16	24^*^	8	2–14	234–6,553	1,892	34–9,113	2,209	234–3,252	974
Pe173B16	24^*^	6	2–32	475–15,390	3,079	151–15,127	2,134	279–6,375	1,523
Pe185J16	24^*^	4	2–21	447–8,773	2,432	201–6,924	2,083	237–2,367	1,035
Pe198H23	24^*^	8	2–6	180–5,279	1,943	1–11,008	2,681	180–3,510	1,143
Pe212I1	24^*^	5	2–35	234–12,694	2,715	53–15,133	2,607	234–3,567	1,080
Pe93J9	23	3	2–16	615–6,131	2,907	3–9,066	1,824	201–3,321	1,295
Pe135J12	23	6	2–15	162–9,543	2,714	81–8,758	1,868	162–4,433	1,260
Pe195F4	23	2	2–20	261–8,364	2,843	9–11,133	2,208	177–5,442	1,192
Pe74I6	22	9	2–39	204–17,655	3,407	146–6,191	1,764	204–4,374	1,164
Pe84M18	22	6	2–10	321–8,124	2,563	22–15,224	2,364	321–4,356	1,160
Pe101O4	22	6	2–19	624–9,702	2,678	315–10,499	2,782	300–2,235	884
Pe141J23	22	6	2–15	189–9,258	2,567	608–12,079	2,407	189–2,550	870
Pe201C11	22	11	2–17	195–5,452	1,865	288–17,891	4,128	195–2,634	822
Pe69G18	21	3	2–22	228–8,658	2,958	61–19,104	2,304	210–3,582	1,192
Pe69H24	21	2	2–14	335–6,461	2,752	445–5705	2,306	234–2,559	1,142
Pe93K19	21	3	2–12	792–10,373	3,523	196–6,322	1,422	387–4,629	1,593
Pe125I23	21	5	2–14	414–7,993	2,526	51–8,659	2,406	414–1,776	1,106
Pe164A12	21	7	2–11	384–7,964	2,354	26–7,406	1,675	228–4,503	1,050
Pe168B17	21	3	2–11	321–6,861	2,509	47–16,932	4,619	174–4,140	1,234
Pe214A18	21	7	2–11	243–6,314	1,944	237–27,586	3,916	243–2,184	924
Pe7M15	20	11	2–15	213–9,031	2,388	12–17,420	3,676	213–3,495	1,046
Pe28D11	20	16	2–4	189–2,430	780	22–28,073	5,567	189–1,410	558
Pe60G10	20	6	2–24	351–9,925	2,513	91–10,947	2,767	261–3,378	1,291
Pe65F7	20	8	2–14	306–7,081	1,973	12–25,539	2,702	213–3,252	844
Pe175N8	20	8	2–27	219–14,245	2,941	15–11,237	2,495	219–3,663	1,299
Pe214N19	20	9	2–13	234–5,913	1,594	37–15,598	3,485	189–2,470	788
Pe43D2	19	3	2–8	447–7,338	2,601	271–19,633	2,158	222–4,872	1,120
Pe51C2	19	5	2–16	357–8,889	3,603	493–6,756	2,110	357–5,088	1,520
Pe85B19	19	7	2–18	372–10,115	2,851	42–8,103	2,368	183–3,228	1,157
Pe101P7	19	3	2–20	234–8,484	3,742	16–2,340	963	234–2,712	1,247
Pe134H15	19	8	2–11	295–7,290	2,527	208–5,953	2,351	219–1,899	844
Pe216F3	19	2	2–37	393–14,151	3,198	241–3,160	914	393–8,943	1,626
Pe216F9	19	5	2–13	207–9,274	3,547	420–5,573	2,107	207–3,417	1,180
Pe20N3 + Pe64C12	18	5	2–12	441–6,941	2,557	266–10,519	2,009	276–2,364	1,223
Pe24G19	18	12	2–6	165–3,803	1,054	184–22,176	3,639	165–1,593	598
Pe69C7	18	7	2–22	210–8,505	3,745	132–18,029	2,165	210–4,164	1,450
Pe69O16	18	4	4–19	590–17,670	4,339	86–1,976	767	177–14,583	2,292
Pe212D7	18	7	2–36	171–21,131	2,654	415–20,035	4,436	171–9,330	1,229
Pe27H17	17	13	2–3	177–2,134	620	197–13,511	4,390	177–1,071	464
Pe85I9	17	5	2–12	207–8,578	2,908	334–20,210	2,892	207–1,956	1,107
Pe89E10	17	10	2–13	183–4,327	974	342–18,584	5,178	174–1,794	509
Pe101P13	17	4	2–21	666 –13,552	4,437	90–4,941	1,072	210–2,307	1,261
Pe209G15	17	3	2–14	219–8,353	3,108	118–17,105	2,754	219–3,084	1,416
Pe21O15	16	7	2–13	189–4,570	1,512	106–14,572	3,633	156–1,902	595
Pe63J18	16	10	2–5	441–6,941	2,750	266–10,519	2,054	213–3,429	970
Pe84K8	16	3	2–18	162–12,356	3,570	178–4,867	1,891	162–2,295	1,072
Pe93M4	16	10	2–7	216–3,063	972	15–37,508	4,704	216–1,998	640
Pe117C17	16	11	2––12	153–6,852	979	7–18,168	5,302	153–1,188	414
Pe138G17	16	10	2–10	178–6,113	1,395	40–13,394	4,513	178–2,934	731
Pe141K8	16	4	2–24	1,053–11,592	4,060	283–5,091	2,179	387–3,975	1,653
Pe216B22	16	1	2–15	1013–8,815	3,931	47–19,862	3,119	795–3,768	1,575
Pe216I5	16	6	4–16	201–5,929	3,296	462–4,563	1,373	201–2,862	1,458
Pe61E2	15	4	3–12	231–8,598	3,100	223–18,187	3,244	231–2,103	973
Pe99P16	15	9	2–33	249–15,022	2,441	501–9,387	2,582	216–4,605	908
Pe123N8	15	5	2–22	163–10,051	2,938	70–13,306	39,979	163–2,397	1,028
Pe3F10	14	4	2–14	652–6,552	2,471	90–4,389	1,557	285–3,252	1,080
Pe28E22	14	1	2–12	379–11,107	3,661	13–16,073	2,221	261–2,718	1,247
Pe34M7	14	6	2–4	225–1,298	652	82–39,701	6,611	192–1,026	459
Pe75F20	14	6	2–13	198–6,418	1,859	182–21,979	5,567	198–1,842	541
Pe85H4	14	1	2–51	489–22,481	3,938	178–17,578	2,764	300–5,706	1,546
Pe85J23	14	2	2–15	760–9,631	3,222	362–9,609	2,597	492–3,066	1,087
Pe101H15	14	10	2–5	225–24,687	2,257	122–15,195	6,521	255–1,008	524
Pe69F22	13	0	2–14	438–6,597	3,680	196–26,118	4,433	207–1,710	1,029
Pe75A21	13	8	3–10	162–5,730	1,569	10–15,569	4,038	162–2,076	630
Pe84M6	13	8	2–13	185–3,026	1,059	262–16,455	4,686	185–1,578	792
Pe86H7	13	7	2–3	213–4,497	1,429	31–28,575	6,964	213–3,459	875
Pe34H9	12	3	2–14	258–6,285	1,961	49–44,532	6,154	258–1,623	748
Pe213C9	12	8	2–5	327–3,599	1,246	213–31,653	7,880	234–2,016	749
Pe71E3	11	2	3–9	207–3,727	2,185	362–31,489	6,138	207–1,698	1,047
Pe93A7	11	8	2–4	162–1,374	582	18–25,472	7,604	162–759	373
Pe93F5	11	2	2–8	192–11,041	2,745	5 – 24,167	7,152	192–1722	707
Pe93O18	11	3	2–11	387–7,643	2,714	596–49,482	9,337	387–1,632	1,080
Pe101F21	11	7	2–7	243–4,835	947	58–27,172	8,438	198–1,806	534
Pe141B12	11	4	2–15	288 – 6,769	2,412	251–24,611	5,214	282–3,417	1,142
Pe75D12	10	6	2–5	219–3,255	778	109–39,945	8,052	216–1,224	456
Pe75N15	10	8	2	204–714	444	78–32,243	7,353	204–714	402
Pe9E4	9	4	2 – 14	342–6,100	2,099	654 – 13,925	6,177	342–2,898	1,171
Pe15E1	9	4	2–13	270–2,896	1,153	700–33,021	9,014	270–1,578	714
Pe20E10	9	4	2–2	159–1,578	605	278–35,112	9,958	159–1,578	496
Pe212M5	9	4	2–6	267–3,170	1,020	851–10,468	4,056	267–1,566	727
Pe103M2	8	2	2–17	222–12,656	3,122	418–32,453	6,547	222–2,010	807
Pe28I20	7	5	2–2	237–881	467	67–30,516	11,363	237–762	437
Pe75F13	7	4	2–3	180–1,636	654	16,743–92,497	58,535	180–1,245	519
Pe85O9	7	1	2–8	441–3,324	2,079	515–6,447	1,784	441–1,329	765
Pe1M17	6	1	2–4	312–2,473	1,099	256–10,848	5,311	312–1,404	784
Pe212J12	6	2	2–15	405–4,357	1,377	81–12,708	3,133	381–1,644	692
Pe216B2	6	1	2–24	218–15,969	5,097	830–4,575	2,306	218–3,819	1,605
Pe113A7	5	3	2	156–2254	1,206	3472–26,026	13,464	156–681	503
Pe1K19	3	0	2–9	958–4,737	3,111	287–37,487	18,877	840–897	869
Pe33M2	3	2	3	210–2,037	824	4,001–69,199	36,600	210–697	377

^*^BAC-inserts with the highest number of annotated genes, used for microsynteny analysis.

One third of the genes (631) had no introns. The remaining (1,252) had up to 50 introns. A total of 6,122 introns (ranging from 26 to 7,869 bp in length) and 8,005 exons (ranging from 3 to 6,249 bp) were recognized. CDS ranged from 153 to 14,583 bp in length, totaling 1,985,892 bp, with a mean of 1,054 bp. A total of 61 were insert-end sequences and therefore incomplete gene sequences. According to the RNA-seq read alignment results, 252 genes exhibited more than one transcript (Supplementary Table S3), including glutamine synthetase leaf enzyme, chloroplastic (6 transcripts), ultraviolet-B receptor UVR8, a protein responsive to UV-B (5), the auxin response factor (2), an abscisic acid insensitive protein (2) and an ethylene receptor protein (2).

**Figure 1:**
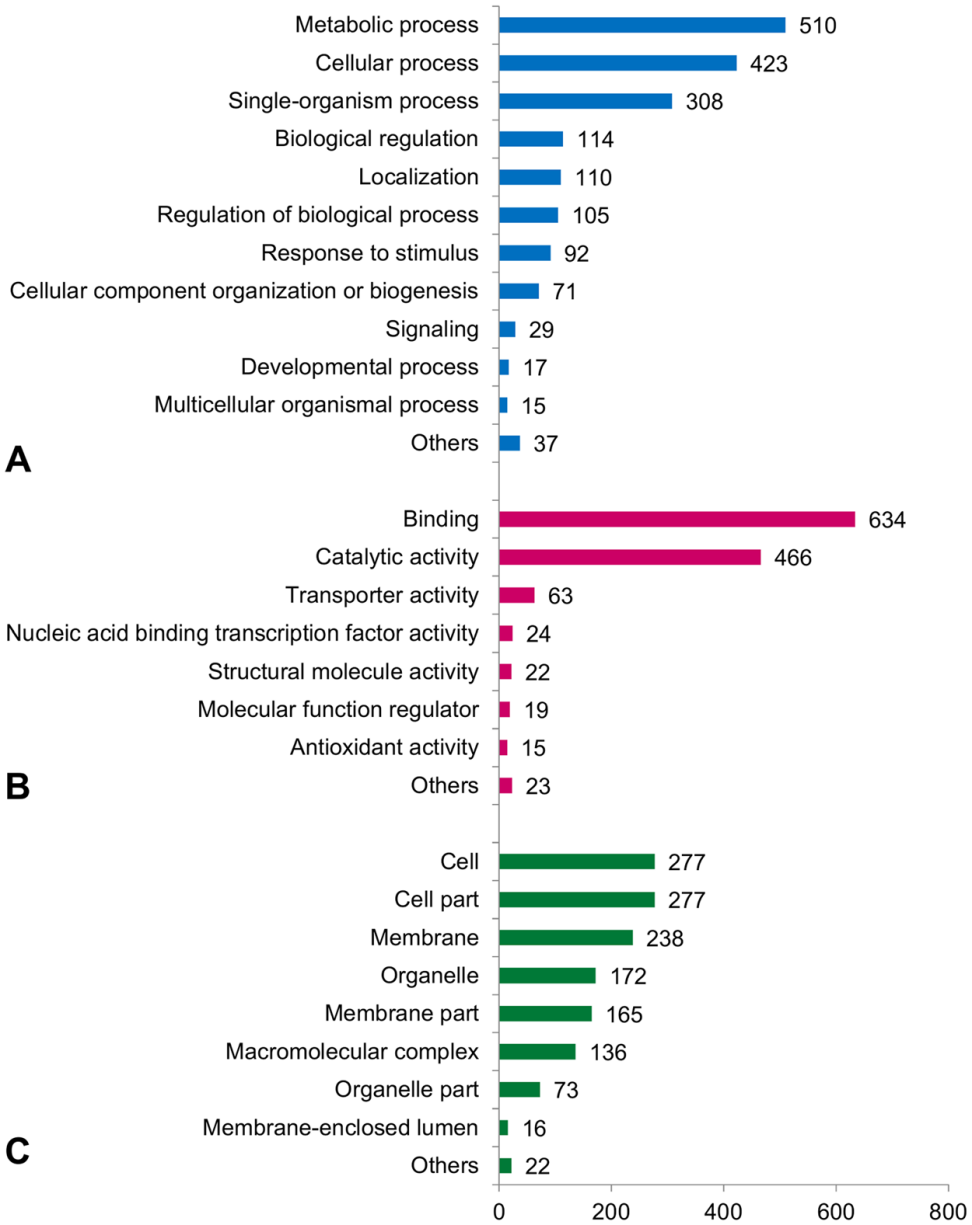
Distribution of GO annotations assigned to gene products in ontological categories: (**A**) Biological process, (**B**) Molecular function and (**C**) Cellular component. GO annotations were extracted from all sequences (10.4 Mb) of *Passiflora edulis*.

Of the 1,883 predicted genes, 1,502 showed significant levels of similarity (e-values < 1 × 10^−6^) to plant proteins (Supplementary Table S3) according to the Blast2GO results. The top hits for this large fraction of genes (~80%) were from *Jatropha curcas* (298), *Populus trichocarpa* (275), *Populus euphratica* (232) and *Ricinus communis* (212). These results were expected, since among all available plant genomes, these species are phylogenetically close to *P. edulis*, and all belong to the Malpighiales order. Functional annotation resulted in 3,178 ontological terms assigned to 1,191 genes. These GO terms were related to several processes, and are usually classified into three broad categories (known as level 1): biological process, molecular function and cellular component. The distribution of level 2 terms within each of these major categories is shown in Fig. 1 and matches the results of BES annotation^15^.

Regarding the 46 regions selected using probes homologous to transcripts induced and repressed by *X. axonopodis* infection, none of the functional categories related to plant defense were found to be overrepresented. However, protein signatures related to plant immunity and defense functions were identified. The serine/threonine-protein kinase active site (32 genes), and the leucine-rich repeat domain, L domain-like (27 genes) were among the most represented signatures (Table 2). In total, automated searches for protein signatures recognized 1,383 signatures in 1,488 genes of *P. edulis*: 783 domains, 453 protein families, 125 sites and 22 replicates (Table 2). Most of these signatures (769) were taken from the Pfam database^54^, and the remainder from SuperFamily (239)^55^ and Smart (223)^56^.

**Table 2:** Most frequent protein signatures (≥10) recognized in genes of *Passiflora edulis* according to InterProScan results.

InterProScan ID	No. of genes
IPR005162 [Domain]: Retrotransposon gag domain	58
IPR011009 [Domain]: Protein kinase-like domain	51
IPR000719 [Domain]: Protein kinase domain	49
IPR027417 [Domain]: P-loop containing nucleoside triphosphate hydrolase	39
IPR001878 [Domain]: Zinc finger, CCHC-type	36
IPR011990 [Domain]: Tetratricopeptide-like helical domain	34
IPR008271 [Active_Site]: Serine/threonine-protein kinase, active site	32
IPR013083 [Domain]: Zinc finger, RING/FYVE/PHD-type	31
IPR029058 [Domain]: Alpha/Beta hydrolase fold	30
IPR017441 [Binding_Site]: Protein kinase, ATP binding site	30
IPR016024 [Domain]: Armadillo-type fold	27
IPR032675 [Domain]: Leucine-rich repeat domain, L domain-like	27
IPR013320 [Domain]: Concanavalin A-like lectin/glucanase domain	25
IPR009057 [Domain]: Homeodomain-like	25
IPR002885 [Repeat]: Pentatricopeptide repeat	25
IPR011989 [Domain]: Armadillo-like helical	22
IPR016040 [Domain]: NAD(P)-binding domain	19
IPR013242 [Domain]: Retroviral aspartyl protease	19
IPR001841 [Domain]: Zinc finger, RING-type	19
IPR017986 [Domain]: WD40-repeat-containing domain	18
IPR012337 [Domain]: Ribonuclease H-like domain	18
IPR015943 [Domain]: WD40/YVTN repeat-like-containing domain	18
IPR001128 [Family]: Cytochrome P450	17
IPR001611 [REPEAT] - Leucine-rich repeat	17
IPR012677 [Domain]: Nucleotide-binding alpha-beta plait domain	16
IPR001680 [Repeat]: WD40 repeat	16
IPR001005 [Domain]: SANT/Myb domain	15
IPR029044 [Domain]: Nucleotide-diphospho-sugar transferases	15
IPR026960 [Domain]: Reverse transcriptase zinc-binding domain	15
IPR017853 [Domain]: Glycoside hydrolase superfamily	15
IPR000504 [Domain]: RNA recognition motif domain	14
IPR013210 [Domain]: Leucine-rich repeat-containing N-terminal, plant-type	14
IPR001245 [Domain]: Serine-threonine/tyrosine-protein kinase catalytic domain	14
IPR018247 [Binding_Site]: EF-Hand 1, calcium-binding site	13
IPR005135 [Domain]: Endonuclease/exonuclease/phosphatase	13
IPR011598 [Domain]: Myc-type, basic helix-loop-helix (bHLH) domain	13
IPR011992 [Domain]: EF-hand domain pair	13
IPR002401 [Family]: Cytochrome P450, E-class, group I	13
IPR005123 [Domain]: Oxoglutarate/iron-dependent dioxygenase	12
IPR002048 [Domain]: EF-hand domain	12
IPR012334 [Domain]: Pectin lyase fold	11
IPR013781 [Domain]: Glycoside hydrolase, catalytic domain	11
IPR011050 [Domain]: Pectin lyase fold/virulence factor	11
IPR017930 [Domain]: Myb domain	11
IPR017972 [Conserved_Site]: Cytochrome P450, conserved site	11
IPR006121 [Domain]: Heavy metal-associated domain, HMA	10
IPR001810 [Domain]: F-box domain	10
IPR000620 [Domain]: EamA domain	10
IPR012336 [Domain]: Thioredoxin-like fold	10
IPR016140 [Domain]: Bifunctional inhibitor/plant lipid transfer protein/seed storage helical	10
IPR025558 [Domain]: Domain of unknown function DUF4283	10

### Richness of Transposable Elements and Microsatellites

The search for transposable elements resulted in the identification of 250 TEs that, in turn, were automatically classified as Class I (retrotransposons) and Class II (DNA transposons), and in terms of order^33^. These TEs represented 17.6% of total data, corresponding to 1,830,620 bp. Class I was prevalent with 96.4% (241/250) retrotransposons (Table 3). These TEs were preferentially hosted in intergenic regions (70.4%, 176/250); 74 TEs were found within genes, including 70 exonic TEs, and only four were located in introns.

**Table 3:** Classification of transposable elements identified in a gene-rich fraction of the *Pasiflora edulis* genome.

Class	Number of elements	Percentage of nucleotides^*^
Class I total (RXX)	241	17.15
DIRS total (RYX)	11	1.11
*DIRS incomplete*	11	
*DIRS potential chimeric*	11	
LINE total (RIX)	7	0.52
*LINE complete*	3	
*LINE incomplete*	4	
*LINE potential chimeric*	6	
LTR total (RLX)	181	13.64
*LTR complete*	73	
*LTR incomplete*	108	
*LTR potential chimeric*	36	
SINE total (RSX)	2	0.01
*SINE incomplete*	2	
LARD total (RXX-LARD)	36	1.82
*LARD potential chimeric*	2	
TRIM total (RXX-TRIM)	4	0.05
Classe II total (DXX)	9	0.45
Helitron total (DHX)	2	0.13
*Helitron complete*	2	
TIR total (DTX)	6	0.31
*TIR incomplete*	6	
*TIR potential chimeric*	1	
MITE total (DXX-MITE)	1	0.01
Total	250	17.60

^*^Percentage of nucleotides in 10.4 Mb of *P. edulis* sequences.

The LTR (Long Terminal Repeat) retrotransposon was the most frequent order, and accounted for 75.1% (181/241) of retrotransposons, corresponding to 1,418,389 bp or 13.6% (1,418,389 bp/10,401,671 bp) of all sequence data. The other orders of Class I were poorly represented, but note that LARDs (Large Retrotransposon Derivatives) accounted for 36 elements (Table 3). Only 3.6% (9/250) of TEs were of Class II, the majority (6) classified as TIR (Terminal Inverted Repeats) (Table 3).

The search for microsatellites resulted in the identification of 11,020 simple sequence repeats (SSR), representing 1.05% of all sequence data (109,695 bp/10,401,671 bp). In CDS (1,985,806 bp) there were 1,762 SSRs (~16% of the total). Taking into account all sequence data, 106 SSRs were found every 100 kb (one SSR every 0.94 kb). Analyzing the CDS region, 89 SSRs were found every 100 kb (one SSR every 1.12 kb); hence, the frequency of SSRs was slightly lower in the CDS region (~1.2×, 1.12 kb/0.94 kb). Our estimates were 10× lower than those reported in^15^ using *P. edulis* BES data as a major resource (10.8 SSRs every 100 kb or one SSR every 9.25 kb).

**Figure 2:**
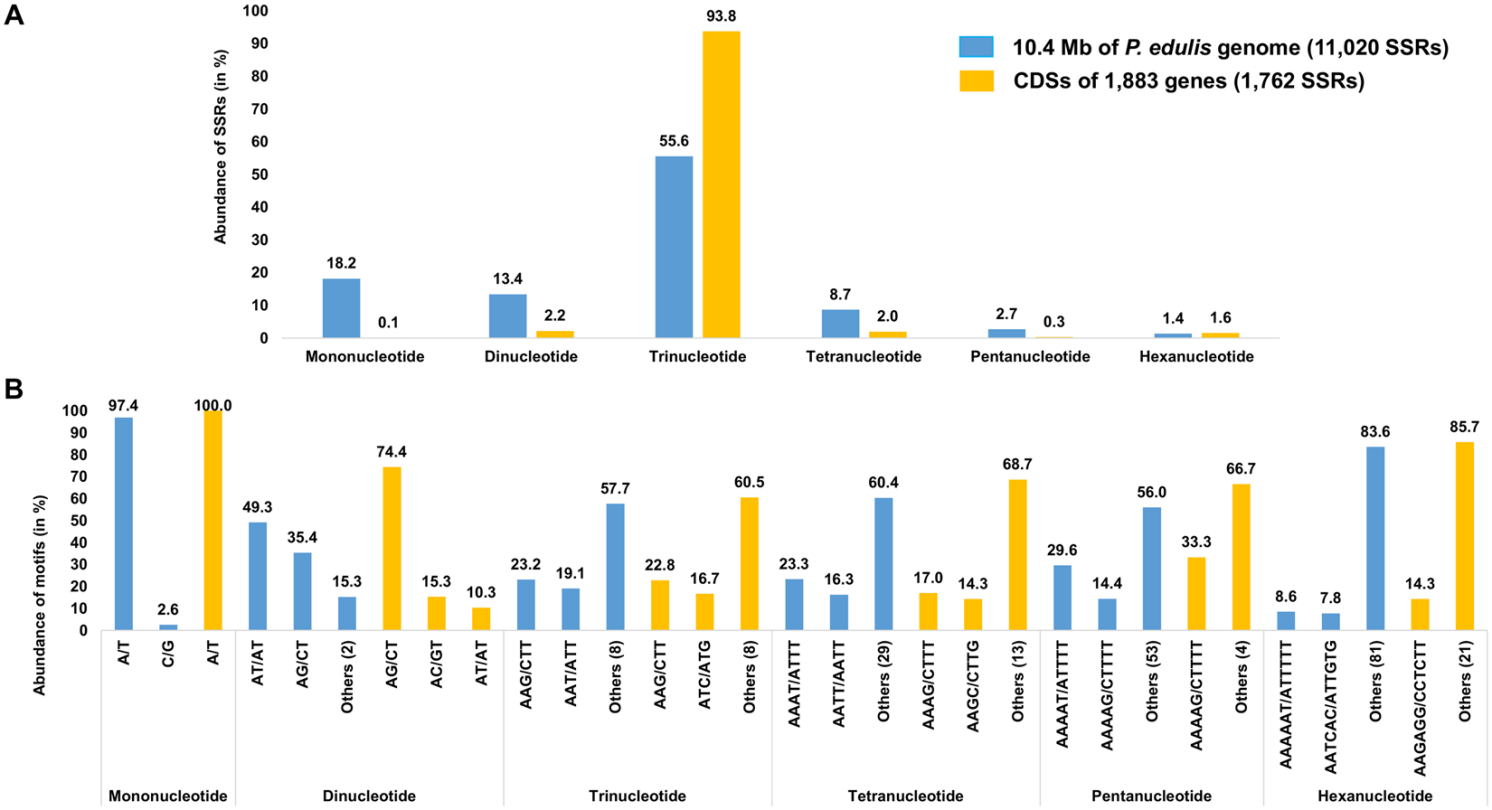
(**A**) Percentage of mono-, di-, tri-, tetra-, penta- and hexanucleotides in microsatellites (SSRs) found in all sequences (10.4 Mb) of *Passiflora edulis* (blue bars) and in coding DNA sequences (CDS, orange bars). (**B**) Percentage of the most frequent motifs in each class of microsatellites (SSRs) found in all sequences (blue bars) and in coding DNA sequences (CDS, orange bars) of *Passiflora edulis*.

Microsatellite sequences were grouped according to motif, and all possible classes of repeats were found, with trinucleotides the most prevalent in both data sources. Compound SSRs accounted for 17.4% (1,919/11,020) of all SSRs, and 15.7% (278/1,762) of these SSRs were found in CDS (Fig. 2A). Among the mononucleotides, the A/T motif far surpassed the number of G/C motifs. The most frequent dinucleotides were AT/AT (49.3%), followed by AG/CT (35.4%), which were prevalent in CDS (74%). Among the trinucleotides, AAG/CTT were the most frequent in both data sources (~23%). Other occurrences (tetra-, penta- and hexanucleotides) are shown in Fig. 2B.

### Microsynteny Analysis Results

The following 20 *P. edulis* BAC-inserts were used for microsynteny analysis: Pe101K14 + 141H13 (36), Pe185D11 (36), Pe164B18 (29), Pe214H11 (29), Pe164D9 (28), Pe186E19 (28), Pe43L2 (27), Pe164K17 (26), Pe215I8 (26), Pe84I14 (25), Pe84M23 (25), Pe93M2 (25), Pe171P13 (25), Pe207D11 (25), Pe93N7 (24), Pe108C16 (24), Pe173B16 (24), Pe185J16 (24), Pe198H23 (24) and Pe212I1 (24). These regions were found to contain the highest number of annotated genes (given in parenthesis) and account for 2,243,840 bp, encompassing 534 genes (Table 1).

Microsynteny analysis showed that 18 of the 20 *P. edulis* regions contained syntenic *P. trichocarpa* chromosomal regions, and 15 *P. edulis* regions had syntenic *M. esculenta* chromosomal regions (Figs 3−7, S1−S13). In some comparisons, the microsyntenic region of *P. edulis* had the opposite orientation with respect to the chromosomes of both (see Fig. 3) or one of the species compared.

**Figure 3:**
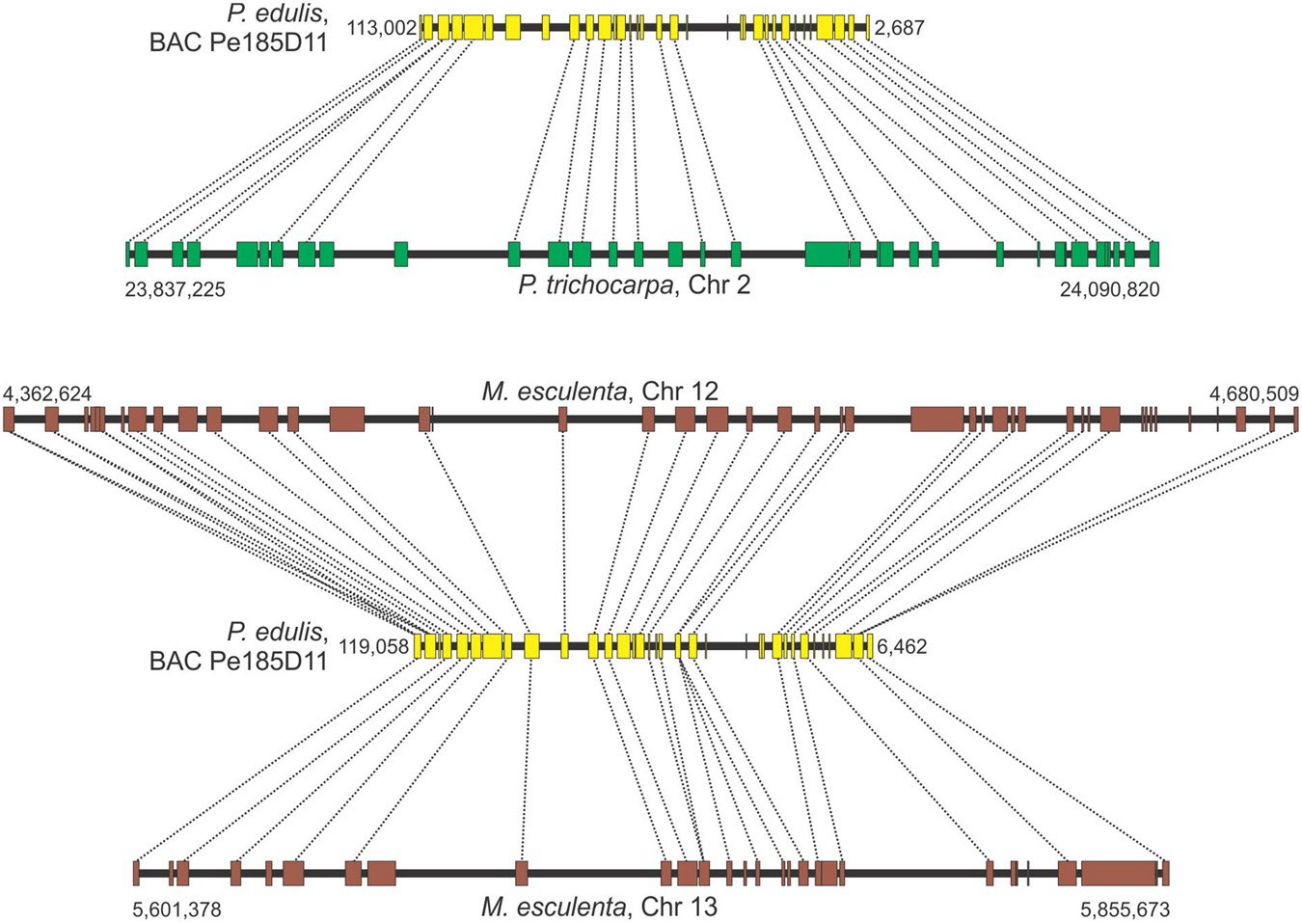
Collinear microsyntenic regions identified in *Passiflora edulis* (yellow bars) and *Populus trichocarpa* chromosome 2 (green bar) and *Manihot esculenta* chromosomes 12 and 13 (brown bars). Note the opposite orientation of the *P. edulis* microsyntenic region relative to the chromosomes of both species. The orthologous genes of *P. edulis* are duplicated in *M. esculenta* chromosomes.

**Figure 4:**
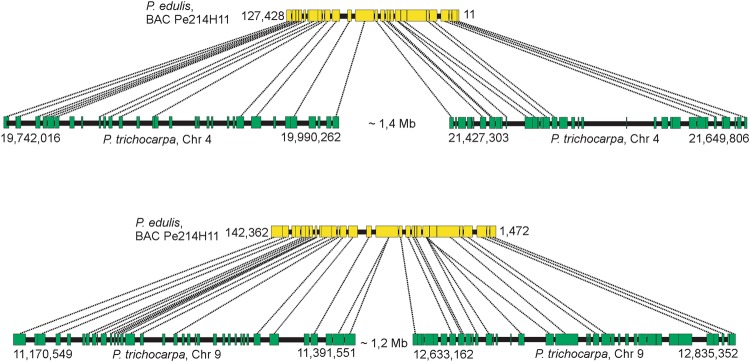
Collinear microsyntenic regions identified in *Passiflora edulis* (yellow bars) and *Populus trichocarpa* chromosomes 4 and 9 (green bars). Note the opposite orientation of *P. edulis* microsyntenic region. The orthologous genes of *P. edulis* are duplicated in *P. trichocarpa* chromosomes.

**Figure 5:**
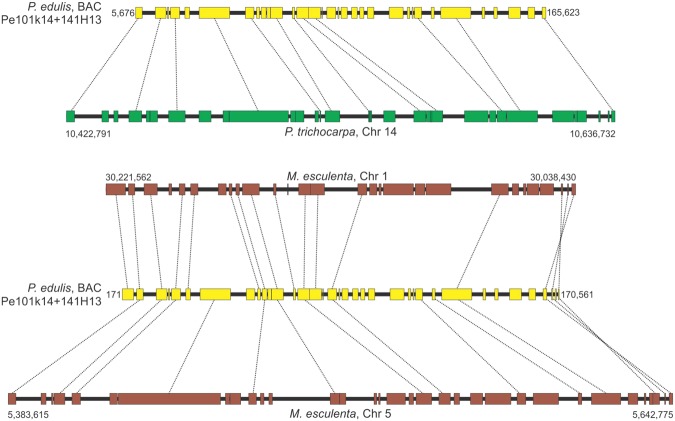
Collinear microsyntenic regions identified in *Passiflora edulis* (yellow bars) and *Populus trichocarpa* chromosome 14 (green bar) and *Manihot esculenta* chromosomes 1 and 5 (brown bars). Note the opposite orientation of *M. esculenta* chromosome 1, and rearranged segments at the end of the *P. edulis* microsyntenic region. The orthologous genes of *P. edulis* are duplicated in *M. esculenta* chromosomes.

**Figure 6:**
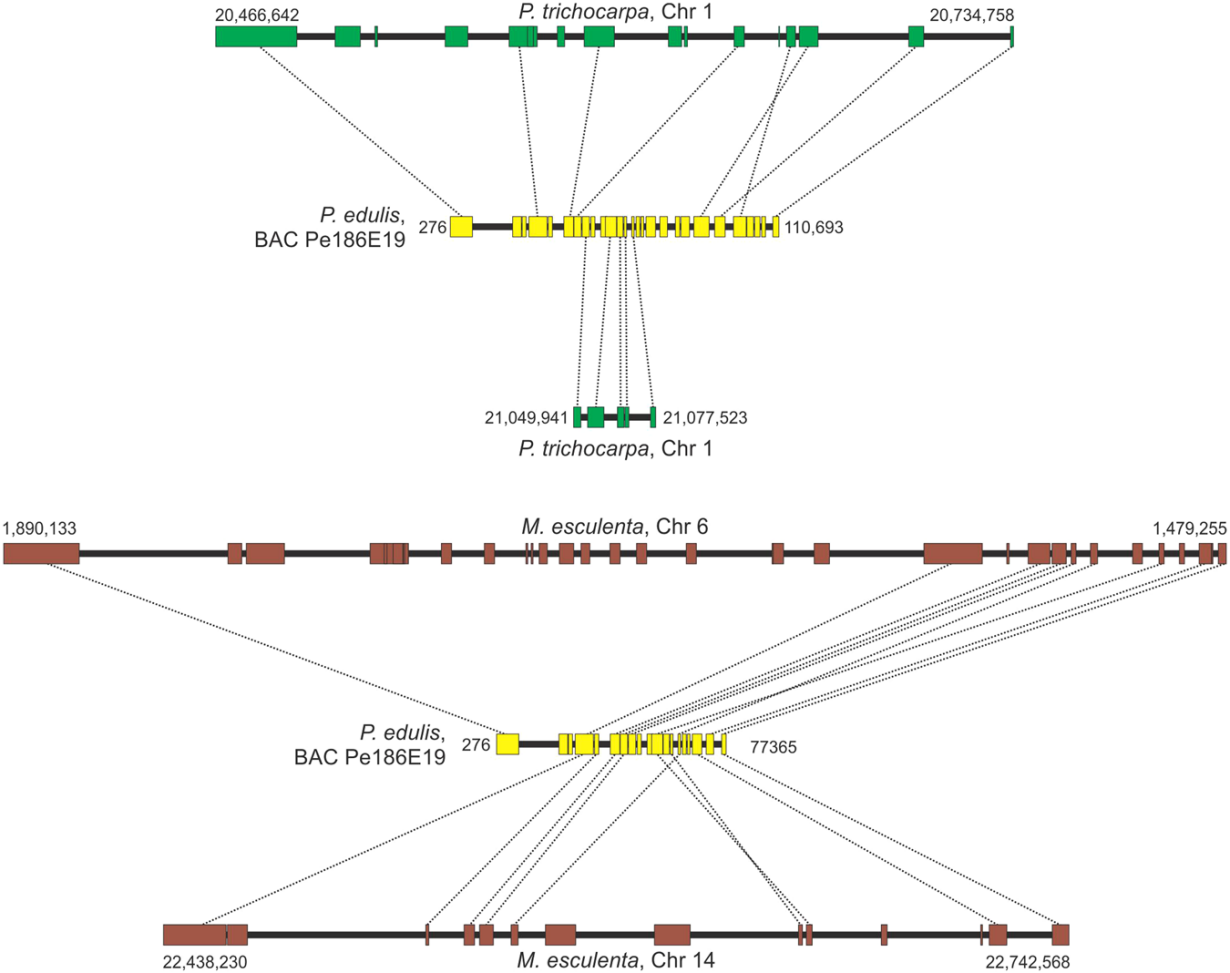
Collinear microsyntenic regions identified in *Passiflora edulis* (yellow bars) and *Populus trichocarpa* chromosome 1 (green bars) and *Manihot esculenta* chromosome 6 and 14 (brown bars). Note the opposite orientation of *M. esculenta* chromosome 6. There are translocated segments in the *P. edulis* microsyntenic region relative to chromosome 1 of *P. trichocarpa*. The orthologous genes of *P. edulis* are duplicated in *M. esculenta* chromosomes.

**Figure 7:**
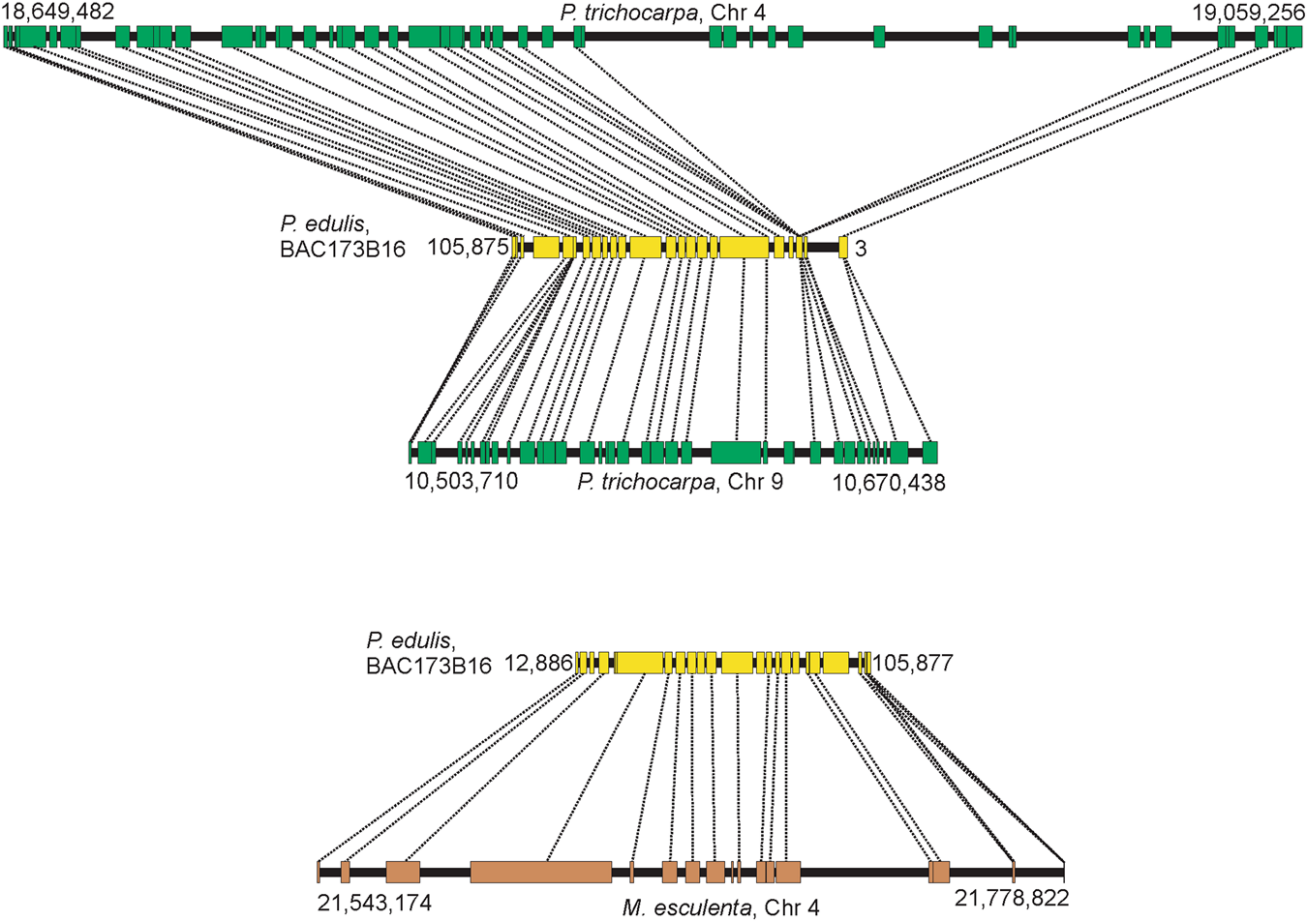
Collinear microsyntenic regions identified in *Passiflora edulis* (yellow bars) and *Populus trichocarpa* chromosomes 4 and 9 (green bars) and *Manihot esculenta* chromosome 4 (brown bar). Note the opposite orientation of the *P. edulis* microsyntenic region relative to *P. trichocarpa* chromosomes, and the large segment of *P. trichocarpa* chromosome 4 that is missing in *P. edulis*. The orthologous genes of *P. edulis* are duplicated in *P. trichocarpa* chromosomes.

The 18 *P. edulis* regions span 1,702,975 bp and contain 406 genes. They matched syntenic segments of *P. trichocarpa* chromosomes that span 7,137,451 bp and contain 966 genes, including 501 orthologs (Table 4). Ten of the syntenic regions of *P. edulis* have orthologous genes that are duplicated in *P. trichocarpa* chromosomes. Interestingly, a continuous region in *P. edulis* (Pe214H11) is syntenic to segments of *P. trichocarpa* chromosome 4, and these segments are separated by 1.4 Mb. The same is true for segments of chromosome 9, separated by 1.2 Mb (Fig. 4). Other large segments of the *P. trichocarpa* chromosome 4 are also missing in the corresponding *P. edulis* syntenic region (Fig. 7). These presumably relate to deletion events that occurred in *P. edulis*.

**Table 4:** Characterization of 18 *Passiflora edulis* regions found to have syntenic *Populus trichocarpa* chromosomal regions.

*Passiflora edulis*			*Populus trichocarpa*		
BAC code	Insert length (bp)	Syntenic region length (bp)	Syntenic region length (bp)	Chromosome	Number of orthologous genes
Pe101K14 + 141H13	172,337	159,949	213,942	14	12
Pe108C16	96,753	68,880	137,749	6	16
		65,309	130,229	18	13
Pe164B18	104,102	103,945	369,800	4	20
		103,945	189,230	9	18
Pe164D9	93,527	80,789	430,901	4	27
		85,112	209,253	17	26
Pe164K17	113,504	113,313	332,637	14	23
		110,607	307,065	2	16
Pe171P13	111,123	85,809	340,005	7	12
Pe173B16	109,801	105,875	409,775	4	28
		105,875	166,729	9	29
Pe185D11	119,061	110,316	253,596	2	22
Pe185J16	103,095	47,587	231,419	12	10
Pe186E19	115,218	17,442	27,583	1	5
		92,977	268,117	1	8
Pe207D11	111,690	31,090	122,497	1	8
Pe212I1	121,384	85,114	162,212	2	14
		85,114	169,126	5	13
Pe214H11	142,456	79,416	221,003	9	17
		64,482	248,247	4	14
		60,720	202,191	9	13
		62,181	222,504	4	11
Pe215I8	129,737	79,415	166,694	1	12
Pe84I14	97,848	93,065	141,647	14	13
Pe84M23	93,217	92,795	171,100	2	15
		89,339	206,947	5	12
Pe93M2	100,436	98,828	199,350	12	17
		88,334	207,961	15	18
Pe93N7	106,968	105,007	340,655	6	23
		99,896	337,287	18	16
Total	2,042,257	1,702,975^*^	7,137,451		501

^*^Non-redundant data.

Average gene length in *P. edulis* (2,785 bp) is slightly lower than that of *P. trichocarpa* (3,290 bp). However, the average intergenic spacer length in *P. trichocarpa* (8,694 bp) is four times that of *P. edulis* (1,871 bp) (Supplementary Table S4). The gene order is conserved in most of the syntenic regions, but rearrangements were observed. On comparing *P. edulis* with *P. trichocarpa*, two typical inversion events in the gene order were recognized (Supplementary Figs S3 and S6). Moreover, two adjacent genes in *P. trichocarpa* chromosome 1 were found to be inverted, and also interrupted in the *P. edulis* syntenic region (Fig. 6). Finally, it is worth noting the occurrence of particular gene duplications within the syntenic regions involving two to seven copies. Figure 4 shows two *P. edulis* genes (8^th^ and 22^nd^) that have four copies in *P. trichocarpa* chromosome 9.

In the comparison with *M. esculenta*, the 15 regions of *P. edulis* span 1,392,795 bp and contain 348 genes, matching syntenic segments of *M. esculenta* chromosomes that span 5,053,254 bp and contain 633 genes, including 365 orthologs (Table 5). Eleven of the syntenic regions of *P. edulis* contain orthologous genes that are duplicated in *M. esculenta* chromosomes.

**Table 5:** Characterization of 15 *Passiflora edulis* regions found to have syntenic *Manihot esculenta* chromosomal regions.

*Passiflora edulis*			*Manihot esculenta*		
BAC code	BAC length (bp)	Syntenic region length (bp)	Syntenic region length (bp)	Chromosome	Number of orthologous genes
Pe101K14-141H13	172,337	170,391	183,133	1	16
		164,887	259,161	5	14
Pe108C16	96,753	68,880	76,043	3	10
		63,474	88,458	16	10
Pe164B18	104,102	103,945	182,720	17	17
		103,945	345,243	15	12
Pe164D9	93,527	93,489	206,242	2	25
		85,112	118,187	1	15
Pe164K17	113,504	101,996	189,788	1	11
		110,607	393,258	5	17
Pe173B16	109,801	92,992	235,649	4	20
Pe185D11	119,061	110,279	317,886	12	27
		112,597	254,296	13	18
Pe185J16	103,095	88,563	308,705	1	12
Pe186E19	115,218	50,679	304,339	14	9
		50,679	101,361	6	8
Pe207D11	111,690	28,902	48,780	15	6
Pe212I1	121,384	85,114	172,143	18	14
Pe215I8	129,737	118,786	162,363	17	14
		124,698	193,725	15	14
Pe84I14	97,848	96,433	135,657	1	12
		94,441	211,686	5	14
Pe84M23	93,217	66,677	148,682	18	13
		53,520	137,511	2	8
Pe93M2	100,436	98,828	126,299	6	17
		78,587	151,939	14	12
TOTAL	1,681,710	1,392,795^*^	5,053,254		365

^*^Non-redundant data.

The average *P. edulis* gene length (2,641 bp) is slightly lower than that of *M. esculenta* (3,886 bp). However, the average intergenic spacer length (6,777 bp) was three times that of *P. edulis* (1,850 bp) (Supplementary Table S4). Gene order is also conserved in most of the syntenic regions, but rearrangements were recognized in genes of both *P. edulis* and *M. esculenta* (Figs S1, S2, S6, S7). The occurrence of particular gene duplications within syntenic regions involving two to five copies was also detected. Figure 3 shows three copies of a *P. edulis* gene (18^th^) arranged in tandem on chromosome 13 of *M. esculenta* and two copies in tandem on chromosome 12, totaling 5 copies. The 2^nd^ gene within the *P. edulis* microsyntenic region is also duplicated in *M. esculenta* chromosome 12.

In terms of specific genes, note that a single copy of the gene encoding a KIN1-related stress-induced protein was found in *P. edulis* but there are seven orthologous copies in *P. trichocarpa* chromosome 4 and three in chromosome 17 (Supplementary Fig. S2). Moreover, five copies in tandem of the gene encoding an endo-1,3 1,4-beta-D-glucanase were found in *P. edulis*, but no orthologs were found in *P. trichocarpa* and *M. esculenta*. Finally, four copies in tandem of the salicylic acid-binding protein 2-like gene were found in *P. edulis*: an orthologous copy was found in chromosome 4 and three in chromosome 9 of *P. trichocarpa*, but only one copy was found in chromosome 17 of *M. esculenta* (Supplementary Fig. S1).

There is a higher degree of comparative microsynteny between *P. edulis* and *P. trichocarpa* than between *P. edulis* and *M. esculenta*. The number of genes is significantly high in most *P. trichocarpa* and *M. esculenta* chromosomes compared to that found in *P. edulis* microsyntenic regions (Tables 4 and 5). The highest level of synteny conservation was found between Pe173B16 and *P. trichocarpa* chromosome 9, with 29 orthologous, collinear gene pairs (Table 4; Fig. 7), and between Pe185D11 and *M. esculenta* chromosome 12, with 27 orthologous, collinear gene pairs (Table 5; Fig. 3).

## Discussion

Despite great advances in genome sequencing, the process of sequencing a plant genome is still laborious, due primarily to the size and complexity of genome regions which pose a challenge when it comes to sequencing and assembly. For instance, *Passiflora* species are extensively diversified in morphological terms, with genome sizes ranging from 207 Mb to 2.15 Gb^14^ and there are no draft genomes for any passion fruits, even the most cultivated species, *P. edulis*. In this study, a gene-rich fraction of the *P. edulis* genome was sequenced and assembled from long sequence reads, allowing us to obtain 10.4 Mb of highly curated data.

About half of all sequences (44%) matched *P. edulis* gene sequences and annotation revealed several functional categories and protein domains. Interestingly, the most frequent domain was retrotransposon gag, associated with transcripts of the LTR retrotransposon, followed by the kinase domains. This abundance was to be expected, since kinases belong to a superfamily of proteins with copies in the hundreds or thousands and are components of all cellular functions. These proteins use ATP γ-phosphate to phosphorylate serine and threonine or tyrosine residues from other proteins^57^. Note that to date there is an enormous scarcity of information on *Passiflora* nuclear genes in databases. This means that obtaining gene-based probes for selecting new regions for whole sequencing is practically impossible. The structural and functional annotation of 1,883 genes provides a significant set of high quality gene sequences that can be used in many other studies on *Passiflora* (see Supplementary Table S3).

Transposable elements (TEs) are highly widespread in plant genomes, accounting for 14% of the *Arabidopsis thaliana* genome^58^, up to 80% of the maize genome^59^ and 17.6% of all *P. edulis* sequences. The vast majority are retroelements that belong to Class I (96.4%), and especially to the LTR order. This abundance is very similar to that previously reported^15^ analyzing ~10,000 BES (18.5% TEs, 94.1% Class I TEs, the majority belonging to the LTR order), and this pattern should be repeated in *P. edulis*. On examining high quality genomes, several authors have stated that the spread of TEs (mostly retrotransposons) is the main driver of genome size variation in plants. This is particularly true of LTR retrotransposons due to the replication mechanism. LTRs are found mainly in centromeric regions, playing important role in chromatin structure maintenance, centromere performance and the regulation of host gene expression^60–62^.

The content of LTR elements in *P. edulis* is comparable to that identified in related Malpighiaceae species with completely sequenced genomes, although the abundance of TEs is highly variable. This variation is to be expected and is indicative of particular TE-driven evolutionary processes^60^. For instance, ~42% of the *P. trichocarpa* genome consists of transposable elements (although only 12.9% of the sequences could be classified as known TEs), the majority belonging to the LTR order (~60%). These figures relate to the draft genome of *P. trichocarpa*^24^, and the authors state that this genome could contain even more non-classified LTRs. In *R. communis*, approximately 50% of the genome consists of transposable elements, and LTRs were the most abundant, making up ~16% of the genome^63^, close to the value observed in *P. edulis* (13.6%), although the genome size of this species is ~3.8× larger than that of *R. communis*. Finally, in *Manihot esculenta*, ~25.7% of the genome consists of transposable elements, and LTR is also the most represented order among classified TEs, forming ~11% of the genomic sequences^25^. In this case, the genome report was based on 65% of an assembled genome of the domesticated variety.

In terms of microsatellite abundance, ~1.0% of all *P. edulis* sequences consisted of SSRs, with trinucleotide repeats prevalent (55.6%), even in CDS (93.8%). Microsatellite abundance generally varies from one genome region to another, but trinucleotides are usually overrepresented in coding sequences, due to selection pressures against mutations that may alter the reading frames^64^. The *P. edulis* results corroborate the findings of a pioneer study^65^ with regard to the effect that trinucleotide repeats are significantly more abundant in the expressed regions of plant genomes. Recently, a total of 1,300 perfect microsatellite sites were described in *P. edulis* genomic regions (with minimum 15× coverage as a cut off; Illumina paired-end reads) that were selected for marker development and *Passiflora* diversity analysis^66^. In this significant sample, the prevalence of tri-, tetra- and dinucleotides was found to be 41.0%, 36.4% and 22.6%, respectively.

In the *P. trichocarpa* genome, the predominance of mono- (69.8%), di- (19.5%) and trinucleotides (9.0%) decreased stepwise as the motif length increased (mono- to hexanucleotide repeats); 98% of *P. trichocarpa* mononucleotides consist of A/T motifs and only 2% of C/G motifs. The same applies to *P. edulis* (Fig. 2B). For di- and trinucleotides, the most frequent motifs were AT/AT (60.5%) and AAT/ATT (48.2%). In terms of coding sequences, 90.3% and 76.6% of the mono- and dinucleotides consist respectively of A/T and AG/CT motifs. Trinucleotides consist mainly of AAG/CTT, ACC/GGT and AGG/CCT motifs (~20% of each), and the frequencies of tetra-, penta- and hexanucleotides were very low^67^.

In *M. esculenta*, 37.4% of all SSRs corresponded to dinucleotides, and tri- and pentanucleotides were found in the same proportion (~24%); within the coding sequences, tri- and hexanucleotides accounted for 95.6%. AT/AT and AAT/ATT were the most common di- and trinucleotide motifs (~23% and ~12%, respectively) and, as in *P. edulis*, AG/CT and AAG/CTT were the most prevalent in coding sequences (~4% and ~23%, respectively)^68^. In the *R. communis* genome, most of the SSRs found were also dinucleotides (70.4%), followed by trinucleotides (24.9%). AT/TA was the most frequent motif among dinucleotides (75.3%) and AAT/TTA among trinucleotides (71%)^69^.

Clearly, the particular occurrence of certain motifs in plant genomes and in different genome regions is due to selection pressure during evolution^70,71^, and structural and functional genome attributes, like GC content and codon usage bias, may be responsible for the unique content and distribution patterns of microsatellites^72,73^.

Remarkable, there are several benefits that can be derived from the knowledge we have generated. First, a draft sequencing of the *Passiflora edulis* nuclear genome, especially of a gene-rich fraction, provides a platform for functional analysis and development of genomic tools in applied passion fruit improvement. Our work also represents a first step towards full sequencing of the *P. edulis* genome. Moreover, wild *Passiflora* species harbor a variety of characteristics that determine their ecological importance and adaptability. The availability of gene sequences could help researchers test for the presence of gene variants or polymorphisms in different environments. This is also possible for cultivated species. Gene prediction has yielded around 1,900 genes, and functional annotation has associated genes with plant immunity and defense functions (Supplementary Table S3).

Taxonomically speaking, the genus is subdivided into four subgenera: three clades were recognized as monophyletic (*Astrophea*, *Decaloba*, and *Passiflora*), but the position of *Deidamioides* remained unresolved, as this particular clade was found to be paraphyletic. Therefore, gene sequences could be used in phylogenetic analysis to obtain accurate evolutionary information.

By providing information on the levels of synteny conservation and rearrangements within the microcollinear regions (inverted and translocated segments, deletion and gene duplication events), this study will help confirm the relationships between a *Passiflora* species and related Malpighiales, with important taxonomical implications. Our previous phylogenetic analyses based on the available chloroplast genomes of members of the four families that compose the Malpighiales order indicated that the Passifloraceae are more closely related to the Salicaceae than to the Euphorbiaceae^16^. This proximity is definitively confirmed herein by microsynteny analysis, confirming the importance of using comparative genomic approaches as an additional resource for elucidating the phylogenetic relationships in the families that compose the Malpighiales order, one of the largest of flowering plants.

Although *P*. *edulis* microsyntenic regions were compared with whole genomes of *P. trichocarpa* (Salicaceae) and *M. esculenta* (Euphorbiaceae), i.e. species that belong to different taxonomic families, the analysis showed that overall gene order was well conserved. The level of microsynteny observed between the majority of *P. edulis* BAC inserts and these genomes is surprising, given the long divergence time that separates them from the common ancestor of the Malpighiales, some 100 million years ago^74^. The event of whole genome duplication (WGD) in *P. trichocarpa* occurred about 60−65 million years ago and reached around 92% of its genome^24^. On the other hand, *M. esculenta* has undergone a paleo-genome duplication event, and a number of its genes were found to have only two copies^25,75^. This may be related to the loss of one of the homologous copies in *M. esculenta* owing to selection pressure that restored the single-copy state of genes that impair fitness when present in multiple copies^76^.

The genome size of *P. edulis* is estimated at ~1.23 Gb, significantly higher than the estimated genome sizes of *P. trichocarpa* (~485 Mb)^24^ and *M. esculenta* (~742 Mb)^25^. These differences raise the question: did an ancestor of the passionflowers undergo genome duplication? Possibly. According to cytogenetic studies, the basic chromosome number in the genus *Passiflora* is *x* = 6, with several species containing secondary numbers, as in the case of *P. edulis* (*x* = *9*). These species with secondary chromosome numbers are possibly of polyploid origin^77,78^. Nevertheless, there is evolutionary evidence indicating *x* = *12* as the basic chromosome number, since *x* = 6 was reported to occur only in the subgenus *Decaloba*. In primitive *Passiflora* species, such as those of the *Astrophea* subgenus, *x* = *12*, and the same applied to other species of the Passifloraceae family^78,79^. This suggests that descending dysploidy events may have occurred in the *Passiflora* (*x* = *9*) and *Decaloba* (*x* = *6*) subgenera, lending weight to the hypothesis that genome duplication occurred in an ancestor of the Passifloraceae. In actual fact the diploid numbers *2n* = *12*, *18, 24, and 72* have been reported for *Passiflora* species^80^.

An examination of the microsyntenic regions shows that the *P. edulis* gene-rich segments are more compact than those of the species compared, even though its genome size is three times longer than that of *P. trichocarpa*, and almost twice the size of the *M. esculenta* genome. The limited sampling of *P. edulis* genome analyzed herein does not account for these apparently contradictory attributes regarding the compactness of gene regions and genome sizes. Further studies are required to elucidate the abundance of repetitive DNA (including TEs) associated with gene-poor regions and/or the occurrence of large heterochromatin blocks in *P. edulis*^81,82^.

Finally, wide variations in genome size occur within the genus *Passiflora*^14^ indicating that genome duplication, DNA sequence acquisition and loss throughout the evolution of the genus (favoring species disruption) have occurred since its diversification from the common ancestor about 38 million years ago^83^.

## Conclusion

The outcome of this research was a unique set of high quality sequence data on a gene-rich fraction of the *Passiflora edulis* genome, describing gene content and abundance of repetitive elements. The structural and functional annotations of 1,883 genes of *P. edulis* are detailed. It is proposed that there is a relatively high degree of conservation in gene regions of *P. edulis*, *Populus trichocarpa* and *Manihot esculenta*, according to our microsynteny analysis results. Collinear orthologous genes are shown to be prevalent, although some disruptions of collinearity have occurred due to rearrangements (inversion, translocation events) within microsyntenic regions. Interestingly, even though the *P. edulis* genome is much larger than those of *P. trichocarpa* (3×) and *M. esculenta* (2×), which evolved by polyploidy, the *P. edulis* gene-rich segments are much more compact. In this study the first steps have been taken, but further studies are required to elucidate the abundance of repetitive DNA associated with gene-poor regions and/or the occurrence of large heterochromatin blocks in *P. edulis*, in order to contribute to our understanding of the evolutionary issues that these genomes raise.

## Electronic supplementary material

Supplementary Figures S1-S13

Supplementary Tables S1 and S2

Supplementary Tables S3 and S4
