# Supplementary material for: A gene-rich fraction analysis of the Passiflora edulis genome reveals highly conserved microsyntenic regions with two related Malpighiales species
Source: Sci Rep. 2018 Aug 29;8:13024. doi: 10.1038/s41598-018-31330-8 (PMC6115403; doi:10.1038/s41598-018-31330-8)

Supplementary Information

A gene-rich fraction analysis of the *Passiflora edulis* genome reveals highly conserved regions with two related Malpighiales species

Carla Freitas Munhoz, Zirlane Portugal Costa, Luiz Augusto Cauz-Santos, Alina Carmen Egoávil Reátegui, Nathalie Rodde, Stephane Cauet, Marcelo Carnier Dornelas, Philipe Leroy, Alessandro Melo Varani, Hélène Berges and Maria Lucia Carneiro Vieira[*]

***Correspondence:**
Maria Lucia Carneiro Vieira
mlcvieir@usp.br

Supplementary Figure S1. Collinear microsyntenic regions identified *in Passiflora edulis* (yellow bars) and *Populus trichocarpa* chromosome 4 and 9 (green bars) and *Manihot esculenta* chromosome 15 and 17 (brown bars). The orthologous genes of *P. edulis* are duplicated in both species.

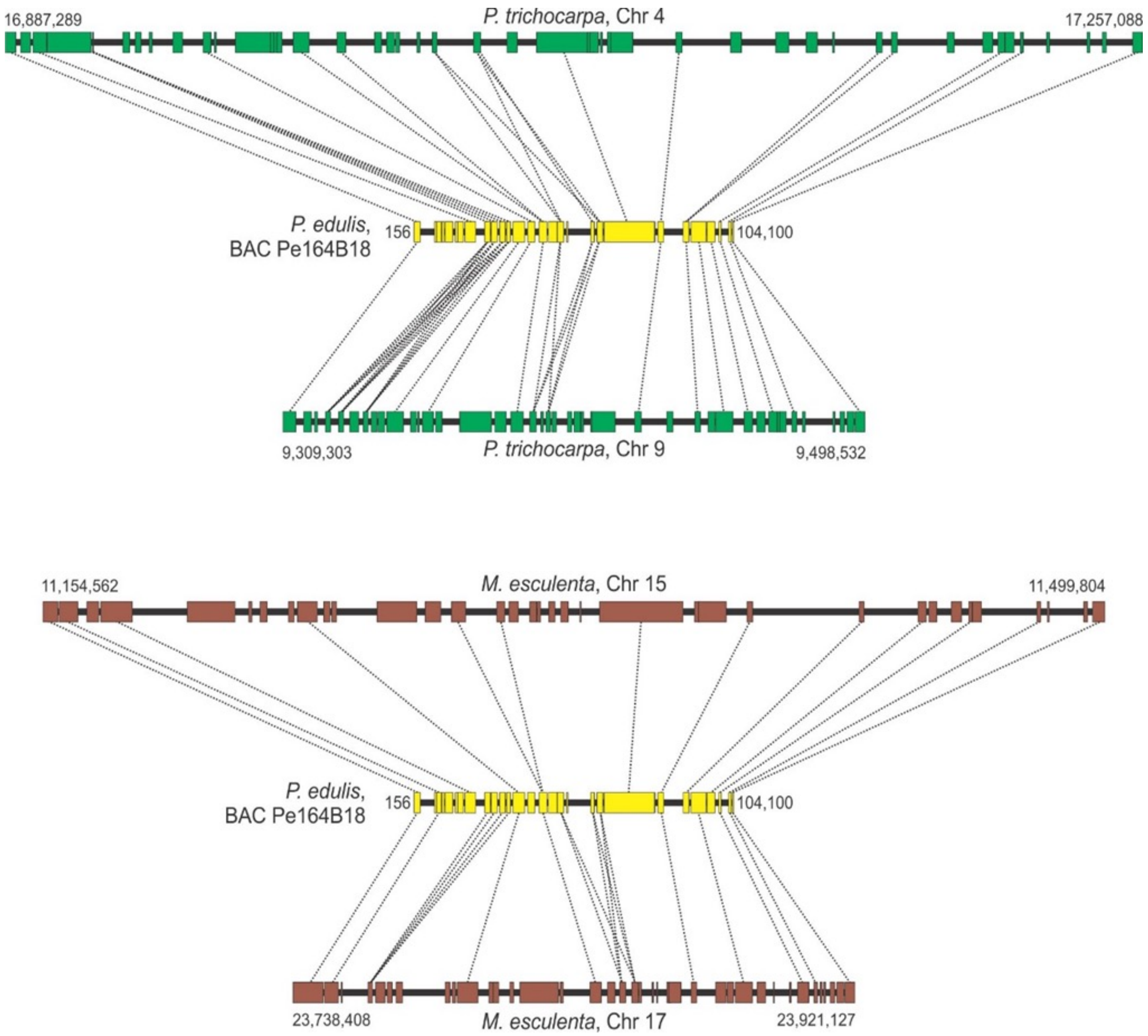

Supplementary Figure S2. Collinear microsyntenic regions identified in *Passiflora edulis* (yellow bars) and *Populus trichocarpa* chromosome 4 and 17 (green bars) and *Manihot esculenta* chromosome 1 and 2 (brown bars). Note the opposite orientation of *P. trichocarpa* chromosome 4. Rearranged segments are seen in both comparisons. The orthologous genes of *P. edulis* are duplicated in both species.

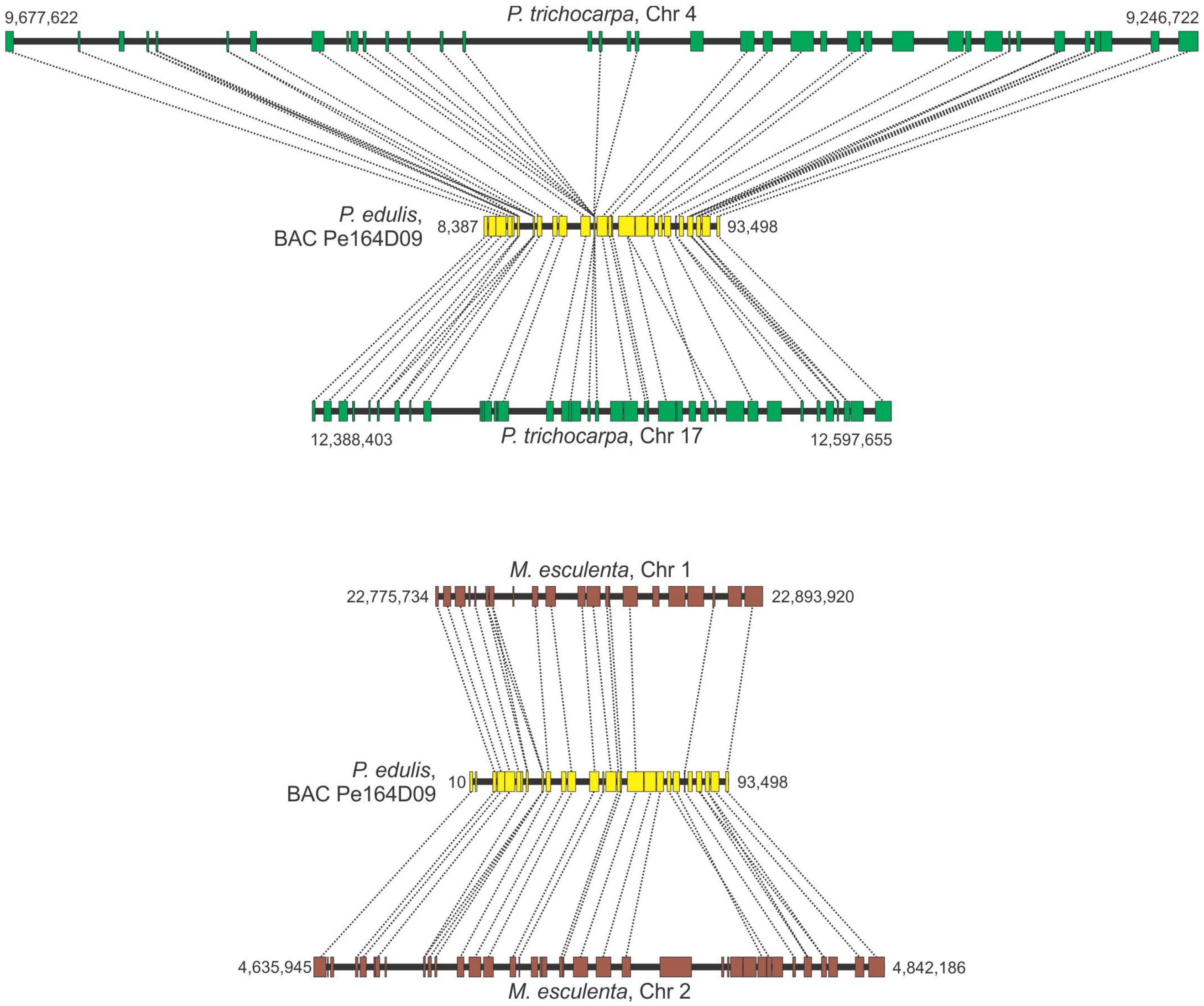

Supplementary Figure S3. Collinear microsyntenic regions identified in *Passiflora edulis* (yellow bars) and *Populus trichocarpa* chromosome 2 and 14 (green bars) and *Manihot esculenta* chromosome 1 and 5 (brown bars). Note the inverted segment on chromosome 2 of *P. trichocarpa*, and the opposite orientation of the *P. edulis* microsyntenic region relatively to *M. esculenta* chromosomes. The orthologous genes of *P. edulis* are duplicated in both species.

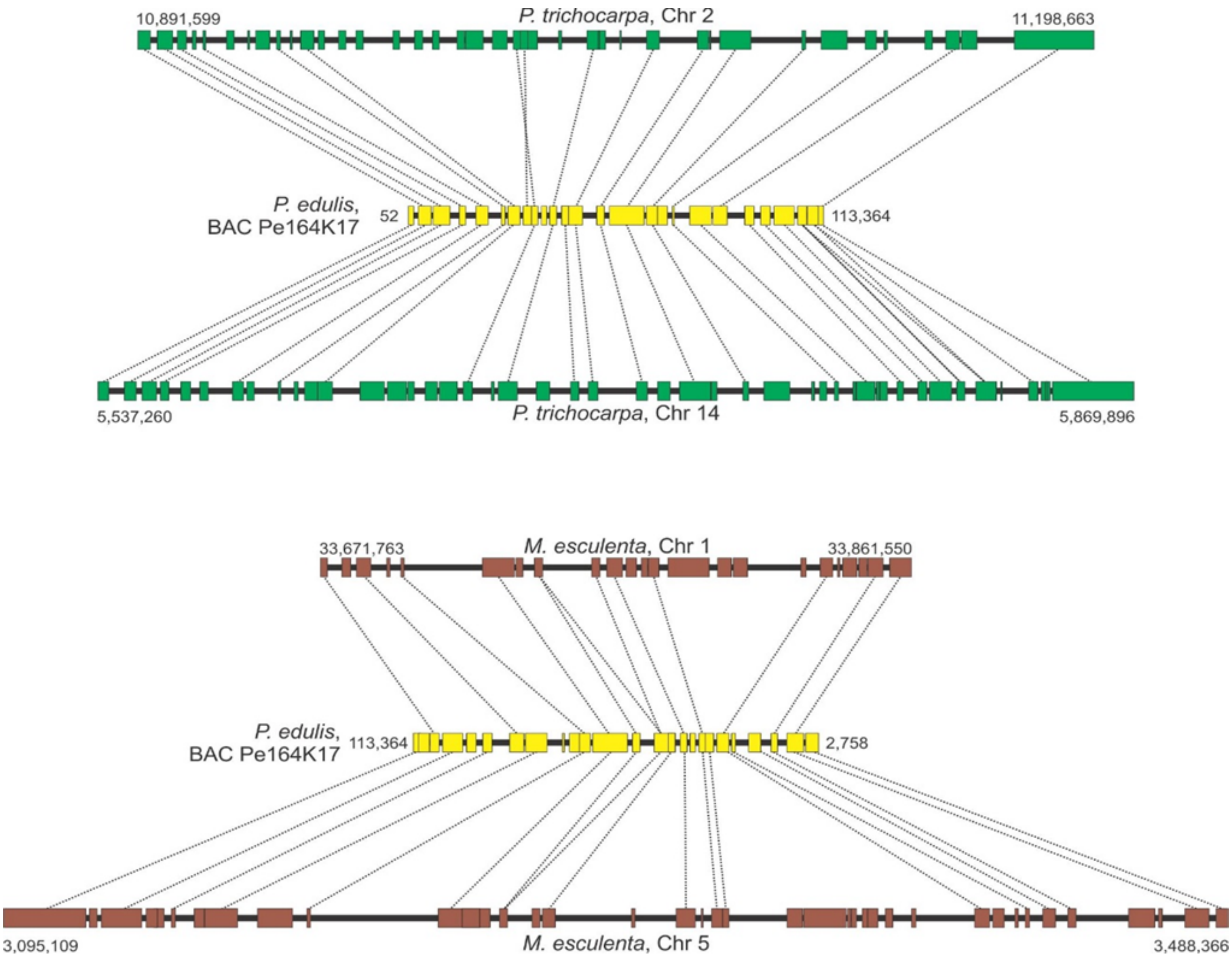

Supplementary Figure S4. Collinear microsyntenic regions identified in *Passiflora edulis* (yellow bars) and *Populus trichocarpa* chromosome 1 (green bar) and *Manihot esculenta* chromosome 15 and 17 (brown bars). Note the opposite orientation of *M. esculenta* chromosome 15. The orthologous genes of *P. edulis* are duplicated in *M. esculenta*.

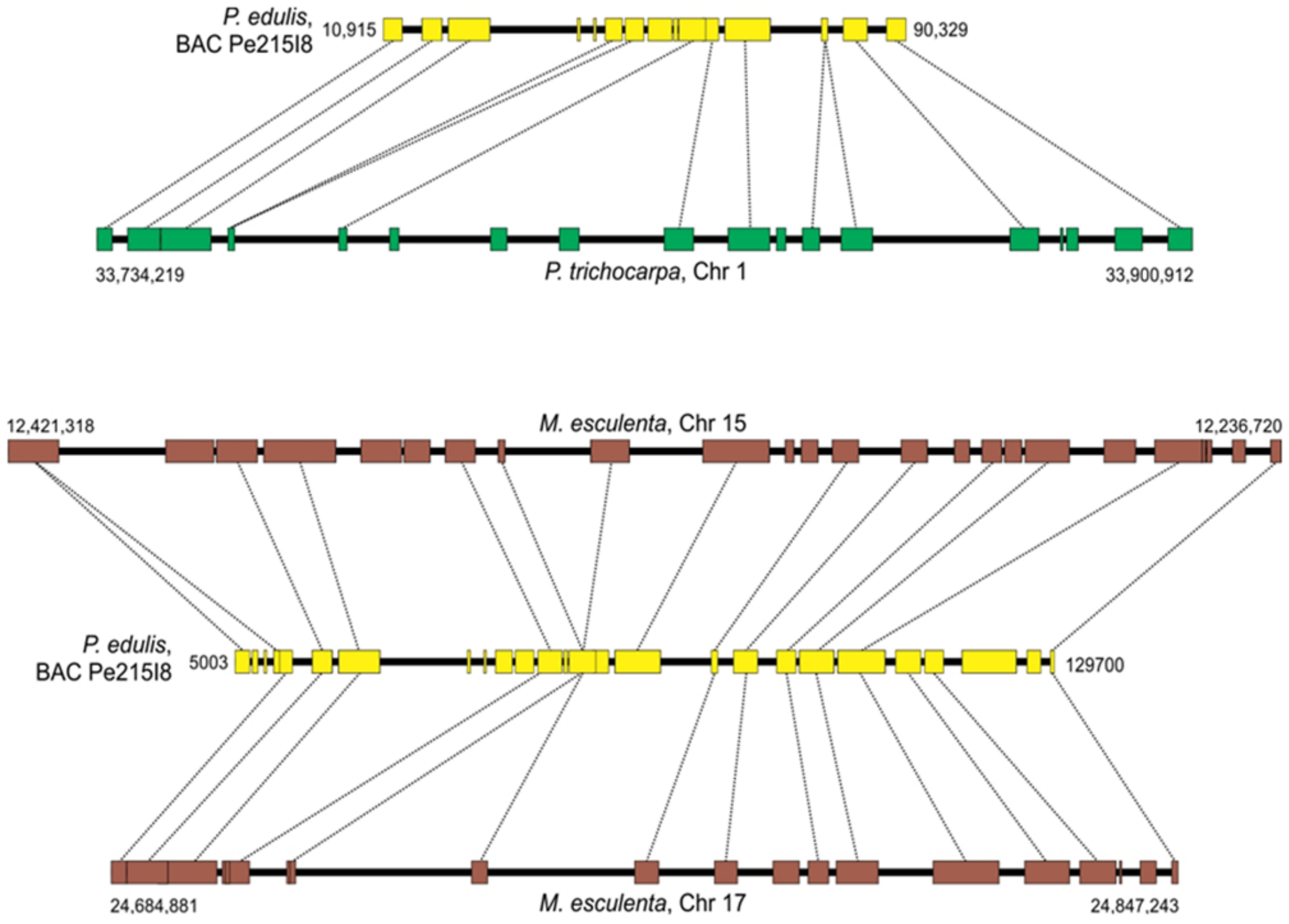

Supplementary Figure S5: Collinear microsyntenic regions identified in *Passiflora edulis* (yellow bars) and *Populus trichocarpa* chromosome 14 (green bar) and *Manihot esculenta* chromosome 1 and 5 (brown bars). Note the opposite orientation of *M. esculenta* chromosome 1. The orthologous genes of *P. edulis* are duplicated in *M. esculenta*.

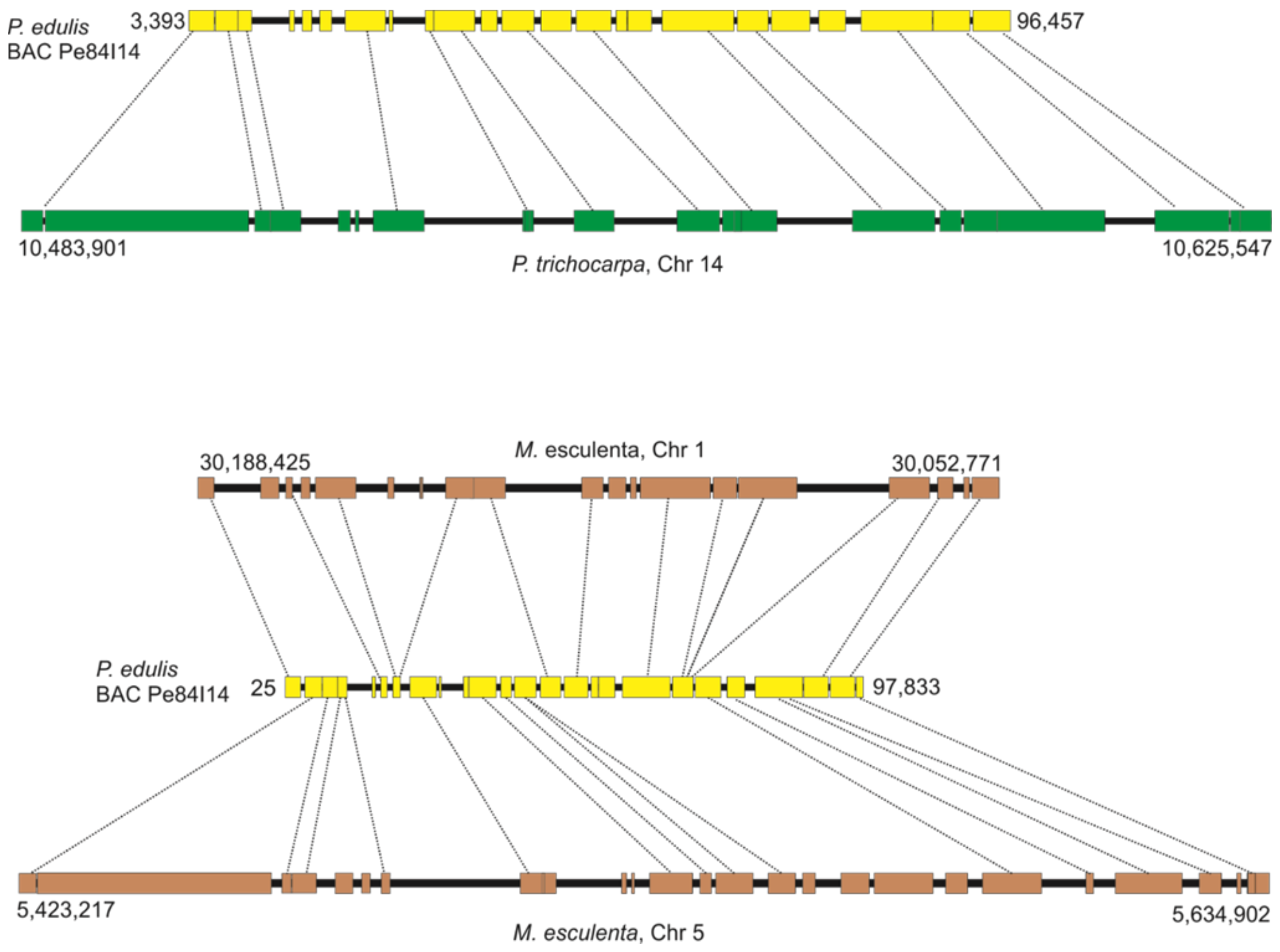

Supplementary Figure S6. Collinear microsyntenic regions identified in *Passiflora edulis* (yellow bars) and *Populus trichocarpa* chromosome 2 and 5 (green bars) and *Manihot esculenta* chromosome 2 and 18 (brown bars). Note the opposite orientation of *P. trichocarpa* chromosome 2. A typical inversion is seen in *P. edulis* syntenic region relatively *P. trichocarpa* chromosome 5. The orthologous genes of *P. edulis* are duplicated in both species.

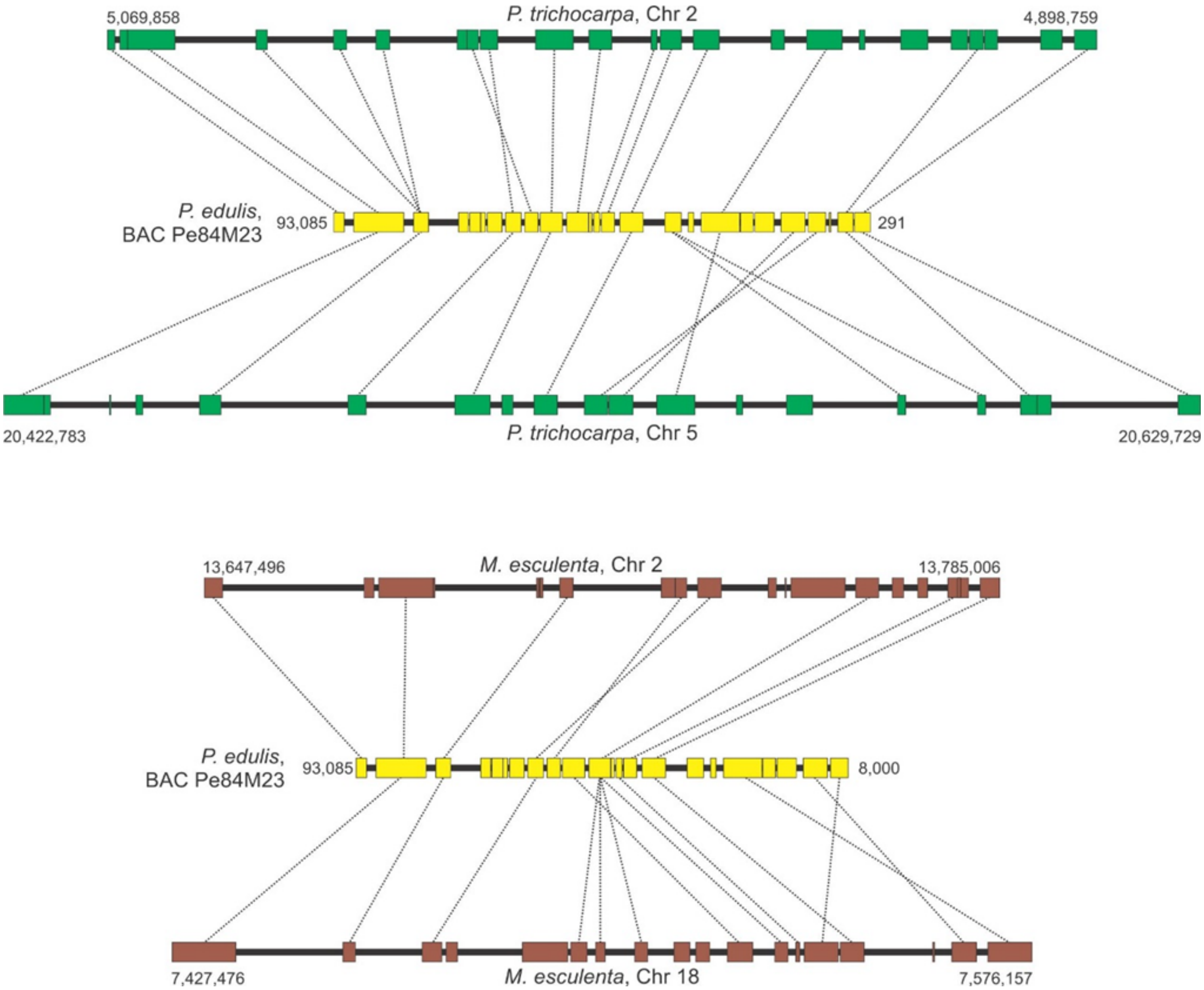

Supplementary Figure S7. Collinear microsyntenic regions identified in *Passiflora edulis* (yellow bars) and *Populus trichocarpa* chromosome 12 and 15 (green bars) and *Manihot esculenta* chromosome 6 and 14 (brown bars). Rearranged segments are seen in both comparisons. Note the opposite orientation of *M. esculenta* chromosome 14. The orthologous genes of *P. edulis* are duplicated in both species.

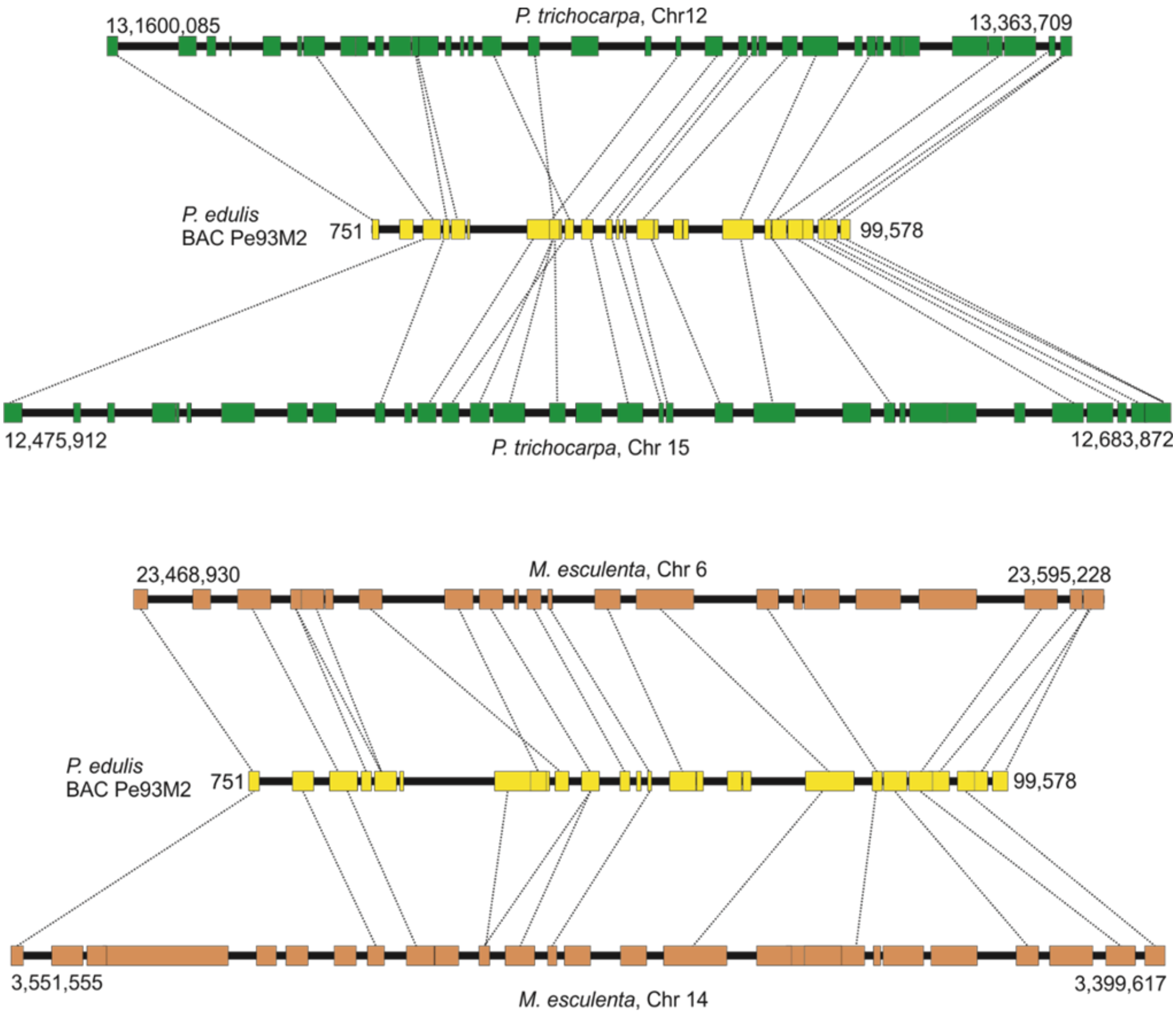

Supplementary Figure S8. Collinear microsyntenic regions identified in *Passiflora edulis* (yellow bar) and *Populus trichocarpa* chromosome 7 (green bars).

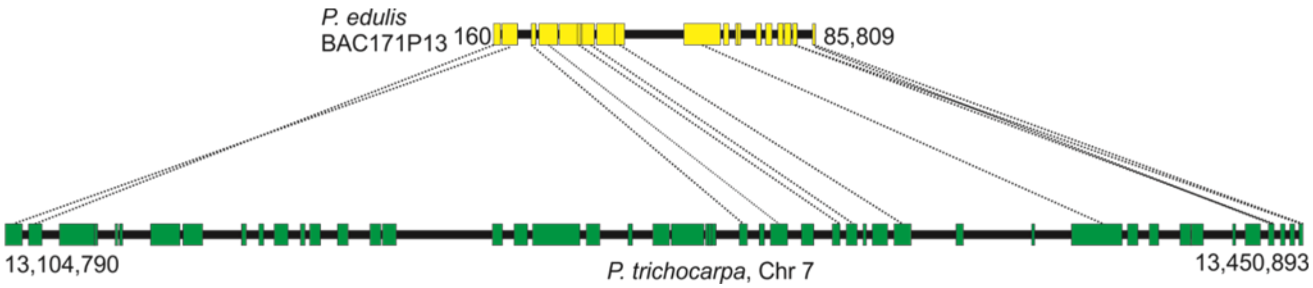

Supplementary Figure S9. Collinear microsyntenic regions identified in *Passiflora edulis* (yellow bars) and *Populus trichocarpa* chromosome 1 (green bar) and *Manihot esculenta* chromosome 15 (brown bar). Note the opposite orientation of *P. trichocarpa* chromosome 1.

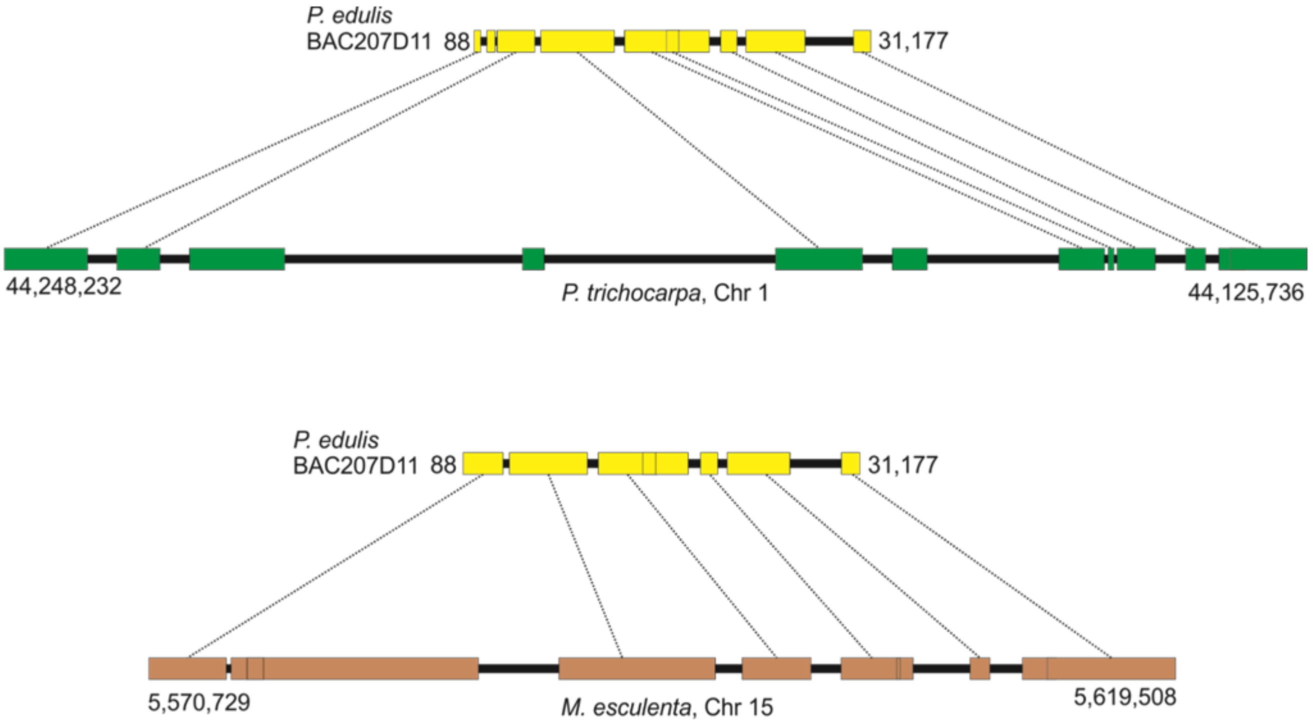

Supplementary Figure S10. Collinear microsyntenic regions identified in *Passiflora edulis* (yellow bar) and *Populus trichocarpa* chromosome 6 and 18 (green bars). Note the opposite orientation of *P. trichocarpa* chromosome 18. The orthologous genes of *P. edulis* are duplicated in *P. trichocarpa*.

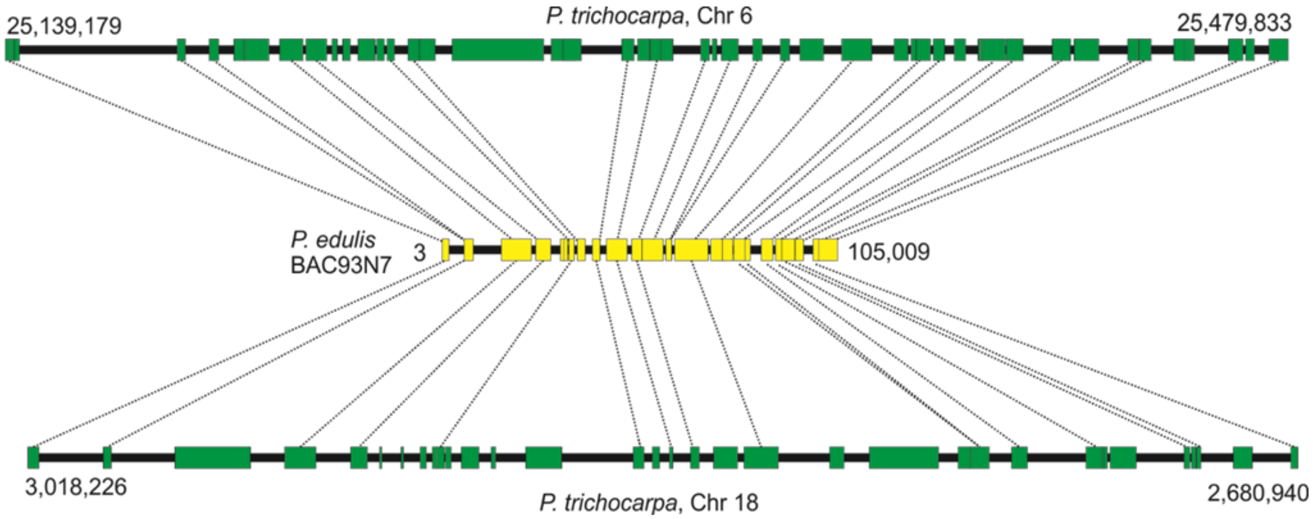

Supplementary Figure S11. Collinear microsyntenic regions identified in *Passiflora edulis* (yellow bars) and *Populus trichocarpa* chromosome 6 and 18 (green bars) and *Manihot esculenta* chromosome 3 and 16 (brown bars). Note the opposite orientation of *P. trichocarpa* chromosome 18 and *M. esculenta* chromosome 3. The orthologous genes of *P. edulis* are duplicated in both species.

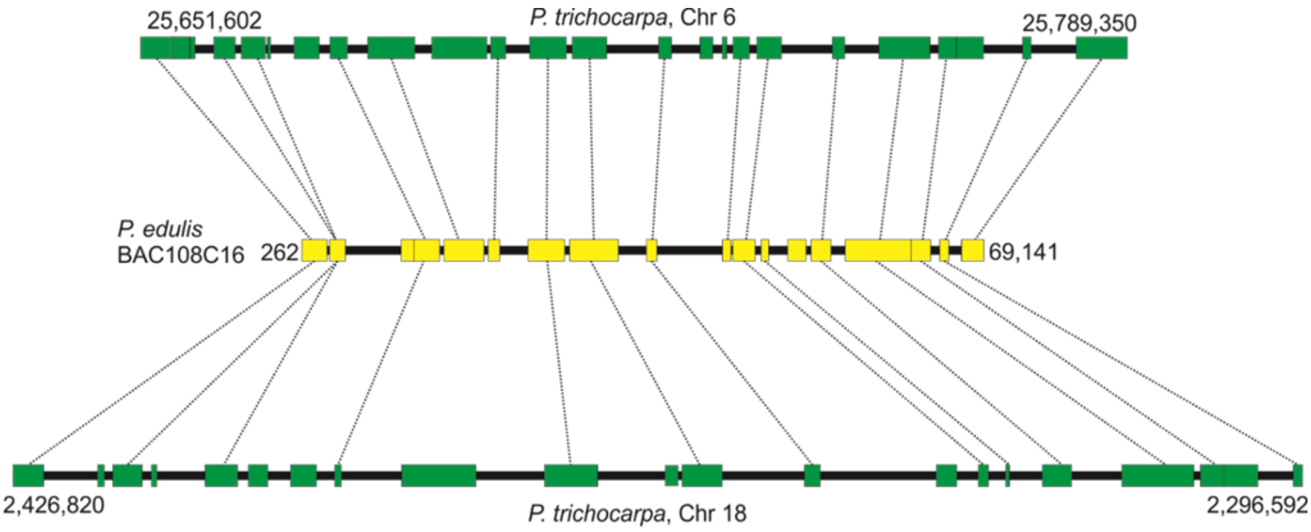

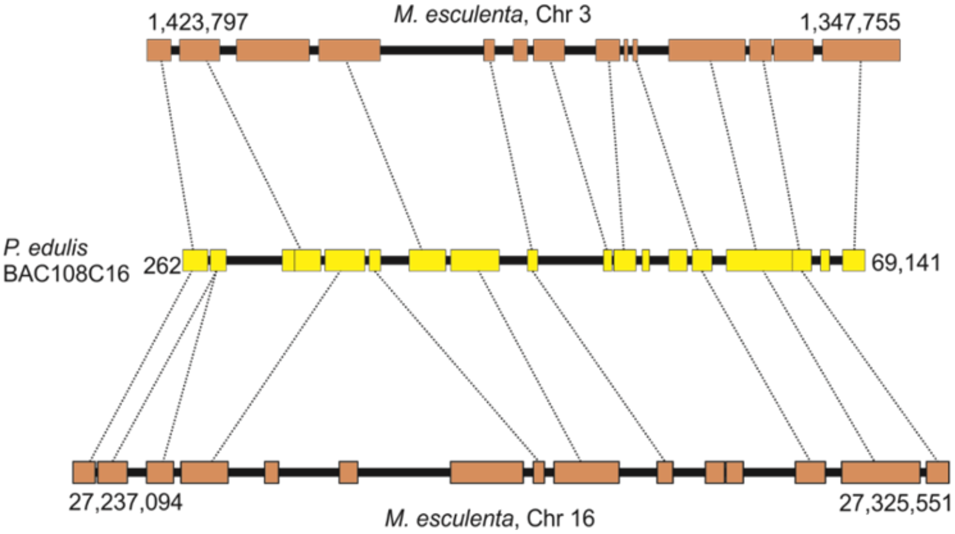

Supplementary Figure S12. Collinear microsyntenic regions identified in *Passiflora edulis* (yellow bars) and *Populus trichocarpa* chromosome 12 (green bar) and *Manihot esculenta* chromosome 1 (brown bar).

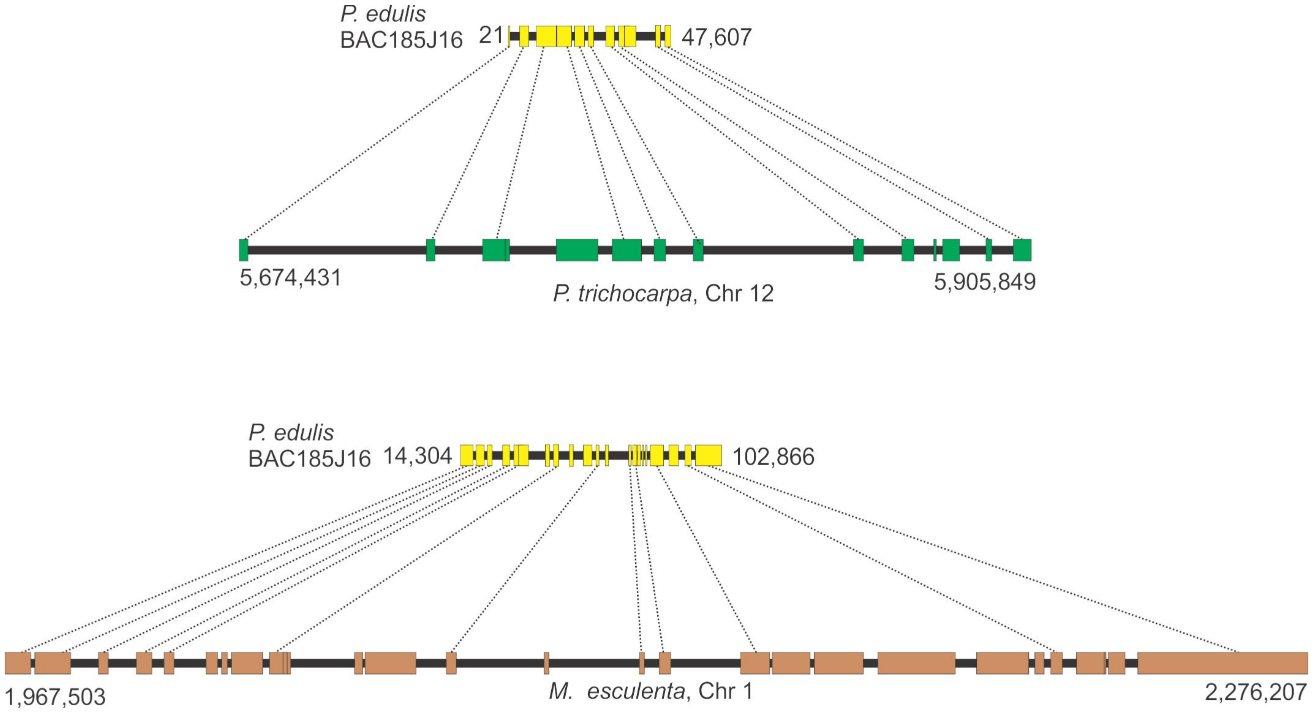

Supplementary Figure S13. Collinear microsyntenic regions identified in *Passiflora edulis* (yellow bars) and *Populus trichocarpa* chromosome 2 and 5 (green bars) and *Manihot esculenta* chromosome 18 (brown bar). Note the opposite orientation of *P. trichocarpa* chromosome 2. The orthologous genes of *P. edulis* are duplicated in *P. trichocarpa*.

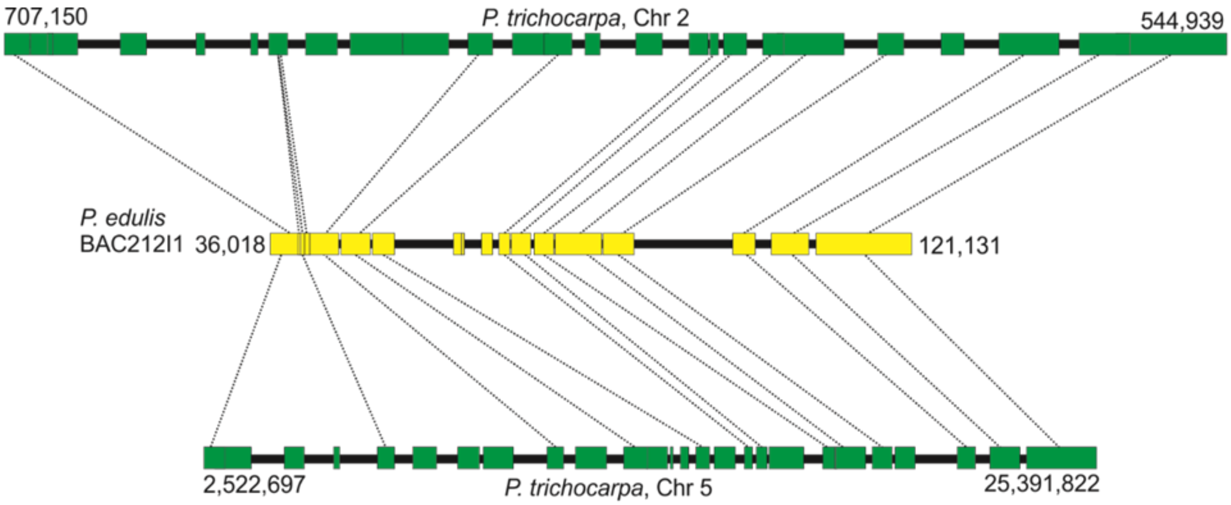

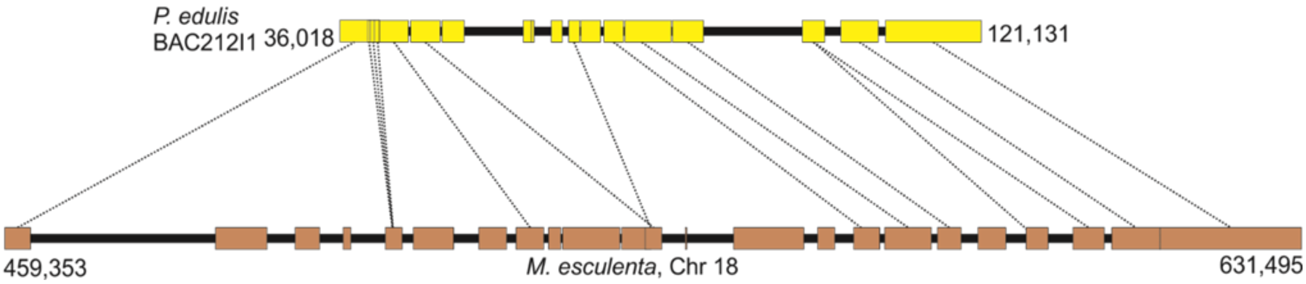

Supplement: Supplementary file 1 — Supplementary Figures S1-S13 [file 41598_2018_31330_MOESM1_ESM.pdf]
