# Supplementary material for: A gene-rich fraction analysis of the Passiflora edulis genome reveals highly conserved microsyntenic regions with two related Malpighiales species
Source: Sci Rep. 2018 Aug 29;8:13024. doi: 10.1038/s41598-018-31330-8 (PMC6115403; doi:10.1038/s41598-018-31330-8)
Supplement: Supplementary file 2 — Supplementary Tables S1 and S2 [file 41598_2018_31330_MOESM2_ESM.pdf]

Supplementary Information

A gene-rich fraction analysis of the *Passiflora edulis* genome reveals highly conserved regions with two related Malpighiales species

Carla Freitas Munhoz, Zirlane Portugal Costa, Luiz Augusto Cauz-Santos, Alina Carmen Egoávil Reátegui, Nathalie Rodde, Stephane Cauet, Marcelo Carnier Dornelas, Philipe Leroy, Alessandro Melo Varani, Hélène Berges and Maria Lucia Carneiro Vieira*

***Correspondence:**
Maria Lucia Carneiro Vieira
mlcvieir@usp,br

Supplementary Table S1. BAC inserts selected from the *Passilfora edulis* genomic library for complete sequencing.

BAC code	BAC selection criterium
Pe1K19	Presence of gene in the forward BAC-end sequence (BES)
Pe1M17	Potencial non co-localized microsyntenic region with *Populus trichocarpa*
Pe3F10	Probe complementary to the gene celulose sintase
Pe7A23	Probe complementary to mitochondrial gene
Pe7M15	Probe complementary to the gene stromal cell-derived factor 2-like protein precursor
Pe9E4	Probe complementary to the gene auxin response factor 2
Pe15E1	Probe complementary to the gene (+)-neomenthol dehydrogenase
Pe20E10	Probe complementary to the gene cytochrome C oxidase subunit 2
Pe20N3	Probe complementary to the gene lipoxygenase 2
Pe21O15	Probe complementary to the gene glutamine synthetase
Pe24G19	Probe complementary to the gene sugar transport protein 13
Pe27H17	Probe complementary to the gene kinesin-like protein
Pe28D11	Probe complementary to the gene heat stress transcription factor C-1
Pe28E22	Probe complementary to the gene glutamate receptor
Pe28I20	Probe complementary to the gene glycolate oxidase
Pe33M2	Presence of genes in both BES
Pe34H9	Probe complementary to the gene opper/zinc-superoxide dismutase 1a
Pe34M7	Presence of genes in both BES
Pe43D2	Presence of genes in both BES
Pe43L2	Presence of genes in both BES
Pe51C2	Probe complementary to the gene F-box/LRR-repeat protein 4
Pe60G10	Probe complementary to the gene serine/threonine-protein kinase-like protein ACR4
Pe61E2	Probe complementary to the gene mitochondrial outer membrane protein porin of 36 kDa
Pe63J18	Probe complementary to the gene chlorophyll a b binding
Pe64C12	Probe complementary to the gene basic endochitinase B
Pe65F7	Probe complementary to the gene celulose sintase
Pe69C7	Potencial non co-localized microsyntenic region with *Populus trichocarpa*
Pe69F22	Presence of genes in both BES
Pe69G18	Presence of genes in both BES
Pe69H24	Presence of genes in both BES
Pe69N18	Potencial collinear microsyntenic region with *Arabidopsis thaliana*
Pe69O16	Presence of genes in both BES
Pe71E3	Probe complementary to the gene stearoyl-acp desaturase
Pe74I6	Probe complementary to the gene beta-amylase 1
Pe75A21	Presence of genes in both BES
Pe75D12	Potencial collinear microsyntenic region with *Populus trichocarpa*
Pe75F13	Presence of gene in the reverse BES
Pe75F20	Presence of gene in the forward BES
Pe75K15	Presence of genes in both BES
Pe75N15	Presence of genes in both BES
Pe84I14	Presence of genes in both BES
Pe84K8	Presence of genes in both BES
Pe84M18	Presence of gene in the forward BES
Pe84M23	Presence of genes in both BES
Pe84M6	Presence of gene in the reverse BES
Pe85B19	Presence of genes in both BES
Pe85H4	Presence of genes in both BES

Pe85I9	Presence of genes in both BES
Pe85J23	Presence of genes in both BES
Pe85L8	Potencial collinear microsyntenic region with *Arabidopsis thaliana*
Pe85O9	Presence of genes in both BES
Pe86F9	Probe complementary to the gene ATP-citrate synthase beta chain protein 2
Pe86H07	Probe complementary to the gene cytochrome C oxidase
Pe89E10	Probe complementary to the gene homeobox-leucine zipper athb-6
Pe93A7	Presence of gene in the forward BES
Pe93F5	Presence of gene in the reverse BES
Pe93J9	Presence of genes in both BES
Pe93K19	Presence of genes in both BES
Pe93M2	Presence of genes in both BES
Pe93M4	Presence of gene in the reverse BES
Pe93N7	Presence of gene in the forward BES
Pe93O18	Presence of genes in both BES
Pe99P16	Probe complementary to the gene glutamate-cysteine ligase
Pe101F21	Presence of genes in both BES
Pe101H15	Presence of genes in both BES
Pe101K14	Presence of genes in both BES
Pe101O4	Presence of genes in both BES
Pe101P13	Presence of genes in both BES
Pe101P7	Presence of gene in the reverse BES
Pe103M2	Probe complementary to the gene alpha-L-arabinofuranosidase 1
Pe108C16	Probe complementary to the gene glutamate-cysteine ligase
Pe113A7	Probe complementary to the gene harpin-induced protein/protein YLS9-like
Pe117C17	Probe complementary to the gene inactive beta-amylase 9
Pe123N8	Probe complementary to the gene phosphoribulokinase
Pe125I23	Probe complementary to the gene cyclin-dependent protein kinase regulator
Pe134H15	Probe complementary to the gene 1-aminocyclopropane-1-carboxylate oxidase
Pe135J12	Probe complementary to the gene ubiquitin-conjugating enzyme E2-23 kDa-like
Pe138G17	Probe complementary to the gene putative serine proteinase
Pe141B12	Presence of genes in both BES
Pe141H13	Presence of genes in both BES
Pe141J23	Presence of genes in both BES
Pe141K8	Potencial non co-localized microsyntenic region with *Populus trichocarpa*
Pe164A12	Potencial collinear microsyntenic region with *Vitis vinifera*
Pe164B18	Presence of genes in both BES
Pe164D9	Presence of genes in both BES
Pe164K17	Potencial collinear microsyntenic region with *Populus trichocarpa*
Pe168B17	Probe complementary to the gene nucleoredoxin 1
Pe171P13	Presence of genes in both BES
Pe173B16	Potencial rearranged microsyntenic region with *Populus trichocarpa*
Pe175N8	Probe complementary to the gene disease resistance family protein
Pe185D11	Presence of genes in both BES
Pe185J16	Presence of genes in both BES
Pe186E19	Probe complementary to the gene brassinosteroid insensitive 1
Pe195F4	Probe complementary to the gene glycerate dehydrogenase
Pe198H23	Probe complementary to the gene ethylene response sensor
Pe201C11	Probe complementary to the gene maltose excess protein 1
Pe207D11	Probe complementary to the gene kinesin-like protein
Pe209G15	Probe complementary to the gene lipoxygenase 2

Pe212D7	Probe complementary to the gene oxygen-evolving enhancer protein 1-1
Pe212I1	Presence of genes in both BES
Pe212J12	Presence of gene in the reverse BES
Pe212M5	Presence of gene in the reverse BES
Pe213C9	Probe complementary to the gene F-box protein PP2-A12 isoform X2
Pe214A18	Potencial non co-localized microsyntenic region with *Populus trichocarpa/Vitis vinifera*
Pe214H11	Potencial non co-localized microsyntenic region with *Populus trichocarpa*
Pe214N19	Presence of genes in both BES
Pe215I8	Probe complementary to the gene glyceraldehyde-3-phosphate dehydrogenase
Pe216B2	Presence of genes in both BES
Pe216B22	Potencial collinear microsyntenic region with *Populus trichocarpa*
Pe216F3	Presence of genes in both BES
Pe216F9	Presence of genes in both BES
Pe216I5	Potencial non co-localized microsyntenic region with *Populus trichocarpa*

Supplementary Table S2. Sequencing results of the 112 BAC inserts selected from the *Passilfora edulis* genomic library.

BAC code	Gel-estimated insert size (bp)	Reads number	Size range of reads (bp)	% GC	Mean coverage (×)	Mean QV*	Contig size (pb)
Pe1K19	52,000	809	599-35,075	42	122	48.51	48,154
Pe1M17	50,000	628	500-23,379	43	40	48.49	54,675
Pe3F10	48,000	572	518-23,463	40	40	48.50	50,560
Pe7A23	30,000	206	509-16,142	43	86	48.40	22,590
Pe7M15	122,000	5,667	500-31,277	42	186	48.55	117,775
Pe9E4	90,000	1,921	501-42,009	42	87	48.54	88,822
Pe15E1	90,000	4,981	500-39,121	42	212	48.55	88,169
Pe20E10	105,000	10,632	500-42,297	41	437	48.52	107,924
Pe20N3	100,000	5,322	503-39,062	40	410	48.55	96,493
Pe21O15	80,000	3,202	510-36,046	40	238	48.52	86,369
Pe24G19	80,000	2,652	501-23,230	44	118	48.57	83,926
Pe27H17	95,000	2,280	504-36,741	43	222	48.77	95,184
Pe28D11	125,000	9,650	500-42,658	45	350	48.54	122,897
Pe28E22	97,000	4,226	501-22,839	41	137	48.58	97,719
Pe28I20	85,000	3,212	502-38,789	41	216	48.51	91,911
Pe33M2	100,000	1,621	500-38,247	41	154	48.81	97,233
Pe34H9	80,000	4,859	502-35,035	40	191	48.56	92,133
Pe34M7	130,000	5,824	500-29,279	42	136	48.57	130,776
Pe43D2	80,000	3,912	504-42,904	40	322	48.55	88,638
Pe43L2	75,000	5,002	520-38,476	39	398	48.56	93,150
Pe51C2	100,000	11,496	501-46,831	40	554	48.56	93,290
Pe60G10	100,000	6,392	500-37,191	42	307	48.55	96,564
Pe61E2	100,000	14,523	500-45,838	38	670	48.56	95,754
Pe63J18	90,000	3,575	501-22,529	43	119	48.56	91,586
Pe64C12	90,000	2,738	500-23,673	42	101	48.58	97,338
Pe65F7	90,000	3,340	500-21,851	41	121	48.58	89,394
Pe69C7	97,000	3,068	500-25,108	40	104	48.59	103,741
Pe69F22	90,000	4,436	501-40,778	39	380	48.56	87,349
Pe69G18	95,000	4,517	500-35,983	38	371	48.57	91,665
Pe69H24	110,000	5,497	517-38,176	39	398	48.57	104,185
Pe69N18	95,000	2,340	500-22,458	37	87	48.48	94,052
Pe69O16	90,000	4,555	500-41,504	40	394	48.55	90,157
Pe71E3	90,000	4,437	500-35,709	41	382	48.55	85,814
Pe74I6	120,000	14,131	501-39,105	39	582	48.56	112,038
Pe75A21	110,000	5,760	501-37,523	42	386	48.56	109,110
Pe75D12	90,000	9,698	500-25,329	42	344	48.54	92,130
Pe75F13	115,000	3,251	500-42,063	41	221	48.53	112,223
Pe75F20	108,000	4,175	500-34,745	39	319	48.52	101,327
Pe75K15	105,000	4,519	503-37,147	44	327	48.55	100,781
Pe75N15	90,000	4,020	503-42,177	43	322	48.54	94,339
Pe84I14	100,000	5,268	504-39,183	40	327	48.55	97,848
Pe84K8	85,000	4,069	503-34,441	39	289	48.55	85,616
Pe84M6	96,000	2,859	501-44,938	42	240	48.51	92,167
Pe84M18	111,000	5,102	509-39,425	40	383	48.55	103,941
Pe84M23	100,000	5,081	502-43,845	38	397	48.56	93,217

Pe85L8	90,000	4,972	500-26,035	38	173	48,57	91,155
Pe85B19	100,000	4,916	508-35,487	40	306	48.54	96,953
Pe85H4	90,000	4,388	500-30,909	39	297	48.55	88,298
Pe85I9	95,000	4,870	500-39,620	41	301	48.55	95,720
Pe85J23	85,000	3,943	501-36,770	39	278	48.54	85,126
Pe85O9	30,000	391	573-33,837	39	78	48.52	25,363
Pe86F9	110,000	6,693	502-34,245	46	234	48.55	103,497
Pe86H7	95,000	4,691	503-37,269	43	284	48.54	96,593
Pe89E10	100,000	12,007	500-41,450	42	529	48.55	99,734
Pe93A7	104,000	3,503	509-38,522	41	272	48.53	99,578
Pe93F5	108,000	3,694	509-38,440	40	278	48.53	102,639
Pe93J9	110,000	6,340	501-35,344	40	362	48.55	107,024
Pe93K19	100,000	5,460	504-38,289	40	335	48.55	99,992
Pe93M2	100,000	5,170	500-38,116	41	311	48.55	100,436
Pe93M4	92,000	1,909	500-37,458	42	170	48.53	87,142
Pe93N7	119,000	5,922	501-39,176	40	423	48.54	106,968
Pe93O18	105,000	1,836	500-40,738	40	173	48.79	98,251
Pe99P16	100,000	12,248	500-42,116	41	545	48.55	99,641
Pe101F21	95,000	6,810	505-36,921	44	456	48.52	96,919
Pe101H15	80,000	6,099	509-35,269	42	335	47.51	88,834
Pe101K14+141H13	80,000/85,000	12,454	501-37,335	39	502	48.52	172,337
Pe101O4	110,000	6,498	503-39,352	38	376	48.53	117,581
Pe101P7	98,000	2,642	520-36,389	38	232	48.52	90,691
Pe101P13	85,000	8,236	501-37,124	37	620	48.52	91,580
Pe103M2	75,000	2,859	501-34,151	40	132	48.53	73,357
Pe108C16	105,000	2,800	500-40,287	41	277	48.79	96,753
Pe113A7	110,000	9,784	500-37,691	41	424	48.55	106,440
Pe117C17	95,000	6,484	501-39,316	42	232	48.53	103,905
Pe123N8	100,000	7,516	500-42,993	41	350	48.55	96,994
Pe125I23	95,000	4,004	500-39,630	39	243	48.55	96,568
Pe134H15	90,000	4,993	500-26,558	40	177	48.55	91,362
Pe135J12	105,000	6,446	503-40,401	40	238	48.55	103,564
Pe138G17	90,000	5,931	503-40,512	43	283	48.54	93,983
Pe141B12	75,000	4,423	502-33,544	43	360	48.53	79,426
Pe141J23	90,000	8,374	508-42,274	41	590	48.51	95,795
Pe141K8	90,000	2,619	500-23,918	40	99	48.58	97,973
Pe164A12	85,000	4,493	501-26,430	41	174	48.57	82,998
Pe164B18	100,000	9,006	501-37,510	40	574	48.52	104,102
Pe164D9	85,000	8,089	500-41,095	40	581	48.53	93,527
Pe164K17	112,000	3,334	502-25,218	40	106	48.58	113,504
Pe168B17	135,000	7,467	500-30,781	41	209	48.55	137,256
Pe171P13	110,000	2,710	500-43,329	40	234	48.79	111,123
Pe173B16	110,000	3,638	501-33,974	40	122	48.59	109,801
Pe175N8	115,000	9,084	500-38,499	41	402	48.55	106,381
Pe185D11	120,000	3,404	500-41,999	40	283	48.79	119,061
Pe185J16	105,000	2,343	506-40,202	39	221	48.78	103,095
Pe186E19	110,000	6,647	500-39,704	39	222	48.55	115,218
Pe195F4	110,000	6,995	500-38,598	40	235	48.55	113,443
Pe198H23	112,000	8,595	500-24,834	42	231	48.56	108,433
Pe201C11	140,000	8,471	500-39,592	40	226	48.56	140,216
Pe207D11	120,000	11,469	500-40,071	41	470	48.56	111,690

Pe209G15	90,000	6,971	500-40,057	39	336	48.56	94,376
Pe212D7	125,000	8,350	500-36,802	39	255	48.55	123,561
Pe212I1	120,000	3,365	502-41,492	40	265	48.79	121,384
Pe212J12	28,000	98	739-30,328	41	26	47.35	24,316
Pe212M5	48,000	549	500-36,767	41	91	48.50	43,763
Pe213C9	110,000	8,311	502-40,348	42	350	48.52	106,552
Pe214A18	97,000	3,152	500-24,957	43	110	48.55	106,977
Pe214H11	140,000	5,952	500-24,825	40	157	48.59	142,456
Pe214N19	100,000	1,871	509-38,049	44	173	48.78	98,343
Pe215I8	130,000	6,262	500-22,437	40	150	48.58	129,737
Pe216B2	50,000	153	504-37,581	36	32	48.57	42,359
Pe216B22	114,000	7,389	500-23,820	40	218	48.57	111,836
Pe216F3	80,000	907	504-41,986	40	111	48.79	79,451
Pe216F9	110,000	2,974	504-43,753	36	276	48.82	105,476
Pe216I5	68,000	1,526	500-18,384	40	77	48.58	72,701

- QV: Quality Value (Probability of incorrect base call: QV40= 1 in 10,000, QV50= 1 in 100,000).
