# Supplementary material for: A gene-rich fraction analysis of the Passiflora edulis genome reveals highly conserved microsyntenic regions with two related Malpighiales species
Source: Sci Rep. 2018 Aug 29;8:13024. doi: 10.1038/s41598-018-31330-8 (PMC6115403; doi:10.1038/s41598-018-31330-8)
Supplement: Supplementary file 3 — Supplementary Tables S3 and S4 [file 41598_2018_31330_MOESM3_ESM.pdf]

Supplementary Table S3. Annotation of 1,833 genes predicted in the *Passilfora edulis* sequences.

Gene code	Note	Frame	No. Exons	Gene size (bp)	CDS size (bp)	Sequence (BLASP results)	Sequence length (bp)	Hit	Hit ACC	E-Value	Alignment length	Positives
Pe1K19.1-2	Incomplete 5'	+	3	4737	840	C2 and GRAM domain-containing At5g50170 isoform X2 [Ricinus communis]	105	gi|359491211|ref|XP_002277671.2|PREDICTED: C2 and GRAM domain-containing protein At5g50170 [Vitis vinifera]	XP_002277671	2.85E-22	61	55
Pe1K19.3		+	9	3639	897	MAK16 homolog A-like isoform X1 [Vitis vinifera]	298	gi|743905651|ref|XP_011046229.1|PREDICTED: protein MAK16 homolog [Populus euphratica]	XP_011046229	3.00E-145	299	257
Pe1K19.4	Incomplete 3'	+	2	958	870	Plant intracellular Ras-group-related LRR 9-like	290	gi|255540519|ref|XP_002511324.1|PREDICTED: plant intracellular Ras-group-related LRR protein 9 [Ricinus communis]gi|223550439|gb|EEF51926.1|leucine-rich repeat-containing protein, putative [Ricinus communis]	XP_002511324, EEF51926	7.48E-101	305	238
Pe1M17.1		+	2	527	429	---Na---	142	No Blast Hit				
Pe1M17.2		+	4	2473	1095	Gag protease poly [Theobroma cacao]	364	gi|923615155|ref|XP_013745554.1|PREDICTED: uncharacterized protein LOC106448175 [Brassica napus]	XP_013745554	1.90E-07	99	57
Pe1M17.3		+	1	312	312	---Na---	103	No Blast Hit				
Pe1M17.4		-	2	447	357	Retrotransposon [Theobroma cacao]	118	gi|657948755|ref|XP_008339280.1|PREDICTED: uncharacterized protein LOC103402318 [Malus domestica]	XP_008339280	1.24E-41	148	96
Pe1M17.5		-	2	1539	1404	Gag protease poly [Theobroma cacao]	467	gi|590728434|ref|XP_007099662.1|Gag protease polyprotein-like protein [Theobroma cacao]gi|508728474|gb|EOY20371.1|Gag protease polyprotein-like protein [Theobroma cacao]	XP_007099662, EOY20371	2.41E-56	410	193
Pe1M17.6		-	2	1296	1110	Gag protease poly [Theobroma cacao]	369	gi|747069370|ref|XP_011081477.1|PREDICTED: uncharacterized protein LOC105164524 [Sesamum indicum]	XP_011081477	1.40E-09	116	67
Pe3F10.1	Incomplete 3'	-	1	1144	1144	Pentatricopeptide repeat-containing mitochondrial-like isoform X1 [Vitis vinifera]	381	gi|1000974665|ref|XP_015572568.1|PREDICTED: pentatricopeptide repeat-containing protein At3g54980. mitochondrial isoform X2 [Ricinus communis]	XP_015572568	6.17E-127	392	263
Pe3F10.2		-	10	3346	1504	tRNA (cytosine(38)-C(5))-methyltransferase-like	373	gi|802627241|ref|XP_012076609.1|PREDICTED: tRNA (cytosine-5-)-methyltransferase isoform X1 [Jatropha curcas]	XP_012076609	0.0	385	296
Pe3F10.3		+	1	1141	930	U-box domain-containing 12-like isoform X1 [Nicotiana sylvestris]	309	gi|743814916|ref|XP_011019814.1|PREDICTED: U-box domain-containing protein 10-like [Populus euphratica]	XP_011019814	1.17E-166	309	279
Pe3F10.4	1 isoform	+	9	5203	771	Acyl- thioesterase 2-like [Populus euphratica]	256	gi|743841444|ref|XP_011026469.1|PREDICTED: acyl-protein thioesterase 2-like [Populus euphratica]gi|743841448|ref|XP_011026470.1|PREDICTED: acyl-protein thioesterase 2-like [Populus euphratica]gi|743841452|ref|XP_011026471.1|PREDICTED: acyl-protein thioesterase 2-like [Populus euphratica]gi|743841458|ref|XP_011026472.1|PREDICTED: acyl-protein thioesterase 2-like [Populus euphratica]	XP_011026469, XP_011026470, XP_011026471, XP_011026472	2.14E-159	256	235
Pe3F10.5	1 isoform	-	8	4803	2013	Tesmin TSO1-like CXC domain-containing isoform	670	gi|802627270|ref|XP_012076620.1|PREDICTED: CRC domain-containing protein TSO1-like	XP_012076620, KDP33626	1.96E-140	650	371

						1 [Theobroma cacao]		isoform X1 [Jatropha curcas]gi|643724425|gb|KDP33626.1|hypothetical protein JCGZ_07197 [Jatropha curcas]				
Pe3F10.6	1 isoform	+	2	2324	468	Zinc finger family [Populus trichocarpa]	155	gi|802627276|ref|XP_012076622.1|PREDICTED: zinc finger Ran-binding domain-containing protein 2 [Jatropha curcas]gi|643724426|gb|KDP33627.1|hypothetical protein JCGZ_07198 [Jatropha curcas]	XP_012076622, KDP33627	3.63E-74	148	131
Pe3F10.7		-	3	829	648	PREDICTED: uncharacterized protein LOC105637663 [Jatropha curcas]	215	gi|802627228|ref|XP_012076604.1|PREDICTED: uncharacterized protein LOC105637663 [Jatropha curcas]gi|643724415|gb|KDP33616.1|hypothetical protein JCGZ_07187 [Jatropha curcas]	XP_012076604, KDP33616	1.23E-120	216	199
Pe3F10.8		+	4	2190	1026	Overexpressor of cationic peroxidase 3	341	gi|743892641|ref|XP_011039713.1|PREDICTED: proline-, glutamic acid- and leucine-rich protein 1 [Populus euphratica]	XP_011039713	2.80E-104	328	241
Pe3F10.9		+	1	652	285	Overexpressor of cationic peroxidase [Theobroma cacao]	102	gi|566213666|ref|XP_002324288.2|hypothetical protein POPTR_0018s01520g [Populus trichocarpa]gi|550317796|gb|EEF02853.2|hypothetical protein POPTR_0018s01520g [Populus trichocarpa]	XP_002324288, EEF02853	8.93E-20	97	65
Pe3F10.10		+	1	1767	1767	Pentatricopeptide repeat-containing At3g56550 [Cucumis melo]	588	gi|566208377|ref|XP_002323212.2|pentatricopeptide repeat-containing family protein [Populus trichocarpa]gi|550320693|gb|EEF04973.2|pentatricopeptide repeat-containing family protein [Populus trichocarpa]	XP_002323212, EEF04973	0.0	590	483
Pe3F10.11		+	4	1936	513	Bzip with a Ring-finger motif family [Populus trichocarpa]	170	gi|224141881|ref|XP_002324289.1|bZIP with a Ring-finger motif family protein [Populus trichocarpa]gi|222865723|gb|EEF02854.1|bZIP with a Ring-finger motif family protein [Populus trichocarpa]	XP_002324289, EEF02854	1.22E-74	168	149
Pe3F10.12		+	14	6552	3252	Cellulose synthase A catalytic subunit 1 [UDP-forming]	1083	gi|802627222|ref|XP_012076601.1|PREDICTED: cellulose synthase A catalytic subunit 1 [UDP-forming] [Jatropha curcas]gi|643724411|gb|KDP33612.1|hypothetical protein JCGZ_07183 [Jatropha curcas]	XP_012076601, KDP33612	0.0	1084	1055
Pe3F10.13	1 isoform	+	5	1890	405	Alba DNA RNA-binding [Theobroma cacao]	134	gi|802627219|ref|XP_012076600.1|PREDICTED: uncharacterized protein At2g34160-like [Jatropha curcas]gi|643724410|gb|KDP33611.1|hypothetical protein JCGZ_07182 [Jatropha curcas]	XP_012076600, KDP33611	7.07E-70	134	122
Pe3F10.14		+	3	819	399	EG45-like domain containing	132	gi|743841383|ref|XP_011026456.1|PREDICTED: EG45-like domain containing protein 2 [Populus euphratica]	XP_011026456	1.03E-65	132	116
Pe7M15.1		-	1	477	477	---Na---	158	No Blast Hit				
Pe7M15.2		+	1	1384	693	Gag protease poly [Theobroma cacao]	230	gi|590689992|ref|XP_007043384.1|Gag protease polyprotein [Theobroma cacao]gi|508707319|gb|EOX99215.1|Gag protease polyprotein [Theobroma cacao]	XP_007043384, EOX99215	4.49E-09	87	53
Pe7M15.3		-	1	213	213	---Na---	70	No Blast Hit				
Pe7M15.4		-	1	1041	306	---Na---	101	No Blast Hit				
Pe7M15.5		+	1	808	495	---Na---	164	No Blast Hit				
Pe7M15.6		+	1	873	513	---Na---	170	No Blast Hit				
Pe7M15.7		-	1	276	276	DNA RNA polymerases superfamily [Theobroma	91	gi|46576009|gb|AAT01370.1|putative non-LTR retroelement reverse transcriptase [Oryza sativa	AAT01370	4.18E-02	63	32

						cacao]		Japonica Group]				
Pe7M15.8		-	1	483	483	Gag protease poly [Theobroma cacao]	160	gi|590633454|ref|XP_007028113.1|Gag protease polyprotein [Theobroma cacao]gi|508716718|gb|EOY08615.1|Gag protease polyprotein [Theobroma cacao]	XP_007028113, EOY08615	4.59E-23	141	84
Pe7M15.9		-	1	525	525	DNA RNA polymerases superfamily [Theobroma cacao]	174	gi|590617810|ref|XP_007023888.1|DNA/RNA polymerases superfamily protein [Theobroma cacao]gi|508779254|gb|EOY26510.1|DNA/RNA polymerases superfamily protein [Theobroma cacao]	XP_007023888, EOY26510	9.03E+01	105	49
Pe7M15.10		-	3	1228	663	---Na---	220	No Blast Hit				
Pe7M15.11		-	1	3626	825	Probable CCR4-associated factor 1 homolog 7	274	gi|802626869|ref|XP_012076450.1|PREDICTED : probable CCR4-associated factor 1 homolog 7 [Jatropha curcas]gi|643724329|gb|KDP33530.1|hypothetica l protein JCGZ_07101 [Jatropha curcas]	XP_012076450, KDP33530	8.24E-174	275	262
Pe7M15.12		+	2	3090	1551	ARM repeat superfamily isoform 1 [Theobroma cacao]	516	gi|802626863|ref|XP_012076447.1|PREDICTED : U-box domain-containing protein 12-like [Jatropha curcas]gi|643724327|gb|KDP33528.1|hypothetica l protein JCGZ_07099 [Jatropha curcas]	XP_012076447, KDP33528	0.0	521	434
Pe7M15.13		-	6	2583	663	Stromal cell-derived factor 2	220	gi|802626856|ref|XP_012076445.1|PREDICTED : stromal cell-derived factor 2-like protein [Jatropha curcas]gi|643724325|gb|KDP33526.1|hypothetica l protein JCGZ_07097 [Jatropha curcas]	XP_012076445, KDP33526	1.93E-125	221	203
Pe7M15.15	1 isoform	+	14	6623	2934	Nucleic acid binding isoform 1 [Theobroma cacao]	977	gi|802626847|ref|XP_012076441.1|PREDICTED : uncharacterized protein LOC105637564 [Jatropha curcas]gi|802626850|ref|XP_012076443.1|PRED ICTED: uncharacterized protein LOC105637564 [Jatropha curcas]gi|643724323|gb|KDP33524.1|hypothetica l protein JCGZ_07095 [Jatropha curcas]	XP_012076441, XP_012076443, KDP33524	0.0	980	681
Pe7M15.16	2 isoforms	-	7	8294	3495	DNA-directed RNA polymerases IV and V subunit 2-like	1164	gi|743882218|ref|XP_011036682.1|PREDICTED : DNA-directed RNA polymerases IV and V subunit 2-like [Populus euphratica]	XP_011036682	0.0	1158	1028
Pe7M15.17		+	1	2092	1359	Calcineurin B -interacting kinase	452	gi|566178522|ref|XP_002308703.2|CBL-interacting protein kinase 25 [Populus trichocarpa]gi|550337238|gb|EEE92226.2|CBL-interacting protein kinase 25 [Populus trichocarpa]	XP_002308703, EEE92226	0.0	447	410
Pe7M15.18		+	5	2576	912	WRKY transcription factor family [Populus trichocarpa]	303	gi|743882190|ref|XP_011036673.1|PREDICTED : probable WRKY transcription factor 40 isoform X1 [Populus euphratica]	XP_011036673	2.42E-131	322	245
Pe7M15.19		+	4	1049	762	Probable WRKY transcription factor 40	253	gi|147864790|emb|CAN84058.1|hypothetical protein VITISV_036455 [Vitis vinifera]	CAN84058	3.00E-56	252	161
Pe7M15.20	6 isoforms	-	15	9031	2709	Formin 18	894	gi|731386288|ref|XP_010648823.1|PREDICTED : formin-like protein 18 [Vitis vinifera]	XP_010648823	0.0	471	394
Pe7M15.21	Incomplete 3'	+	3	1499	1071	POLAR LOCALIZATION DURING ASYMMETRIC DIVISION AND REDISTRIBUTION-like	357	gi|566213862|ref|XP_002324329.2|hypothetical protein POPTR_0018s02510g [Populus trichocarpa]gi|550317880|gb|EEF02894.2|hypoth etical protein POPTR_0018s02510g [Populus trichocarpa]	XP_002324329, EEF02894	5.32E-59	314	203
Pe9E4.1		+	1	471	471	---Na---	156	No Blast Hit				

Pe9E4.2		+	1	405	405	NETWORKED 1D-like	134	gi|566172886|ref|XP_002306789.2|hypothetical protein POPTR_0005s23510g [Populus trichocarpa]gi|550339604|gb|EEE93785.2|hypothetical protein POPTR_0005s23510g [Populus trichocarpa]	XP_002306789, EEE93785	1.77E-17	99	72
Pe9E4.3		-	1	408	408	Ribonuclease H At1g65750 partial	135	gi|731324777|ref|XP_010673150.1|PREDICTED: uncharacterized protein LOC104889591 [Beta vulgaris subsp. vulgaris]	XP_010673150	5.53E-09	132	62
Pe9E4.4		-	2	778	672	---Na---	223	No Blast Hit				
Pe9E4.5		+	1	342	342	---Na---	113	No Blast Hit				
Pe9E4.6		-	2	1247	1146	Replication factor C subunit 3	381	gi|734427908|gb|KHN44528.1|hypothetical protein glysoja_045970, partial [Glycine soja]	KHN44528	3.27E-09	322	148
Pe9E4.7	2 isoforms	+	14	5502	2565	Auxin response factor 2 family [Populus trichocarpa]	854	gi|224122162|ref|XP_002318767.1|auxin response factor 2 family protein [Populus trichocarpa]gi|222859440|gb|EEE96987.1|auxin response factor 2 family protein [Populus trichocarpa]	XP_002318767, EEE96987	0.0	863	716
Pe9E4.8		-	4	3640	1638	Transcription factor ICE1-like	545	gi|743894031|ref|XP_011040262.1|PREDICTED: transcription factor ICE1-like [Populus euphratica]	XP_011040262	0.0	565	482
Pe9E4.9	1 isoform	+	10	6100	2898	ATP-dependent clp protease ATP-binding subunit clpa family [Populus trichocarpa]	965	gi|743894019|ref|XP_011040255.1|PREDICTED: chaperone protein ClpC, chloroplastic [Populus euphratica]gi|743894021|ref|XP_011040256.1|PREDICTED: chaperone protein ClpC, chloroplastic [Populus euphratica]	XP_011040255, XP_011040256	0.0	919	888
Pe15E1.1		+	1	375	375	---Na---	124	No Blast Hit				
Pe15E1.2		+	1	270	270	---Na---	89	No Blast Hit				
Pe15E1.3		-	2	502	426	Gag protease poly [Theobroma cacao]	141	gi|590648106|ref|XP_007032083.1|Gag protease polyprotein [Theobroma cacao]gi|508711112|gb|EOY03009.1|Gag protease polyprotein [Theobroma cacao]	XP_007032083, EOY03009	3.92E-06	155	67
Pe15E1.4		-	2	1132	594	---Na---	197	No Blast Hit				
Pe15E1.5		+	5	2559	1512	Hypothetical protein KK1_034167	503	gi|1012332930|gb|KYP44342.1|hypothetical protein KK1_034167 [Cajanus cajan]	KYP44342	2.19E+00	82	41
Pe15E1.6		+	1	573	573	PREDICTED: uncharacterized protein LOC107616310	190	gi|1021564842|ref|XP_016173772.1|PREDICTED: uncharacterized protein LOC107616310 [Arachis ipaensis]	XP_016173772	1.25E-21	181	94
Pe15E1.7		+	1	537	537	Ribonuclease H At1g65750 partial	178	gi|1012359710|gb|KYP70894.1|Putative ribonuclease H protein At1g65750 family, partial [Cajanus cajan]	KYP70894	2.44E-18	177	94
Pe15E1.8		+	13	2896	1578	Beta-glucosidase 12-like	525	gi|601587996|gb|AHN85654.1|glycoside hydrolase family 1 [Drypetes roxburghii]	AHN85654	0.0	522	376
Pe15E1.9		-	4	1537	567	(+)-Neomenthol dehydrogenase-like	188	gi|802561017|ref|XP_012066288.1|PREDICTED: short-chain dehydrogenase/reductase 2b-like [Jatropha curcas]gi|802561019|ref|XP_012066289.1|PREDICTED: short-chain dehydrogenase/reductase 2b-like [Jatropha curcas]gi|643736616|gb|KDP42906.1|hypothetical protein JCGZ_23848 [Jatropha curcas]	XP_012066288, XP_012066289, KDP42906	3.19E-94	179	160
Pe20E10.1		+	1	159	159	---Na---	52	No Blast Hit				
Pe20E10.2		+	2	351	258	---Na---	85	No Blast Hit				
Pe20E10.3		+	2	466	291	---Na---	96	No Blast Hit				

Pe20E10.4		+	1	1578	1578	Gag protease poly [Theobroma cacao]	525	gi|590581218|ref|XP_007014287.1|Gag protease polyprotein [Theobroma cacao]gi|508784650|gb|EOY31906.1|Gag protease polyprotein [Theobroma cacao]	XP_007014287, EOY31906	4.38E-49	322	167
Pe20E10.5		+	2	323	231	Retrovirus-related Pol poly from transposon TNT 1-94	76	gi|923839771|ref|XP_013700505.1|PREDICTED : uncharacterized protein LOC106404326 [Brassica napus]	XP_013700505	2.40E-02	50	34
Pe20E10.6		+	1	333	333	Probable pectinesterase pectinesterase inhibitor 13	110	gi|720098741|ref|XP_010247726.1|PREDICTED : uncharacterized protein LOC104590695 [Nelumbo nucifera]	XP_010247726	2.62E-09	81	50
Pe20E10.7		+	2	816	459	Uncharacterized protein TCM_012596 [Theobroma cacao]	152	gi|590665339|ref|XP_007036712.1|Uncharacteriz ed protein TCM_012596 [Theobroma cacao]gi|508773957|gb|EOY21213.1|Uncharacter ized protein TCM_012596 [Theobroma cacao]	XP_007036712, EOY21213	2.58E-01	63	40
Pe20E10.8		-	2	1000	735	DNA RNA polymerases superfamily [Theobroma cacao]	244	gi|672145351|ref|XP_008796591.1|PREDICTED : LOW QUALITY PROTEIN: uncharacterized protein LOC103712006 [Phoenix dactylifera]	XP_008796591	1.43E-22	152	90
Pe20E10.9		+	1	423	423	---Na---	140	No Blast Hit				
Pe20N3+ 64C12.1	Incomplete 3'	-	12	6941	1935	Histone-lysine N-methyltransferase ATX2 isoform X1 [Populus euphratica]	645	gi|802560335|ref|XP_012066265.1|PREDICTED : histone-lysine N-methyltransferase ATX2-like [Jatropha curcas]gi|643736599|gb|KDP42889.1|hypothetica l protein JCGZ_23831 [Jatropha curcas]	XP_012066265, KDP42889	0.0	667	520
Pe20N3+ 64C12.2		-	4	1172	729	Hypothetical protein POPTR_0014s14360g [Populus trichocarpa]	242	gi|224131452|ref|XP_002321088.1|hypothetical protein POPTR_0014s14360g [Populus trichocarpa]gi|222861861|gb|EEE99403.1|hypoth etical protein POPTR_0014s14360g [Populus trichocarpa]	XP_002321088, EEE99403	5.39E-105	245	197
Pe20N3+ 64C12.3		+	5	2575	1266	Probable phosphatase 2C 33	421	gi|659089831|ref|XP_008445704.1|PREDICTED : probable protein phosphatase 2C 33 [Cucumis melo]	XP_008445704	4.14E-162	387	290
Pe20N3+ 64C12.4		-	8	3864	1245	Defective in meristem silencing 3	414	gi|566196389|ref|XP_002318428.2|hypothetical protein POPTR_0012s02300g [Populus trichocarpa]gi|550326211|gb|EEE96648.2|hypoth etical protein POPTR_0012s02300g [Populus trichocarpa]	XP_002318428, EEE96648	0.0	409	321
Pe20N3+ 64C12.5		+	3	2068	1266	GDSL esterase lipase [Theobroma cacao]	421	gi|255539020|ref|XP_002510575.1|PREDICTED : uncharacterized protein LOC8274702 [Ricinus communis]gi|223551276|gb|EEF52762.1|conserv ed hypothetical protein [Ricinus communis]	XP_002510575, EEF52762	5.57E-174	425	319
Pe20N3+ 64C12.6		-	9	3381	2307	CO(2)-response secreted protease-like	768	gi|743875606|ref|XP_011034984.1|PREDICTED : CO(2)-response secreted protease [Populus euphratica]	XP_011034984	0.0	772	608
Pe20N3+ 64C12.7		+	4	1427	951	Peroxidase 64-like	316	gi|743875594|ref|XP_011034979.1|PREDICTED : peroxidase 64-like [Populus euphratica]	XP_011034979	0.0	316	289
Pe20N3+ 64C12.8		-	8	3435	753	Ribonuclease P subunit p25 [Populus euphratica]	250	gi|1012187450|ref|XP_015969816.1|PREDICTE D: ribonuclease P protein subunit p25-like protein [Arachis duranensis]gi|1021522840|ref|XP_016204821.1|P REDICTED: ribonuclease P protein subunit p25-like protein [Arachis ipaensis]	XP_015969816, XP_016204821	1.82E-107	254	221
Pe20N3+ 64C12.9	1 isoform	+	9	4140	1236	Maltose excess chloroplastic-like	411	gi|1000955636|ref|XP_015577727.1|PREDICTE D: maltose excess protein 1, chloroplastic isoform X1 [Ricinus communis]	XP_015577727	0.0	415	336
Pe20N3+ 64C12.10		+	1	2364	2364	Pentatricopeptide repeat-containing At1g20230	787	gi|225459736|ref|XP_002284744.1|PREDICTED : pentatricopeptide repeat-containing protein At1g20230 [Vitis	XP_002284744, XP_010664142, XP_010664143,	0.0	758	629

								vinifera]gi|731427875|ref|XP_010664142.1|PREDICTED: pentatricopeptide repeat-containing protein At1g20230 [Vitis vinifera]gi|731427877|ref|XP_010664143.1|PREDICTED: pentatricopeptide repeat-containing protein At1g20230 [Vitis vinifera]gi|731427879|ref|XP_010664144.1|PREDICTED: pentatricopeptide repeat-containing protein At1g20230 [Vitis vinifera]	XP_010664144			
Pe20N3+ 64C12.11		-	1	441	441	60S ribosomal L26-1-like	146	gi|802645921|ref|XP_012079404.1|PREDICTED: 60S ribosomal protein L26-1 [Jatropha curcas]gi|643722194|gb|KDP32073.1|hypothetical protein JCGZ_12534 [Jatropha curcas]	XP_012079404, KDP32073	7.30E-91	146	145
Pe20N3+ 64C12.12	1 isoform	+	8	3327	1569	Calcium-dependent kinase 29	522	gi|255539058|ref|XP_002510594.1|PREDICTED: calcium-dependent protein kinase 29 [Ricinus communis]gi|223551295|gb|EEF52781.1|calcium-dependent protein kinase, putative [Ricinus communis]	XP_002510594, EEF52781	0.0	524	465
Pe20N3+ 64C12.13		-	7	3995	870	Probable prolyl 4-hydroxylase 3	289	gi|566173388|ref|XP_002307750.2|oxidoreductase family protein [Populus trichocarpa]gi|550339795|gb|EEE94746.2|oxidoreductase family protein [Populus trichocarpa]	XP_002307750, EEE94746	0.0	289	268
Pe20N3+ 64C12.14		+	1	1665	1665	Pentatricopeptide repeat-containing mitochondrial	554	gi|743918175|ref|XP_011003090.1|PREDICTED: pentatricopeptide repeat-containing protein At1g20300, mitochondrial-like [Populus euphratica]	XP_011003090	0.0	542	459
Pe20N3+ 64C12.15		-	1	906	906	Thioredoxin chloroplastic	301	gi|255539068|ref|XP_002510599.1|PREDICTED: thioredoxin-like protein CDSP32, chloroplastic [Ricinus communis]gi|223551300|gb|EEF52786.1|conserved hypothetical protein [Ricinus communis]	XP_002510599, EEF52786	2.23E-162	302	267
Pe20N3+ 64C12.16		-	1	2275	1089	24-methylenesterol C-methyltransferase 2	362	gi|1009109012|ref|XP_015887788.1|PREDICTED: 24-methylenesterol C-methyltransferase 2 [Ziziphus jujuba]	XP_015887788	0.0	362	343
Pe20N3+ 64C12.17		-	2	737	276	---Na---	91	No Blast Hit				
Pe20N3+ 64C12.18		-	3	1320	1155	---Na---	373	No Blast Hit				
Pe21O15.1		-	1	258	258	---Na---	85	No Blast Hit				
Pe21O15.2		-	3	811	426	---Na---	141	No Blast Hit				
Pe21O15.3		-	1	189	189	---Na---	62	No Blast Hit				
Pe21O15.4	6 isoforms	+	13	4938	1290	Glutamine synthetase leaf chloroplastic	429	gi|255551511|ref|XP_002516801.1|PREDICTED: glutamine synthetase leaf isozyme, chloroplastic [Ricinus communis]gi|1000971495|ref|XP_015573327.1|PREDICTED: glutamine synthetase leaf isozyme, chloroplastic [Ricinus communis]gi|1000971498|ref|XP_015573328.1|PREDICTED: glutamine synthetase leaf isozyme, chloroplastic [Ricinus communis]gi|223543889|gb|EEF45415.1|glutamine synthetase plant, putative [Ricinus communis]	XP_002516801, XP_015573327, XP_015573328, EEF45415	0.0	432	414
Pe21O15.5	1 isoform	+	4	1052	510	AWPM-19 [Arabidopsis thaliana]	169	gi|802578887|ref|XP_012069489.1|PREDICTED: uncharacterized protein LOC105631891 [Jatropha curcas]gi|643733135|gb|KDP40082.1|hypothetica	XP_012069489, KDP40082	1.28E-76	162	140

								l protein JCGZ_02080 [Jatropha curcas]				
Pe21O15.6		-	1	468	468	DNA RNA polymerases superfamily [Theobroma cacao]	155	gi|590693137|ref|XP_007044250.1|DNA/RNA polymerases superfamily protein [Theobroma cacao]gi|508708185|gb|EOY00082.1|DNA/RNA polymerases superfamily protein [Theobroma cacao]	XP_007044250, EOY00082	2.47E+00	166	68
Pe21O15.7		-	1	228	228	---Na---	75	No Blast Hit				
Pe21O15.8		-	4	722	156	---Na---	51	No Blast Hit				
Pe21O15.9		-	2	308	231	---Na---	76	No Blast Hit				
Pe21O15.10		-	1	417	417	Gag protease poly [Theobroma cacao]	138	gi|590714090|ref|XP_007049818.1|Gag protease polyprotein [Theobroma cacao]gi|508702079|gb|EOX93975.1|Gag protease polyprotein [Theobroma cacao]	XP_007049818, EOX93975	5.23E-08	141	68
Pe21O15.11		-	1	453	453	---Na---	150	No Blast Hit				
Pe21O15.12		+	1	474	474	---Na---	157	No Blast Hit				
Pe21O15.13		+	4	2031	735	Bi1	244	gi|802578866|ref|XP_012069478.1|PREDICTED: BI1-like protein [Jatropha curcas]gi|643733126|gb|KDP40073.1|hypothetical protein JCGZ_02071 [Jatropha curcas]	XP_012069478, KDP40073	2.02E-152	246	233
Pe21O15.14		+	4	1472	750	BI1 [Ziziphus jujuba]	249	gi|573944042|ref|XP_006654376.1|PREDICTED: BI1-like protein [Oryza brachyantha]	XP_006654376	4.74E-32	231	123
Pe21O15.15	1 isoform	-	11	5814	1038	Calcium-binding 39	345	gi|1009177503|ref|XP_015870008.1|PREDICTED: calcium-binding protein 39 [Ziziphus jujuba]	XP_015870008	0.0	345	314
Pe21O15.16	4 isoforms	+	8	4570	1902	AP-5 complex subunit mu	633	gi|802578674|ref|XP_012069473.1|PREDICTED: AP-5 complex subunit mu [Jatropha curcas]gi|643733122|gb|KDP40069.1|hypothetical protein JCGZ_02067 [Jatropha curcas]	XP_012069473, KDP40069	0.0	631	557
Pe24G19.1		+	5	3435	1524	Gag protease poly [Theobroma cacao]	507	gi|590728434|ref|XP_007099662.1|Gag protease polyprotein-like protein [Theobroma cacao]gi|508728474|gb|EOY20371.1|Gag protease polyprotein-like protein [Theobroma cacao]	XP_007099662, EOY20371	3.74E-16	171	88
Pe24G19.2		+	1	759	759	PREDICTED: uncharacterized protein LOC107261398	252	gi|1000962090|ref|XP_015575809.1|PREDICTED: uncharacterized protein LOC107261398 [Ricinus communis]	XP_015575809	7.98E-75	254	165
Pe24G19.3		-	1	714	714	---Na---	237	No Blast Hit				
Pe24G19.4		+	1	528	528	---Na---	175	No Blast Hit				
Pe24G19.5		+	1	375	375	Gag protease poly [Theobroma cacao]	124	gi|590693137|ref|XP_007044250.1|DNA/RNA polymerases superfamily protein [Theobroma cacao]gi|508708185|gb|EOY00082.1|DNA/RNA polymerases superfamily protein [Theobroma cacao]	XP_007044250, EOY00082	3.49E+00	118	51
Pe24G19.6		+	1	462	462	---Na---	153	No Blast Hit				
Pe24G19.7		-	1	207	207	---Na---	68	No Blast Hit				
Pe24G19.8		+	1	414	414	PREDICTED: uncharacterized protein LOC104605763	137	gi|720041566|ref|XP_010268950.1|PREDICTED: uncharacterized protein LOC104605763 [Nelumbo nucifera]	XP_010268950	7.39E-27	142	90
Pe24G19.9		+	1	177	177	---Na---	58	No Blast Hit				
Pe24G19.10		+	2	1321	660	---Na---	219	No Blast Hit				
Pe24G19.11		+	1	165	165	---Na---	54	No Blast Hit				

Pe24G19.12	+	1	534	534	---Na---	177	No Blast Hit				
Pe24G19.13	+	2	601	372	---Na---	123	No Blast Hit				
Pe24G19.14	-	1	276	276	---Na---	91	No Blast Hit				
Pe24G19.15	-	1	417	417	---Na---	138	No Blast Hit				
Pe24G19.16	+	5	3803	1593	Sugar transport 13	530	gi|255552343|ref|XP_002517216.1|PREDICTED: sugar transport protein 13 [Ricinus communis]gi|223543851|gb|EEF45379.1|sugar transporter, putative [Ricinus communis]	XP_002517216, EEF45379	0.0	531	496
Pe24G19.17	+	2	3089	558	Plastocyanin-like domain [Medicago truncatula]	185	gi|224108071|ref|XP_002314710.1|hypothetical protein POPTR_0010s10020g [Populus trichocarpa]gi|118485573|gb|ABK94638.1|unknown [Populus trichocarpa]gi|222863750|gb|EEF00881.1|hypothetical protein POPTR_0010s10020g [Populus trichocarpa]	XP_002314710, ABK94638, EEF00881	1.69E-63	186	143
Pe24G19.18	-	6	1705	603	Chloride channel CLC-d isoform X2	200	gi|255552337|ref|XP_002517213.1|PREDICTED: chloride channel protein CLC-d isoform X3 [Ricinus communis]gi|223543848|gb|EEF45376.1|chloride channel clc, putative [Ricinus communis]	XP_002517213, EEF45376	5.17E-104	178	171
Pe27H17.1	+	1	288	288	---Na---	95	No Blast Hit				
Pe27H17.2	-	1	228	228	---Na---	75	No Blast Hit				
Pe27H17.3	+	1	1207	1026	---Na---	341	No Blast Hit				
Pe27H17.4	+	3	2134	216	---Na---	71	No Blast Hit				
Pe27H17.5	+	1	630	630	Gag protease poly [Theobroma cacao]	209	gi|590689992|ref|XP_007043384.1|Gag protease polyprotein [Theobroma cacao]gi|508707319|gb|EOX99215.1|Gag protease polyprotein [Theobroma cacao]	XP_007043384, EOX99215	2.38E-24	203	102
Pe27H17.6	-	1	207	207	---Na---	68	No Blast Hit				
Pe27H17.7	+	1	652	522	---Na---	173	No Blast Hit				
Pe27H17.8	-	1	177	177	---Na---	58	No Blast Hit				
Pe27H17.9	+	1	336	336	PREDICTED: uncharacterized protein LOC104589505	111	gi|720093782|ref|XP_010246157.1|PREDICTED: uncharacterized protein LOC104589505 [Nelumbo nucifera]	XP_010246157	1.83E-03	111	56
Pe27H17.10	-	1	312	312	---Na---	103	No Blast Hit				
Pe27H17.11	+	1	1071	1071	PREDICTED: uncharacterized protein LOC103328423	356	gi|645245765|ref|XP_008229034.1|PREDICTED: uncharacterized protein LOC103328423 [Prunus mume]	XP_008229034	3.63E-79	340	213
Pe27H17.12	+	2	840	642	PREDICTED: uncharacterized protein LOC107620531	213	gi|1021574018|ref|XP_016178162.1|PREDICTED: uncharacterized protein LOC107620531 [Arachis ipaensis]	XP_016178162	2.04E-22	185	104
Pe27H17.13	+	1	777	777	S-locus lectin kinase family [Theobroma cacao]	258	gi|734399979|gb|KHN31113.1|hypothetical protein glysoja_046590, partial [Glycine soja]	KHN31113	3.71E-09	283	121
Pe27H17.14	+	1	234	234	---Na---	77	No Blast Hit				
Pe27H17.15	+	2	435	366	---Na---	121	No Blast Hit				
Pe27H17.16	+	2	755	594	Retrotransposon Ty1-copia subclass	197	gi|823155331|ref|XP_012477568.1|PREDICTED: uncharacterized protein LOC105793188 [Gossypium raimondii]	XP_012477568	2.55E-48	176	120
Pe27H17.17	+	1	267	267	Retrotransposon Ty1-copia	88	gi|823155331|ref|XP_012477568.1|PREDICTED	XP_012477568	3.80E-16	85	60

						subclass		: uncharacterized protein LOC105793188 [Gossypium raimondii]				
Pe28D11.1		+	1	925	678	PREDICTED: uncharacterized protein LOC103328423	225	gi|645245765|ref|XP_008229034.1|PREDICTED : uncharacterized protein LOC103328423 [Prunus mume]	XP_008229034	2.59E-46	225	142
Pe28D11.2		+	1	687	687	Retrovirus-related Pol poly from transposon opus	228	gi|1021504437|ref|XP_016195524.1|PREDICTED: uncharacterized protein LOC107636536 [Arachis ipaensis]	XP_016195524	2.91E-45	221	134
Pe28D11.3		-	1	216	216	---Na---	71	No Blast Hit				
Pe28D11.4		+	1	1730	1239	S-locus lectin kinase family [Theobroma cacao]	412	gi|734399979|gb|KHN31113.1|hypothetical protein glysoja_046590, partial [Glycine soja]	KHN31113	3.38E-10	286	121
Pe28D11.5		+	1	192	192	---Na---	63	No Blast Hit				
Pe28D11.6		+	1	516	516	---Na---	171	No Blast Hit				
Pe28D11.7		-	1	357	357	---Na---	118	No Blast Hit				
Pe28D11.8		-	1	414	414	---Na---	137	No Blast Hit				
Pe28D11.9		-	1	189	189	---Na---	62	No Blast Hit				
Pe28D11.10		-	2	2430	549	---Na---	182	No Blast Hit				
Pe28D11.11		+	4	2241	804	PREDICTED: uncharacterized protein LOC105764707	267	gi|823127726|ref|XP_012438865.1|PREDICTED : uncharacterized protein LOC105764707 [Gossypium raimondii]	XP_012438865	1.97E-01	197	81
Pe28D11.12		+	1	954	954	Cytochrome P450 82C4-like	317	gi|255538466|ref|XP_002510298.1|PREDICTED : cytochrome P450 82C4 [Ricinus communis]gi|223550999|gb|EEF52485.1|cytochrome P450, putative [Ricinus communis]	XP_002510298, EEF52485	3.44E-119	323	233
Pe28D11.13		-	1	711	615	Retrovirus-related Pol poly from transposon TNT 1-94	204	gi|147798561|emb|CAN76689.1|hypothetical protein VITISV_001362 [Vitis vinifera]	CAN76689	1.23E-63	157	127
Pe28D11.14		+	1	319	201	---Na---	66	No Blast Hit				
Pe28D11.15		-	1	333	333	---Na---	110	No Blast Hit				
Pe28D11.16		-	2	1083	996	Gag protease poly [Theobroma cacao]	331	gi|590728434|ref|XP_007099662.1|Gag protease polyprotein-like protein [Theobroma cacao]gi|508728474|gb|EOY20371.1|Gag protease polyprotein-like protein [Theobroma cacao]	XP_007099662, EOY20371	2.07E-41	364	169
Pe28D11.17		-	1	375	375	---Na---	124	No Blast Hit				
Pe28D11.18		-	1	225	225	---Na---	74	No Blast Hit				
Pe28D11.19		-	1	210	210	---Na---	69	No Blast Hit				
Pe28D11.20		+	2	1506	1410	Gag protease poly [Theobroma cacao]	469	gi|590689992|ref|XP_007043384.1|Gag protease polyprotein [Theobroma cacao]gi|508707319|gb|EOX99215.1|Gag protease polyprotein [Theobroma cacao]	XP_007043384, EOX99215	2.45E-21	164	87
Pe28E22.1	1 isoform	+	7	3547	1623	Transparent testa 12	540	gi|567910471|ref|XP_006447549.1|hypothetical protein CICLE_v10014926mg [Citrus clementina]gi|568830830|ref|XP_006469688.1|PREDICTED: protein DETOXIFICATION 40 [Citrus sinensis]gi|557550160|gb|ESR60789.1|hypothetical protein CICLE_v10014926mg [Citrus clementina]	XP_006447549, XP_006469688, ESR60789	0.0	515	427
Pe28E22.2		+	1	648	648	PREDICTED: uncharacterized protein	215	gi|697150105|ref|XP_009629264.1|PREDICTED : uncharacterized protein LOC104119453	XP_009629264	2.59E-04	102	55

						LOC104119453		[Nicotiana tomentosiformis]				
Pe28E22.3		+	2	2165	2085	AF525305_2 AP endonuclease reverse transcriptase	694	gi|731373493|ref|XP_010666661.1|PREDICTED: uncharacterized protein LOC104883796 [Beta vulgaris subsp. vulgaris]	XP_010666661	9.49E-153	707	394
Pe28E22.4		+	2	3495	696	Ribonuclease H At1g65750	231	gi|685297784|ref|XP_009139674.1|PREDICTED: putative ribonuclease H protein At1g65750 [Brassica rapa]	XP_009139674	5.27E-24	237	110
Pe28E22.5	6 isoforms	+	6	3901	2718	Glutamate receptor	905	gi|225447292|ref|XP_002273713.1|PREDICTED: glutamate receptor 3.7 [Vitis vinifera]	XP_002273713	0.0	912	748
Pe28E22.6	3 isoforms	-	3	2246	264	Hexokinase-1 [Gossypium arboreum]	87	gi|802732166|ref|XP_012086338.1|PREDICTED: uncharacterized protein LOC105645369 [Jatropha curcas]gi|802732171|ref|XP_012086339.1|PREDICTED: uncharacterized protein LOC105645369 [Jatropha curcas]	XP_012086338, XP_012086339	1.32E-51	87	85
Pe28E22.7		+	2	981	516	Transmembrane 97-like	171	gi|645247952|ref|XP_008230078.1|PREDICTED: transmembrane protein 97-like [Prunus mume]	XP_008230078	2.67E-67	171	133
Pe28E22.8		-	11	4604	2286	Homeobox-leucine zipper HDG2-like isoform X5 [Populus euphratica]	761	gi|743922073|ref|XP_011005113.1|PREDICTED: homeobox-leucine zipper protein HDG2 isoform X2 [Populus euphratica]	XP_011005113	0.0	761	730
Pe28E22.9		+	3	892	438	Actin-related 8 [Jatropha curcas]	145	gi|743922077|ref|XP_011005117.1|PREDICTED: uncharacterized protein LOC105111456 [Populus euphratica]	XP_011005117	2.76E-33	142	100
Pe28E22.10	2 isoforms	-	12	7635	1377	Pentatricopeptide repeat (PPR) superfamily isoform 2 [Theobroma cacao]	458	gi|1000944192|ref|XP_015581699.1|PREDICTED: uncharacterized protein At3g49140 [Ricinus communis]	XP_015581699	0.0	454	368
Pe28E22.11		+	5	11107	933	Phosphoenolpyruvate carboxylase family isoform 2 [Theobroma cacao]	310	gi|802559892|ref|XP_012066241.1|PREDICTED: petal death protein isoform X2 [Jatropha curcas]	XP_012066241	2.18E-173	305	273
Pe28E22.12		+	2	379	261	---Na---	86	No Blast Hit				
Pe28E22.13		+	5	4563	1053	Phosphoenolpyruvate carboxylase family isoform 2 [Theobroma cacao]	350	gi|763770676|gb|KJB37891.1|hypothetical protein B456_006G227600 [Gossypium raimondii]gi|763770678|gb|KJB37893.1|hypothetical protein B456_006G227600 [Gossypium raimondii]	KJB37891, KJB37893	1.15E-170	332	285
Pe28E22.14		-	10	5092	2562	Clathrin interactor EPSIN 2 [Nelumbo nucifera]	853	gi|1000940321|ref|XP_015583028.1|PREDICTED: clathrin interactor EPSIN 3 isoform X2 [Ricinus communis]	XP_015583028	0.0	914	652
Pe28I20.1		-	1	306	306	---Na---	101	No Blast Hit				
Pe28I20.2		-	2	657	570	PREDICTED: uncharacterized protein LOC104884660	189	gi|731376695|ref|XP_010667645.1|PREDICTED: uncharacterized protein LOC104884660 [Beta vulgaris subsp. vulgaris]	XP_010667645	4.20E-36	215	126
Pe28I20.3		+	2	881	762	PREDICTED: LOW QUALITY PROTEIN: uncharacterized protein LOC107261199. partial	253	gi|1000967872|ref|XP_015574193.1|PREDICTED: LOW QUALITY PROTEIN: uncharacterized protein LOC107261199, partial [Ricinus communis]	XP_015574193	2.06E-09	83	55
Pe28I20.4		-	1	237	237	---Na---	78	No Blast Hit				
Pe28I20.5		-	1	441	441	---Na---	146	No Blast Hit				
Pe28I20.6		-	1	408	408	---Na---	135	No Blast Hit				
Pe28I20.7		+	1	339	339	---Na---	112	No Blast Hit				
Pe33M2.1		-	1	210	210	Gag pol	69	gi|763798846|gb|KJB65801.1|hypothetical protein B456_010G1135002, partial [Gossypium raimondii]	KJB65801	8.77E-05	54	32

Pe33M2.2		-	1	225	225	---Na---	74	No Blast Hit				
Pe33M2.3	Incomplete 3'	+	3	2037	697	Cytochrome P450 714A1-like	232	gi|743911302|ref|XP_010999513.1|PREDICTED: cytochrome P450 714A1-like [Populus euphratica]	XP_010999513	3.23E-110	232	193
Pe34H9.1		-	1	519	351	---Na---	116	No Blast Hit				
Pe34H9.2	1 isoform	+	8	4472	894	Probable mitochondrial import inner membrane translocase subunit TIM21	297	gi|566202703|ref|XP_006375220.1|hypothetical protein POPTR_0014s05400g [Populus trichocarpa]gi|550323539|gb|ERP53017.1|hypothetical protein POPTR_0014s05400g [Populus trichocarpa]	XP_006375220, ERP53017	3.72E-133	312	239
Pe34H9.3		+	14	6285	1623	Na+ H+ antiporter family [Populus trichocarpa]	540	gi|566169479|ref|XP_002307194.2|Na+/H+ antiporter family protein [Populus trichocarpa]gi|550338076|gb|EEE94190.2|Na+/H+ antiporter family protein [Populus trichocarpa]	XP_002307194, EEE94190	0.0	535	501
Pe34H9.4		-	2	1901	1539	Premnaspirodiene oxygenase-like	526	gi|643732064|gb|KDP39256.1|hypothetical protein JCGZ_01013 [Jatropha curcas]	KDP39256	0.0	467	368
Pe34H9.5		-	8	2997	1170	Vesicle-associated 2-2 [Ricinus communis]	389	gi|1000980541|ref|XP_015570407.1|PREDICTED: vesicle-associated protein 2-2 [Ricinus communis]gi|1000980543|ref|XP_015570408.1|PREDICTED: vesicle-associated protein 2-2 [Ricinus communis]gi|1000980545|ref|XP_015570409.1|PREDICTED: vesicle-associated protein 2-2 [Ricinus communis]gi|1000980547|ref|XP_015570410.1|PREDICTED: vesicle-associated protein 2-2 [Ricinus communis]gi|1000980549|ref|XP_015570411.1|PREDICTED: vesicle-associated protein 2-2 [Ricinus communis]gi|1000980551|ref|XP_015570412.1|PREDICTED: vesicle-associated protein 2-2 [Ricinus communis]	XP_015570407, XP_015570408, XP_015570409, XP_015570410, XP_015570411, XP_015570412	1.25E-111	414	259
Pe34H9.6		+	4	1893	420	Acyl carrier 1 [Populus trichocarpa]	139	gi|743829325|ref|XP_011023489.1|PREDICTED: acyl carrier protein 1, chloroplastic-like isoform X2 [Populus euphratica]	XP_011023489	1.75E-64	136	120
Pe34H9.7		+	6	2553	459	Cu Zn superoxide dismutase family [Populus trichocarpa]	152	gi|844625821|gb|AKN10570.1|copper/zinc-superoxide dismutase 1a [Camellia sinensis]	AKN10570	1.58E-85	152	141
Pe34H9.8		+	2	641	414	---Na---	137	No Blast Hit				
Pe34H9.9		+	2	783	699	PREDICTED: uncharacterized protein LOC107491798	232	gi|1012181105|ref|XP_015968205.1|PREDICTED: uncharacterized protein LOC107491798 [Arachis duranensis]	XP_015968205	2.96E-40	151	103
Pe34H9.10		+	2	908	825	Hypothetical protein GLYMA_17G252900	274	gi|947056405|gb|KRH05858.1|hypothetical protein GLYMA_17G252900 [Glycine max]	KRH05858	3.43E+01	185	92
Pe34H9.11		+	1	330	330	---Na---	109	No Blast Hit				
Pe34H9.12		-	1	258	258	---Na---	85	No Blast Hit				
Pe34M7.1		-	2	1201	501	---Na---	166	No Blast Hit				
Pe34M7.2		+	1	300	300	---Na---	99	No Blast Hit				
Pe34M7.3		+	4	1298	528	---Na---	175	No Blast Hit				
Pe34M7.4		+	2	440	330	---Na---	109	No Blast Hit				
Pe34M7.5		-	1	225	225	---Na---	74	No Blast Hit				

Pe34M7.6		+	2	371	264	---Na---	87	No Blast Hit				
Pe34M7.7		+	1	465	465	PREDICTED: uncharacterized protein LOC106774649	154	gi|951038589|ref|XP_014517179.1|PREDICTED: uncharacterized protein LOC106774649 [Vigna radiata var. radiata]	XP_014517179	7.52E-31	131	94
Pe34M7.8		+	1	483	483	Transposon Ty3-G Gag-Pol poly	160	gi|1027091605|ref|XP_016646912.1|PREDICTED: uncharacterized protein LOC103318979 [Prunus mume]	XP_016646912	3.32E-20	159	94
Pe34M7.9		-	1	609	609	---Na---	202	No Blast Hit				
Pe34M7.10		+	2	1131	1026	S-locus lectin kinase family [Theobroma cacao]	341	gi|1021036421|gb|KZM94204.1|hypothetical protein DCAR_017447 [Daucus carota subsp. sativus]	KZM94204	1.92E-07	213	87
Pe34M7.11		+	1	495	495	---Na---	164	No Blast Hit				
Pe34M7.12		+	2	1201	531	---Na---	176	No Blast Hit				
Pe34M7.13		+	2	637	480	Gag protease poly [Theobroma cacao]	159	gi|590689992|ref|XP_007043384.1|Gag protease polyprotein [Theobroma cacao]gi|508707319|gb|EOX99215.1|Gag protease polyprotein [Theobroma cacao]	XP_007043384, EOX99215	5.61E-03	82	42
Pe34M7.14		+	2	276	192	---Na---	64	No Blast Hit				
Pe43D2.1	Incomplete 3'	-	4	1093	686	Polyamine oxidase-like	228	gi|566206628|ref|XP_002321586.2|hypothetical protein POPTR_0015s08590g [Populus trichocarpa]gi|550322319|gb|EEF05713.2|hypothetical protein POPTR_0015s08590g [Populus trichocarpa]	XP_002321586, EEF05713	9.08E-112	229	198
Pe43D2.2		-	2	2779	1911	Probable polygalacturonase non-catalytic subunit JP650	636	gi|566197370|ref|XP_002318036.2|hypothetical protein POPTR_0012s08060g [Populus trichocarpa]gi|550326630|gb|EEE96256.2|hypothetical protein POPTR_0012s08060g [Populus trichocarpa]	XP_002318036, EEE96256	0.0	632	545
Pe43D2.3	1 isoform	+	3	1105	546	Bhlh transcription factor [Gossypium hirsutum]	181	gi|224121682|ref|XP_002318646.1|hypothetical protein POPTR_0012s08050g [Populus trichocarpa]gi|222859319|gb|EEE96866.1|hypothetical protein POPTR_0012s08050g [Populus trichocarpa]	XP_002318646. EEE96866	3.21E-65	181	143
Pe43D2.4		+	6	2750	1125	Serine threonine- kinase HT1-like	374	gi|802652381|ref|XP_012080088.1|PREDICTED: serine/threonine-protein kinase HT1-like [Jatropha curcas]gi|643720851|gb|KDP31115.1|hypothetical protein JCGZ_11491 [Jatropha curcas]	XP_012080088, KDP31115	0.0	382	357
Pe43D2.5		-	6	1449	714	Bidirectional sugar transporter SWEET5-like	237	gi|1028939638|ref|XP_016713025.1|PREDICTED: bidirectional sugar transporter SWEET5-like [Gossypium hirsutum]	XP_016713025	3.14E-106	235	191
Pe43D2.6		-	2	1381	222	Deletion of SUV3 suppressor 1(I) isoform 1 [Theobroma cacao]	73	gi|743930893|ref|XP_011009708.1|PREDICTED: probable 26S proteasome complex subunit sem1-1 [Populus euphratica]gi|118485237|gb|ABK94478.1|unknown [Populus trichocarpa]	XP_011009708, ABK94478	5.73E-13	73	68
Pe43D2.7		-	1	2306	2052	U-box domain-containing 19-like	683	gi|224119294|ref|XP_002318035.1|hypothetical protein POPTR_0012s08030g [Populus trichocarpa]gi|222858708|gb|EEE96255.1|hypothetical protein POPTR_0012s08030g [Populus trichocarpa]	XP_002318035, EEE96255	0.0	683	553
Pe43D2.8	1 isoform	+	3	6033	2586	Probable alpha,alpha-trehalose-phosphate synthase [UDP-forming] 9 [Populus euphratica]	861	gi|949801618|gb|ALN13340.1|trehalose-6-phosphate synthase-7 [Hevea brasiliensis]	ALN13340	0.0	858	767

Pe43D2.9		+	1	1782	507	Serine arginine repetitive matrix [Theobroma cacao]	168	gi|643720863|gb|KDP31127.1|hypothetical protein JCGZ_11503 [Jatropha curcas]	KDP31127	4.08E-62	173	130
Pe43D2.10		-	1	1149	1149	RING-H2 finger ATL46-like	382	gi|743902005|ref|XP_011044332.1|PREDICTED: RING-H2 finger protein ATL46-like [Populus euphratica]gi|743902007|ref|XP_011044333.1|PREDICTED: RING-H2 finger protein ATL46-like [Populus euphratica]	XP_011044332, XP_011044333	3.63E-161	375	298
Pe43D2.11	1 isoform	-	6	5097	561	Ubiquitin-conjugating enzyme E2 5 isoform 1 [Theobroma cacao]	186	gi|743849887|ref|XP_011028598.1|PREDICTED: ubiquitin-conjugating enzyme E2-23 kDa-like isoform X1 [Populus euphratica]gi|743849891|ref|XP_011028599.1|PREDICTED: ubiquitin-conjugating enzyme E2-23 kDa-like isoform X1 [Populus euphratica]	XP_011028598, XP_011028599	4.87E-114	186	178
Pe43D2.12		-	2	1934	252	RING-H2 finger ATL48	83	gi|566206610|ref|XP_006374514.1|hypothetical protein POPTR_0015s08460g [Populus trichocarpa]gi|743944008|ref|XP_011016516.1|PREDICTED: RING-H2 finger protein ATL48-like [Populus euphratica]gi|550322312|gb|ERP52311.1|hypothetical protein POPTR_0015s08460g [Populus trichocarpa]	XP_006374514, XP_011016516, ERP52311	1.40E-39	83	76
Pe43D2.13		+	7	1887	1074	WAT1-related At3g28050-like	357	gi|743849922|ref|XP_011028603.1|PREDICTED: LOW QUALITY PROTEIN: WAT1-related protein At5g40240-like [Populus euphratica]	XP_011028603	1.02E-164	332	281
Pe43D2.14		+	7	2779	1080	WAT1-related At3g28050-like	389	gi|743849922|ref|XP_011028603.1|PREDICTED: LOW QUALITY PROTEIN: WAT1-related protein At5g40240-like [Populus euphratica]	XP_011028603	9.63E-155	342	279
Pe43D2.15		+	8	3251	636	Rac-like GTP-binding ARAC8	211	gi|743902015|ref|XP_011044337.1|PREDICTED: rac-like GTP-binding protein ARAC8 [Populus euphratica]	XP_011044337	1.93E-138	211	201
Pe43D2.16		-	3	7338	4872	BAH domain,TFIIS helical bundle-like domain isoform 1 [Theobroma cacao]	1623	gi|566206598|ref|XP_002321574.2|hypothetical protein POPTR_0015s08400g [Populus trichocarpa]gi|566206600|ref|XP_002321573.2|hypothetical protein POPTR_0015s08400g [Populus trichocarpa]gi|550322306|gb|EEF05701.2|hypothetical protein POPTR_0015s08400g [Populus trichocarpa]gi|550322307|gb|EEF05700.2|hypothetical protein POPTR_0015s08400g [Populus trichocarpa]	XP_002321574, XP_002321573, EEF05701, EEF05700	0.0	1661	1258
Pe43D2.17	3 isoforms	-	3	3331	438	39S ribosomal mitochondrial-like	145	gi|743790908|ref|XP_011040021.1|PREDICTED: 39S ribosomal protein L47, mitochondrial-like isoform X1 [Populus euphratica]	XP_011040021	2.94E-77	145	133
Pe43D2.18		+	2	447	411	---Na---	136	No Blast Hit				
Pe43D2.19	Incomplete 3'	+	4	1546	470	Nucleobase-ascorbate transporter 6-like [Populus euphratica]	156	gi|566197332|ref|XP_006376839.1|hypothetical protein POPTR_0012s07890g [Populus trichocarpa]gi|550326615|gb|ERP54636.1|hypothetical protein POPTR_0012s07890g [Populus trichocarpa]	XP_006376839, ERP54636	1.21E-95	155	152
Pe43L2.1		+	3	2535	519	Chloroplast-targeted copper chaperone [Theobroma cacao]	172	gi|731410542|ref|XP_010657601.1|PREDICTED: uncharacterized protein LOC100243595 [Vitis vinifera]	XP_010657601	4.97E-61	175	130
Pe43L2.2		+	13	3189	1974	Sulfate transporter -like [Populus euphratica]	657	gi|590688676|ref|XP_007043016.1|Sulfate transporter 1,3 isoform 1 [Theobroma cacao]gi|590688684|ref|XP_007043018.1|Sulfate transporter 1,3 isoform 1 [Theobroma cacao]gi|590688687|ref|XP_007043019.1|Sulfate	XP_007043016, XP_007043018, XP_007043019, EOX98847, EOX98849,	0.0	656	607

								transporter 1,3 isoform 1 [Theobroma cacao]gi|508706951|gb|EOX98847.1|Sulfate transporter 1,3 isoform 1 [Theobroma cacao]gi|508706953|gb|EOX98849.1|Sulfate transporter 1,3 isoform 1 [Theobroma cacao]gi|508706954|gb|EOX98850.1|Sulfate transporter 1,3 isoform 1 [Theobroma cacao]	EOX98850			
Pe43L2.3		+	3	833	543	Zinc finger zfs1-like	180	gi|720016204|ref|XP_010261083.1|PREDICTED: uncharacterized protein LOC104599994 [Nelumbo nucifera]gi|720016208|ref|XP_010261084.1|PREDICTED: uncharacterized protein LOC104599994 [Nelumbo nucifera]	XP_010261083, XP_010261084	4.99E-03	127	56
Pe43L2.4		-	5	1227	774	20 kda chloroplastic-like isoform X1 [Gossypium hirsutum]	257	gi|802628447|ref|XP_012077092.1|PREDICTED: 20 kDa chaperonin, chloroplastic [Jatropha curcas]gi|643724747|gb|KDP33948.1|hypothetical protein JCGZ_07519 [Jatropha curcas]	XP_012077092, KDP33948	1.36E-147	257	240
Pe43L2.5		-	3	769	540	Non-specific lipid-transfer At5g64080	179	gi|118485342|gb|ABK94530.1|unknown [Populus trichocarpa]	ABK94530	3.48E-48	184	135
Pe43L2.6		+	2	879	780	Chlorophyll a-b binding CP24 chloroplastic	259	gi|743922235|ref|XP_011005195.1|PREDICTED: chlorophyll a-b binding protein CP24 10A, chloroplastic-like [Populus euphratica]gi|743941455|ref|XP_011015216.1|PREDICTED: chlorophyll a-b binding protein CP24 10A, chloroplastic-like [Populus euphratica]	XP_011005195, XP_011015216	1.10E-156	259	242
Pe43L2.7		-	4	2077	702	Beta-hydroxyacyl-ACP dehydratase family [Populus trichocarpa]	233	gi|924434384|gb|ALB76802.1|hydroxyacyl-ACP dehydratase [Jatropha curcas]	ALB76802	1.94E-119	244	207
Pe43L2.8	1 isoform	+	1	3679	1362	MTL1-like [Jatropha curcas]	453	gi|1000950698|ref|XP_002526903.2|PREDICTED: uncharacterized protein LOC8270482, partial [Ricinus communis]	XP_002526903	1.12E-164	411	312
Pe43L2.9	1 isoform	-	1	1978	375	Lipid transfer	124	gi|345104193|gb|AEN70918.1|lipid transfer protein [Gossypium turneri]gi|345104219|gb|AEN70931.1|lipid transfer protein [Gossypium armourianum]gi|345104221|gb|AEN70932.1|lipid transfer protein [Gossypium harknessii]	AEN70918, AEN70931, AEN70932	2.89E-33	124	88
Pe43L2.10		+	3	1361	987	Peroxidase N1-like	328	gi|225626273|gb|ACN97186.1|peroxidase [Populus trichocarpa]	ACN97186	0.0	323	284
Pe43L2.11		+	2	2464	1629	Probable inactive receptor kinase At2g26730	542	gi|223530209|gb|EEF32117.1|serine-threonine protein kinase, plant-type, putative [Ricinus communis]	EEF32117	0.0	499	390
Pe43L2.12		+	5	2486	1350	5 -3 exoribonuclease-like [Gossypium hirsutum]	449	gi|802711239|ref|XP_012084609.1|PREDICTED: uncharacterized protein LOC105643976 [Jatropha curcas]gi|643715123|gb|KDP27373.1|hypothetical protein JCGZ_20197 [Jatropha curcas]	XP_012084609, KDP27373	0.0	453	390
Pe43L2.13	6 isoforms	-	7	3237	759	Endoplasmic reticulum-Golgi intermediate compartment 3-like	252	gi|590688586|ref|XP_007042992.1|Endoplasmic reticulum vesicle transporter protein [Theobroma cacao]gi|508706927|gb|EOX98823.1|Endoplasmic reticulum vesicle transporter protein [Theobroma cacao]	XP_007042992, EOX98823	4.63E-146	248	229
Pe43L2.14		+	2	540	354	Lipid transfer precursor [Gossypium hirsutum]	117	gi|763804597|gb|KJB71535.1|hypothetical protein B456_011G128300 [Gossypium raimondii]	KJB71535	4.65E-44	119	99
Pe43L2.15		-	2	1335	357	Lipid transfer	118	gi|823243397|ref|XP_012454350.1|PREDICTED: non-specific lipid-transfer protein-like	XP_012454350, XP_016698971,	2.40E-42	120	97

								[Gossypium raimondii]gi|1029077556|ref|XP_016698971.1|PREDICTED: non-specific lipid-transfer protein-like [Gossypium hirsutum]gi|7012719|gb|AAF35184.1|AF195863_1lipid transfer protein precursor [Gossypium hirsutum]gi|208427039|gb|ACI26701.1|lipid transfer protein [Gossypium hirsutum]gi|403391431|gb|AFR43273.1|lipid transfer protein precursor [Gossypium raimondii]gi|403391433|gb|AFR43274.1|lipid transfer protein precursor [Gossypium hirsutum]gi|403391435|gb|AFR43275.1|lipid transfer protein precursor [Gossypium barbadense]gi|763804574|gb|KJB71512.1|hypothetical protein B456_011G128200 [Gossypium raimondii]	AAF35184, ACI26701, AFR43273, AFR43274, AFR43275, KJB71512			
Pe43L2.16		+	1	339	339	---Na---	112	No Blast Hit				
Pe43L2.17	3 isoforms	-	10	6025	1584	Dentin sialophospho isoform X1 [Cucumis melo]	527	gi|1029121789|ref|XP_016739342.1|PREDICTED: protein LNK1-like isoform X3 [Gossypium hirsutum]	XP_016739342	3.41E-125	565	320
Pe43L2.18		+	4	1370	978	Peroxidase 43	325	gi|1028936978|ref|XP_016711666.1|PREDICTED: peroxidase 43-like [Gossypium hirsutum]	XP_016711666	0.0	317	279
Pe43L2.19		-	5	2898	2199	Pumilio homolog chloroplastic	732	gi|1009137772|ref|XP_015886236.1|PREDICTED: uncharacterized protein LOC107421499 [Ziziphus jujuba]	XP_015886236	0.0	779	519
Pe43L2.20		+	3	5745	483	03g49990d [Brassica napus]	160	gi|698588827|ref|XP_009779573.1|PREDICTED: uncharacterized protein LOC104228746 [Nicotiana sylvestris]gi|1025403315|ref|XP_016437602.1|PREDICTED: uncharacterized protein LOC107763627 isoform X1 [Nicotiana tabacum]	XP_009779573, XP_016437602	3.19E-72	158	140
Pe43L2.21		-	3	3005	2748	Probable LRR receptor-like serine threonine-kinase At4g08850	915	gi|743917775|ref|XP_011002880.1|PREDICTED: probable LRR receptor-like serine/threonine-protein kinase At4g08850 isoform X1 [Populus euphratica]	XP_011002880	0.0	937	705
Pe43L2.22		-	12	2914	1482	Beta-glucosidase 12-like	493	gi|28628597|gb|AAO49267.1|AF480476_1P66 protein [Hevea brasiliensis]	AAO49267	2.21E-175	512	345
Pe43L2.23		-	18	10097	2700	Copper-transporting atpase chloroplastic	899	gi|743921834|ref|XP_011004985.1|PREDICTED: copper-transporting ATPase PAA1, chloroplastic-like [Populus euphratica]	XP_011004985	0.0	932	788
Pe43L2.24		+	6	3405	1041	Pentatricopeptide repeat-containing chloroplastic [Nelumbo nucifera]	346	gi|720059738|ref|XP_010274657.1|PREDICTED: pentatricopeptide repeat-containing protein At4g18975, chloroplastic [Nelumbo nucifera]gi|720059741|ref|XP_010274658.1|PREDICTED: pentatricopeptide repeat-containing protein At4g18975, chloroplastic [Nelumbo nucifera]gi|720059745|ref|XP_010274659.1|PREDICTED: pentatricopeptide repeat-containing protein At4g18975, chloroplastic [Nelumbo nucifera]gi|720059748|ref|XP_010274660.1|PREDICTED: pentatricopeptide repeat-containing protein At4g18975, chloroplastic [Nelumbo nucifera]gi|720059751|ref|XP_010274661.1|PREDICTED: pentatricopeptide repeat-containing protein At4g18975, chloroplastic [Nelumbo nucifera]	XP_010274657, XP_010274658, XP_010274659, XP_010274660, XP_010274661	7.07E-115	287	220

Pe43L2.25		-	4	1487	978	Mitochondrial alternative oxidase 2 [Olea europaea]	325	gi|802710797|ref|XP_012084589.1|PREDICTED: ubiquinol oxidase, mitochondrial [Jatropha curcas]gi|643715102|gb|KDP27352.1|hypothetical protein JCGZ_20176 [Jatropha curcas]	XP_012084589, KDP27352	0.0	352	282
Pe43L2.26		+	13	6948	3123	Calmodulin-binding transcription activator 2-like	1040	gi|802710799|ref|XP_012084590.1|PREDICTED: calmodulin-binding transcription activator 2 [Jatropha curcas]gi|643715103|gb|KDP27353.1|hypothetical protein JCGZ_20177 [Jatropha curcas]	XP_012084590, KDP27353	0.0	1099	797
Pe43L2.27		-	4	575	279	Tobamovirus multiplication 1-like [Elaeis guineensis]	92	gi|590688466|ref|XP_007042956.1|Tobamovirus multiplication 1 isoform 1 [Theobroma cacao]gi|508706891|gb|EOX98787.1|Tobamovirus multiplication 1 isoform 1 [Theobroma cacao]	XP_007042956, EOX98787	7.18E-48	92	88
Pe51C2.1	Incomplete 3'	-	1	1407	1407	RING U-box superfamily isoform 1 [Theobroma cacao]	469	gi|802578551|ref|XP_012069461.1|PREDICTED: putative E3 ubiquitin-protein ligase RF298 [Jatropha curcas]gi|802578553|ref|XP_012069462.1|PREDICTED: putative E3 ubiquitin-protein ligase RF298 [Jatropha curcas]gi|643733115|gb|KDP40062.1|hypothetical protein JCGZ_02060 [Jatropha curcas]	XP_012069461, XP_012069462, KDP40062	0.0	467	371
Pe51C2.2		-	1	1888	1173	DOS2-like isoform X2	390	gi|802578680|ref|XP_012069477.1|PREDICTED: BSD domain-containing protein 1 [Jatropha curcas]gi|643733125|gb|KDP40072.1|hypothetical protein JCGZ_02070 [Jatropha curcas]	XP_012069477, KDP40072	9.69E-102	428	272
Pe51C2.3	1 isoform	+	8	6479	1821	F-box LRR-repeat 4	606	gi|802578670|ref|XP_012069471.1|PREDICTED: F-box/LRR-repeat protein 4 isoform X2 [Jatropha curcas]	XP_012069471	0.0	609	550
Pe51C2.4		+	1	1527	1038	S-adenosylmethionine decarboxylase proenzyme 4-like	387	gi|802578666|ref|XP_012069469.1|PREDICTED: S-adenosylmethionine decarboxylase proenzyme 4 [Jatropha curcas]gi|643733120|gb|KDP40067.1|hypothetical protein JCGZ_02065 [Jatropha curcas]	XP_012069469, KDP40067	0.0	345	317
Pe51C2.5	1 isoform	+	2	4538	1059	Probable phosphatase 2C 35 isoform X1 [Vitis vinifera]	352	gi|743933023|ref|XP_011010816.1|PREDICTED: probable protein phosphatase 2C 35 [Populus euphratica]	XP_011010816	0.0	352	320
Pe51C2.6		-	16	8889	5088	SAC3 family B-like isoform X2	1695	gi|1028939311|ref|XP_016712855.1|PREDICTED: SAC3 family protein B-like isoform X2 [Gossypium hirsutum]	XP_016712855	0.0	1738	1096
Pe51C2.7		+	2	3991	2364	FAR1-RELATED SEQUENCE 7-like isoform X1 [Tarenaya hassleriana]	787	gi|802578870|ref|XP_012069480.1|PREDICTED: protein FAR1-RELATED SEQUENCE 7-like isoform X1 [Jatropha curcas]	XP_012069480	0.0	789	647
Pe51C2.8		-	2	2514	2178	Pentatricopeptide repeat-containing At5g18950	725	gi|590660268|ref|XP_007035355.1|Tetratricopeptide repeat-like superfamily protein, putative isoform 1 [Theobroma cacao]gi|508714384|gb|EOY06281.1|Tetratricopeptide repeat-like superfamily protein, putative isoform 1 [Theobroma cacao]	XP_007035355, EOY06281	0.0	577	394
Pe51C2.9		+	1	357	357	F-box kelch-repeat At3g06240-like	118	gi|590660247|ref|XP_007035349.1|F-box family protein, putative [Theobroma cacao]gi|508714378|gb|EOY06275.1|F-box family protein, putative [Theobroma cacao]	XP_007035349, EOY06275	6.80E-26	93	67
Pe51C2.10	2 isoforms	-	7	5212	1143	Chloroplastic	380	gi|224102575|ref|XP_002312731.1|hypothetical protein POPTR_0008s20440g [Populus trichocarpa]gi|566185043|ref|XP_006380046.1|hypothetical protein POPTR_0008s20440g [Populus	XP_002312731, XP_006380046, EEE90098, ERP57843	0.0	382	330

								trichocarpa]gi|222852551|gb|EEE90098.1|hypothetical protein POPTR_0008s20440g [Populus trichocarpa]gi|550333533|gb|ERP57843.1|hypothetical protein POPTR_0008s20440g [Populus trichocarpa]				
Pe51C2.11	1 isoform	-	4	1195	483	AWPM-19 [Arabidopsis thaliana]	160	gi|255551513|ref|XP_002516802.1|PREDICTED: uncharacterized protein LOC8261491 [Ricinus communis]gi|223543890|gb|EEF45416.1|conserved hypothetical protein [Ricinus communis]	XP_002516802, EEF45416	3.87E-78	157	138
Pe51C2.12	4 isoforms	-	13	2834	1170	Glutamine synthetase leaf chloroplastic [Sesamum indicum]	389	gi|641856092|gb|KDO74872.1|hypothetical protein CISIN_1g013478mg [Citrus sinensis]gi|641856093|gb|KDO74873.1|hypothetical protein CISIN_1g013478mg [Citrus sinensis]gi|641856094|gb|KDO74874.1|hypothetical protein CISIN_1g013478mg [Citrus sinensis]	KDO74872, KDO74873, KDO74874	0.0	432	361
Pe51C2.13	3 isoforms	+	14	4755	1737	Alpha beta-Hydrolases superfamily [Theobroma cacao]	578	gi|743940091|ref|XP_011014506.1|PREDICTED: uncharacterized protein LOC105118291 [Populus euphratica]	XP_011014506	0.0	588	501
Pe51C2.14		-	2	3422	2484	ARM repeat superfamily [Theobroma cacao]	827	gi|224107257|ref|XP_002314424.1|hypothetical protein POPTR_0010s03030g [Populus trichocarpa]gi|222863464|gb|EEF00595.1|hypothetical protein POPTR_0010s03030g [Populus trichocarpa]	XP_002314424, EEF00595	0.0	827	741
Pe51C2.15		-	1	2970	864	Guanylate kinase chloroplastic [Populus euphratica]	287	gi|566188905|ref|XP_002314423.2|hypothetical protein POPTR_0010s03040g [Populus trichocarpa]gi|550328988|gb|EEF00594.2|hypothetical protein POPTR_0010s03040g [Populus trichocarpa]	XP_002314423, EEF00594	7.67E-136	298	240
Pe51C2.16		+	4	3886	1209	BTB POZ and MATH domain-containing 2-like	402	gi|802579175|ref|XP_012069503.1|PREDICTED: BTB/POZ and MATH domain-containing protein 2-like [Jatropha curcas]gi|643733140|gb|KDP40087.1|hypothetical protein JCGZ_02085 [Jatropha curcas]	XP_012069503, KDP40087	0.0	383	350
Pe51C2.17		-	10	7222	1683	Mitogen-activated kinase [Populus trichocarpa]	560	gi|802579504|ref|XP_012069505.1|PREDICTED: mitogen-activated protein kinase 15 isoform X2 [Jatropha curcas]	XP_012069505	0.0	560	533
Pe51C2.18		-	3	1244	387	Endo-1,3 1,4-beta-D-glucanase-like	128	gi|629094887|gb|KCW60882.1|hypothetical protein EUGRSUZ_H03614 [Eucalyptus grandis]	KCW60882	4.69E-46	103	87
Pe51C2.19		-	6	4131	1239	Serine incorporator 3	412	gi|747086599|ref|XP_011090806.1|PREDICTED: probable serine incorporator [Sesamum indicum]	XP_011090806	0.0	413	379
Pe60G10.1	Incomplete 3'	-	2	397	261	Integral membrane HRF1 family [Theobroma cacao]	87	gi|697161980|ref|XP_009589789.1|PREDICTED: protein YIF1B-like isoform X2 [Nicotiana tomentosiformis]	XP_009589789	4.45E-18	129	69
Pe60G10.2		+	7	2386	909	PREDICTED: uncharacterized protein LOC105134404 isoform X1	305	gi|743883748|ref|XP_011037106.1|PREDICTED: uncharacterized protein LOC105134404 isoform X1 [Populus euphratica]	XP_011037106	2.72E-148	300	248
Pe60G10.3		-	4	2039	771	Probable fructokinase-4	256	gi|697191881|ref|XP_009605011.1|PREDICTED: fructokinase-2 [Nicotiana tomentosiformis]gi|1025296110|ref|XP_016498734.1|PREDICTED: fructokinase-2-like [Nicotiana tabacum]	XP_009605011, XP_016498734	9.71E-163	255	241
Pe60G10.4	6 isoforms	-	4	3328	1251	Uncharacterized CRM domain-containing chloroplastic	416	gi|802554972|ref|XP_012065185.1|PREDICTED: uncharacterized CRM domain-containing protein At3g25440, chloroplastic [Jatropha curcas]	XP_012065185	0.0	362	300

Pe60G10.5	1 isoform	+	2	1972	717	FAR1-RELATED SEQUENCE 12-like isoform X1 [Gossypium hirsutum]	251	gi|802613810|ref|XP_012074747.1|PREDICTED: protein FAR1-RELATED SEQUENCE 5 isoform X1 [Jatropha curcas]gi|802613812|ref|XP_012074748.1|PREDICTED: protein FAR1-RELATED SEQUENCE 5 isoform X1 [Jatropha curcas]gi|643727214|gb|KDP35748.1|hypothetical protein JCGZ_10520 [Jatropha curcas]	XP_012074747, XP_012074748, KDP35748	1.58E-143	251	225
Pe60G10.6		+	1	1155	564	Actin-like atpase superfamily isoform 1 [Theobroma cacao]	187	gi|743883731|ref|XP_011037101.1|PREDICTED: probable tRNA N6-adenosine threonylcarbamoyltransferase [Populus euphratica]gi|743883735|ref|XP_011037102.1|PREDICTED: probable tRNA N6-adenosine threonylcarbamoyltransferase [Populus euphratica]	XP_011037101, XP_011037102	3.00E-125	186	185
Pe60G10.7		-	2	1407	582	FAR1-RELATED SEQUENCE 5-like [Pyrus x bretschneideri]	193	gi|802554968|ref|XP_012065183.1|PREDICTED: protein FAR1-RELATED SEQUENCE 5 [Jatropha curcas]	XP_012065183	2.11E-117	192	181
Pe60G10.8	3 isoforms	+	3	3405	729	FAR1-RELATED SEQUENCE 5-like isoform X1 [Citrus sinensis]	242	gi|566179137|ref|XP_006380293.1|hypothetical protein POPTR_0007s01930g [Populus trichocarpa]gi|550333930|gb|ERP58090.1|hypothetical protein POPTR_0007s01930g [Populus trichocarpa]	XP_006380293, ERP58090	2.76E-116	233	198
Pe60G10.9		-	1	465	465	Calmodulin 3	154	gi|1009139197|ref|XP_015886996.1|PREDICTED: calmodulin-like protein 3 [Ziziphus jujuba]	XP_015886996	1.48E-79	149	139
Pe60G10.10		-	1	351	351	Glycerophosphodiester phosphodiesterase kinase domain-containing GDPDL2-like	116	gi|1000978964|ref|XP_015571341.1|PREDICTED: probable receptor-like protein kinase At5g39020 [Ricinus communis]	XP_015571341	2.69E-31	95	74
Pe60G10.11		+	5	2224	1302	Probable receptor kinase At1g67000	433	gi|743883524|ref|XP_011037045.1|PREDICTED: probable receptor-like protein kinase At5g39020 [Populus euphratica]	XP_011037045	1.27E-128	411	279
Pe60G10.12		+	4	2988	1995	Probable receptor kinase At1g67000	664	gi|743883524|ref|XP_011037045.1|PREDICTED: probable receptor-like protein kinase At5g39020 [Populus euphratica]	XP_011037045	0.0	679	514
Pe60G10.13		-	1	851	600	Probable receptor kinase At5g39020	199	gi|743883501|ref|XP_011037041.1|PREDICTED: glycerophosphodiester phosphodiesterase protein kinase domain-containing GDPDL2-like isoform X1 [Populus euphratica]	XP_011037041	9.77E-35	176	115
Pe60G10.14		-	1	1008	975	Hypothetical protein	324	gi|332322201|emb|CCA66222.1|hypothetical protein [Beta vulgaris subsp. vulgaris]	CCA66222	1.22E-36	293	148
Pe60G10.15		-	3	2752	2487	Ribonuclease H At1g65750 family	828	gi|1012351323|gb|KYP62512.1|Putative ribonuclease H protein At1g65750 family [Cajanus cajan]	KYP62512	4.85E-27	222	116
Pe60G10.16		+	2	1897	1842	Gag protease poly [Theobroma cacao]	613	gi|590728434|ref|XP_007099662.1|Gag protease polyprotein-like protein [Theobroma cacao]gi|508728474|gb|EOY20371.1|Gag protease polyprotein-like protein [Theobroma cacao]	XP_007099662, EOY20371	6.93E-75	460	240
Pe60G10.17		+	3	2678	2157	Probable receptor kinase At5g39020	718	gi|743883524|ref|XP_011037045.1|PREDICTED: probable receptor-like protein kinase At5g39020 [Populus euphratica]	XP_011037045	0.0	703	507
Pe60G10.18	5 isoforms	+	9	4891	1737	PREDICTED: uncharacterized protein LOC105628378 isoform X2	578	gi|802554918|ref|XP_012065159.1|PREDICTED: uncharacterized protein LOC105628378 isoform X2 [Jatropha curcas]	XP_012065159	1.79E-139	570	347
Pe60G10.19		+	1	4145	2757	Serine threonine- kinase ACR4	918	gi|255585507|ref|XP_002533445.1|PREDICTED: serine/threonine-protein kinase-like protein	XP_002533445, EEF28941	0.0	921	833

Pe60G10.20		-	24	9925	3378	Probable serine threonine-kinase GCN2 [Populus euphratica]	1125	ACR4 [Ricinus communis]gi|223526707|gb|EEF28941.1|receptor protein kinase, putative [Ricinus communis] gi|743937058|ref|XP_011012926.1|PREDICTED: probable serine/threonine-protein kinase GCN2 [Populus euphratica]gi|743937060|ref|XP_011012927.1|PREDICTED: probable serine/threonine-protein kinase GCN2 [Populus euphratica]gi|743937062|ref|XP_011012928.1|PREDICTED: probable serine/threonine-protein kinase GCN2 [Populus euphratica]	XP_011012926, XP_011012927, XP_011012928	0.0	1112	968
Pe61E2.1		+	1	1323	390	---Na---	129	No Blast Hit				
Pe61E2.2		-	4	1584	1323	Pectate lyase-like	440	gi|566184026|ref|XP_002311599.2|pectate lyase family protein [Populus trichocarpa]gi|550333093|gb|EEE88966.2|pectate lyase family protein [Populus trichocarpa]	XP_002311599, EEE88966	0.0	433	361
Pe61E2.3		+	6	3116	831	Mitochondrial outer membrane porin of 36 kda	276	gi|224102515|ref|XP_002312708.1|porin family protein [Populus trichocarpa]gi|118484777|gb|ABK94257.1|unknown [Populus trichocarpa]gi|222852528|gb|EEE90075.1|porin family protein [Populus trichocarpa]	XP_002312708, ABK94257, EEE90075	1.17E-168	276	258
Pe61E2.4		-	5	3572	858	Hypersensitive-induced response 2 [Gossypium raimondii]	285	gi|802787478|ref|XP_012091938.1|PREDICTED: hypersensitive-induced response protein 2 [Jatropha curcas]gi|802787482|ref|XP_012091939.1|PREDICTED: hypersensitive-induced response protein 2 [Jatropha curcas]gi|643704162|gb|KDP21226.1|hypothetical protein JCGZ_21697 [Jatropha curcas]	XP_012091938, XP_012091939, KDP21226	0.0	282	277
Pe61E2.5	1 isoform	+	5	6950	666	Mediator of RNA polymerase II transcription subunit 19a-like	221	gi|731335988|ref|XP_010679015.1|PREDICTED: mediator of RNA polymerase II transcription subunit 19a-like isoform X1 [Beta vulgaris subsp. vulgaris]gi|731335990|ref|XP_010679016.1|PREDICTED: mediator of RNA polymerase II transcription subunit 19a-like isoform X1 [Beta vulgaris subsp. vulgaris]gi|731335992|ref|XP_010679017.1|PREDICTED: mediator of RNA polymerase II transcription subunit 19a-like isoform X1 [Beta vulgaris subsp. vulgaris]gi|731335994|ref|XP_010679018.1|PREDICTED: mediator of RNA polymerase II transcription subunit 19a-like isoform X1 [Beta vulgaris subsp. vulgaris]	XP_010679015, XP_010679016, XP_010679017, XP_010679018	5.29E-92	240	205
Pe61E2.6		-	6	3719	1449	Probable receptor kinase At5g15080	482	gi|255558222|ref|XP_002520138.1|PREDICTED: probable receptor-like protein kinase At5g15080 [Ricinus communis]gi|223540630|gb|EEF42193.1|Protein kinase APK1B, chloroplast precursor, putative [Ricinus communis]	XP_002520138, EEF42193	0.0	499	447
Pe61E2.7	1 isoform	-	7	8598	2103	PREDICTED: uncharacterized protein LOC8287018 isoform X1 [Ricinus communis]	700	gi|255558224|ref|XP_002520139.1|PREDICTED: uncharacterized protein LOC8287018 isoform X1 [Ricinus communis]gi|1000964234|ref|XP_015575275.1|P	XP_002520139, XP_015575275, EEF42194	0.0	697	544

Pe61E2.8		-	6	3756	1248	KINASE 2B family [Populus trichocarpa]	415	REDICTED: uncharacterized protein LOC8287018 isoform X1 [Ricinus communis]gi|223540631|gb|EEF42194.1|conserved hypothetical protein [Ricinus communis] gi|255558180|ref|XP_002520117.1|PREDICTED: protein kinase 2B, chloroplastic [Ricinus communis]gi|223540609|gb|EEF42172.1|Protein kinase APK1A, chloroplast precursor, putative [Ricinus communis]	XP_002520117, EEF42172	0.0	419	381
Pe61E2.9		+	7	3952	996	UDP-galactose UDP-glucose transporter 3	331	gi|590700510|ref|XP_007046181.1|UDP-galactose transporter 3 isoform 1 [Theobroma cacao]gi|508710116|gb|EOY02013.1|UDP-galactose transporter 3 isoform 1 [Theobroma cacao]	XP_007046181, EOY02013	0.0	329	317
Pe61E2.10		-	12	3591	1440	Transcription factor MYB51-like isoform X1 [Ziziphus jujuba]	479	gi|802566305|ref|XP_012067595.1|PREDICTED: uncharacterized protein LOC105630404 isoform X1 [Jatropha curcas]gi|643734494|gb|KDP41164.1|hypothetical protein JCGZ_15571 [Jatropha curcas]gi|696739792|gb|AIT52216.1|MYB family protein [Jatropha curcas]	XP_012067595, KDP41164, AIT52216	0.0	479	392
Pe61E2.11		-	3	1195	606	ABC transporter B family member 6	201	gi|567186276|ref|XP_006403472.1|hypothetical protein EUTSA_v10010064mg [Eutrema salsugineum]gi|557104591|gb|ESQ44925.1|hypothetical protein EUTSA_v10010064mg [Eutrema salsugineum]	XP_006403472, ESQ44925	1.07E-38	200	108
Pe61E2.12		+	1	869	342	---Na---	113	No Blast Hit				
Pe61E2.13	2 isoforms	-	3	2762	831	Homeobox-leucine zipper ATHB-13-like	281	gi|802566327|ref|XP_012067598.1|PREDICTED: homeobox-leucine zipper protein ATHB-13 [Jatropha curcas]	XP_012067598	5.22E-159	288	253
Pe61E2.14		-	1	231	231	---Na---	76	No Blast Hit				
Pe61E2.15		-	1	1287	1287	F-box kelch-repeat SKIP11 [Malus domestica]	428	gi|255587925|ref|XP_002534442.1|PREDICTED: F-box/kelch-repeat protein SKIP11 [Ricinus communis]gi|223525283|gb|EEF27941.1|conserved hypothetical protein [Ricinus communis]	XP_002534442, EEF27941	0.0	427	366
Pe63J18.1		-	1	6941	1104	Hypothetical protein CISIN_1g046866mg, partial	367	gi|641851407|gb|KDO70278.1|hypothetical protein CISIN_1g046866mg, partial [Citrus sinensis]	KDO70278	1.32E-52	263	163
Pe63J18.2		-	1	1172	312	---Na---	103	No Blast Hit				
Pe63J18.3		-	1	2575	1086	Ribonuclease H At1g65750	361	gi|1021535283|ref|XP_016164673.1|PREDICTED: uncharacterized protein LOC107607211 [Arachis ipaensis]	XP_016164673	3.65E-64	342	190
Pe63J18.4		-	1	3864	768	PREDICTED: uncharacterized protein LOC107461000	255	gi|1012217746|ref|XP_015934914.1|PREDICTED: uncharacterized protein LOC107461000 [Arachis duranensis]	XP_015934914	9.36E-34	180	104
Pe63J18.5		+	1	2068	213	---Na---	70	No Blast Hit				
Pe63J18.6		-	1	3381	798	Chlorophyll a-b binding 2 [Populus trichocarpa]	265	gi|224114357|ref|XP_002316737.1|Chlorophyll a-b binding protein 2 [Populus trichocarpa]gi|222859802|gb|EEE97349.1|Chlorophyll a-b binding protein 2 [Populus trichocarpa]	XP_002316737, EEE97349	1.05E-171	265	251
Pe63J18.7		+	1	1427	798	Chlorophyll a-b binding 2 [Populus trichocarpa]	265	gi|224114357|ref|XP_002316737.1|Chlorophyll a-b binding protein 2 [Populus trichocarpa]gi|222859802|gb|EEE97349.1|Chlorophyll a-b binding protein 2 [Populus trichocarpa]	XP_002316737, EEE97349	7.05E-172	265	252
Pe63J18.8		-	2	3435	594	Ribonuclease H	197	gi|659121154|ref|XP_008460525.1|PREDICTED	XP_008460525	1.15E-11	188	87

						At1g65750		: LOW QUALITY PROTEIN: putative ribonuclease H protein At1g65750 [Cucumis melo]				
Pe63J18.9		-	5	4140	612	---Na---	203	No Blast Hit				
Pe63J18.10		-	2	2364	618	PREDICTED: uncharacterized protein LOC104108390	205	gi|697123427|ref|XP_009615707.1|PREDICTED : uncharacterized protein LOC104108390 [Nicotiana tomentosiformis]	XP_009615707	1.90E-07	127	63
Pe63J18.11		-	1	441	717	Gag-Pol poly	238	gi|1025093233|ref|XP_016457574.1|PREDICTE D: uncharacterized mitochondrial protein AtMg00300-like [Nicotiana tabacum]	XP_016457574	4.09E-32	176	108
Pe63J18.12		-	1	3327	1530	Retrotransposable element Tf2	509	gi|950956371|ref|XP_014496836.1|PREDICTED : uncharacterized protein LOC106758422 [Vigna radiata var. radiata]	XP_014496836	1.97E-71	275	186
Pe63J18.13		-	1	3995	1149	Transposon Ty3-I Gag-Pol poly	382	gi|950927111|ref|XP_014491240.1|PREDICTED : uncharacterized protein LOC106753853 [Vigna radiata var. radiata]	XP_014491240	2.15E-34	234	123
Pe63J18.14	4 isoforms	+	2	1665	702	PREDICTED: uncharacterized protein LOC105633969 [Jatropha curcas]	233	gi|802596133|ref|XP_012072077.1|PREDICTED : uncharacterized protein LOC105633969 [Jatropha curcas]gi|643730511|gb|KDP37943.1|hypothetica l protein JCGZ_04586 [Jatropha curcas]	XP_012072077, KDP37943	1.31E-64	247	171
Pe63J18.15		-	2	906	3429	LRR receptor-like serine threonine- kinase FLS2	1142	gi|566165232|ref|XP_002305701.2|FLAGELLIN -SENSITIVE 2 family protein [Populus trichocarpa]gi|550340449|gb|EEE86212.2|FLAG ELLIN-SENSITIVE 2 family protein [Populus trichocarpa]	XP_002305701, EEE86212	0.0	1142	948
Pe63J18.16-18		-	4	2307	1190	Tetratricopeptide TPR-1	130	gi|802702008|ref|XP_012083989.1|PREDICTED : induced during hyphae development protein 1 [Jatropha curcas]gi|643716070|gb|KDP27843.1|hypothetica l protein JCGZ_18923 [Jatropha curcas]	XP_012083989, KDP27843	3.08E-43	127	95
Pe65F7.1	Incomplete 5'	+	1	450	450	Hypothetical protein PRUPE_ppa017845mg [Prunus persica]	149	gi|596296979|ref|XP_007227259.1|hypothetical protein PRUPE_ppa017845mg [Prunus persica]gi|462424195|gb|EMJ28458.1|hypothetic al protein PRUPE_ppa017845mg [Prunus persica]	XP_007227259, EMJ28458	3.11E-76	147	135
Pe65F7.2		-	3	883	648	PREDICTED: uncharacterized protein LOC105637663 [Jatropha curcas]	215	gi|802627228|ref|XP_012076604.1|PREDICTED : uncharacterized protein LOC105637663 [Jatropha curcas]gi|643724415|gb|KDP33616.1|hypothetica l protein JCGZ_07187 [Jatropha curcas]	XP_012076604, KDP33616	6.51E-124	209	199
Pe65F7.3		+	1	306	306	Overexpressor of cationic peroxidase [Theobroma cacao]	101	gi|223545482|gb|EEF46987.1|conserved hypothetical protein [Ricinus communis]	EEF46987	2.15E-22	102	69
Pe65F7.4	1 isoform	+	4	2459	510	Bzip with a Ring-finger motif family [Populus trichocarpa]	241	gi|224089199|ref|XP_002308656.1|bZIP with a Ring-finger motif family protein [Populus trichocarpa]gi|222854632|gb|EEE92179.1|bZIP with a Ring-finger motif family protein [Populus trichocarpa]	XP_002308656, EEE92179	1.08E-73	169	148
Pe65F7.5		+	14	7081	3252	Cellulose synthase A catalytic subunit 1 [UDP-forming]	1083	gi|802627222|ref|XP_012076601.1|PREDICTED : cellulose synthase A catalytic subunit 1 [UDP-forming] [Jatropha curcas]gi|643724411|gb|KDP33612.1|hypothetica l protein JCGZ_07183 [Jatropha curcas]	XP_012076601, KDP33612	0.0	1084	1058
Pe65F7.6		-	3	2713	1149	Adenine nucleotide transporter chloroplastic mitochondrial-like	382	gi|255548956|ref|XP_002515534.1|PREDICTED : adenine nucleotide transporter BT1, chloroplastic/mitochondrial [Ricinus	XP_002515534, EEF46983	0.0	382	345

								communis]gi|223545478|gb|EEF46983.1|ADP,ATP carrier protein, putative [Ricinus communis]				
Pe65F7.7		-	1	2068	1047	Probable sugar phosphate phosphate translocator At5g25400	348	gi|802627198|ref|XP_012076592.1|PREDICTED: probable sugar phosphate/phosphate translocator At5g25400 isoform X1 [Jatropha curcas]	XP_012076592	0.0	348	335
Pe65F7.8		-	11	4883	1281	DEAD-box ATP-dependent RNA helicase 56-like isoform X1 [Gossypium raimondii]	426	gi|823237042|ref|XP_012451180.1|PREDICTED: DEAD-box ATP-dependent RNA helicase 56-like isoform X1 [Gossypium raimondii]gi|823237044|ref|XP_012451181.1|PREDICTED: DEAD-box ATP-dependent RNA helicase 56-like isoform X1 [Gossypium raimondii]gi|763798856|gb|KJB65811.1|hypothetical protein B456_010G114300 [Gossypium raimondii]	XP_012451180, XP_012451181, KJB65811	0.0	428	425
Pe65F7.9		-	3	1206	1020	SPX domain-containing 1-like	339	gi|802627171|ref|XP_012076581.1|PREDICTED: SPX domain-containing protein 1-like [Jatropha curcas]gi|802627174|ref|XP_012076582.1|PREDICTED: SPX domain-containing protein 1-like [Jatropha curcas]gi|643724399|gb|KDP33600.1|hypothetical protein JCGZ_07171 [Jatropha curcas]	XP_012076581, XP_012076582, KDP33600	7.47E-121	281	225
Pe65F7.10		-	5	963	444	Coatomer subunit zeta-1	147	gi|659132839|ref|XP_008466411.1|PREDICTED: coatomer subunit zeta-1 [Cucumis melo]	XP_008466411	5.25E-77	146	131
Pe65F7.11	1 isoform	-	2	1765	564	Ethylene-responsive transcription factor SHINE 2-like	187	gi|449437136|ref|XP_004136348.1|PREDICTED: ethylene-responsive transcription factor SHINE 2 [Cucumis sativus]gi|778686968|ref|XP_011652482.1|PREDICTED: ethylene-responsive transcription factor SHINE 2 [Cucumis sativus]gi|700204968|gb|KGN60101.1|hypothetical protein Csa_3G878210 [Cucumis sativus]	XP_004136348, XP_011652482, KGN60101	7.51E-82	187	149
Pe65F7.12		-	1	492	492	PREDICTED: uncharacterized protein LOC107832345, partial	163	gi|1025007312|ref|XP_016515689.1|PREDICTED: uncharacterized protein LOC107832345, partial [Nicotiana tabacum]	XP_016515689	2.06E+00	38	31
Pe65F7.13		-	1	620	234	---Na---	77	No Blast Hit				
Pe65F7.14		+	1	1188	306	---Na---	101	No Blast Hit				
Pe65F7.15		+	1	671	213	---Na---	70	No Blast Hit				
Pe65F7.16		-	1	339	339	---Na---	112	No Blast Hit				
Pe65F7.17		+	6	2511	582	PREDICTED: uncharacterized protein LOC8259455 isoform X3	193	gi|1000974478|ref|XP_015572513.1|PREDICTED: uncharacterized protein LOC8259455 isoform X3 [Ricinus communis]	XP_015572513	6.52E-65	158	125
Pe65F7.18		+	2	3858	1938	EIN3-binding F-box 1-like	645	gi|566178356|ref|XP_002308665.2|grr1 family protein [Populus trichocarpa]gi|550337168|gb|EEE92188.2|grr1 family protein [Populus trichocarpa]	XP_002308665, EEE92188	0.0	646	554
Pe65F7.19		+	3	998	804	mRNA maturation factor chloroplastic	267	gi|566178359|ref|XP_002308666.2|hypothetical protein POPTR_0006s27040g [Populus trichocarpa]gi|550337169|gb|EEE92189.2|hypothetical protein POPTR_0006s27040g [Populus trichocarpa]	XP_002308666, EEE92189	9.80E-48	278	162
Pe65F7.20		-	8	4007	1317	Ankyrin repeat domain-containing 12 [Populus euphratica]	438	gi|802627125|ref|XP_012076560.1|PREDICTED: serine/threonine-protein kinase PRP4 homolog [Jatropha curcas]gi|643724391|gb|KDP33592.1|hypothetica	XP_012076560, KDP33592	1.80E-134	464	309

								l protein JCGZ_07163 [Jatropha curcas]				
Pe69C7.1	Incomplete 5'	+	2	1591	264	Calmodulin-7-like isoform X1 [Erythranthe guttata]	87	gi|848875962|ref|XP_012838448.1|PREDICTED: calmodulin-7-like isoform X1 [Erythranthe guttata]	XP_012838448	1.98E-16	79	57
Pe69C7.2		-	3	3347	792	ABA-responsive element binding 3 isoform 1 [Theobroma cacao]	268	gi|743919650|ref|XP_011003851.1|PREDICTED: ABSCISIC ACID-INSENSITIVE 5-like protein 2 isoform X1 [Populus euphratica]gi|743919652|ref|XP_011003852.1|PREDICTED: ABSCISIC ACID-INSENSITIVE 5-like protein 2 isoform X1 [Populus euphratica]gi|743919654|ref|XP_011003853.1|PREDICTED: ABSCISIC ACID-INSENSITIVE 5-like protein 2 isoform X2 [Populus euphratica]	XP_011003851, XP_011003852, XP_011003853	6.39E-130	276	223
Pe69C7.3	1 isoform	+	7	4986	732	Probable ATP synthase 24 kda mitochondrial	243	gi|702450474|ref|XP_010025519.1|PREDICTED: probable ATP synthase 24 kDa subunit, mitochondrial [Eucalyptus grandis]gi|629096224|gb|KCW62219.1|hypothetical protein EUGRSUZ_H04880 [Eucalyptus grandis]	XP_010025519, KCW62219	1.34E-118	243	216
Pe69C7.4		-	7	5276	1758	Pentatricopeptide repeat-containing At3g59040	585	gi|743923882|ref|XP_011006049.1|PREDICTED: pentatricopeptide repeat-containing protein At3g59040 [Populus euphratica]	XP_011006049	0.0	599	498
Pe69C7.5	2 isoforms	+	1	4526	1440	UBP1-associated 2B-like [Gossypium hirsutum]	479	gi|1028991299|ref|XP_016750489.1|PREDICTED: UBP1-associated protein 2B-like [Gossypium hirsutum]gi|1028991302|ref|XP_016750490.1|PREDICTED: UBP1-associated protein 2B-like [Gossypium hirsutum]gi|1028991305|ref|XP_016750491.1|PREDICTED: UBP1-associated protein 2B-like [Gossypium hirsutum]	XP_016750489, XP_016750490, XP_016750491	2.10E-163	399	314
Pe69C7.6		+	16	6151	1353	DNA primase small subunit	450	gi|590679147|ref|XP_007040498.1|DNA primase isoform 1 [Theobroma cacao]gi|508777743|gb|EOY24999.1|DNA primase isoform 1 [Theobroma cacao]	XP_007040498, EOY24999	0.0	453	403
Pe69C7.7	1 isoform	+	15	8505	2712	La-related 1A	903	gi|566185915|ref|XP_002313496.2|hypothetical protein POPTR_0009s02330g [Populus trichocarpa]gi|550330877|gb|EEE87451.2|hypothetical protein POPTR_0009s02330g [Populus trichocarpa]	XP_002313496, EEE87451	0.0	935	708
Pe69C7.8	1 isoform	-	22	7337	2754	Argonaute 4-like [Pyrus x bretschneideri]	917	gi|743923892|ref|XP_011006053.1|PREDICTED: protein argonaute 4-like [Populus euphratica]gi|743923894|ref|XP_011006054.1|PREDICTED: protein argonaute 4-like [Populus euphratica]	XP_011006053, XP_011006054	0.0	913	853
Pe69C7.9		+	1	210	210	---Na---	69	No Blast Hit				
Pe69C7.10		+	4	2971	804	Probable calcium-binding CML49	267	gi|802730522|ref|XP_012086224.1|PREDICTED: probable calcium-binding protein CML49 [Jatropha curcas]gi|643713106|gb|KDP26092.1|hypothetical protein JCGZ_21125 [Jatropha curcas]	XP_012086224, KDP26092	3.19E-143	277	238
Pe69C7.11		-	1	903	903	Chitinase 2-like	300	gi|802600562|ref|XP_012073007.1|PREDICTED: chitinase 2-like [Jatropha curcas]gi|643729505|gb|KDP37337.1|hypothetical protein JCGZ_06791 [Jatropha curcas]	XP_012073007, KDP37337	6.46E-161	292	249
Pe69C7.12		+	1	261	261	---Na---	86	No Blast Hit				

Pe69C7.13		+	1	381	381	---Na---	126	No Blast Hit				
Pe69C7.14		+	9	4641	2712	Linoleate 13S-lipoxygenase 2-chloroplastic-like	903	gi|71999169|gb|AAZ57444.1|lipoxygenase LOX1 [Populus deltoides]	AAZ57444	0.0	901	675
Pe69C7.15		-	14	4044	2637	Respiratory burst oxidase homolog H	878	gi|224087712|ref|XP_002308210.1|ferric reductase-like transmembrane component family protein [Populus trichocarpa]gi|222854186|gb|EEE91733.1|ferric reductase-like transmembrane component family protein [Populus trichocarpa]	XP_002308210, EEE91733	0.0	846	713
Pe69C7.16	1 isoform	+	1	6791	4164	Isoform 1 [Theobroma cacao]	1387	gi|802552025|ref|XP_012064934.1|PREDICTED: uncharacterized protein LOC105628177 [Jatropha curcas]gi|802552027|ref|XP_012064935.1|PREDICTED: uncharacterized protein LOC105628177 [Jatropha curcas]gi|643738167|gb|KDP44155.1|hypothetical protein JCGZ_05622 [Jatropha curcas]	XP_012064934, XP_012064935, KDP44155	0.0	1402	1131
Pe69C7.17		-	1	2205	1395	UDP-glycosyltransferase 73C1-like	484	gi|224121206|ref|XP_002318525.1|UTP-glucose glucosyltransferase family protein [Populus trichocarpa]gi|222859198|gb|EEE96745.1|UTP-glucose glucosyltransferase family protein [Populus trichocarpa]	XP_002318525, EEE96745	0.0	483	397
Pe69C7.18	Incomplete 5'	-	5	3300	837	DNAJ heat shock N-terminal domain-containing family [Populus trichocarpa]	278	gi|566174470|ref|XP_006381002.1|hypothetical protein POPTR_0006s04630g [Populus trichocarpa]gi|550335459|gb|ERP58799.1|hypothetical protein POPTR_0006s04630g [Populus trichocarpa]	XP_006381002, ERP58799	5.13E-134	275	239
Pe69F22.1	Incomplete 3'	-	10	2697	707	Pollen-specific SF21-like isoform X2	235	gi|743813338|ref|XP_011019494.1|PREDICTED: pollen-specific protein SF21-like isoform X2 [Populus euphratica]	XP_011019494	2.76E-148	235	220
Pe69F22.2	3 isoforms	-	4	3308	1146	Hypothetical protein POPTR_0006s24700g [Populus trichocarpa]	381	gi|566177987|ref|XP_002309508.2|hypothetical protein POPTR_0006s24700g [Populus trichocarpa]gi|550337023|gb|EEE93031.2|hypothetical protein POPTR_0006s24700g [Populus trichocarpa]	XP_002309508, EEE93031	4.20E-148	376	275
Pe69F22.3	1 isoform	+	14	6597	1554	Serine threonine phosphatase 2A 55 kda regulatory subunit B beta isoform-like isoform X1	517	gi|566177991|ref|XP_002308570.2|hypothetical protein POPTR_0006s24710g [Populus trichocarpa]gi|550337025|gb|EEE92093.2|hypothetical protein POPTR_0006s24710g [Populus trichocarpa]	XP_002308570, EEE92093	0.0	522	471
Pe69F22.4	3 isoforms	+	2	2774	657	NADH dehydrogenase [ubiquinone] iron-sulfur mitochondrial	218	gi|255550832|ref|XP_002516464.1|PREDICTED: NADH dehydrogenase [ubiquinone] iron-sulfur protein 7, mitochondrial [Ricinus communis]gi|223544284|gb|EEF45805.1|NADH-plastoquinone oxidoreductase, putative [Ricinus communis]	XP_002516464, EEF45805	1.58E-132	218	200
Pe69F22.5		+	5	6070	1710	KH domain-containing isoform 1 [Theobroma cacao]	569	gi|566214296|ref|XP_002324756.2|hypothetical protein POPTR_0018s05010g [Populus trichocarpa]gi|550318073|gb|EEF03321.2|hypothetical protein POPTR_0018s05010g [Populus trichocarpa]	XP_002324756, EEF03321	0.0	581	458
Pe69F22.6	1 isoform	-	9	3141	1428	Ferrochelatase-chloroplastic-like	475	gi|566178005|ref|XP_002309511.2|hypothetical protein POPTR_0006s24820g [Populus trichocarpa]gi|550337031|gb|EEE93034.2|hypothetical protein POPTR_0006s24820g [Populus trichocarpa]	XP_002309511, EEE93034	0.0	491	409

Pe69F22.7	1 isoform	-	11	6095	1548	Serine hydroxymethyltransferase chloroplastic-like [Nicotiana tabacum]	515	gi|224092216|ref|XP_002309513.1|glycine hydroxymethyltransferase family protein [Populus trichocarpa]gi|222855489|gb|EEE93036.1|glycine hydroxymethyltransferase family protein [Populus trichocarpa]	XP_002309513, EEE93036	0.0	528	478
Pe69F22.8		+	3	1008	780	PREDICTED: uncharacterized protein LOC8275622 [Ricinus communis]	259	gi|255550842|ref|XP_002516469.1|PREDICTED: uncharacterized protein LOC8275622 [Ricinus communis]gi|223544289|gb|EEF45810.1|conserved hypothetical protein [Ricinus communis]	XP_002516469, EEF45810	8.93E-158	262	242
Pe69F22.9	1 isoform	-	4	3989	750	RNA-binding [Theobroma cacao]	249	gi|802632468|ref|XP_012077323.1|PREDICTED: uncharacterized protein LOC105638175 isoform X1 [Jatropha curcas]gi|643724923|gb|KDP34124.1|hypothetical protein JCGZ_07695 [Jatropha curcas]	XP_012077323, KDP34124	1.30E-102	255	205
Pe69F22.10		+	9	3819	1473	Vacuolar-processing enzyme-like	490	gi|255550848|ref|XP_002516472.1|PREDICTED: vacuolar-processing enzyme [Ricinus communis]gi|223544292|gb|EEF45813.1|Vacuolar-processing enzyme precursor, putative [Ricinus communis]	XP_002516472, EEF45813	0.0	493	446
Pe69F22.11		+	3	438	207	---Na---	68	No Blast Hit				
Pe69F22.12		+	5	4450	927	Myb D isoform X2 [Gossypium hirsutum]	308	gi|571449212|ref|XP_006578074.1|PREDICTED: uncharacterized protein LOC100807540 isoform X2 [Glycine max]gi|947113182|gb|KRH61484.1|hypothetical protein GLYMA_04G050000 [Glycine max]gi|947113183|gb|KRH61485.1|hypothetical protein GLYMA_04G050000 [Glycine max]	XP_006578074, KRH61484, KRH61485	1.63E-96	286	211
Pe69F22.13		-	4	3461	495	UPF0587 c1orf123 homolog	164	gi|224092234|ref|XP_002309521.1|hypothetical protein POPTR_0006s24980g [Populus trichocarpa]gi|222855497|gb|EEE93044.1|hypothetical protein POPTR_0006s24980g [Populus trichocarpa]	XP_002309521, EEE93044	2.10E-92	166	149
Pe69G18.1		-	2	1640	1434	PREDICTED: uncharacterized protein LOC107177920	477	gi|985458955|ref|XP_015387963.1|PREDICTED: uncharacterized protein LOC107177920 [Citrus sinensis]	XP_015387963	9.12E-62	478	223
Pe69G18.2		-	1	808	387	---Na---	128	No Blast Hit				
Pe69G18.3		-	3	1625	735	Transposon Ty3-I Gag-Pol poly	244	gi|823262277|ref|XP_012463885.1|PREDICTED: uncharacterized protein LOC105783159 [Gossypium raimondii]	XP_012463885	1.35E-32	173	99
Pe69G18.4		-	4	3329	1419	CHROMATIN REMODELING 4-like isoform X1 [Populus euphratica]	472	gi|1000987098|ref|XP_015576951.1|PREDICTED: uncharacterized protein LOC8274695 [Ricinus communis]	XP_015576951	7.56E-51	492	232
Pe69G18.5		-	22	8658	3582	Portal 56	1193	gi|802545798|ref|XP_012083996.1|PREDICTED: uncharacterized protein LOC105643473 [Jatropha curcas]	XP_012083996	0.0	1220	1025
Pe69G18.6		+	6	4219	1188	Myb-like transcription factor family [Medicago truncatula]	395	gi|743927993|ref|XP_011008189.1|PREDICTED: uncharacterized protein LOC105113636 isoform X1 [Populus euphratica]	XP_011008189	3.76E-56	429	226
Pe69G18.7		+	8	2917	1173	Elongation factor mitochondrial isoform X2	390	gi|802545804|ref|XP_012084028.1|PREDICTED: elongation factor Ts, mitochondrial isoform X1 [Jatropha curcas]gi|643739412|gb|KDP45166.1|hypothetical protein JCGZ_15031 [Jatropha curcas]	XP_012084028, KDP45166	0.0	391	322
Pe69G18.8		-	3	1034	828	Chlorophyll a-b binding chloroplastic	275	gi|590712862|ref|XP_007049480.1|Chlorophyll a-b binding protein 3, chloroplastic [Theobroma	XP_007049480, EOX93637	0.0	275	262

								cacao]gi|508701741|gb|EOX93637.1|Chlorophyll a-b binding protein 3, chloroplastic [Theobroma cacao]				
Pe69G18.9		-	1	228	228	---Na---	75	No Blast Hit				
Pe69G18.10		+	3	3250	411	Iron-stress related	136	gi|802545884|ref|XP_012084082.1|PREDICTED: uncharacterized protein LOC105643541 isoform X1 [Jatropha curcas]gi|643739416|gb|KDP45170.1|hypothetical protein JCGZ_15035 [Jatropha curcas]	XP_012084082, KDP45170	1.10E-38	106	83
Pe69G18.11		-	7	3368	1533	Adenine DNA glycosylase	510	gi|743928001|ref|XP_011008193.1|PREDICTED: A/G-specific adenine DNA glycosylase [Populus euphratica]	XP_011008193	0.0	511	410
Pe69G18.12		+	8	2589	870	Glyoxylate succinic semialdehyde reductase 1	289	gi|743928016|ref|XP_011008201.1|PREDICTED: glyoxylate/succinic semialdehyde reductase 1-like [Populus euphratica]gi|743937433|ref|XP_011013122.1|PREDICTED: glyoxylate/succinic semialdehyde reductase 1-like [Populus euphratica]	XP_011008201, XP_011013122	0.0	289	278
Pe69G18.13	1 isoform	-	8	6197	2565	Auxilin-related 2-like [Glycine max]	854	gi|224131974|ref|XP_002321224.1|auxilin-related family protein [Populus trichocarpa]gi|222861997|gb|EEE99539.1|auxilin-related family protein [Populus trichocarpa]	XP_002321224, EEE99539	0.0	896	574
Pe69G18.14		+	4	2036	1107	Cytochrome b561 and DOMON domain-containing At3g07570-like	368	gi|224129298|ref|XP_002320550.1|hypothetical protein POPTR_0014s17170g [Populus trichocarpa]gi|222861323|gb|EEE98865.1|hypothetical protein POPTR_0014s17170g [Populus trichocarpa]	XP_002320550, EEE98865	3.68E-166	365	292
Pe69G18.15	2 isoforms	+	3	1815	210	Fanconi anemia group D2 [Theobroma cacao]	74	gi|566204735|ref|XP_006375592.1|hypothetical protein POPTR_0014s17190g, partial [Populus trichocarpa]gi|550324388|gb|ERP53389.1|hypothetical protein POPTR_0014s17190g, partial [Populus trichocarpa]	XP_006375592, ERP53389	1.86E-12	66	45
Pe69G18.16		-	7	5053	774	Uncharacterized protein	257	gi|1000954786|ref|XP_015578117.1|PREDICTED: uncharacterized protein LOC8288313 [Ricinus communis]	XP_015578117	6.31E-135	246	218
Pe69G18.17		-	4	2188	1449	Mitochondrial transcription termination factor family isoform 1 [Theobroma cacao]	482	gi|802539386|ref|XP_012070783.1|PREDICTED: uncharacterized protein LOC105632922 [Jatropha curcas]	XP_012070783	0.0	481	356
Pe69G18.18	1 isoform	+	5	3166	870	Peroxisomal membrane 13	289	gi|568830159|ref|XP_006469374.1|PREDICTED: peroxisomal membrane protein 13 [Citrus sinensis]	XP_006469374	4.45E-115	298	244
Pe69G18.19		-	9	3021	891	60S ribosomal L5	296	gi|566204743|ref|XP_002321226.2|hypothetical protein POPTR_0014s17230g [Populus trichocarpa]gi|550324392|gb|EEE99541.2|hypothetical protein POPTR_0014s17230g [Populus trichocarpa]	XP_002321226, EEE99541	0.0	289	277
Pe69G18.20		+	1	716	348	PREDICTED: uncharacterized protein LOC107888408	115	gi|1029008223|ref|XP_016667999.1|PREDICTED: uncharacterized protein LOC107888408 [Gossypium hirsutum]	XP_016667999	2.72E-01	114	55
Pe69G18.21	Incomplete 3'	+	5	4275	3045	Formin 1	1015	gi|255583387|ref|XP_002532454.1|PREDICTED: formin-like protein 1 [Ricinus communis]gi|223527844|gb|EEF29940.1|conserved hypothetical protein [Ricinus communis]	XP_002532454, EEF29940	0.0	1080	737
Pe69H24.1	Incomplete 3'	-	6	1500	947	Adomet-dependent rRNA methyltransferase spb1	315	gi|802564176|ref|XP_012067253.1|PREDICTED: adoMet-dependent rRNA methyltransferase spb1 [Jatropha	XP_012067253, KDP41776	0.0	316	303

							curcas]gi|643735135|gb|KDP41776.1|hypothetical protein JCGZ_26794 [Jatropha curcas]				
Pe69H24.2	-	3	2338	585	RER1A-like [Gossypium hirsutum]	194	gi|823203783|ref|XP_012436262.1|PREDICTED: protein RER1A-like [Gossypium raimondii]gi|1029111676|ref|XP_016734338.1|PREDICTED: protein RER1A-like [Gossypium hirsutum]gi|763780430|gb|KJB47501.1|hypothetical protein B456_008G029700 [Gossypium raimondii]gi|763780431|gb|KJB47502.1|hypothetical protein B456_008G029700 [Gossypium raimondii]gi|763780432|gb|KJB47503.1|hypothetical protein B456_008G029700 [Gossypium raimondii]	XP_012436262, XP_016734338, KJB47501, KJB47502, KJB47503	6.84E-116	194	180
Pe69H24.3	+	3	2512	1980	F-box family isoform 1 [Theobroma cacao]	659	gi|1000979906|ref|XP_015570873.1|PREDICTED: uncharacterized protein LOC8285333 isoform X1 [Ricinus communis]gi|1000979908|ref|XP_015570874.1|PREDICTED: uncharacterized protein LOC8285333 isoform X1 [Ricinus communis]gi|223547934|gb|EEF49426.1|hypothetical protein RCOM_1447500 [Ricinus communis]	XP_015570873, XP_015570874, EEF49426	3.36E-160	719	418
Pe69H24.4	-	6	2056	1182	SNF1-related kinase regulatory subunit gamma-1-like	393	gi|743941874|ref|XP_011015436.1|PREDICTED: SNF1-related protein kinase regulatory subunit gamma-1-like [Populus euphratica]	XP_011015436	9.57E-156	435	305
Pe69H24.5	-	14	3419	1557	Glucose-1-phosphate adenylyltransferase large subunit 1-like [Populus euphratica]	518	gi|224103389|ref|XP_002313036.1|ADP-glucose pyrophosphorylase large subunit family protein [Populus trichocarpa]gi|222849444|gb|EEE86991.1|ADP-glucose pyrophosphorylase large subunit family protein [Populus trichocarpa]	XP_002313036, EEE86991	0.0	528	482
Pe69H24.6	-	3	1540	327	40S ribosomal S25-4-like [Gossypium hirsutum]	108	gi|729405964|ref|XP_010555290.1|PREDICTED: 40S ribosomal protein S25-4 [Tarenaya hassleriana]	XP_010555290	1.37E-65	108	107
Pe69H24.7	-	2	335	234	Defensin 19	77	gi|727434288|ref|XP_010498383.1|PREDICTED: defensin-like protein 19 [Camelina sativa]	XP_010498383	2.38E-15	76	50
Pe69H24.8	-	10	6461	1584	Bifunctional dihydrofolate reductase-thymidylate synthase-like isoform X1 [Citrus sinensis]	527	gi|802564134|ref|XP_012067239.1|PREDICTED: bifunctional dihydrofolate reductase-thymidylate synthase 1 [Jatropha curcas]gi|643735124|gb|KDP41765.1|hypothetical protein JCGZ_26783 [Jatropha curcas]	XP_012067239, KDP41765	0.0	527	493
Pe69H24.9	-	8	3251	1449	Diaminopimelate decarboxylase family [Populus trichocarpa]	482	gi|802564131|ref|XP_012067238.1|PREDICTED: diaminopimelate decarboxylase 2, chloroplastic-like [Jatropha curcas]gi|643735123|gb|KDP41764.1|hypothetical protein JCGZ_26782 [Jatropha curcas]	XP_012067238, KDP41764	0.0	489	458
Pe69H24.10	-	14	4341	1866	Sec14p-like phosphatidylinositol transfer family isoform 1 [Theobroma cacao]	621	gi|802564125|ref|XP_012067236.1|PREDICTED: phosphatidylinositol/phosphatidylcholine transfer protein SFH8 isoform X2 [Jatropha curcas]	XP_012067236	0.0	625	576
Pe69H24.11	-	8	3150	1125	Transcription factor TFIIIB component B isoform X5 [Citrus sinensis]	374	gi|1000979949|ref|XP_015570891.1|PREDICTED: uncharacterized protein LOC8285747 isoform X7 [Ricinus communis]	XP_015570891	1.83E-86	385	243
Pe69H24.12	+	1	462	462	Bzip transcription factor family [Populus trichocarpa]	153	gi|224105353|ref|XP_002313781.1|bZIP transcription factor family protein [Populus trichocarpa]gi|118484762|gb|ABK94250.1|unknown [Populus trichocarpa]gi|118487006|gb|ABK95334.1|unkno	XP_002313781, ABK94250, ABK95334, EEE87736	6.48E-53	136	111

								wn [Populus trichocarpa]gi|222850189|gb|EEE87736.1|bZIP transcription factor family protein [Populus trichocarpa]				
Pe69H24.13		+	9	3264	1017	Chaperone DNAJ 10-like	371	gi|255543767|ref|XP_002512946.1|PREDICTED : chaperone protein DNAJ 10 [Ricinus communis]gi|223547957|gb|EEF49449.1|Chaperone protein DNAJ, putative [Ricinus communis]	XP_002512946, EEF49449	0.0	338	312
Pe69H24.14		+	3	734	297	Aspartate carbamoyltransferase [Gossypium arboreum]	98	gi|224105349|ref|XP_002313779.1|hypothetical protein POPTR_0009s12270g [Populus trichocarpa]gi|222850187|gb|EEE87734.1|hypothetical protein POPTR_0009s12270g [Populus trichocarpa]	XP_002313779, EEE87734	5.86E-32	100	79
Pe69H24.15		+	4	3117	1143	A-agglutinin anchorage subunit [Jatropha curcas]	380	gi|566166886|ref|XP_002305445.2|hypothetical protein POPTR_0004s16580g [Populus trichocarpa]gi|550341173|gb|EEE85956.2|hypothetical protein POPTR_0004s16580g [Populus trichocarpa]	XP_002305445, EEE85956	7.67E-146	377	279
Pe69H24.16		+	2	1086	597	Late embryogenesis abundant -like	198	gi|442022395|gb|AGC51773.1|dehydrin protein [Manihot esculenta]	AGC51773	2.35E-36	207	126
Pe69H24.17		+	9	2204	1011	Bifunctional phosphatase chloroplastic	336	gi|566187626|ref|XP_002313028.2|hypothetical protein POPTR_0009s12340g [Populus trichocarpa]gi|550331578|gb|EEE86983.2|hypothetical protein POPTR_0009s12340g [Populus trichocarpa]	XP_002313028, EEE86983	0.0	333	285
Pe69H24.18		-	10	2752	1203	26S protease regulatory subunit 10B homolog A	400	gi|694452754|ref|XP_009351259.1|PREDICTED : 26S protease regulatory subunit 10B homolog A-like [Pyrus x bretschneideri]	XP_009351259	0.0	398	393
Pe69H24.19	1 isoform	-	10	5227	1989	SUMO-activating enzyme subunit 2	662	gi|743793714|ref|XP_011047909.1|PREDICTED : SUMO-activating enzyme subunit 2 isoform X2 [Populus euphratica]	XP_011047909	0.0	610	537
Pe69H24.20		+	6	5333	2559	CHROMATIN REMODELING 35-like isoform X1 [Ziziphus jujuba]	852	gi|743793674|ref|XP_011047795.1|PREDICTED : SNF2 domain-containing protein CLASSY 3 [Populus euphratica]	XP_011047795	0.0	856	688
Pe69H24.21	2 isoforms / Incomplete 3'	+	1	2721	885	BEL1-like homeodomain 6	295	gi|802564046|ref|XP_012067209.1|PREDICTED : BEL1-like homeodomain protein 6 [Jatropha curcas]gi|643735108|gb|KDP41749.1|hypothetical protein JCGZ_26767 [Jatropha curcas]	XP_012067209, KDP41749	5.93E-142	297	246
Pe69O16.1	Incomplete 5'	+	1	1347	1347	Pentatricopeptide repeat-containing At3g02330	448	gi|566166021|ref|XP_006384245.1|hypothetical protein POPTR_0004s11010g [Populus trichocarpa]gi|550340791|gb|ERP62042.1|hypothetical protein POPTR_0004s11010g [Populus trichocarpa]	XP_006384245, ERP62042	0.0	444	392
Pe69O16.2		-	4	3506	2058	WPP domain-interacting tail-anchored 1 [Vitis vinifera]	685	gi|566213394|ref|XP_006373525.1|hypothetical protein POPTR_0017s14520g [Populus trichocarpa]gi|550320347|gb|ERP51322.1|hypothetical protein POPTR_0017s14520g [Populus trichocarpa]	XP_006373525, ERP51322	0.0	709	522
Pe69O16.3		+	5	3211	1383	Probable sodium-coupled neutral amino acid transporter 6 [Gossypium hirsutum]	460	gi|743820358|ref|XP_011021127.1|PREDICTED : probable sodium-coupled neutral amino acid transporter 6 [Populus euphratica]	XP_011021127	0.0	460	428
Pe69O16.4		-	17	11799	6411	Furry homolog [Solanum tuberosum]	2136	gi|802751754|ref|XP_012088092.1|PREDICTED : protein furry homolog [Jatropha curcas]	XP_012088092	0.0	2148	1935
Pe69O16.5		+	12	4921	2148	Kanadaptin	715	gi|743933041|ref|XP_011010826.1|PREDICTED : kanadaptin [Populus euphratica]	XP_011010826	0.0	664	563

Pe69O16.6	-	19	9143	3129	DNA-directed RNA polymerase chloroplastic mitochondrial	1042	gi|743933039|ref|XP_011010825.1|PREDICTED: DNA-directed RNA polymerase 2B, chloroplastic/mitochondrial-like isoform X2 [Populus euphratica]	XP_011010825	0.0	1045	885
Pe69O16.7	-	6	3226	1143	Alpha beta hydrolase domain-containing 17B [Jatropha curcas]	380	gi|743820320|ref|XP_011021114.1|PREDICTED: alpha/beta hydrolase domain-containing protein 17B-like [Populus euphratica]gi|743820324|ref|XP_011021116.1|PREDICTED: alpha/beta hydrolase domain-containing protein 17B-like [Populus euphratica]	XP_011021114, XP_011021116	0.0	393	348
Pe69O16.8	+	1	2600	1317	F-box kelch-repeat At5g15710	438	gi|255585841|ref|XP_002533598.1|PREDICTED: F-box/kelch-repeat protein At5g15710 [Ricinus communis]gi|223526527|gb|EEF28789.1|ubiquitin-protein ligase, putative [Ricinus communis]	XP_002533598, EEF28789	0.0	437	400
Pe69O16.9	+	5	1329	657	Transmembrane [Medicago truncatula]	218	gi|743911107|ref|XP_010999413.1|PREDICTED: uncharacterized protein LOC105107254 isoform X2 [Populus euphratica]	XP_010999413	1.89E-31	201	120
Pe69O16.10-11	-	5	1328	735	GDSL esterase lipase 7-like	124	gi|566166053|ref|XP_006384261.1|hypothetical protein POPTR_0004s11160g [Populus trichocarpa]gi|550340807|gb|ERP62058.1|hypothetical protein POPTR_0004s11160g [Populus trichocarpa]	XP_006384261, ERP62058	1.45E-44	121	95
Pe69O16.12	+	10	5353	1758	O-fucosyltransferase family isoform 1 [Theobroma cacao]	585	gi|1000952340|ref|XP_015578921.1|PREDICTED: uncharacterized protein At1g04910 [Ricinus communis]	XP_015578921	0.0	572	529
Pe69O16.13	+	1	590	177	Hypothetical protein POPTR_0004s11190g [Populus trichocarpa]	58	gi|566166059|ref|XP_006384264.1|hypothetical protein POPTR_0004s11190g [Populus trichocarpa]gi|550340810|gb|ERP62061.1|hypothetical protein POPTR_0004s11190g [Populus trichocarpa]	XP_006384264, ERP62061	5.24E+00	51	31
Pe69O16.14	+	7	4495	996	Transcription factor BIM2-like isoform X1	331	gi|743911119|ref|XP_010999419.1|PREDICTED: transcription factor BIM2-like isoform X1 [Populus euphratica]	XP_010999419	2.88E-127	337	256
Pe69O16.15	-	8	3279	1281	O-fucosyltransferase family isoform 1 [Theobroma cacao]	426	gi|743820251|ref|XP_011021095.1|PREDICTED: uncharacterized protein At1g04910 [Populus euphratica]	XP_011021095	0.0	417	382
Pe69O16.16	+	4	1672	549	U3 small nucleolar ribonucleo IMP3-like	182	gi|255569960|ref|XP_002525943.1|PREDICTED: U3 small nucleolar ribonucleoprotein protein IMP3 [Ricinus communis]gi|223534772|gb|EEF36463.1|U3 small nucleolar ribonucleoprotein protein IMP3, putative [Ricinus communis]	XP_002525943, EEF36463	3.18E-113	182	175
Pe69O16.17	-	12	17670	14583	Auxin transport BIG	4860	gi|743820235|ref|XP_011021091.1|PREDICTED: auxin transport protein BIG isoform X1 [Populus euphratica]	XP_011021091	0.0	4850	4223
Pe69O16.18	-	7	1829	777	60S ribosomal L7a [Populus trichocarpa]	258	gi|255569948|ref|XP_002525937.1|PREDICTED: 60S ribosomal protein L7a [Ricinus communis]gi|223534766|gb|EEF36457.1|60S ribosomal protein L7a, putative [Ricinus communis]	XP_002525937, EEF36457	2.17E-172	258	254
Pe69O16.19	-	1	810	810	Serpin-ZX [Ricinus communis]	269	gi|802751696|ref|XP_012088079.1|PREDICTED: serpin-ZX-like [Jatropha curcas]	XP_012088079	7.79E-97	265	198
Pe71E3.1	+	5	3005	1641	IQ-DOMAIN 14 [Jatropha curcas]	546	gi|802553851|ref|XP_012065034.1|PREDICTED: protein IQ-DOMAIN 14 [Jatropha curcas]gi|802553853|ref|XP_012065035.1|PREDICTED: protein IQ-DOMAIN 14 [Jatropha curcas]gi|802553855|ref|XP_012065036.1|PREDICTED: protein IQ-DOMAIN 14 [Jatropha	XP_012065034, XP_012065035, XP_012065036, XP_012065037, XP_012065038, XP_012065039,	0.0	548	469

								curcas]gi|802554698|ref|XP_012065037.1|PREDICTED: protein IQ-DOMAIN 14 [Jatropha curcas]gi|802554700|ref|XP_012065038.1|PREDICTED: protein IQ-DOMAIN 14 [Jatropha curcas]gi|802554702|ref|XP_012065039.1|PREDICTED: protein IQ-DOMAIN 14 [Jatropha curcas]gi|643738248|gb|KDP44236.1|hypothetical protein JCGZ_05703 [Jatropha curcas]	KDP44236			
Pe71E3.2		+	3	2451	1191	Stearoyl-ACP desaturase	396	gi|743884833|ref|XP_011037382.1|PREDICTED: acyl-[acyl-carrier-protein] desaturase, chloroplastic [Populus euphratica]gi|743884837|ref|XP_011037383.1|PREDICTED: acyl-[acyl-carrier-protein] desaturase, chloroplastic [Populus euphratica]	XP_011037382, XP_011037383	0.0	396	380
Pe71E3.3		-	8	2462	1452	Fatty acyl- reductase 2	483	gi|743896211|ref|XP_011041378.1|PREDICTED: fatty acyl-CoA reductase 2-like isoform X1 [Populus euphratica]	XP_011041378	0.0	502	349
Pe71E3.4		-	3	574	390	Fatty acyl- reductase 2	129	gi|720005897|ref|XP_010257805.1|PREDICTED: LOW QUALITY PROTEIN: fatty acyl-CoA reductase 2 [Nelumbo nucifera]	XP_010257805	1.25E-46	128	103
Pe71E3.5		-	9	2527	1566	Jojoba acyl reductase-related male sterility [Theobroma cacao]	521	gi|743896211|ref|XP_011041378.1|PREDICTED: fatty acyl-CoA reductase 2-like isoform X1 [Populus euphratica]	XP_011041378	0.0	509	374
Pe71E3.6		-	5	3360	1698	Zinc finger BED domain-containing RICESLEEPER 2-like	565	gi|698552078|ref|XP_009769521.1|PREDICTED: zinc finger BED domain-containing protein RICESLEEPER 2-like [Nicotiana sylvestris]	XP_009769521	1.55E-57	266	167
Pe71E3.7		+	1	1857	1203	Calcineurin-like phosphoesterase family [Populus trichocarpa]	400	gi|802617477|ref|XP_012075416.1|PREDICTED: uncharacterized protein At1g18480 [Jatropha curcas]gi|643726448|gb|KDP35155.1|hypothetical protein JCGZ_10689 [Jatropha curcas]	XP_012075416, KDP35155	0.0	398	341
Pe71E3.8		+	3	1368	684	FAM136A-like [Camelina sativa]	227	gi|566201059|ref|XP_006376445.1|hypothetical protein POPTR_0013s13110g [Populus trichocarpa]gi|550325721|gb|ERP54242.1|hypothetical protein POPTR_0013s13110g [Populus trichocarpa]	XP_006376445, ERP54242	6.34E-68	151	127
Pe71E3.9		-	1	207	207	Transmembrane [Medicago truncatula]	68	gi|590654547|ref|XP_007033727.1|Uncharacterized protein TCM_019853 [Theobroma cacao]gi|508712756|gb|EOY04653.1|Uncharacterized protein TCM_019853 [Theobroma cacao]	XP_007033727, EOY04653	1.48E-26	68	64
Pe71E3.10		-	4	2507	894	(+)-Neomenthol dehydrogenase	297	gi|566201065|ref|XP_006376448.1|hypothetical protein POPTR_0013s13140g [Populus trichocarpa]gi|550325724|gb|ERP54245.1|hypothetical protein POPTR_0013s13140g [Populus trichocarpa]	XP_006376448, ERP54245	7.87E-155	284	249
Pe71E3.11		-	7	3727	597	Cysteine synthase-like	198	gi|695027581|ref|XP_009401140.1|PREDICTED: cysteine synthase-like [Musa acuminata subsp. malaccensis]	XP_009401140	1.48E-117	197	190
Pe74I6.1	Incomplete 3'	-	1	813	813	PREDICTED: uncharacterized protein LOC104106381	271	gi|697118574|ref|XP_009613217.1|PREDICTED: uncharacterized protein LOC104106381 [Nicotiana tomentosiformis]	XP_009613217	3.02E-34	189	110
Pe74I6.2		-	1	1605	1605	Histone-lysine N-methyltransferase family member SUVH9 [Gossypium raimondii]	534	gi|802633719|ref|XP_012077634.1|PREDICTED: histone-lysine N-methyltransferase family member SUVH9-like [Jatropha curcas]gi|643724042|gb|KDP33342.1|hypothetical protein JCGZ_12891 [Jatropha curcas]	XP_012077634, KDP33342	0.0	504	399
Pe74I6.3		+	3	1047	543	AWPM-19-like membrane family [Populus trichocarpa]	180	gi|224107537|ref|XP_002314514.1|AWPM-19-like membrane family protein [Populus trichocarpa]gi|222863554|gb|EEF00685.1|AWP	XP_002314514, EEF00685	8.67E-101	181	162

								M-19-like membrane family protein [Populus trichocarpa]				
Pe74I6.4		-	1	2058	1035	Nucleolar matrix family [Populus trichocarpa]	344	gi|224102259|ref|XP_002312613.1|nucleolar matrix family protein [Populus trichocarpa]gi|222852433|gb|EEE89980.1|nucleolar matrix family protein [Populus trichocarpa]	XP_002312613, EEE89980	1.29E-131	345	279
Pe74I6.5		+	3	1056	813	Myb family transcription factor family [Populus trichocarpa]	270	gi|224099991|ref|XP_002311700.1|myb family transcription factor family protein [Populus trichocarpa]gi|222851520|gb|EEE89067.1|myb family transcription factor family protein [Populus trichocarpa]	XP_002311700, EEE89067	1.17E-131	273	232
Pe74I6.6	1 isoform	+	1	3586	1887	Pentatricopeptide repeat-containing At3g12770-like	628	gi|224107543|ref|XP_002314516.1|hypothetical protein POPTR_0010s07440g [Populus trichocarpa]gi|222863556|gb|EEF00687.1|hypothetical protein POPTR_0010s07440g [Populus trichocarpa]	XP_002314516, EEF00687	0.0	626	510
Pe74I6.7		+	1	2336	1503	Ammonium transporter family [Populus trichocarpa]	500	gi|566189592|ref|XP_002314518.2|ammonium transporter family protein [Populus trichocarpa]gi|550329294|gb|EEF00689.2|ammonium transporter family protein [Populus trichocarpa]	XP_002314518, EEF00689	0.0	500	468
Pe74I6.8		+	12	3854	1545	Carboxyl-terminal-processing peptidase chloroplastic isoform X1	514	gi|567854583|ref|XP_006420411.1|hypothetical protein CICLE_v10004718mg [Citrus clementina]gi|557522284|gb|ESR33651.1|hypothetical protein CICLE_v10004718mg [Citrus clementina]	XP_006420411, ESR33651	0.0	434	392
Pe74I6.9		+	4	3099	1749	Beta-amylase chloroplastic	582	gi|566189584|ref|XP_002314522.2|hypothetical protein POPTR_0010s07340g [Populus trichocarpa]gi|550329291|gb|EEF00693.2|hypothetical protein POPTR_0010s07340g [Populus trichocarpa]	XP_002314522, EEF00693	0.0	587	526
Pe74I6.10		+	3	681	285	Late embryogenesis abundant 1-like	94	gi|224107571|ref|XP_002314523.1|hypothetical protein POPTR_0010s07330g [Populus trichocarpa]gi|222863563|gb|EEF00694.1|hypothetical protein POPTR_0010s07330g [Populus trichocarpa]	XP_002314523, EEF00694	4.43E-09	102	62
Pe74I6.11	1 isoform	+	39	17655	4374	Myosin XI B isoform 1 [Theobroma cacao]	1457	gi|566184509|ref|XP_002311708.2|hypothetical protein POPTR_0008s17460g [Populus trichocarpa]gi|550333298|gb|EEE89075.2|hypothetical protein POPTR_0008s17460g [Populus trichocarpa]	XP_002311708, EEE89075	0.0	1526	1289
Pe74I6.12		-	1	204	204	---Na---	67	No Blast Hit				
Pe74I6.13		+	3	2043	1272	Probable indole-3-pyruvate monooxygenase YUCCA3	423	gi|224100011|ref|XP_002311709.1|flavin-containing monooxygenase family protein [Populus trichocarpa]gi|222851529|gb|EEE89076.1|flavin-containing monooxygenase family protein [Populus trichocarpa]	XP_002311709, EEE89076	0.0	421	387
Pe74I6.14		-	12	9109	2085	BLISTER [Theobroma cacao]	694	gi|743854130|ref|XP_011029707.1|PREDICTED: uncharacterized protein LOC105129379 isoform X1 [Populus euphratica]	XP_011029707	0.0	755	507
Pe74I6.15		+	2	1798	1302	E3 ubiquitin- ligase RING1 [Morus notabilis]	433	gi|590565275|ref|XP_007009903.1|Uncharacterized protein TCM_043210 [Theobroma cacao]gi|508726816|gb|EOY18713.1|Uncharacterized protein TCM_043210 [Theobroma cacao]	XP_007009903, EOY18713	5.52E-14	260	126
Pe74I6.16_Pe74I6.17	1 isoform	+	2	8881	672	Probable glutathione S-transferase	223	gi|224107591|ref|XP_002314529.1|hypothetical protein POPTR_0010s07070g [Populus	XP_002314529, EEF00700,	2.19E-100	223	182

								trichocarpa]gi|222863569|gb|EEF00700.1|hypothetical protein POPTR_0010s07070g [Populus trichocarpa]gi|283135850|gb|ADB11316.1|tau class glutathione transferase GSTU12 [Populus trichocarpa]	ADB11316			
Pe74I6.18		-	1	615	615	Dual specificity tyrosine-phosphorylation-regulated kinase 1A-like	204	gi|567889246|ref|XP_006437145.1|hypothetical protein CICLE_v10033421mg [Citrus clementina]gi|557539341|gb|ESR50385.1|hypothetical protein CICLE_v10033421mg [Citrus clementina]	XP_006437145, ESR50385	9.69E-15	149	79
Pe74I6.19	2 isoforms	-	9	4782	768	ELMO CED-12 family isoform 2 [Theobroma cacao]	255	gi|590692390|ref|XP_007044043.1|ELMO/CED-12 family protein isoform 2 [Theobroma cacao]gi|508707978|gb|EOX99874.1|ELMO/CED-12 family protein isoform 2 [Theobroma cacao]	XP_007044043, EOX99874	5.17E-146	242	219
Pe74I6.20		-	4	2537	1314	Exostosin family isoform 1 [Theobroma cacao]	437	gi|255542540|ref|XP_002512333.1|PREDICTED: probable arabinosyltransferase ARAD1 [Ricinus communis]gi|223548294|gb|EEF49785.1|catalytic, putative [Ricinus communis]	XP_002512333, EEF49785	0.0	438	373
Pe74I6.21	1 isoform	+	7	5053	534	60S ribosomal L17-2	177	gi|802724253|ref|XP_012085663.1|PREDICTED: 60S ribosomal protein L17-2 [Jatropha curcas]	XP_012085663	6.79E-118	174	171
Pe74I6.22		+	1	1727	282	Probable signal peptidase complex subunit 1	93	gi|593791310|ref|XP_007158694.1|hypothetical protein PHAVU_002G174400g [Phaseolus vulgaris]gi|561032109|gb|ESW30688.1|hypothetical protein PHAVU_002G174400g [Phaseolus vulgaris]	XP_007158694, ESW30688	7.43E-43	93	81
Pe74I6.23	Incomplete 3'	+	1	426	426	F-box kelch-repeat At1g67480-like [Populus euphratica]	142	gi|802724242|ref|XP_012085660.1|PREDICTED: F-box/kelch-repeat protein At1g67480 [Jatropha curcas]gi|643714123|gb|KDP26788.1|hypothetical protein JCGZ_17946 [Jatropha curcas]	XP_012085660, KDP26788	5.99E-59	143	115
Pe75A21.1		-	1	216	216	---Na---	71	No Blast Hit				
Pe75A21.2		+	1	2750	762	Sterile alpha motif domain-containing isoform 1 [Theobroma cacao]	253	gi|802777419|ref|XP_012090880.1|PREDICTED: uncharacterized protein LOC105648978 [Jatropha curcas]gi|643705385|gb|KDP21931.1|hypothetical protein JCGZ_03069 [Jatropha curcas]	XP_012090880, KDP21931	1.22E-85	251	179
Pe75A21.3		+	3	3351	1296	Amino acid transporter ANT1-like	431	gi|255566253|ref|XP_002524114.1|PREDICTED: amino acid transporter ANT1 [Ricinus communis]gi|223536682|gb|EEF38324.1|amino acid transporter, putative [Ricinus communis]	XP_002524114, EEF38324	0.0	431	367
Pe75A21.4	1 isoform	+	10	5730	2076	Probable glutamate carboxypeptidase 2	691	gi|802776776|ref|XP_012090878.1|PREDICTED: probable glutamate carboxypeptidase 2 isoform X2 [Jatropha curcas]gi|643705382|gb|KDP21928.1|hypothetical protein JCGZ_03066 [Jatropha curcas]	XP_012090878, KDP21928	0.0	704	567
Pe75A21.5		-	7	4172	1272	Plant T8M16-80 [Medicago truncatula]	423	gi|743844644|ref|XP_011027258.1|PREDICTED: uncharacterized protein LOC105127599 isoform X2 [Populus euphratica]	XP_011027258	0.0	421	362
Pe75A21.6		-	1	162	162	---Na---	53	No Blast Hit				
Pe75A21.7		-	3	1099	177	---Na---	58	No Blast Hit				
Pe75A21.8		-	3	1175	495	---Na---	164	No Blast Hit				
Pe75A21.9		+	1	396	396	Probable pectinesterase pectinesterase inhibitor 13	131	gi|720100240|ref|XP_010248181.1|PREDICTED: uncharacterized protein LOC104591088	XP_010248181	2.02E-16	119	75

								[Nelumbo nucifera]				
Pe75A21.10		+	1	312	312	Uncharacterized protein TCM_012596 [Theobroma cacao]	103	gi|590665339|ref|XP_007036712.1|Uncharacterized protein TCM_012596 [Theobroma cacao]gi|508773957|gb|EOY21213.1|Uncharacterized protein TCM_012596 [Theobroma cacao]	XP_007036712, EOY21213	2.68E-12	90	56
Pe75A21.11		+	1	282	282	---Na---	93	No Blast Hit				
Pe75A21.12		+	1	360	360	---Na---	119	No Blast Hit				
Pe75A21.13		-	1	396	396	Gag protease poly [Theobroma cacao]	131	gi|596296602|ref|XP_007227199.1|hypothetical protein PRUPE_ppa019381mg [Prunus persica]gi|462424135|gb|EMJ28398.1|hypothetical protein PRUPE_ppa019381mg [Prunus persica]	XP_007227199, EMJ28398	1.02E-12	111	64
Pe75D12.1		-	1	369	369	---Na---	122	No Blast Hit				
Pe75D12.2		-	1	792	792	---Na---	263	No Blast Hit				
Pe75D12.3		-	1	351	351	PREDICTED: uncharacterized protein LOC104886366, partial	116	gi|731381935|ref|XP_010669126.1|PREDICTED: uncharacterized protein LOC104886366, partial [Beta vulgaris subsp. vulgaris]	XP_010669126	3.20E-40	105	84
Pe75D12.4		-	1	549	549	---Na---	182	No Blast Hit				
Pe75D12.5		+	4	1120	339	---Na---	112	No Blast Hit				
Pe75D12.6		+	5	3255	1224	Gag protease poly [Theobroma cacao]	407	gi|590568718|ref|XP_007010875.1|DNA/RNA polymerases superfamily protein, putative [Theobroma cacao]gi|508727788|gb|EOY19685.1|DNA/RNA polymerases superfamily protein, putative [Theobroma cacao]	XP_007010875, EOY19685	9.98E-29	332	150
Pe75D12.7		+	2	589	216	---Na---	71	No Blast Hit				
Pe75D12.8		+	2	269	234	---Na---	77	No Blast Hit				
Pe75D12.9		+	1	219	219	Gag protease poly [Theobroma cacao]	72	gi|590580527|ref|XP_007014093.1|Gag protease polyprotein [Theobroma cacao]gi|508784456|gb|EOY31712.1|Gag protease polyprotein [Theobroma cacao]	XP_007014093, EOY31712	1.11E-04	73	44
Pe75D12.10	Incomplete 5'	-	1	270	270	---Na---	89	No Blast Hit				
Pe75F13.1		-	1	261	261	---Na---	86	No Blast Hit				
Pe75F13.2		+	3	1636	924	DNA RNA polymerases superfamily [Theobroma cacao]	307	gi|590728434|ref|XP_007099662.1|Gag protease polyprotein-like protein [Theobroma cacao]gi|508728474|gb|EOY20371.1|Gag protease polyprotein-like protein [Theobroma cacao]	XP_007099662, EOY20371	5.36E-06	142	66
Pe75F13.3		-	2	452	366	---Na---	121	No Blast Hit				
Pe75F13.4		+	1	408	408	---Na---	135	No Blast Hit				
Pe75F13.5		-	1	249	249	---Na---	82	No Blast Hit				
Pe75F13.6		+	1	180	180	---Na---	59	No Blast Hit				
Pe75F13.7		+	3	1395	1245	Gag protease poly [Theobroma cacao]	414	gi|590612264|ref|XP_007022336.1|Gag protease polyprotein [Theobroma cacao]gi|508721964|gb|EOY13861.1|Gag protease polyprotein [Theobroma cacao]	XP_007022336, EOY13861	3.55E-33	357	170
Pe75F20.1		-	2	675	480	PREDICTED: uncharacterized protein LOC107416916	159	gi|1009127929|ref|XP_015880953.1|PREDICTED: uncharacterized protein LOC107416916 [Ziziphus jujuba]	XP_015880953	3.01E-04	142	66

Pe75F20.2		-	1	417	417	Gag protease poly [Theobroma cacao]	138	gi|590689992|ref|XP_007043384.1|Gag protease polyprotein [Theobroma cacao]gi|508707319|gb|EOX99215.1|Gag protease polyprotein [Theobroma cacao]	XP_007043384, EOX99215	3.67E-17	92	60
Pe75F20.3		-	1	198	198	---Na---	65	No Blast Hit				
Pe75F20.4		-	1	294	294	---Na---	97	No Blast Hit				
Pe75F20.5		-	1	759	759	Hypothetical protein CISIN_1g046520mg	252	gi|641817638|gb|KDO38897.1|hypothetical protein CISIN_1g046520mg [Citrus sinensis]	KDO38897	1.60E-27	177	95
Pe75F20.6	3 isoforms	-	3	2481	273	Dual specificity phosphatase Cdc25	90	gi|567871519|ref|XP_006428349.1|hypothetical protein CICLE_v10013072mg [Citrus clementina]gi|567871521|ref|XP_006428350.1|hypothetical protein CICLE_v10013072mg [Citrus clementina]gi|568877326|ref|XP_006491690.1|PREDICTED: dual specificity phosphatase Cdc25 isoform X1 [Citrus sinensis]gi|557530406|gb|ESR41589.1|hypothetical protein CICLE_v10013072mg [Citrus clementina]gi|557530407|gb|ESR41590.1|hypothetical protein CICLE_v10013072mg [Citrus clementina]	XP_006428349, XP_006428350, XP_006491690, ESR41589, ESR41590	3.48E-32	83	73
Pe75F20.7	1 isoform	+	13	6418	1842	E3 ubiquitin- ligase RFWD3 isoform X1	613	gi|743810553|ref|XP_011018781.1|PREDICTED: E3 ubiquitin-protein ligase RFWD3 [Populus euphratica]gi|743810557|ref|XP_011018782.1|PREDICTED: E3 ubiquitin-protein ligase RFWD3 [Populus euphratica]gi|743810561|ref|XP_011018783.1|PREDICTED: E3 ubiquitin-protein ligase RFWD3 [Populus euphratica]	XP_011018781, XP_011018782, XP_011018783	0.0	637	445
Pe75F20.8		-	4	2484	885	Probable xyloglucan endotransglucosylase hydrolase 32	294	gi|802687694|ref|XP_012082446.1|PREDICTED: probable xyloglucan endotransglucosylase/hydrolase protein 32 [Jatropha curcas]gi|643717732|gb|KDP29175.1|hypothetical protein JCGZ_16564 [Jatropha curcas]	XP_012082446, KDP29175	0.0	293	276
Pe75F20.9		-	3	1544	246	---Na---	81	No Blast Hit				
Pe75F20.10		-	3	1981	240	---Na---	79	No Blast Hit				
Pe75F20.11		-	1	336	336	---Na---	111	No Blast Hit				
Pe75F20.12		-	2	2064	198	---Na---	65	No Blast Hit				
Pe75F20.13		+	1	261	261	---Na---	86	No Blast Hit				
Pe75F20.14		+	11	6115	1149	FAD synthase isoform X1 [Citrus sinensis]	382	gi|802687670|ref|XP_012082439.1|PREDICTED: FAD synthase isoform X3 [Jatropha curcas]gi|643717727|gb|KDP29170.1|hypothetical protein JCGZ_16559 [Jatropha curcas]	XP_012082439, KDP29170	0.0	381	349
Pe75K15.1		+	1	303	303	DNA RNA polymerases superfamily [Theobroma cacao]	100	gi|590581221|ref|XP_007014288.1|Uncharacterized protein TCM_039264 [Theobroma cacao]gi|508784651|gb|EOY31907.1|Uncharacterized protein TCM_039264 [Theobroma cacao]	XP_007014288, EOY31907	2.17E-26	98	69
Pe75K15.2		+	1	201	201	---Na---	66	No Blast Hit				
Pe75K15.3		+	2	762	453	Gag protease poly [Theobroma cacao]	150	gi|590568718|ref|XP_007010875.1|DNA/RNA polymerases superfamily protein, putative [Theobroma cacao]gi|508727788|gb|EOY19685.1|DNA/RNA	XP_007010875, EOY19685	4.64E-18	179	93

							polymerases superfamily protein, putative [Theobroma cacao]				
Pe75K15.4	+	1	264	264	---Na---	87	No Blast Hit				
Pe75K15.5	+	3	1064	840	Gag protease poly [Theobroma cacao]	279	gi|590691529|ref|XP_007043808.1|Gag protease polyprotein [Theobroma cacao]gi|508707743|gb|EOX99639.1|Gag protease polyprotein [Theobroma cacao]	XP_007043808, EOX99639	2.19E-20	119	75
Pe75K15.6	+	2	306	222	---Na---	73	No Blast Hit				
Pe75K15.7	+	3	1095	795	Gag protease poly [Theobroma cacao]	264	gi|590728434|ref|XP_007099662.1|Gag protease polyprotein-like protein [Theobroma cacao]gi|508728474|gb|EOY20371.1|Gag protease polyprotein-like protein [Theobroma cacao]	XP_007099662, EOY20371	4.28E-20	352	145
Pe75K15.8	+	1	552	552	PREDICTED: uncharacterized protein LOC106766267	183	gi|950999367|ref|XP_014506495.1|PREDICTED: uncharacterized protein LOC106766267 [Vigna radiata var. radiata]	XP_014506495	2.74E-44	162	120
Pe75K15.9	+	1	564	564	PREDICTED: uncharacterized protein LOC103328423	187	gi|645245765|ref|XP_008229034.1|PREDICTED: uncharacterized protein LOC103328423 [Prunus mume]	XP_008229034	5.68E-20	163	95
Pe75K15.10	+	2	777	702	Proline-rich receptor kinase PERK4	233	gi|802607599|ref|XP_012073893.1|PREDICTED: uncharacterized protein LOC105635422 [Jatropha curcas]	XP_012073893	5.74E-27	227	119
Pe75K15.11	-	2	517	291	PREDICTED: uncharacterized protein LOC105639525 [Jatropha curcas]	96	gi|802640777|ref|XP_012078997.1|PREDICTED: uncharacterized protein LOC105639525 [Jatropha curcas]gi|802640779|ref|XP_012078998.1|PREDICTED: uncharacterized protein LOC105639526 [Jatropha curcas]	XP_012078997, XP_012078998	4.41E-05	78	47
Pe75K15.12	+	1	360	360	---Na---	119	No Blast Hit				
Pe75K15.13	+	5	4193	2025	Gag protease poly [Theobroma cacao]	674	gi|590581218|ref|XP_007014287.1|Gag protease polyprotein [Theobroma cacao]gi|508784650|gb|EOY31906.1|Gag protease polyprotein [Theobroma cacao]	XP_007014287, EOY31906	6.71E-39	322	158
Pe75K15.14	+	1	417	417	Gag protease poly [Theobroma cacao]	138	gi|590583129|ref|XP_007014815.1|Gag protease polyprotein [Theobroma cacao]gi|508785178|gb|EOY32434.1|Gag protease polyprotein [Theobroma cacao]	XP_007014815, EOY32434	7.99E-21	126	81
Pe75K15.15	+	2	578	408	Gag protease poly [Theobroma cacao]	135	gi|590693137|ref|XP_007044250.1|DNA/RNA polymerases superfamily protein [Theobroma cacao]gi|508708185|gb|EOY00082.1|DNA/RNA polymerases superfamily protein [Theobroma cacao]	XP_007044250, EOY00082	5.08E-04	130	60
Pe75K15.16	+	1	336	336	---Na---	111	No Blast Hit				
Pe75K15.17	+	2	2251	2100	Gag protease poly [Theobroma cacao]	699	gi|590728434|ref|XP_007099662.1|Gag protease polyprotein-like protein [Theobroma cacao]gi|508728474|gb|EOY20371.1|Gag protease polyprotein-like protein [Theobroma cacao]	XP_007099662, EOY20371	7.06E-77	461	246
Pe75K15.18	+	1	398	315	---Na---	104	No Blast Hit				
Pe75K15.19	+	1	748	276	---Na---	101	No Blast Hit				
Pe75K15.20	+	3	2044	696	PREDICTED: uncharacterized protein LOC105778996	231	gi|823251192|ref|XP_012458202.1|PREDICTED: uncharacterized protein LOC105778996 [Gossypium raimondii]	XP_012458202	2.03E+00	40	30
Pe75K15.21	+	1	201	201	---Na---	66	No Blast Hit				

Pe75K15.22		+	2	1918	1830	Gag protease poly [Theobroma cacao]	609	gi|590728434|ref|XP_007099662.1|Gag protease polyprotein-like protein [Theobroma cacao]gi|508728474|gb|EOY20371.1|Gag protease polyprotein-like protein [Theobroma cacao]	XP_007099662, EOY20371	3.22E-69	420	218
Pe75K15.23		+	1	225	225	---Na---	74	No Blast Hit				
Pe75K15.24		+	1	186	186	---Na---	61	No Blast Hit				
Pe75K15.25		+	1	1173	234	---Na---	77	No Blast Hit				
Pe75N15.1		+	1	204	204	---Na---	67	No Blast Hit				
Pe75N15.2		+	2	612	252	---Na---	83	No Blast Hit				
Pe75N15.3		+	2	417	360	---Na---	119	No Blast Hit				
Pe75N15.4		+	1	414	414	Gag protease poly [Theobroma cacao]	137	gi|590650204|ref|XP_007032596.1|Gag protease polyprotein [Theobroma cacao]gi|508711625|gb|EOY03522.1|Gag protease polyprotein [Theobroma cacao]	XP_007032596, EOY03522	1.64E-15	109	69
Pe75N15.5		-	1	303	303	PREDICTED: uncharacterized protein LOC107175490	100	gi|985433085|ref|XP_015382400.1|PREDICTED: uncharacterized protein LOC107175490 [Citrus sinensis]	XP_015382400	2.46E-05	91	48
Pe75N15.6		+	1	396	396	---Na---	131	No Blast Hit				
Pe75N15.7		-	1	585	585	Gag protease poly [Theobroma cacao]	194	gi|590650921|ref|XP_007032766.1|Gag protease polyprotein [Theobroma cacao]gi|508711795|gb|EOY03692.1|Gag protease polyprotein [Theobroma cacao]	XP_007032766, EOY03692	3.57E-10	190	90
Pe75N15.8		-	1	522	522	Gag protease poly [Theobroma cacao]	173	gi|590580527|ref|XP_007014093.1|Gag protease polyprotein [Theobroma cacao]gi|508784456|gb|EOY31712.1|Gag protease polyprotein [Theobroma cacao]	XP_007014093, EOY31712	9.49E-07	68	44
Pe75N15.9		-	1	279	279	---Na---	92	No Blast Hit				
Pe75N15.10		-	1	714	714	Gag protease poly [Theobroma cacao]	237	gi|590691529|ref|XP_007043808.1|Gag protease polyprotein [Theobroma cacao]gi|508707743|gb|EOX99639.1|Gag protease polyprotein [Theobroma cacao]	XP_007043808, EOX99639	2.34E-22	230	117
Pe84I14.1	1 isoform / Incomplete 3'	-	1	2572	1450	E3 ubiquitin- ligase RF298	483	gi|1027107385|ref|XP_008234929.2|PREDICTED: putative E3 ubiquitin-protein ligase RF298 [Prunus mume]	XP_008234929	2.95E-103	511	299
Pe84I14.2		+	2	2814	552	Methyl- -binding domain-containing 4-like	183	gi|224128656|ref|XP_002320386.1|methyl-CpG-binding domain-containing family protein [Populus trichocarpa]gi|118485542|gb|ABK94623.1|unknown [Populus trichocarpa]gi|222861159|gb|EEE98701.1|methyl-CpG-binding domain-containing family protein [Populus trichocarpa]	XP_002320386, ABK94623, EEE98701	1.65E-102	183	168
Pe84I14.3		+	3	2452	564	Resistance to phytophthora 1 isoform 1 [Theobroma cacao]	187	gi|255567634|ref|XP_002524796.1|PREDICTED: uncharacterized protein LOC8285855 [Ricinus communis]gi|223535980|gb|EEF37639.1|conserved hypothetical protein [Ricinus communis]	XP_002524796, EEF37639	1.91E-52	180	130
Pe84I14.4		-	2	1559	1494	Mitochondrial transcription termination factor family isoform 1 [Theobroma cacao]	497	gi|802794177|ref|XP_012092310.1|PREDICTED: uncharacterized protein LOC105650047 [Jatropha curcas]gi|802794181|ref|XP_012092311.1|PREDICTED: uncharacterized protein LOC105650047 [Jatropha	XP_012092310, XP_012092311, KDP21517	0.0	522	450

								curcas]gi|643704453|gb|KDP21517.1|hypothetical protein JCGZ_21988 [Jatropha curcas]				
Pe84I14.5		+	3	462	198	---Na---	65	No Blast Hit				
Pe84I14.6		+	1	1053	1053	Probable galacturonosyltransferase-like 4	350	gi|743831941|ref|XP_011024154.1|PREDICTED: probable galacturonosyltransferase-like 4 [Populus euphratica]	XP_011024154	0.0	343	311
Pe84I14.7		+	1	1314	1314	BSD domain-containing 1-like	437	gi|255567648|ref|XP_002524803.1|PREDICTED: BSD domain-containing protein 1 [Ricinus communis]gi|223535987|gb|EEF37646.1|synapse-associated protein, putative [Ricinus communis]	XP_002524803, EEF37646	3.10E-123	453	299
Pe84I14.8		-	7	4510	1677	Oxidoreductase family [Populus trichocarpa]	560	gi|1000954435|ref|XP_015578229.1|PREDICTED: uncharacterized protein LOC8285863 isoform X2 [Ricinus communis]	XP_015578229	0.0	567	464
Pe84I14.9		+	1	345	345	---Na---	114	No Blast Hit				
Pe84I14.10		-	1	855	564	Class 2 F G isoform-like	187	gi|255567654|ref|XP_002524806.1|PREDICTED: uncharacterized protein LOC8285865 [Ricinus communis]gi|1000954356|ref|XP_015578204.1|PREDICTED: uncharacterized protein LOC8285865 [Ricinus communis]gi|223535990|gb|EEF37649.1|conserved hypothetical protein [Ricinus communis]	XP_002524806, XP_015578204, EEF37649	4.67E-12	204	90
Pe84I14.11	1 isoform	-	12	4586	1053	Mitochondrial ubiquitin ligase activator of NFKB 1	341	gi|743870792|ref|XP_011033674.1|PREDICTED: mitochondrial ubiquitin ligase activator of NFKB 1 [Populus euphratica]	XP_011033674	0.0	345	322
Pe84I14.12		-	5	1802	1146	GDSL esterase lipase At1g54790-like isoform X2 [Nelumbo nucifera]	381	gi|223535992|gb|EEF37651.1|Alpha-L-fucosidase 2 precursor, putative [Ricinus communis]	EEF37651	0.0	361	299
Pe84I14.13		+	12	3611	1356	Elongation factor mitochondrial	451	gi|1009147713|ref|XP_015891555.1|PREDICTED: elongation factor Tu, mitochondrial [Ziziphus jujuba]	XP_015891555	0.0	450	419
Pe84I14.14		-	5	3352	1257	Hypothetical protein JCGZ_21975	418	gi|643704440|gb|KDP21504.1|hypothetical protein JCGZ_21975 [Jatropha curcas]	KDP21504	2.35E-165	415	314
Pe84I14.15		+	4	3941	1224	BTB POZ and MATH domain-containing 2-like	407	gi|731395256|ref|XP_010652109.1|PREDICTED: BTB/POZ and MATH domain-containing protein 2-like [Vitis vinifera]gi|296086694|emb|CBI32329.3|unnamed protein product [Vitis vinifera]	XP_010652109, CBI32329	0.0	407	377
Pe84I14.16		+	3	1122	867	Heavy-metal-associated domain-containing family [Populus trichocarpa]	288	gi|566166635|ref|XP_006384446.1|heavy-metal-associated domain-containing family protein [Populus trichocarpa]gi|550341064|gb|ERP62243.1|heavy-metal-associated domain-containing family protein [Populus trichocarpa]	XP_006384446, ERP62243	5.66E-40	305	153
Pe84I14.17	2 isoforms	-	7	2713	756	Endo-1,3 1,4-beta-D-glucanase-like	251	gi|460404042|ref|XP_004247494.1|PREDICTED: endo-1,3;1,4-beta-D-glucanase-like isoform X2 [Solanum lycopersicum]	XP_004247494	5.70E-116	239	199
Pe84I14.18		+	12	8118	3981	Reduced epidermal fluorescence isoform 1 [Theobroma cacao]	1326	gi|590717753|ref|XP_007050679.1|Reduced epidermal fluorescence 4, putative isoform 1 [Theobroma cacao]gi|508702940|gb|EOX94836.1|Reduced epidermal fluorescence 4, putative isoform 1 [Theobroma cacao]	XP_007050679, EOX94836	0.0	1337	1114
Pe84I14.19		+	6	3454	771	PALE chloroplastic	308	gi|566204102|ref|XP_002320390.2|hypothetical protein POPTR_0014s13450g [Populus trichocarpa]gi|550324123|gb|EEE98705.2|hypothetical protein POPTR_0014s13450g [Populus trichocarpa]	XP_002320390, EEE98705	4.26E-142	313	264

Pe84I14.20		-	11	4352	2271	Csc1 at4g02900	756	gi|702500003|ref|XP_010038117.1|PREDICTED: CSC1-like protein At4g02900 [Eucalyptus grandis]gi|629083484|gb|KCW49929.1|hypothetical protein EUGRSUZ_K03391 [Eucalyptus grandis]	XP_010038117, KCW49929	0.0	756	658
Pe84I14.21		-	1	2972	1005	EID1-like F-box 3	334	gi|731395314|ref|XP_002276328.2|PREDICTED: EID1-like F-box protein 3 [Vitis vinifera]	XP_002276328	1.81E-135	243	219
Pe84I14.22	2 isoforms	+	11	7979	4275	Tudor PWWP MBT domain-containing isoform 2 [Theobroma cacao]	1424	gi|802793215|ref|XP_012092286.1|PREDICTED: HUA2-like protein 3 isoform X1 [Jatropha curcas]gi|643704430|gb|KDP21494.1|hypothetical protein JCGZ_21965 [Jatropha curcas]	XP_012092286, KDP21494	0.0	1458	962
Pe84I14.23	1 isoform	+	5	4100	513	Probable glutathione peroxidase 4	170	gi|719999934|ref|XP_010255873.1|PREDICTED: probable glutathione peroxidase 4 [Nelumbo nucifera]gi|719999937|ref|XP_010255874.1|PREDICTED: probable glutathione peroxidase 4 [Nelumbo nucifera]	XP_010255873, XP_010255874	2.63E-98	169	154
Pe84I14.24	1 isoform	-	8	4163	1824	Stress response NST1 [Glycine max]	607	gi|223527136|gb|EEF29311.1|conserved hypothetical protein [Ricinus communis]	EEF29311	4.07E-140	633	403
Pe84I14.25	Incomplete 3'	+	2	1155	879	Plant organelle RNA recognition domain [Medicago truncatula]	293	gi|802793199|ref|XP_012092282.1|PREDICTED: protein ROOT PRIMORDIUM DEFECTIVE 1 [Jatropha curcas]	XP_012092282	3.19E-140	256	227
Pe84K8.1	Incomplete 3'	-	1	162	162	Hypothetical protein CISIN_1g026254mg	54	gi|641843916|gb|KDO62812.1|hypothetical protein CISIN_1g026254mg [Citrus sinensis]	KDO62812	1.98E-19	54	49
Pe84K8.2	3 isoforms	-	12	6278	2295	Poly(A) polymerase 1 isoform 1 [Theobroma cacao]	764	gi|743930739|ref|XP_011009627.1|PREDICTED: nuclear poly(A) polymerase 1 [Populus euphratica]	XP_011009627	0.0	780	655
Pe84K8.3	1 isoform	-	8	12356	1137	Uncharacterized PKHD-type hydroxylase At1g22950-like	378	gi|802617592|ref|XP_012075419.1|PREDICTED: uncharacterized PKHD-type hydroxylase At1g22950 isoform X1 [Jatropha curcas]gi|643726450|gb|KDP35157.1|hypothetical protein JCGZ_10691 [Jatropha curcas]	XP_012075419, KDP35157	0.0	378	339
Pe84K8.4		+	1	3108	1608	T-complex 1 subunit delta-like	535	gi|802536731|ref|XP_012080304.1|PREDICTED: T-complex protein 1 subunit delta [Jatropha curcas]gi|643741570|gb|KDP46998.1|hypothetical protein JCGZ_02434 [Jatropha curcas]	XP_012080304, KDP46998	0.0	535	527
Pe84K8.5		-	6	2236	1632	Glycosyltransferase family 61 [Theobroma cacao]	543	gi|566196832|ref|XP_002317893.2|hypothetical protein POPTR_0012s04840g [Populus trichocarpa]gi|550326399|gb|EEE96113.2|hypothetical protein POPTR_0012s04840g [Populus trichocarpa]	XP_002317893, EEE96113	0.0	557	433
Pe84K8.6		-	3	1653	1392	Glycosyltransferase family 61 [Theobroma cacao]	463	gi|743913191|ref|XP_011000494.1|PREDICTED: uncharacterized protein LOC105108049 isoform X3 [Populus euphratica]	XP_011000494	0.0	524	375
Pe84K8.7		+	1	348	348	MATE efflux family [Theobroma cacao]	115	gi|590665176|ref|XP_007036669.1|MATE efflux family protein [Theobroma cacao]gi|508773914|gb|EOY21170.1|MATE efflux family protein [Theobroma cacao]	XP_007036669, EOY21170	5.48E-24	101	78
Pe84K8.8	1 isoform	+	7	1904	657	DETOXIFICATION 16-like	218	gi|743890598|ref|XP_011039093.1|PREDICTED: MATE efflux family protein 5 isoform X2 [Populus euphratica]	XP_011039093	6.73E-120	218	197
Pe84K8.9	1 isoform	+	7	2574	486	Clathrin assembly AP19 homolog [Arabidopsis thaliana]	161	gi|567859436|ref|XP_006422372.1|hypothetical protein CICLE_v10029448mg [Citrus clementina]gi|557524306|gb|ESR35612.1|hypothetical protein CICLE_v10029448mg [Citrus clementina]gi|641849563|gb|KDO68438.1|hypothetical protein CISIN_1g031366mg [Citrus sinensis]gi|641849564|gb|KDO68439.1|hypothetical protein CISIN_1g031366mg [Citrus sinensis]	XP_006422372, ESR35612, KDO68438, KDO68439	1.29E-101	161	156

Pe84K8.10	1 isoform	-	6	2968	1104	Cyclin-dependent kinase D-3-like	367	gi|1000940586|ref|XP_002532521.2|PREDICTED: cyclin-dependent kinase D-3 [Ricinus communis]gi|1000940588|ref|XP_015582936.1|PREDICTED: cyclin-dependent kinase D-3 [Ricinus communis]	XP_002532521, XP_015582936	0.0	370	304
Pe84K8.11	1 isoform	-	4	1749	834	Microbial collagenase	277	gi|224118714|ref|XP_002317888.1|hypothetical protein POPTR_0012s04760g [Populus trichocarpa]gi|222858561|gb|EEE96108.1|hypothetical protein POPTR_0012s04760g [Populus trichocarpa]	XP_002317888, EEE96108	3.27E-117	278	215
Pe84K8.12	3 isoforms	-	18	9022	1530	Eukaryotic peptide chain release factor GTP-binding subunit ERF3A-like isoform X1	509	gi|802599721|ref|XP_012072559.1|PREDICTED: eukaryotic peptide chain release factor GTP-binding subunit ERF3A isoform X3 [Jatropha curcas]	XP_012072559	0.0	510	464
Pe84K8.13	2 isoforms	-	6	3840	768	Pre-mRNA-splicing factor SPF27 homolog	255	gi|224135477|ref|XP_002322083.1|hypothetical protein POPTR_0015s04220g [Populus trichocarpa]gi|222869079|gb|EEF06210.1|hypothetical protein POPTR_0015s04220g [Populus trichocarpa]	XP_002322083, EEF06210	2.89E-141	254	230
Pe84K8.14		-	7	5310	1101	Peroxin 3 isoform 1 [Theobroma cacao]	366	gi|1029009260|ref|XP_016668512.1|PREDICTED: peroxisome biogenesis protein 3-2-like isoform X1 [Gossypium hirsutum]gi|1029009262|ref|XP_016668513.1|PREDICTED: peroxisome biogenesis protein 3-2-like isoform X2 [Gossypium hirsutum]gi|728833394|gb|KHG12837.1|Peroxisome biogenesis 3-2 -like protein [Gossypium arboreum]	XP_016668512, XP_016668513, KHG12837	1.89E-175	368	306
Pe84K8.15		+	2	2066	987	Guanine nucleotide-binding subunit beta	328	gi|802599689|ref|XP_012072542.1|PREDICTED: guanine nucleotide-binding protein subunit beta-like protein [Jatropha curcas]gi|643730422|gb|KDP37901.1|hypothetical protein JCGZ_05340 [Jatropha curcas]	XP_012072542, KDP37901	0.0	329	316
Pe84K8.16	Incomplete 5'	-	5	1560	1123	Snrna-activating complex subunit 4 isoform X1	373	gi|743913154|ref|XP_011000476.1|PREDICTED: uncharacterized protein LOC105108037 isoform X1 [Populus euphratica]	XP_011000476	3.34E-75	257	176
Pe84M6.1		+	1	862	729	Galactoside 2-alpha-L-fucosyltransferase-like [Populus euphratica]	252	gi|255569183|ref|XP_002525560.1|PREDICTED: galactoside 2-alpha-L-fucosyltransferase [Ricinus communis]gi|223535139|gb|EEF36819.1|Galactoside 2-alpha-L-fucosyltransferase, putative [Ricinus communis]	XP_002525560, EEF36819	1.01E-115	251	205
Pe84M6.2		+	5	1341	603	rRNA-processing fcf2-like	200	gi|224102645|ref|XP_002312762.1|hypothetical protein POPTR_0008s21130g [Populus trichocarpa]gi|118488000|gb|ABK95821.1|unknown [Populus trichocarpa]gi|222852582|gb|EEE90129.1|hypothetical protein POPTR_0008s21130g [Populus trichocarpa]	XP_002312762, ABK95821, EEE90129	1.61E-97	203	172
Pe84M6.3		+	1	1521	1521	PREDICTED: uncharacterized protein LOC103343557	506	gi|645281008|ref|XP_008245436.1|PREDICTED: uncharacterized protein LOC103343557 [Prunus mume]	XP_008245436	6.21E-54	512	238
Pe84M6.4		+	2	1332	1218	Amaranthin-like lectin	405	gi|225465427|ref|XP_002264911.1|PREDICTED: uncharacterized protein LOC100263724 [Vitis vinifera]	XP_002264911	1.87E-30	152	94
Pe84M6.5		+	13	3026	1578	Beta-glucosidase 12-like	525	gi|601587996|gb|AHN85654.1|glycoside hydrolase family 1 [Drypetes roxburghii]	AHN85654	0.0	507	375

Pe84M6.6		-	1	1302	1302	Retrovirus-related Pol poly from transposon opus	433	gi|734399979|gb|KHN31113.1|hypothetical protein glysoja_046590, partial [Glycine soja]	KHN31113	2.29E-11	287	127
Pe84M6.7		-	3	2390	1431	PREDICTED: uncharacterized protein LOC107177920	476	gi|985458955|ref|XP_015387963.1|PREDICTED : uncharacterized protein LOC107177920 [Citrus sinensis]	XP_015387963	1.49E-85	471	254
Pe84M6.8		-	1	375	375	---Na---	124	No Blast Hit				
Pe84M6.9		+	1	264	264	---Na---	87	No Blast Hit				
Pe84M6.10		+	1	378	378	Retrovirus-related Pol poly from transposon 297 [Morus notabilis]	125	gi|703100725|ref|XP_010096995.1|hypothetical protein L484_024918 [Morus notabilis]gi|587877587|gb|EXB66622.1|hypothetical protein L484_024918 [Morus notabilis]	XP_010096995, EXB66622	1.47E-11	122	66
Pe84M6.11		-	2	446	366	---Na---	121	No Blast Hit				
Pe84M6.12		+	1	357	357	---Na---	118	No Blast Hit				
Pe84M6.13	Incomplete 3'	+	1	185	185	---Na---	61	No Blast Hit				
Pe84M18.1		+	7	3199	924	Pre-mRNA-splicing factor ATP-dependent RNA helicase DHX16 [Jatropha curcas]	307	gi|848910813|ref|XP_012853960.1|PREDICTED : putative pre-mRNA-splicing factor ATP-dependent RNA helicase DHX16 [Erythranthe guttata]	XP_012853960	0.0	305	299
Pe84M18.2		+	1	393	393	ATP-binding cassette sub-family D member [Theobroma cacao]	130	gi|643721057|gb|KDP31321.1|hypothetical protein JCGZ_11697 [Jatropha curcas]	KDP31321	1.03E-18	132	80
Pe84M18.3		-	7	5130	2115	Diacylglycerol kinase 1-like [Gossypium hirsutum]	704	gi|224121474|ref|XP_002318591.1|Diacylglycerol kinase 1 family protein [Populus trichocarpa]gi|222859264|gb|EEE96811.1|Diacylglycerol kinase 1 family protein [Populus trichocarpa]	XP_002318591, EEE96811	0.0	728	630
Pe84M18.4	1 isoform	+	8	2879	900	PREDICTED: uncharacterized protein LOC8284444 isoform X2 [Ricinus communis]	299	gi|255547223|ref|XP_002514669.1|PREDICTED : uncharacterized protein LOC8284444 isoform X2 [Ricinus communis]gi|223546273|gb|EEF47775.1|conserved hypothetical protein [Ricinus communis]	XP_002514669, EEF47775	5.14E-123	300	228
Pe84M18.5		-	7	8124	4356	Dentin sialophospho [Populus trichocarpa]	1451	gi|566197127|ref|XP_006376803.1|hypothetical protein POPTR_0012s06820g [Populus trichocarpa]gi|550326529|gb|ERP54600.1|hypothetical protein POPTR_0012s06820g [Populus trichocarpa]	XP_006376803, ERP54600	0.0	1417	889
Pe84M18.6		+	1	2344	1407	RING-H2 finger ATL43	468	gi|743926244|ref|XP_011007280.1|PREDICTED : RING-H2 finger protein ATL43 [Populus euphratica]	XP_011007280	8.98E-125	429	280
Pe84M18.7		+	3	2616	1542	Flavonoid 3 -monooxygenase	513	gi|802653390|ref|XP_012080364.1|PREDICTED : flavonoid 3'-monooxygenase [Jatropha curcas]gi|643721070|gb|KDP31334.1|hypothetical protein JCGZ_11710 [Jatropha curcas]	XP_012080364, KDP31334	0.0	508	457
Pe84M18.8		-	2	703	642	Ribonuclease H At1g65750	213	gi|595809859|ref|XP_007202982.1|hypothetical protein PRUPE_ppa018691mg, partial [Prunus persica]gi|462398513|gb|EMJ04181.1|hypothetical protein PRUPE_ppa018691mg, partial [Prunus persica]	XP_007202982, EMJ04181	8.56E-11	86	51
Pe84M18.9		-	1	381	381	---Na---	126	No Blast Hit				
Pe84M18.10		-	3	1406	681	---Na---	226	No Blast Hit				
Pe84M18.11		-	3	1764	1245	LINE-1 reverse transcriptase isogeny	414	gi|1021574760|ref|XP_016178547.1|PREDICTED: uncharacterized protein LOC107621000 [Arachis ipaensis]	XP_016178547	6.40E-75	410	216

Pe84M18.12		-	1	321	321	---Na---	106	No Blast Hit				
Pe84M18.13		-	2	1398	798	---Na---	265	No Blast Hit				
Pe84M18.14	3 isoforms	+	6	2644	924	Transmembrane adipocyte-associated 1 homolog	307	gi|802653394|ref|XP_012080365.1|PREDICTED: transmembrane protein adipocyte-associated 1 homolog isoform X1 [Jatropha curcas]	XP_012080365	4.18E-133	298	234
Pe84M18.15		+	4	4297	597	Plasmodesmata callose-binding protein 3	198	gi|743939801|ref|XP_011014355.1|PREDICTED: LOW QUALITY PROTEIN: PLASMODESMATA CALLOSE-BINDING PROTEIN 3-like [Populus euphratica]	XP_011014355	1.69E-65	208	148
Pe84M18.16		+	10	2996	1089	ATP synthase subunit mitochondrial	362	gi|802653454|ref|XP_012080377.1|PREDICTED: ATP synthase subunit gamma, mitochondrial [Jatropha curcas]gi|643721082|gb|KDP31346.1|hypothetical protein JCGZ_11722 [Jatropha curcas]	XP_012080377, KDP31346	0.0	362	297
Pe84M18.17	3 isoforms	+	7	4212	894	Phosphoserine chloroplastic-like [Gossypium hirsutum]	297	gi|802653458|ref|XP_012080378.1|PREDICTED: phosphoserine phosphatase, chloroplastic [Jatropha curcas]gi|802653462|ref|XP_012080379.1|PREDICTED: phosphoserine phosphatase, chloroplastic [Jatropha curcas]gi|802653465|ref|XP_012080380.1|PREDICTED: phosphoserine phosphatase, chloroplastic [Jatropha curcas]gi|643721083|gb|KDP31347.1|hypothetical protein JCGZ_11723 [Jatropha curcas]	XP_012080378, XP_012080379, XP_012080380, KDP31347	1.45E-165	297	259
Pe84M18.18		+	3	3368	2067	Formate hydrogenlyase subunit 5	688	gi|1000976230|ref|XP_015572072.1|PREDICTED: uncharacterized protein LOC8284431 [Ricinus communis]	XP_015572072	0.0	704	484
Pe84M18.19		-	6	2303	996	Glycine-rich RNA-binding mitochondrial-like	282	gi|743941320|ref|XP_011015146.1|PREDICTED: glycine-rich RNA-binding protein 4, mitochondrial-like [Populus euphratica]	XP_011015146	1.12E-56	285	187
Pe84M18.20		-	1	2369	1842	Exocyst complex component EXO70B1	625	gi|743924887|ref|XP_011006574.1|PREDICTED: exocyst complex component EXO70B1 [Populus euphratica]	XP_011006574	0.0	638	480
Pe84M18.21		+	2	1134	1017	Cell wall RBR3 [Fragaria vesca vesca]	338	gi|566197003|ref|XP_002318571.2|hypothetical protein POPTR_0012s05880g [Populus trichocarpa]gi|550326470|gb|EEE96791.2|hypothetical protein POPTR_0012s05880g [Populus trichocarpa]	XP_002318571, EEE96791	7.81E-49	271	163
Pe84M18.22	1 isoform	+	1	2419	393	PREDICTED: uncharacterized protein LOC105640640 [Jatropha curcas]	130	gi|802653529|ref|XP_012080395.1|PREDICTED: uncharacterized protein LOC105640640 [Jatropha curcas]gi|802653533|ref|XP_012080396.1|PREDICTED: uncharacterized protein LOC105640640 [Jatropha curcas]gi|643721096|gb|KDP31360.1|hypothetical protein JCGZ_11736 [Jatropha curcas]	XP_012080395, XP_012080396, KDP31360	9.73E-69	130	116
Pe84M23.1		+	2	2726	1740	LRR receptor-like serine threonine- kinase RCH1	579	gi|566156695|ref|XP_002300934.2|hypothetical protein POPTR_0002s07170g [Populus trichocarpa]gi|550344464|gb|EEE80207.2|hypothetical protein POPTR_0002s07170g [Populus trichocarpa]	XP_002300934, EEE80207	0.0	579	516
Pe84M23.2		-	5	2729	1875	Endoglucanase 25-like	517	gi|743911982|ref|XP_010999860.1|PREDICTED: endoglucanase 9-like [Populus euphratica]	XP_010999860	0.0	514	420
Pe84M23.3		-	2	305	177	---Na---	58	No Blast Hit				

Pe84M23.4-5		-	2	3035	3018	Pentatricopeptide repeat-containing mitochondrial	775	gi|743911907|ref|XP_010999820.1|PREDICTED: pentatricopeptide repeat-containing protein At3g09040, mitochondrial [Populus euphratica]gi|743911909|ref|XP_010999821.1|PREDICTED: pentatricopeptide repeat-containing protein At3g09040, mitochondrial [Populus euphratica]	XP_010999820, XP_010999821	0.0	770	668
Pe84M23.6		-	4	4163	1941	UDP-glucuronate:xylan alpha-glucuronosyltransferase 3	646	gi|802559241|ref|XP_012066076.1|PREDICTED: putative UDP-glucuronate:xylan alpha-glucuronosyltransferase 3 isoform X2 [Jatropha curcas]gi|643736770|gb|KDP43041.1|hypothetical protein JCGZ_25227 [Jatropha curcas]	XP_012066076, KDP43041	0.0	642	589
Pe84M23.7		+	7	3290	963	CMP-sialic acid transporter 5	320	gi|590707261|ref|XP_007047955.1|Nucleotide-sugar transporter family protein [Theobroma cacao]gi|508700216|gb|EOX92112.1|Nucleotide-sugar transporter family protein [Theobroma cacao]	XP_007047955, EOX92112	0.0	313	294
Pe84M23.8	1 isoform	+	1	2160	519	Ribosomal L23 [Populus trichocarpa]	172	gi|223529144|gb|EEF31123.1|RNA binding protein, putative [Ricinus communis]	EEF31123	3.20E-71	175	139
Pe84M23.9		+	13	6571	1707	Vacuolar sorting-associated 45 homolog	568	gi|590600238|ref|XP_007019403.1|Vacuolar protein sorting 45 [Theobroma cacao]gi|508724731|gb|EOY16628.1|Vacuolar protein sorting 45 [Theobroma cacao]	XP_007019403, EOY16628	0.0	568	546
Pe84M23.10		-	1	907	312	Histone superfamily isoform partial [Theobroma cacao]	103	gi|195617694|gb|ACG30677.1|histone H4 [Zea mays]	ACG30677	4.63E-61	103	103
Pe84M23.11		+	8	2795	1887	Polygalacturonase [Ricinus communis]	628	gi|566211237|ref|XP_006372692.1|hypothetical protein POPTR_0017s03930g [Populus trichocarpa]gi|550319321|gb|ERP50489.1|hypothetical protein POPTR_0017s03930g [Populus trichocarpa]	XP_006372692, ERP50489	0.0	384	312
Pe84M23.12	2 isoforms	+	10	4017	1443	Serine carboxypeptidase-like 42	480	gi|224062940|ref|XP_002300939.1|serine carboxypeptidase S10 family protein [Populus trichocarpa]gi|222842665|gb|EEE80212.1|serine carboxypeptidase S10 family protein [Populus trichocarpa]	XP_002300939, EEE80212	0.0	480	430
Pe84M23.13		+	3	2246	1248	Trichome berefringence-like 7	421	gi|743845056|ref|XP_011027378.1|PREDICTED: protein trichome berefringence-like 7 [Populus euphratica]	XP_011027378	0.0	410	354
Pe84M23.14		+	1	1053	795	Proline-rich receptor kinase PERK2	264	gi|731430113|ref|XP_010664882.1|PREDICTED: uncharacterized protein LOC100259240 [Vitis vinifera]	XP_010664882	1.37E-49	171	121
Pe84M23.15		-	1	552	552	Cell wall vacuolar inhibitor of fructosidase 2-like	183	gi|645219391|ref|XP_008235482.1|PREDICTED: cell wall / vacuolar inhibitor of fructosidase 2-like [Prunus mume]	XP_008235482	4.99E-31	176	107
Pe84M23.16		-	10	3716	1140	Alcohol dehydrogenase	379	gi|802559257|ref|XP_012066085.1|PREDICTED: alcohol dehydrogenase 1 [Jatropha curcas]gi|643736779|gb|KDP43050.1|hypothetical protein JCGZ_25236 [Jatropha curcas]	XP_012066085, KDP43050	0.0	377	363
Pe84M23.17	1 isoform	-	7	3858	1617	Probable auxin efflux carrier component 6	538	gi|1000951987|ref|XP_002526168.2|PREDICTED: probable auxin efflux carrier component 6 [Ricinus communis]	XP_002526168	0.0	566	493
Pe84M23.18		-	5	2252	834	Psbp domain-containing chloroplastic	277	gi|255570434|ref|XP_002526176.1|PREDICTED: psbP domain-containing protein 4, chloroplastic isoform X2 [Ricinus communis]gi|223534553|gb|EEF36252.1|Thylakoid lumenal 29.8 kDa protein, chloroplast precursor, putative [Ricinus communis]	XP_002526176, EEF36252	3.87E-149	277	239

Pe84M23.19		-	8	2664	1395	Exostosin family [Populus trichocarpa]	464	gi|566172442|ref|XP_002307477.2|exostosin family protein [Populus trichocarpa]gi|550339425|gb|EEE94473.2|exostosin family protein [Populus trichocarpa]	XP_002307477, EEE94473	0.0	464	406
Pe84M23.20		-	5	2537	654	Mediator of RNA polymerase II transcription subunit 18 [Arachis duranensis]	217	gi|802673444|ref|XP_012081604.1|PREDICTED: mediator of RNA polymerase II transcription subunit 18 [Jatropha curcas]gi|643718513|gb|KDP29707.1|hypothetical protein JCGZ_18642 [Jatropha curcas]	XP_012081604, KDP29707	1.06E-134	217	209
Pe84M23.21		+	3	780	303	Heavy metal-associated isoprenylated plant 26-like	104	gi|255579045|ref|XP_002530373.1|PREDICTED: heavy metal-associated isoprenylated plant protein 26 [Ricinus communis]gi|223530090|gb|EEF32006.1|copper ion binding protein, putative [Ricinus communis]	XP_002530373, EEF32006	7.41E-36	94	79
Pe84M23.22	2 isoforms	+	2	1837	372	50S ribosomal L20 [Ricinus communis]	123	gi|698525757|ref|XP_009759705.1|PREDICTED: 50S ribosomal protein L20, chloroplastic [Nicotiana sylvestris]gi|1025245830|ref|XP_016487793.1|PREDICTED: 50S ribosomal protein L20-like [Nicotiana tabacum]	XP_009759705, XP_016487793	5.75E-37	105	80
Pe84M23.23	2 isoforms	-	1	1711	324	30S ribosomal chloroplastic [Vitis vinifera]	107	gi|1000959754|ref|XP_002521665.2|PREDICTED: 30S ribosomal protein S17 [Ricinus communis]	XP_002521665	2.51E-50	100	94
Pe84M23.24	1 isoform	+	6	2553	738	Bidirectional sugar transporter SWEET1	245	gi|255570438|ref|XP_002526178.1|PREDICTED: bidirectional sugar transporter SWEET1 [Ricinus communis]gi|223534555|gb|EEF36254.1|conserved hypothetical protein [Ricinus communis]	XP_002526178, EEF36254	5.44E-143	248	226
Pe84M23.25-26		+	8	8652	2607	Transcriptional corepressor SEUSS	527	gi|1000963890|ref|XP_015575385.1|PREDICTED: transcriptional corepressor SEUSS [Ricinus communis]	XP_015575385	0.0	511	469
Pe84M23.27	Incomplete 3'	+	5	1738	1061	Mitogen-activated kinase kinase kinase YODA-like isoform X1	353	gi|802559289|ref|XP_012066102.1|PREDICTED: mitogen-activated protein kinase kinase kinase YODA-like [Jatropha curcas]	XP_012066102	1.37E-62	367	210
Pe85B19.1	Incomplete 5'	+	4	906	584	Probable phosphatase 2C 72	193	gi|566214219|ref|XP_002324150.2|hypothetical protein POPTR_0018s04620g [Populus trichocarpa]gi|550318039|gb|EEF02715.2|hypothetical protein POPTR_0018s04620g [Populus trichocarpa]	XP_002324150, EEF02715	2.02E-104	193	174
Pe85B19.2		+	9	3097	1479	Vacuolar processing enzyme	492	gi|743813514|ref|XP_011019532.1|PREDICTED: vacuolar-processing enzyme isoform X1 [Populus euphratica]	XP_011019532	0.0	489	449
Pe85B19.3		-	2	4258	2568	Sarcoplasmic reticulum histidine-rich calcium-binding [Prunus mume]	855	gi|743813530|ref|XP_011019534.1|PREDICTED: serine/arginine repetitive matrix protein 2 [Populus euphratica]	XP_011019534	1.92E-113	900	475
Pe85B19.4		-	1	543	360	Serine arginine repetitive matrix 2	119	gi|255550852|ref|XP_002516474.1|PREDICTED: protein starmaker [Ricinus communis]gi|223544294|gb|EEF45815.1|conserved hypothetical protein [Ricinus communis]	XP_002516474, EEF45815	8.81E-54	119	107
Pe85B19.5		-	1	1321	1146	Gag protease poly [Theobroma cacao]	381	gi|590728434|ref|XP_007099662.1|Gag protease polyprotein-like protein [Theobroma cacao]gi|508728474|gb|EOY20371.1|Gag protease polyprotein-like protein [Theobroma cacao]	XP_007099662, EOY20371	7.82E-39	341	164
Pe85B19.6		+	1	663	183	---Na---	60	No Blast Hit				
Pe85B19.7		+	1	540	540	---Na---	179	No Blast Hit				

Pe85B19.8		-	1	372	372	---Na---	123	No Blast Hit				
Pe85B19.9		+	4	6037	900	Myb D isoform X2 [Gossypium hirsutum]	299	gi|950975990|ref|XP_014501199.1|PREDICTED: uncharacterized protein LOC106762032 [Vigna radiata var. radiata]gi|950975993|ref|XP_014501200.1|PREDICTED: uncharacterized protein LOC106762032 [Vigna radiata var. radiata]gi|950975997|ref|XP_014501201.1|PREDICTED: uncharacterized protein LOC106762032 [Vigna radiata var. radiata]gi|950976003|ref|XP_014501202.1|PREDICTED: uncharacterized protein LOC106762032 [Vigna radiata var. radiata]	XP_014501199, XP_014501200, XP_014501201, XP_014501202	2.30E-109	318	231
Pe85B19.10		-	1	784	195	Arabinogalactan 15 [Arabidopsis thaliana]	64	gi|763787742|gb|KJB54738.1|hypothetical protein B456_009G047100, partial [Gossypium raimondii]	KJB54738	1.51E-05	64	44
Pe85B19.11		-	2	3452	1152	Core-2 I-branching beta-1,6-N-acetylglucosaminyltransferase family [Theobroma cacao]	383	gi|1000972467|ref|XP_015573130.1|PREDICTED: uncharacterized protein LOC8275630 [Ricinus communis]	XP_015573130	0.0	384	354
Pe85B19.12	1 isoform	-	12	5736	1821	Clathrin interactor EPSIN 1	606	gi|802632672|ref|XP_012077334.1|PREDICTED: clathrin interactor EPSIN 1 [Jatropha curcas]gi|643724933|gb|KDP34134.1|hypothetical protein JCGZ_07705 [Jatropha curcas]	XP_012077334, KDP34134	0.0	616	448
Pe85B19.13	3 isoforms	-	3	1947	933	Transcription factor RAX2-like	310	gi|743813585|ref|XP_011019548.1|PREDICTED: transcription factor RAX2-like [Populus euphratica]gi|743942946|ref|XP_011015974.1|PREDICTED: transcription factor RAX2-like [Populus euphratica]	XP_011019548, XP_011015974	8.38E-114	325	235
Pe85B19.14		-	1	2045	1092	Zinc finger CCCH domain-containing 23-like	363	gi|802632676|ref|XP_012077337.1|PREDICTED: zinc finger CCCH domain-containing protein 20 [Jatropha curcas]gi|643724936|gb|KDP34137.1|hypothetical protein JCGZ_07708 [Jatropha curcas]	XP_012077337, KDP34137	7.72E-164	382	306
Pe85B19.15	1 isoform	+	7	3787	1665	Sigma factor	554	gi|224088962|ref|XP_002308586.1|hypothetical protein POPTR_0006s25090g [Populus trichocarpa]gi|222854562|gb|EEE92109.1|hypothetical protein POPTR_0006s25090g [Populus trichocarpa]	XP_002308586, EEE92109	0.0	561	411
Pe85B19.16		+	18	10115	3228	PREDICTED: uncharacterized protein LOC105122249	1075	gi|743813602|ref|XP_011019552.1|PREDICTED: uncharacterized protein LOC105122249 [Populus euphratica]	XP_011019552	0.0	1069	915
Pe85B19.17		+	4	1987	774	1-aminocyclopropane-1-carboxylate deaminase	257	gi|743813602|ref|XP_011019552.1|PREDICTED: uncharacterized protein LOC105122249 [Populus euphratica]	XP_011019552	8.45E-94	259	207
Pe85B19.18		-	5	1028	426	Glutamyl-tRNA(Gln) amidotransferase subunit chloroplastic mitochondrial	141	gi|743934466|ref|XP_011011569.1|PREDICTED: glutamyl-tRNA(Gln) amidotransferase subunit C, chloroplastic/mitochondrial isoform X1 [Populus euphratica]	XP_011011569	1.81E-54	145	111
Pe85B19.19		-	11	5555	2568	Tetratricopeptide repeat-like superfamily isoform 1 [Theobroma cacao]	855	gi|643724941|gb|KDP34142.1|hypothetical protein JCGZ_07713 [Jatropha curcas]	KDP34142	0.0	866	709
Pe85H4.1	Incomplete 3'	-	4	1422	1103	Pectate lyase 22 precursor family [Populus trichocarpa]	367	gi|743923030|ref|XP_011005597.1|PREDICTED: probable pectate lyase 18 [Populus euphratica]	XP_011005597	0.0	349	338
Pe85H4.2		+	5	4199	2793	STICHEL-like 2	930	gi|802649228|ref|XP_012079933.1|PREDICTED	XP_012079933,	0.0	985	714

								: protein STICHEL-like 2 [Jatropha curcas]gi|802649233|ref|XP_012079934.1|PREDICTED: protein STICHEL-like 2 [Jatropha curcas]	XP_012079934			
Pe85H4.3		+	6	2513	885	Serine threonine- kinase rio1-like	294	gi|802647461|ref|XP_012079836.1|PREDICTED: serine/threonine-protein kinase rio1-like [Jatropha curcas]gi|643720653|gb|KDP30917.1|hypothetical protein JCGZ_11293 [Jatropha curcas]	XP_012079836, KDP30917	6.09E-130	209	199
Pe85H4.4		+	2	551	453	Serine threonine- kinase rio1-like	150	gi|703119126|ref|XP_010101787.1|Serine/threonine-protein kinase RIO1 [Morus notabilis]gi|587901367|gb|EXB89642.1|Serine/threonine-protein kinase RIO1 [Morus notabilis]	XP_010101787, EXB89642	2.60E-13	129	91
Pe85H4.5		+	3	3108	2160	MA3 domain-containing family [Populus trichocarpa]	719	gi|568846192|ref|XP_006476941.1|PREDICTED: uncharacterized protein LOC102613560 [Citrus sinensis]	XP_006476941	0.0	715	658
Pe85H4.6		+	7	4155	1683	Zinc finger CCCH domain-containing 43	560	gi|802648882|ref|XP_012079920.1|PREDICTED: zinc finger CCCH domain-containing protein 67 [Jatropha curcas]gi|643720720|gb|KDP30984.1|hypothetical protein JCGZ_11360 [Jatropha curcas]	XP_012079920, KDP30984	0.0	464	328
Pe85H4.7		-	51	22481	5706	Callose synthase 10	1901	gi|1000984791|ref|XP_015579596.1|PREDICTED: callose synthase 10 [Ricinus communis]gi|1000984793|ref|XP_015579600.1|PREDICTED: callose synthase 10 [Ricinus communis]	XP_015579596, XP_015579600	0.0	1909	1766
Pe85H4.8		+	2	1214	1101	GDSL esterase lipase At3g48460	366	gi|743831693|ref|XP_011024095.1|PREDICTED: GDSL esterase/lipase At3g48460 [Populus euphratica]	XP_011024095	0.0	343	293
Pe85H4.9		-	7	1562	843	Probable ubiquitin-like-specific protease 2A isoform X1	280	gi|743831647|ref|XP_011024082.1|PREDICTED: probable ubiquitin-like-specific protease 2A [Populus euphratica]gi|743831649|ref|XP_011024083.1|PREDICTED: probable ubiquitin-like-specific protease 2A [Populus euphratica]	XP_011024082, XP_011024083	6.44E-116	288	218
Pe85H4.10		+	8	2870	1161	26S proteasome non-atpase regulatory subunit 6 homolog	386	gi|802648411|ref|XP_012079902.1|PREDICTED: 26S proteasome non-ATPase regulatory subunit 6 homolog [Jatropha curcas]gi|643720707|gb|KDP30971.1|hypothetical protein JCGZ_11347 [Jatropha curcas]	XP_012079902, KDP30971	0.0	386	377
Pe85H4.11		-	7	5007	1665	Binding isoform 1 [Theobroma cacao]	554	gi|1000947413|ref|XP_015580612.1|PREDICTED: uncharacterized protein At5g08430 isoform X1 [Ricinus communis]	XP_015580612	0.0	560	378
Pe85H4.12	2 isoforms	+	12	4977	1446	Uncharacterized aarf domain-containing kinase 1-like isoform X1 [Populus euphratica]	481	gi|566206897|ref|XP_006374554.1|hypothetical protein POPTR_0015s10290g [Populus trichocarpa]gi|550322426|gb|ERP52351.1|hypothetical protein POPTR_0015s10290g [Populus trichocarpa]	XP_006374554, ERP52351	0.0	481	458
Pe85H4.13		-	2	489	300	Nucleoside diphosphate kinase chloroplastic-like	99	gi|566197644|ref|XP_002318702.2|hypothetical protein POPTR_0012s09540g [Populus trichocarpa]gi|550326750|gb|EEE96922.2|hypothetical protein POPTR_0012s09540g [Populus trichocarpa]	XP_002318702, EEE96922	2.72E-02	129	56
Pe85H4.14		-	1	588	354	DELLA RGL1-like	117	gi|802648221|ref|XP_012079895.1|PREDICTED: DELLA protein RGL1-like [Jatropha curcas]gi|643720703|gb|KDP30967.1|hypothetical protein JCGZ_11343 [Jatropha curcas]gi|1008986611|gb|AMR43783.1|GRAS32	XP_012079895, KDP30967, AMR43783	1.24E-75	117	115

								protein [Jatropha curcas]				
Pe85I9.1	Incomplete 3'	-	1	647	647	PLASTID MOVEMENT IMPAIRED 1-RELATED 1 [Prunus mume]	215	gi|743812916|ref|XP_011019387.1|PREDICTED: uncharacterized protein LOC105122155 [Populus euphratica]	XP_011019387	1.34E-90	214	177
Pe85I9.2	2 isoforms	-	4	3940	1629	Abscisic insensitive 1B [Populus tremula]	542	gi|566214208|ref|XP_002324741.2|hypothetical protein POPTR_0018s04570g [Populus trichocarpa]gi|550318034|gb|EEF03306.2|hypothetical protein POPTR_0018s04570g [Populus trichocarpa]	XP_002324741, EEF03306	0.0	552	453
Pe85I9.3		-	12	8578	1515	Serine threonine- kinase 38-like isoform X2	504	gi|1000973305|ref|XP_015572964.1|PREDICTED: serine/threonine-protein kinase 38-like isoform X2 [Ricinus communis]	XP_015572964	0.0	493	472
Pe85I9.4	3 isoforms	+	6	2761	771	U2 small nuclear ribonucleo B -like isoform X2	256	gi|743812997|ref|XP_011019404.1|PREDICTED: uncharacterized protein LOC105122162 [Populus euphratica]	XP_011019404	1.20E-84	276	178
Pe85I9.5		-	1	2711	1524	Nuclease HARBI1	507	gi|224092160|ref|XP_002309488.1|hypothetical protein POPTR_0006s24160g [Populus trichocarpa]gi|222855464|gb|EEE93011.1|hypothetical protein POPTR_0006s24160g [Populus trichocarpa]	XP_002309488, EEE93011	0.0	506	444
Pe85I9.6		-	11	5390	1359	Peptidyl-prolyl cis-trans isomerase CYP57	452	gi|659103904|ref|XP_008452743.1|PREDICTED: peptidyl-prolyl cis-trans isomerase CWC27 homolog isoform X1 [Cucumis melo]	XP_008452743	0.0	499	360
Pe85I9.7	2 isoforms	+	8	3713	1947	U-box domain-containing 52-like isoform X2	648	gi|1000939541|ref|XP_015583281.1|PREDICTED: U-box domain-containing protein 52 isoform X2 [Ricinus communis]	XP_015583281	0.0	664	530
Pe85I9.8		+	1	1180	591	Leucine-rich repeat extensin 1	196	gi|743814855|ref|XP_011019798.1|PREDICTED: extensin-3 [Populus euphratica]	XP_011019798	7.20E-15	188	107
Pe85I9.9		+	12	4441	1719	Conserved oligomeric Golgi complex subunit 8	572	gi|743813028|ref|XP_011019411.1|PREDICTED: conserved oligomeric Golgi complex subunit 8 [Populus euphratica]	XP_011019411	0.0	575	529
Pe85I9.10		+	12	4637	1662	Methyltransferase [Citrus unshiu]	553	gi|566177884|ref|XP_002308544.2|hypothetical protein POPTR_0006s24210g [Populus trichocarpa]gi|550336981|gb|EEE92067.2|hypothetical protein POPTR_0006s24210g [Populus trichocarpa]	XP_002308544, EEE92067	0.0	555	476
Pe85I9.11		-	1	207	207	---Na---	68	No Blast Hit				
Pe85I9.12		+	2	2034	861	Eukaryotic initiation factor 4A-1	286	gi|1024017283|gb|KZV36516.1|eukaryotic initiation factor 4A-14-like [Dorcoceras hygrometricum]	KZV36516	0.0	286	284
Pe85I9.13		-	3	2719	1956	DNA RNA polymerases superfamily [Theobroma cacao]	651	gi|590728434|ref|XP_007099662.1|Gag protease polyprotein-like protein [Theobroma cacao]gi|508728474|gb|EOY20371.1|Gag protease polyprotein-like protein [Theobroma cacao]	XP_007099662, EOY20371	2.21E-73	502	255
Pe85I9.14		-	4	1140	531	PREDICTED: uncharacterized protein LOC107486813	176	gi|1012122950|ref|XP_015962868.1|PREDICTED: uncharacterized protein LOC107486813 [Arachis duranensis]	XP_015962868	3.49E-16	145	74
Pe85I9.15		-	1	369	369	PREDICTED: uncharacterized protein LOC107176124	122	gi|985438818|ref|XP_015383672.1|PREDICTED: uncharacterized protein LOC107176124 [Citrus sinensis]	XP_015383672	3.91E-10	99	58
Pe85I9.16		+	2	896	501	PREDICTED: uncharacterized protein LOC105638122 [Jatropha curcas]	166	gi|802630755|ref|XP_012077251.1|PREDICTED: uncharacterized protein LOC105638122 [Jatropha curcas]gi|643724874|gb|KDP34075.1|hypothetical protein JCGZ_07646 [Jatropha curcas]	XP_012077251, KDP34075	3.47E-36	121	91

Pe85I9.17	2 isoforms	+	9	4084	1035	Probable magnesium transporter NIPA9	344	gi|802630858|ref|XP_012077252.1|PREDICTED: probable magnesium transporter NIPA9 [Jatropha curcas]gi|643724875|gb|KDP34076.1|hypothetical protein JCGZ_07647 [Jatropha curcas]	XP_012077252, KDP34076	0.0	344	315
Pe85J23.1		+	7	3932	966	(Dimethylallyl)adenosine tRNA methylthiotransferase	321	gi|743803943|ref|XP_011017222.1|PREDICTED: uncharacterized protein LOC105120641 isoform X1 [Populus euphratica]gi|743803948|ref|XP_011017223.1|PREDICTED: uncharacterized protein LOC105120641 isoform X1 [Populus euphratica]	XP_011017222, XP_011017223	0.0	322	292
Pe85J23.2		+	2	1033	651	Hypothetical protein POPTR_0002s12930g [Populus trichocarpa]	216	gi|224063537|ref|XP_002301193.1|hypothetical protein POPTR_0002s12930g [Populus trichocarpa]gi|222842919|gb|EEE80466.1|hypothetical protein POPTR_0002s12930g [Populus trichocarpa]	XP_002301193, EEE80466	3.09E-98	217	177
Pe85J23.3-4		+	2	977	954	Probable carboxylesterase 2	209	gi|566202191|ref|XP_006374970.1|hypothetical protein POPTR_0014s03230g [Populus trichocarpa]gi|550323283|gb|ERP52767.1|hypothetical protein POPTR_0014s03230g [Populus trichocarpa]	XP_006374970, ERP52767	9.46E-97	207	172
Pe85J23.5		-	6	3571	939	26S proteasome non-atpase regulatory subunit 14 homolog	312	gi|1026045118|ref|XP_016568742.1|PREDICTED: 26S proteasome non-ATPase regulatory subunit 14 homolog [Capsicum annuum]	XP_016568742	0.0	312	312
Pe85J23.6		+	3	3369	1281	Zinc finger BED domain-containing RICESLEEPER 2-like	365	gi|1025031472|ref|XP_016495234.1|PREDICTED: zinc finger BED domain-containing protein RICESLEEPER 2-like [Nicotiana tabacum]	XP_016495234	5.68E-43	242	138
Pe85J23.7		+	4	2787	1008	Probable inactive heme oxygenase chloroplastic [Populus euphratica]	335	gi|566202219|ref|XP_006374984.1|hypothetical protein POPTR_0014s03350g [Populus trichocarpa]gi|550323297|gb|ERP52781.1|hypothetical protein POPTR_0014s03350g [Populus trichocarpa]	XP_006374984, ERP52781	3.24E-123	330	238
Pe85J23.8		-	15	9631	3066	Calcineurin-like metallo-phosphoesterase superfamily isoform 1 [Theobroma cacao]	1021	gi|566202223|ref|XP_006374985.1|hypothetical protein POPTR_0014s03370g [Populus trichocarpa]gi|566202225|ref|XP_006374986.1|hypothetical protein POPTR_0014s03370g [Populus trichocarpa]gi|550323299|gb|ERP52782.1|hypothetical protein POPTR_0014s03370g [Populus trichocarpa]gi|550323300|gb|ERP52783.1|hypothetical protein POPTR_0014s03370g [Populus trichocarpa]	XP_006374985, XP_006374986, ERP52782, ERP52783	0.0	1022	959
Pe85J23.9		+	1	760	492	Os03g0394200 [Oryza sativa Japonica Group]	163	gi|1021030862|gb|KZM88647.1|hypothetical protein DCAR_025722 [Daucus carota subsp. sativus]	KZM88647	1.37E-10	190	85
Pe85J23.10		+	6	4956	1362	Polyadenylate-binding RBP47-like	362	gi|743937297|ref|XP_011013053.1|PREDICTED: polyadenylate-binding protein RBP47-like [Populus euphratica]	XP_011013053	0.0	313	281
Pe85J23.11	1 isoform	+	9	5743	834	Uroporphyrinogen-III chloroplastic isoform X1 [Prunus mume]	277	gi|645226698|ref|XP_008220161.1|PREDICTED: uroporphyrinogen-III synthase, chloroplastic isoform X2 [Prunus mume]	XP_008220161	2.60E-132	235	216
Pe85J23.12		-	8	3068	1449	Serine carboxypeptidase II-3-like	482	gi|802592656|ref|XP_012071699.1|PREDICTED: serine carboxypeptidase II-3-like [Jatropha curcas]gi|643731053|gb|KDP38391.1|hypothetical protein JCGZ_04316 [Jatropha curcas]	XP_012071699, KDP38391	0.0	458	382
Pe85J23.13	1 isoform	+	4	1915	570	Hypothetical protein POPTR_0014s03410g [Populus trichocarpa]	189	gi|566202239|ref|XP_006374993.1|hypothetical protein POPTR_0014s03410g [Populus trichocarpa]gi|550323307|gb|ERP52790.1|hypoth	XP_006374993, ERP52790	1.01E-35	222	143

								etical protein POPTR_0014s03410g [Populus trichocarpa]				
Pe85J23.14		+	1	1620	1026	mRNA for AR781 family [Populus trichocarpa]	341	gi|802592662|ref|XP_012071703.1|PREDICTED: uncharacterized protein LOC105633680 [Jatropha curcas]	XP_012071703	1.70E-117	358	264
Pe85J23.15		-	6	1751	630	Acylamino-acid-releasing isoform 1 [Theobroma cacao]	209	gi|643731057|gb|KDP38395.1|hypothetical protein JCGZ_04320 [Jatropha curcas]	KDP38395	5.61E-119	196	185
Pe85O9.1	Incomplete 3'	-	8	2837	1227	Cell cycle checkpoint RAD17 isoform X2	409	gi|802753511|ref|XP_012088508.1|PREDICTED: cell cycle checkpoint protein RAD17 isoform X1 [Jatropha curcas]gi|643709458|gb|KDP23999.1|hypothetical protein JCGZ_25387 [Jatropha curcas]	XP_012088508, KDP23999	0.0	404	331
Pe85O9.2		+	8	3205	1329	3-dehydroquinate chloroplastic	442	gi|802753495|ref|XP_012088505.1|PREDICTED: 3-dehydroquinate synthase, chloroplastic [Jatropha curcas]gi|643709456|gb|KDP23997.1|hypothetical protein JCGZ_25385 [Jatropha curcas]	XP_012088505, KDP23997	0.0	444	407
Pe85O9.3	1 isoform	+	7	3324	594	Rac-like GTP-binding RHO1 [Nicotiana tabacum]	197	gi|595807043|ref|XP_007202540.1|hypothetical protein PRUPE_ppa011074mg [Prunus persica]gi|645273803|ref|XP_008242051.1|PREDICTED: rac-like GTP-binding protein ARAC1 [Prunus mume]gi|658005682|ref|XP_008337990.1|PREDICTED: rac-like GTP-binding protein ARAC1 [Malus domestica]gi|694372123|ref|XP_009363468.1|PREDICTED: rac-like GTP-binding protein ARAC1 [Pyrus x bretschneideri]gi|764511950|ref|XP_011462150.1|PREDICTED: rac-like GTP-binding protein ARAC1 [Fragaria vesca subsp. vesca]gi|462398071|gb|EMJ03739.1|hypothetical protein PRUPE_ppa011074mg [Prunus persica]	XP_007202540, XP_008242051, XP_008337990, XP_009363468, XP_011462150, EMJ03739	1.19E-134	197	195
Pe85O9.4		-	2	744	468	Heavy metal-associated isoprenylated plant 26	155	gi|641852436|gb|KDO71301.1|hypothetical protein CISIN_1g045611mg, partial [Citrus sinensis]	KDO71301	1.01E-76	159	135
Pe85O9.5	3 isoforms	-	4	2720	801	F-box At4g35930-like [Gossypium raimondii]	266	gi|802753434|ref|XP_012088491.1|PREDICTED: F-box protein At4g35930 [Jatropha curcas]gi|643709445|gb|KDP23986.1|hypothetical protein JCGZ_25374 [Jatropha curcas]	XP_012088491, KDP23986	5.12E-146	267	238
Pe85O9.6		+	1	441	441	Ribonuclease H At1g65750 family	146	gi|1021535283|ref|XP_016164673.1|PREDICTED: uncharacterized protein LOC107607211 [Arachis ipaensis]	XP_016164673	1.28E-25	129	76
Pe85O9.7	Incomplete 3'	+	2	1286	496	Mid1-complementing activity 1	165	gi|223529267|gb|EEF31239.1|conserved hypothetical protein [Ricinus communis]	EEF31239	8.95E-106	164	161
Pe86F9.1		+	2	757	427	---Na---	141	No Blast Hit				
Pe86F9.2		+	3	1704	1029	Cytochrome P450 CYP749A22-like	346	gi|566190890|ref|XP_002316006.2|hypothetical protein POPTR_0010s14900g [Populus trichocarpa]gi|550329837|gb|EEF02177.2|hypothetical protein POPTR_0010s14900g [Populus trichocarpa]	XP_002316006, EEF02177	4.52E-153	345	272
Pe86F9.3		-	1	306	306	PREDICTED: uncharacterized protein LOC107832345, partial	101	gi|1025007312|ref|XP_016515689.1|PREDICTED: uncharacterized protein LOC107832345, partial [Nicotiana tabacum]	XP_016515689	3.43E-01	46	33
Pe86F9.4		+	2	798	699	Gag protease poly [Theobroma cacao]	232	gi|590689992|ref|XP_007043384.1|Gag protease polyprotein [Theobroma	XP_007043384, EOX99215	2.00E-24	147	87

								cacao]gi|508707319|gb|EOX99215.1|Gag protease polyprotein [Theobroma cacao]				
Pe86F9.5		-	1	339	339	---Na---	112	No Blast Hit				
Pe86F9.6		-	2	631	507	---Na---	168	No Blast Hit				
Pe86F9.7		-	2	644	546	---Na---	181	No Blast Hit				
Pe86F9.8		+	1	255	255	---Na---	84	No Blast Hit				
Pe86F9.9		-	2	1218	1128	Gag protease poly [Theobroma cacao]	375	gi|590616752|ref|XP_007023594.1|Gag protease polyprotein [Theobroma cacao]gi|508778960|gb|EOY26216.1|Gag protease polyprotein [Theobroma cacao]	XP_007023594, EOY26216	9.41E-47	297	154
Pe86F9.10		+	1	631	423	---Na---	140	No Blast Hit				
Pe86F9.11		+	1	384	384	---Na---	127	No Blast Hit				
Pe86F9.12	1 isoform	+	3	20501	444	Gag protease poly [Theobroma cacao]	580	gi|590728434|ref|XP_007099662.1|Gag protease polyprotein-like protein [Theobroma cacao]gi|508728474|gb|EOY20371.1|Gag protease polyprotein-like protein [Theobroma cacao]	XP_007099662, EOY20371	1.25E-64	435	219
Pe86F9.13		+	1	821	384	---Na---	127	No Blast Hit				
Pe86F9.14		-	3	3050	1740	Gag protease poly [Theobroma cacao]	579	gi|590581218|ref|XP_007014287.1|Gag protease polyprotein [Theobroma cacao]gi|508784650|gb|EOY31906.1|Gag protease polyprotein [Theobroma cacao]	XP_007014287, EOY31906	1.40E-49	323	168
Pe86F9.15		+	1	718	588	Modifier of snc1 1	195	gi|548862062|gb|ERN19427.1|hypothetical protein AMTR_s00069p00173060 [Amborella trichopoda]	ERN19427	8.08E-02	127	71
Pe86F9.17		-	2	1163	975	Gag protease poly [Theobroma cacao]	324	gi|590581218|ref|XP_007014287.1|Gag protease polyprotein [Theobroma cacao]gi|508784650|gb|EOY31906.1|Gag protease polyprotein [Theobroma cacao]	XP_007014287, EOY31906	1.77E-31	202	117
Pe86F9.18		-	2	650	381	---Na---	126	No Blast Hit				
Pe86F9.19		-	3	1695	678	---Na---	225	No Blast Hit				
Pe86F9.20		-	3	1020	381	---Na---	126	No Blast Hit				
Pe86F9.21		+	1	372	372	Cytochrome P450 CYP749A22-like	123	gi|743801782|ref|XP_011015632.1|PREDICTED : cytochrome P450 CYP749A22-like [Populus euphratica]	XP_011015632	4.44E-18	88	62
Pe86F9.22		+	5	2094	1431	Cytochrome P450 CYP749A22-like	515	gi|566190890|ref|XP_002316006.2|hypothetical protein POPTR_0010s14900g [Populus trichocarpa]gi|550329837|gb|EEF02177.2|hypothetical protein POPTR_0010s14900g [Populus trichocarpa]	XP_002316006, EEF02177	0.0	512	407
Pe86F9.23		+	1	294	294	---Na---	97	No Blast Hit				
Pe86F9.24		-	3	2675	1587	Gag protease poly [Theobroma cacao]	528	gi|590581218|ref|XP_007014287.1|Gag protease polyprotein [Theobroma cacao]gi|508784650|gb|EOY31906.1|Gag protease polyprotein [Theobroma cacao]	XP_007014287, EOY31906	3.99E-50	323	166
Pe86F9.25		+	1	255	255	---Na---	84	No Blast Hit				
Pe86F9.26		+	1	201	201	---Na---	66	No Blast Hit				
Pe86F9.27		-	1	351	351	---Na---	116	No Blast Hit				
Pe86F9.28		+	1	288	288	---Na---	95	No Blast Hit				

Pe86H7.1		+	3	772	579	---Na---	192	No Blast Hit				
Pe86H7.2		+	1	213	213	---Na---	70	No Blast Hit				
Pe86H7.3		-	1	1268	798	Chlorophyll a-b binding 2 [Populus trichocarpa]	265	gi|224114357|ref|XP_002316737.1|Chlorophyll a-b binding protein 2 [Populus trichocarpa]gi|222859802|gb|EEE97349.1|Chlorophyll a-b binding protein 2 [Populus trichocarpa]	XP_002316737, EEE97349	1.05E-171	265	251
Pe86H7.4		+	1	1277	798	Chlorophyll a-b binding 2 [Populus trichocarpa]	265	gi|224114357|ref|XP_002316737.1|Chlorophyll a-b binding protein 2 [Populus trichocarpa]gi|222859802|gb|EEE97349.1|Chlorophyll a-b binding protein 2 [Populus trichocarpa]	XP_002316737, EEE97349	2.26E-172	265	252
Pe86H7.5		-	2	660	510	Ribonuclease H At1g65750	169	gi|702502859|ref|XP_010038887.1|PREDICTED: uncharacterized protein LOC104427495 [Eucalyptus grandis]	XP_010038887	2.06E-03	131	58
Pe86H7.6		-	3	1143	498	---Na---	165	No Blast Hit				
Pe86H7.7		-	2	833	582	PREDICTED: uncharacterized protein LOC107767132	193	gi|1025417097|ref|XP_016441540.1|PREDICTED: uncharacterized protein LOC107767132 [Nicotiana tabacum]	XP_016441540	1.44E-04	88	49
Pe86H7.8		-	1	228	228	---Na---	75	No Blast Hit				
Pe86H7.9		-	1	1617	681	Gag-Pol poly	226	gi|1025093233|ref|XP_016457574.1|PREDICTED: uncharacterized mitochondrial protein AtMg00300-like [Nicotiana tabacum]	XP_016457574	2.11E-32	176	108
Pe86H7.10		-	1	1053	1053	Transposon Ty3-G Gag-Pol poly	350	gi|950956371|ref|XP_014496836.1|PREDICTED: uncharacterized protein LOC106758422 [Vigna radiata var. radiata]	XP_014496836	4.77E-73	275	184
Pe86H7.11		-	1	1278	1278	Retrotransposable element Tf2	425	gi|571452688|ref|XP_006579166.1|PREDICTED: uncharacterized protein LOC102667767 [Glycine max]	XP_006579166	8.75E-48	407	196
Pe86H7.12	4 isoforms	+	2	4497	702	PREDICTED: uncharacterized protein LOC105633969 [Jatropha curcas]	233	gi|802596133|ref|XP_012072077.1|PREDICTED: uncharacterized protein LOC105633969 [Jatropha curcas]gi|643730511|gb|KDP37943.1|hypothetical protein JCGZ_04586 [Jatropha curcas]	XP_012072077, KDP37943	2.61E-64	247	171
Pe86H7.13		-	2	3738	3459	LRR receptor-like serine threonine- kinase FLS2	1152	gi|566165232|ref|XP_002305701.2|FLAGELLIN-SENSITIVE 2 family protein [Populus trichocarpa]gi|550340449|gb|EEE86212.2|FLAGELLIN-SENSITIVE 2 family protein [Populus trichocarpa]	XP_002305701, EEE86212	0.0	1155	953
Pe89E10.1		-	1	309	309	---Na---	102	No Blast Hit				
Pe89E10.2		+	3	1080	174	---Na---	57	No Blast Hit				
Pe89E10.3		-	1	183	183	---Na---	60	No Blast Hit				
Pe89E10.4		-	1	417	417	---Na---	138	No Blast Hit				
Pe89E10.5		-	2	511	423	---Na---	140	No Blast Hit				
Pe89E10.6		+	1	344	198	---Na---	65	No Blast Hit				
Pe89E10.7		+	1	192	192	---Na---	63	No Blast Hit				
Pe89E10.8		+	3	2469	942	Homeobox-leucine zipper HAT5-like	313	gi|566206481|ref|XP_002321530.2|hypothetical protein POPTR_0015s07640g [Populus trichocarpa]gi|118488004|gb|ABK95823.1|unknown [Populus trichocarpa]gi|550322261|gb|EEF05657.2|hypothetical protein POPTR_0015s07640g [Populus trichocarpa]	XP_002321530, ABK95823, EEF05657	0.0	315	285

Pe89E10.9	1 isoform	-	5	2526	729	Integral membrane HPP family [Theobroma cacao]	242	gi|224135645|ref|XP_002322125.1|hypothetical protein POPTR_0015s07650g [Populus trichocarpa]gi|222869121|gb|EEF06252.1|hypothetical protein POPTR_0015s07650g [Populus trichocarpa]	XP_002322125, EEF06252	7.24E-110	248	199
Pe89E10.10	1 isoform	-	13	4327	1794	LRR receptor-like serine threonine- kinase FEI 2	597	gi|118484080|gb|ABK93925.1|unknown [Populus trichocarpa]	ABK93925	0.0	603	547
Pe89E10.11		+	3	2242	1752	NRT1 PTR FAMILY -like	583	gi|255540891|ref|XP_002511510.1|PREDICTED: protein NRT1/ PTR FAMILY 2.8 [Ricinus communis]gi|223550625|gb|EEF52112.1|nitrate transporter, putative [Ricinus communis]	XP_002511510, EEF52112	0.0	566	457
Pe89E10.12		-	1	234	234	---Na---	77	No Blast Hit				
Pe89E10.13		-	1	201	201	---Na---	66	No Blast Hit				
Pe89E10.14		-	1	243	243	---Na---	80	No Blast Hit				
Pe89E10.15		-	1	234	234	---Na---	77	No Blast Hit				
Pe89E10.16		+	1	592	306	---Na---	101	No Blast Hit				
Pe89E10.17	Incomplete 3'	+	3	466	324	---Na---	108	No Blast Hit				
Pe93A7.1		-	2	853	759	Aquaporin TIP1-1-like	252	gi|387235454|gb|AFJ74380.1|tonoplast intrinsic protein 2 [Hevea brasiliensis]	AFJ74380	6.17E-152	252	236
Pe93A7.2		+	1	162	162	---Na---	53	No Blast Hit				
Pe93A7.3		-	1	330	330	---Na---	109	No Blast Hit				
Pe93A7.4		-	4	1125	759	---Na---	252	No Blast Hit				
Pe93A7.5		-	1	288	288	---Na---	95	No Blast Hit				
Pe93A7.6		-	1	1248	489	Retrotransposon [Theobroma cacao]	162	gi|823155331|ref|XP_012477568.1|PREDICTED: uncharacterized protein LOC105793188 [Gossypium raimondii]	XP_012477568	1.47E-38	157	111
Pe93A7.7		-	1	210	210	---Na---	69	No Blast Hit				
Pe93A7.8		+	1	240	240	---Na---	79	No Blast Hit				
Pe93A7.9		+	2	392	297	---Na---	98	No Blast Hit				
Pe93A7.10		+	1	1374	390	Retrotransposon [Theobroma cacao]	165	gi|823155331|ref|XP_012477568.1|PREDICTED: uncharacterized protein LOC105793188 [Gossypium raimondii]	XP_012477568	1.43E-38	160	113
Pe93A7.11	Incomplete 3'	+	1	188	188	---Na---	62	No Blast Hit				
Pe93F5.1		+	3	660	294	Zinc finger BED domain-containing RICESLEEPER 2-like	97	gi|1026059536|ref|XP_016574213.1|PREDICTED: zinc finger BED domain-containing protein RICESLEEPER 2-like [Capsicum annuum]	XP_016574213	3.42E+00	75	41
Pe93F5.2		+	2	323	246	---Na---	81	No Blast Hit				
Pe93F5.3		+	3	1533	357	---Na---	118	No Blast Hit				
Pe93F5.4		+	1	192	192	---Na---	63	No Blast Hit				
Pe93F5.5		-	1	2003	1290	Eukaryotic aspartyl protease family [Theobroma cacao]	429	gi|567923190|ref|XP_006453601.1|hypothetical protein CICLE_v10010459mg [Citrus clementina]gi|557556827|gb|ESR66841.1|hypothetical protein CICLE_v10010459mg [Citrus clementina]gi|641840555|gb|KDO59475.1|hypothetical protein CISIN_1g044367mg [Citrus sinensis]	XP_006453601, ESR66841, KDO59475	3.27E-116	449	276
Pe93F5.6		-	6	5271	1722	Probable cyclic nucleotide-	573	gi|743807605|ref|XP_011018037.1|PREDICTED	XP_011018037	0.0	573	532

						gated ion channel 5 [Citrus sinensis]		: probable cyclic nucleotide-gated ion channel 5 [Populus euphratica]				
Pe93F5.7	1 isoform	-	2	2437	246	Probable cyclic nucleotide-gated ion channel 5	84	gi|566215322|ref|XP_002325129.2|cyclic nucleotide-gated ion channel 5 family protein [Populus trichocarpa]gi|550318518|gb|EEF03694.2|cyclic nucleotide-gated ion channel 5 family protein [Populus trichocarpa]	XP_002325129, EEF03694	7.29E-18	69	54
Pe93F5.8	1 isoform	-	5	4197	927	Phospholipid glycerol acyltransferase family isoform 1 [Theobroma cacao]	368	gi|566177140|ref|XP_002308407.2|hypothetical protein POPTR_0006s19730g [Populus trichocarpa]gi|550336671|gb|EEE91930.2|hypothetical protein POPTR_0006s19730g [Populus trichocarpa]	XP_002308407, EEE91930	1.05E-155	350	268
Pe93F5.9		-	2	11041	1140	APO chloroplastic	438	gi|223531136|gb|EEF32984.1|APO protein 2, chloroplast precursor, putative [Ricinus communis]	EEF32984	0.0	444	383
Pe93F5.10		-	2	785	537	Early nodulin 1	178	gi|1000946215|ref|XP_015581001.1|PREDICTED: early nodulin-like protein 1 [Ricinus communis]	XP_015581001	1.51E-53	175	123
Pe93F5.11		-	8	1759	828	Signal recognition particle receptor subunit alpha [Cicer arietinum]	275	gi|743807613|ref|XP_011018039.1|PREDICTED: signal recognition particle receptor subunit alpha-like [Populus euphratica]	XP_011018039	0.0	275	274
Pe93J9.1	Incomplete 3'	-	4	767	474	Glyceraldehyde-3-phosphate dehydrogenase chloroplastic	158	gi|743875087|ref|XP_011034836.1|PREDICTED: glyceraldehyde-3-phosphate dehydrogenase B, chloroplastic isoform X1 [Populus euphratica]	XP_011034836	1.04E-86	159	144
Pe93J9.2		-	6	2104	528	Localized to the inner membrane of the chloroplast [Theobroma cacao]	175	gi|590595616|ref|XP_007018106.1|Localized to the inner membrane of the chloroplast [Theobroma cacao]gi|508723434|gb|EOY15331.1|Localized to the inner membrane of the chloroplast [Theobroma cacao]	XP_007018106, EOY15331	5.43E-54	157	124
Pe93J9.3		+	15	4404	1560	MLO 9	519	gi|449453091|ref|XP_004144292.1|PREDICTED: MLO-like protein 9 [Cucumis sativus]gi|700192320|gb|KGN47524.1|hypothetical protein Csa_6G355430 [Cucumis sativus]	XP_004144292, KGN47524	0.0	535	415
Pe93J9.4		-	10	3768	1833	Dolichyl-diphosphooligosaccharide--glycosyltransferase subunit 1A	610	gi|224061851|ref|XP_002300630.1|ribophorin 1 family protein [Populus trichocarpa]gi|118483703|gb|ABK93745.1|unknown [Populus trichocarpa]gi|222842356|gb|EEE79903.1|ribophorin 1 family protein [Populus trichocarpa]	XP_002300630, ABK93745, EEE79903	0.0	615	536
Pe93J9.5		+	1	947	579	RING-H2 finger ATL80-like	192	gi|255539298|ref|XP_002510714.1|PREDICTED: RING-H2 finger protein ATL8 [Ricinus communis]gi|223551415|gb|EEF52901.1|protein with unknown function [Ricinus communis]	XP_002510714, EEF52901	1.02E-75	195	147
Pe93J9.6		+	9	5631	2688	Polymerase histidinol phosphatase- isoform 1 [Theobroma cacao]	895	gi|1000940073|ref|XP_015583112.1|PREDICTED: uncharacterized protein LOC8278033 [Ricinus communis]	XP_015583112	1.57E-120	380	264
Pe93J9.7		+	8	4198	1692	PREDICTED: uncharacterized protein LOC105137790	511	gi|743897358|ref|XP_011041967.1|PREDICTED: uncharacterized protein LOC105137790 [Populus euphratica]	XP_011041967	1.64E-02	230	107
Pe93J9.8		-	5	3349	2211	Monosaccharide-sensing 2-like [Gossypium hirsutum]	736	gi|590595661|ref|XP_007018119.1|Tonoplast monosaccharide transporter2 isoform 1 [Theobroma cacao]gi|590595664|ref|XP_007018120.1|Tonoplast monosaccharide transporter2 isoform 1 [Theobroma cacao]gi|590595668|ref|XP_007018121.1|Tonopl	XP_007018119, XP_007018120, XP_007018121, EOY15344, EOY15345, EOY15346	0.0	739	679

								ast monosaccharide transporter2 isoform 1 [Theobroma cacao]gi|508723447|gb|EOY15344.1|Tonoplast monosaccharide transporter2 isoform 1 [Theobroma cacao]gi|508723448|gb|EOY15345.1|Tonoplast monosaccharide transporter2 isoform 1 [Theobroma cacao]gi|508723449|gb|EOY15346.1|Tonoplast monosaccharide transporter2 isoform 1 [Theobroma cacao]				
Pe93J9.9		-	3	2987	1197	Cup-shaped cotyledon 3	398	gi|224061845|ref|XP_002300627.1|hypothetical protein POPTR_0002s00730g [Populus trichocarpa]gi|222842353|gb|EEE79900.1|hypothetical protein POPTR_0002s00730g [Populus trichocarpa]	XP_002300627, EEE79900	0.0	409	333
Pe93J9.10		-	8	5862	1107	RNA recognition motif-containing family [Populus trichocarpa]	368	gi|802604341|ref|XP_012073568.1|PREDICTED: RNA-binding protein 24-A [Jatropha curcas]gi|643728805|gb|KDP36742.1|hypothetical protein JCGZ_08033 [Jatropha curcas]	XP_012073568, KDP36742	7.27E-145	288	254
Pe93J9.11		+	4	3300	1812	Hydroxymethylglutaryl-reductase [Hevea brasiliensis]	603	gi|224065641|ref|XP_002301898.1|hydroxymethylglutaryl-CoA reductase family protein [Populus trichocarpa]gi|222843624|gb|EEE81171.1|hydroxymethylglutaryl-CoA reductase family protein [Populus trichocarpa]	XP_002301898, EEE81171	0.0	605	538
Pe93J9.12		-	4	2457	201	---Na---	66	No Blast Hit				
Pe93J9.13	1 isoform	+	3	2642	294	PREDICTED: uncharacterized protein LOC100259899 [Vitis vinifera]	97	gi|225457662|ref|XP_002275896.1|PREDICTED: uncharacterized protein LOC100259899 [Vitis vinifera]gi|731427554|ref|XP_010664021.1|PREDICTED: uncharacterized protein LOC100259899 [Vitis vinifera]gi|297745609|emb|CBI40774.3|unnamed protein product [Vitis vinifera]	XP_002275896, XP_010664021, CBI40774	7.00E-50	97	89
Pe93J9.14		+	2	908	810	DNA-binding ESCAROLA	269	gi|255539338|ref|XP_002510734.1|PREDICTED: AT-hook motif nuclear-localized protein 25 [Ricinus communis]gi|223551435|gb|EEF52921.1|DNA binding protein, putative [Ricinus communis]	XP_002510734, EEF52921	1,71E-111	296	229
Pe93J9.15		-	16	6131	3321	Pyrophosphate--fructose 6-phosphate 1-phosphotransferase subunit alpha	617	gi|224086120|ref|XP_002307823.1|pyrophosphate-dependent phosphofructokinase alpha subunit family protein [Populus trichocarpa]gi|222857272|gb|EEE94819.1|pyrophosphate-dependent phosphofructokinase alpha subunit family protein [Populus trichocarpa]	XP_002307823, EEE94819	0.0	617	585
Pe93J9.16		-	3	3050	945	bZIP transcription factor 60	314	gi|224086126|ref|XP_002307824.1|bZIP transcription factor family protein [Populus trichocarpa]gi|222857273|gb|EEE94820.1|bZIP transcription factor family protein [Populus trichocarpa]	XP_002307824, EEE94820	3.26E-76	312	210
Pe93J9.17		-	2	1160	855	Diacylglycerol kinase theta	284	gi|566173656|ref|XP_002307829.2|hypothetical protein POPTR_0005s28020g [Populus trichocarpa]gi|550339908|gb|EEE94825.2|hypothetical protein POPTR_0005s28020g [Populus trichocarpa]	XP_002307829, EEE94825	5.59E-119	292	225
Pe93J9.18		-	2	718	618	Zinc-binding family [Populus trichocarpa]	205	gi|118489570|gb|ABK96587.1|unknown [Populus trichocarpa x Populus deltoides]	ABK96587	5.48E-27	99	74
Pe93J9.19		+	1	615	615	Poly polymerase	204	gi|224083398|ref|XP_002307010.1|hypothetical	XP_002307010,	5.99E-55	220	149

						[Theobroma cacao]		protein POPTR_0005s28050g [Populus trichocarpa]gi|222856459|gb|EEE94006.1|hypothetical protein POPTR_0005s28050g [Populus trichocarpa]	EEE94006			
Pe93J9.20		-	1	1126	588	Calcium-binding CML19	195	gi|224086146|ref|XP_002307832.1|hypothetical protein POPTR_0005s28110g [Populus trichocarpa]gi|222857281|gb|EEE94828.1|hypothetical protein POPTR_0005s28110g [Populus trichocarpa]	XP_002307832, EEE94828	5.94E-63	180	135
Pe93J9.21		-	3	3897	1335	PREDICTED: uncharacterized protein At1g76660 [Ricinus communis]	444	gi|255539390|ref|XP_002510760.1|PREDICTED: uncharacterized protein At1g76660 [Ricinus communis]gi|223551461|gb|EEF52947.1|conserved hypothetical protein [Ricinus communis]	XP_002510760, EEF52947	0.0	472	397
Pe93J9.22		+	11	4457	2928	Cellulose synthase	975	gi|645255252|ref|XP_008233413.1|PREDICTED: probable cellulose synthase A catalytic subunit 3 [UDP-forming] [Prunus mume]	XP_008233413	0.0	750	655
Pe93J9.23	Incomplete 5'	-	3	2395	1604	Citron Rho-interacting kinase [Populus euphratica]	533	gi|566155477|ref|XP_002300606.2|hypothetical protein POPTR_0002s00310g [Populus trichocarpa]gi|550343966|gb|EEE79879.2|hypothetical protein POPTR_0002s00310g [Populus trichocarpa]	XP_002300606, EEE79879	0.0	533	442
Pe93K19.1		-	1	792	792	Subtilisin-like protease	263	gi|566161203|ref|XP_002304129.2|hypothetical protein POPTR_0003s06530g [Populus trichocarpa]gi|550342556|gb|EEE79108.2|hypothetical protein POPTR_0003s06530g [Populus trichocarpa]	XP_002304129, EEE79108	1.90E-106	213	186
Pe93K19.2		-	1	1799	480	Uncharacterized protein TCM_029159 [Theobroma cacao]	159	gi|590620896|ref|XP_007024658.1|Uncharacterized protein TCM_029159 [Theobroma cacao]gi|508780024|gb|EOY27280.1|Uncharacterized protein TCM_029159 [Theobroma cacao]	XP_007024658, EOY27280	5.70E-86	161	144
Pe93K19.3		-	7	5317	1965	Mono- di-acylglycerol lipase isoform 1 [Theobroma cacao]	654	gi|743881386|ref|XP_011036456.1|PREDICTED: uncharacterized protein LOC105133971 [Populus euphratica]gi|743881388|ref|XP_011036457.1|PREDICTED: uncharacterized protein LOC105133971 [Populus euphratica]	XP_011036456, XP_011036457	0.0	670	558
Pe93K19.4		-	9	2507	741	Proteasome subunit alpha type-6	246	gi|1011993226|ref|XP_015940788.1|PREDICTED: proteasome subunit alpha type-6 [Arachis duranensis]gi|1021468759|ref|XP_016181042.1|PREDICTED: proteasome subunit alpha type-6 [Arachis ipaensis]	XP_015940788, XP_016181042	6.64E-169	246	240
Pe93K19.5		+	4	1326	804	PREDICTED: uncharacterized protein LOC105116386 [Populus euphratica]	267	gi|743935313|ref|XP_011012020.1|PREDICTED: uncharacterized protein LOC105116386 [Populus euphratica]gi|743935315|ref|XP_011012021.1|PREDICTED: uncharacterized protein LOC105116386 [Populus euphratica]	XP_011012020, XP_011012021	9.72E-136	268	231
Pe93K19.6		+	5	2990	387	Small nuclear ribonucleo [Populus trichocarpa]	128	gi|255583960|ref|XP_002532727.1|PREDICTED: sm-like protein LSM1B [Ricinus communis]gi|223527535|gb|EEF29658.1|lsm1, putative [Ricinus communis]	XP_002532727, EEF29658	2.17E-80	128	128
Pe93K19.7	1 isoform	+	5	3954	1545	Transmembrane 87A	514	gi|802576360|ref|XP_012068854.1|PREDICTED: transmembrane protein 87A [Jatropha curcas]gi|643733833|gb|KDP40676.1|hypothetical protein JCGZ_24675 [Jatropha curcas]	XP_012068854, KDP40676	0.0	517	463
Pe93K19.8		-	1	2382	1875	Exocyst complex component EXO70A1-like	624	gi|802576358|ref|XP_012068853.1|PREDICTED: exocyst complex component EXO70A1-like [Jatropha	XP_012068853, KDP40675	0.0	630	561

								curcas]gi|643733832|gb|KDP40675.1|hypothetical protein JCGZ_24674 [Jatropha curcas]				
Pe93K19.9		-	2	2776	2106	Pollen receptor-like kinase 3	701	gi|224070780|ref|XP_002303233.1|hypothetical protein POPTR_0003s06660g [Populus trichocarpa]gi|222840665|gb|EEE78212.1|hypothetical protein POPTR_0003s06660g [Populus trichocarpa]	XP_002303233, EEE78212	0.0	665	476
Pe93K19.10		+	5	2195	756	Jasmonate ZIM domain 6 protein	251	gi|724086333|gb|AIY25007.1|JAZ2 [Hevea brasiliensis]	AIY25007	1.31E-67	238	163
Pe93K19.11		+	12	4691	1269	Oligouridylate-binding protein 1B-like	422	gi|1012339226|gb|KYP50462.1|Nucleolysin TIAR [Cajanus cajan]	KYP50462	0.0	400	380
Pe93K19.12		-	5	2663	984	FLUORESCENT IN BLUE chloroplastic isoform X1	327	gi|566149422|ref|XP_006369118.1|hypothetical protein POPTR_0001s16620g [Populus trichocarpa]gi|550347477|gb|ERP65687.1|hypothetical protein POPTR_0001s16620g [Populus trichocarpa]	XP_006369118, ERP65687	8.34E-138	329	256
Pe93K19.13		+	12	7516	4062	Mediator of RNA polymerase II transcription subunit 15a	1353	gi|255576609|ref|XP_002529195.1|PREDICTED: uncharacterized protein LOC8260445 isoform X1 [Ricinus communis]gi|223531373|gb|EEF33209.1|DNA binding protein, putative [Ricinus communis]	XP_002529195, EEF33209	0.0	1403	1091
Pe93K19.14		-	10	2594	822	Peroxisomal (S)-2-hydroxy-acid oxidase GLO4 [Ricinus communis]	273	gi|747052957|ref|XP_011072615.1|PREDICTED: peroxisomal (S)-2-hydroxy-acid oxidase GLO4-like isoform X5 [Sesamum indicum]gi|747052959|ref|XP_011072616.1|PREDICTED: peroxisomal (S)-2-hydroxy-acid oxidase GLO4-like isoform X5 [Sesamum indicum]gi|747052961|ref|XP_011072617.1|PREDICTED: peroxisomal (S)-2-hydroxy-acid oxidase GLO4-like isoform X5 [Sesamum indicum]	XP_011072615, XP_011072616, XP_011072617	1.31E-157	270	249
Pe93K19.15		-	9	3275	1857	Zinc finger CCCH domain-containing 62-like	618	gi|225442050|ref|XP_002272277.1|PREDICTED: zinc finger CCCH domain-containing protein 62 [Vitis vinifera]gi|297742965|emb|CBI35832.3|unnamed protein product [Vitis vinifera]	XP_002272277, CBI35832	1.61E-114	303	243
Pe93K19.16		-	5	3347	657	Kda vesicle transport [Malus domestica]	218	gi|743910164|ref|XP_011048585.1|PREDICTED: 25.3 kDa vesicle transport protein-like [Populus euphratica]	XP_011048585	6.50E-152	218	217
Pe93K19.17	1 isoform	+	4	2762	1389	2-oxoglutarate-dependent dioxygenase family isoform 1 [Theobroma cacao]	495	gi|566161258|ref|XP_002303323.2|hypothetical protein POPTR_0003s06770g [Populus trichocarpa]gi|550342578|gb|EEE78302.2|hypothetical protein POPTR_0003s06770g [Populus trichocarpa]	XP_002303323, EEE78302	6.54E-120	482	293
Pe93K19.18		-	4	3076	1608	Hypothetical protein POPTR_0003s06780g [Populus trichocarpa]	535	gi|566161260|ref|XP_002304236.2|hypothetical protein POPTR_0003s06780g [Populus trichocarpa]gi|550342579|gb|EEE79215.2|hypothetical protein POPTR_0003s06780g [Populus trichocarpa]	XP_002304236, EEE79215	0.0	536	439
Pe93K19.19		-	9	5668	4629	ABC transporter B family member 19-like	1542	gi|743910166|ref|XP_011048586.1|PREDICTED: ABC transporter B family member 1-like [Populus euphratica]	XP_011048586	0.0	1556	1326
Pe93K19.20		-	10	10373	4254	PREDICTED: uncharacterized protein LOC105133993 isoform X4	1417	gi|743881565|ref|XP_011036504.1|PREDICTED: uncharacterized protein LOC105133993 isoform X4 [Populus euphratica]	XP_011036504	0.0	1442	1046
Pe93K19.21	Incomplete 5'	-	5	1999	477	DNAJ homolog subfamily B member 6 isoform X1	158	gi|566149394|ref|XP_006369104.1|hypothetical protein POPTR_0001s16500g [Populus	XP_006369104, ERP65673	4.58E-58	157	124

								trichocarpa]gi|550347463|gb|ERP65673.1|hypothetical protein POPTR_0001s16500g [Populus trichocarpa]				
Pe93M2.1		+	2	1287	747	Ethylene-responsive transcription factor ERF113	248	gi|297734512|emb|CBI15759.3|unnamed protein product [Vitis vinifera]	CBI15759	8.26E-52	130	106
Pe93M2.2		-	6	2672	777	CRAL-TRIO domain-containing YKL091C-like isoform X1 [Citrus sinensis]	258	gi|566212441|ref|XP_006373203.1|hypothetical protein POPTR_0017s09620g [Populus trichocarpa]gi|550319909|gb|ERP51000.1|hypothetical protein POPTR_0017s09620g [Populus trichocarpa]	XP_006373203, ERP51000	3.24E-127	254	215
Pe93M2.3	1 isoform	+	4	3605	1254	Probable beta-1,4-xylosyltransferase IRX10L	417	gi|802770173|ref|XP_012090559.1|PREDICTED: probable beta-1,4-xylosyltransferase IRX10L [Jatropha curcas]gi|802770177|ref|XP_012090560.1|PREDICTED: probable beta-1,4-xylosyltransferase IRX10L [Jatropha curcas]gi|802770181|ref|XP_012090561.1|PREDICTED: probable beta-1,4-xylosyltransferase IRX10L [Jatropha curcas]gi|317106607|dbj|BAJ53114.1|JHL07K02.4 [Jatropha curcas]gi|643706388|gb|KDP22520.1|hypothetical protein JCGZ_26351 [Jatropha curcas]	XP_012090559, XP_012090560, XP_012090561, BAJ53114, KDP22520	0.0	417	403
Pe93M2.4		+	4	1146	507	Rubredoxin family [Populus trichocarpa]	168	gi|118489155|gb|ABK96384.1|unknown [Populus trichocarpa x Populus deltoides]	ABK96384	9.01E-73	171	135
Pe93M2.5		-	9	2827	816	Chromophore lyase chloroplastic-like isoform X1 [Glycine max]	271	gi|224122242|ref|XP_002318786.1|hypothetical protein POPTR_0012s11160g [Populus trichocarpa]gi|222859459|gb|EEE97006.1|hypothetical protein POPTR_0012s11160g [Populus trichocarpa]	XP_002318786, EEE97006	1.51E-175	267	255
Pe93M2.6		-	1	399	192	Transferring glycosyl [Theobroma cacao]	63	gi|118489234|gb|ABK96423.1|unknown [Populus trichocarpa x Populus deltoides]gi|118489750|gb|ABK96676.1|unknown [Populus trichocarpa x Populus deltoides]	ABK96423, ABK96676	5.35E-07	51	35
Pe93M2.7		-	5	7069	567	Ankyrin repeat family	188	gi|802770209|ref|XP_012090569.1|PREDICTED: succinate dehydrogenase assembly factor 2, mitochondrial [Jatropha curcas]gi|317106611|dbj|BAJ53118.1|JHL07K02.8 [Jatropha curcas]gi|643706392|gb|KDP22524.1|hypothetical protein JCGZ_26355 [Jatropha curcas]	XP_012090569, BAJ53118, KDP22524	1.12E-91	188	158
Pe93M2.8		+	3	1912	1224	G2 mitotic-specific cyclin-B	407	gi|1000985031|ref|XP_002511074.2|PREDICTED: uncharacterized protein LOC8275475 isoform X2 [Ricinus communis]	XP_002511074	0.0	413	316
Pe93M2.9		-	4	1747	942	Carbonyl reductase [NADPH] 1-like	313	gi|224122250|ref|XP_002318788.1|short-chain dehydrogenase/reductase family protein [Populus trichocarpa]gi|222859461|gb|EEE97008.1|short-chain dehydrogenase/reductase family protein [Populus trichocarpa]	XP_002318788, EEE97008	0.0	313	282
Pe93M2.10		+	5	2299	1404	Calcium-binding mitochondrial carrier S -1-like	467	gi|802770150|ref|XP_012090554.1|PREDICTED: calcium-binding mitochondrial carrier protein SCaMC-1-like isoform X1 [Jatropha curcas]gi|317106605|dbj|BAJ53112.1|JHL07K02.2 [Jatropha curcas]gi|643706386|gb|KDP22518.1|hypothetical protein JCGZ_26349 [Jatropha curcas]	XP_012090554, BAJ53112, KDP22518	0.0	506	417

Pe93M2.11		+	1	1221	1221	Pentatricopeptide repeat-containing At5g61800	406	gi|566197920|ref|XP_002318186.2|hypothetical protein POPTR_0012s11260g [Populus trichocarpa]gi|550326865|gb|EEE96406.2|hypothetical protein POPTR_0012s11260g [Populus trichocarpa]	XP_002318186, EEE96406	1.40E-173	382	304
Pe93M2.12		+	2	580	501	NFU1 iron-sulfur mitochondrial	166	gi|802770138|ref|XP_012090551.1|PREDICTED: uncharacterized protein LOC105648695 [Jatropha curcas]gi|643706384|gb|KDP22516.1|hypothetical protein JCGZ_26347 [Jatropha curcas]	XP_012090551, KDP22516	2.57E-49	163	120
Pe93M2.13		+	1	450	450	Uncharacterized protein LOC105648695 [Jatropha curcas]	149	gi|802770138|ref|XP_012090551.1|PREDICTED: uncharacterized protein LOC105648695 [Jatropha curcas]gi|643706384|gb|KDP22516.1|hypothetical protein JCGZ_26347 [Jatropha curcas]	XP_012090551, KDP22516	3.44E-53	150	117
Pe93M2.14		+	6	3397	1632	Calnexin precursor family [Populus trichocarpa]	543	gi|255540003|ref|XP_002511066.1|PREDICTED: calnexin homolog [Ricinus communis]gi|223550181|gb|EEF51668.1|calnexin, putative [Ricinus communis]	XP_002511066, EEF51668	0.0	517	472
Pe93M2.15		+	2	781	690	PREDICTED: uncharacterized protein LOC107832538	229	gi|1025375703|ref|XP_016515889.1|PREDICTED: uncharacterized protein LOC107832538 [Nicotiana tabacum]	XP_016515889	1.24E-35	207	108
Pe93M2.16		+	1	1872	1872	PREDICTED: uncharacterized protein LOC104883796	623	gi|731373493|ref|XP_010666661.1|PREDICTED: uncharacterized protein LOC104883796 [Beta vulgaris subsp. vulgaris]	XP_010666661	8.21E-117	588	340
Pe93M2.17		+	2	1052	993	Ribonuclease H At1g65750	330	gi|659126450|ref|XP_008463188.1|PREDICTED: putative ribonuclease H protein At1g65750 [Cucumis melo]	XP_008463188	1.28E-63	282	169
Pe93M2.18		-	16	6197	2961	Staphylococcal nuclease domain-containing 1-like	986	gi|743919028|ref|XP_011003528.1|PREDICTED: staphylococcal nuclease domain-containing protein 1-like [Populus euphratica]	XP_011003528	0.0	984	898
Pe93M2.19		+	2	1132	960	Inositol polyphosphate multikinase alpha-like [Nelumbo nucifera]	319	gi|566207181|ref|XP_002321769.2|hypothetical protein POPTR_0015s12100g [Populus trichocarpa]gi|550322541|gb|EEF05896.2|hypothetical protein POPTR_0015s12100g [Populus trichocarpa]	XP_002321769, EEF05896	6.49E-152	290	245
Pe93M2.20		+	1	2920	2169	Pentatricopeptide repeat-containing mitochondrial [Vitis vinifera]	605	gi|1000984818|ref|XP_015579664.1|PREDICTED: pentatricopeptide repeat-containing protein At2g01510, mitochondrial [Ricinus communis]	XP_015579664	0.0	605	526
Pe93M2.21		+	3	3001	2040	Frigida	684	gi|802770094|ref|XP_012090538.1|PREDICTED: protein FRIGIDA [Jatropha curcas]gi|643706371|gb|KDP22503.1|hypothetical protein JCGZ_26334 [Jatropha curcas]	XP_012090538, KDP22503	0.0	649	447
Pe93M2.22		+	4	2635	987	Peroxidase 60	411	gi|743894288|ref|XP_011040390.1|PREDICTED: peroxidase 60 [Populus euphratica]	XP_011040390	9.66E-159	324	263
Pe93M2.23		+	7	2178	1257	Cell division cycle cofactor of APC complex-like	418	gi|802770086|ref|XP_012090536.1|PREDICTED: cell division cycle 20.2, cofactor of APC complex-like [Jatropha curcas]	XP_012090536	0.0	420	349
Pe93M2.24	5 isoforms	-	7	2608	618	Superoxide dismutase [Fe] chloroplastic isoform X2	205	gi|821324908|ref|NP_001295626.1|superoxide dismutase [Fe], chloroplastic [Jatropha curcas]gi|380085077|gb|AFD34189.1|Fe superoxide dismutase [Jatropha curcas]	NP_001295626, AFD34189	7.71E-107	221	179
Pe93M2.25		-	9	1882	954	Superoxide dismutase [Fe] chloroplastic isoform X1	317	gi|255539971|ref|XP_002511050.1|PREDICTED: superoxide dismutase [Fe], chloroplastic [Ricinus communis]gi|223550165|gb|EEF51652.1|superoxide dismutase [fe], putative [Ricinus communis]	XP_002511050, EEF51652	1.74E-148	314	260

Pe93M4.1		+	1	420	420	NBS-LRR resistance [Theobroma cacao]	139	gi|566224699|ref|XP_006370959.1|hypothetical protein POPTR_0019s02150g [Populus trichocarpa]gi|550316542|gb|ERP48756.1|hypothetical protein POPTR_0019s02150g [Populus trichocarpa]	XP_006370959, ERP48756	1.63E-34	140	98
Pe93M4.2		-	3	1002	792	Chlorophyll a-b binding chloroplastic	263	gi|20671|emb|CAA49149.1|chlorophyll a/b-binding protein [Pisum sativum]gi|141448064|gb|ABO87611.1|chloroplast chlorophyll-a/b binding protein [Pisum sativum]	CAA49149, ABO87611	5.14E-174	263	253
Pe93M4.3		+	6	2620	1365	Mediator of RNA polymerase II transcription subunit 1-like	454	gi|566154168|ref|XP_006370339.1|hypothetical protein POPTR_0001s41790g [Populus trichocarpa]gi|550349518|gb|ERP66908.1|hypothetical protein POPTR_0001s41790g [Populus trichocarpa]	XP_006370339, ERP66908	3.04E-112	429	280
Pe93M4.4		-	1	222	222	---Na---	73	No Blast Hit				
Pe93M4.5		-	1	306	306	---Na---	101	No Blast Hit				
Pe93M4.6		-	1	774	774	Transposon TX1 149 kda partial [Glycine soja]	257	gi|702287125|ref|XP_010046745.1|PREDICTED: LOW QUALITY PROTEIN: uncharacterized protein LOC104435745 [Eucalyptus grandis]	XP_010046745	5.48E-34	255	131
Pe93M4.7		-	1	216	216	---Na---	71	No Blast Hit				
Pe93M4.8		+	1	243	243	---Na---	80	No Blast Hit				
Pe93M4.9		+	2	741	303	---Na---	100	No Blast Hit				
Pe93M4.10		-	1	282	282	---Na---	93	No Blast Hit				
Pe93M4.11		-	1	243	243	---Na---	80	No Blast Hit				
Pe93M4.12		-	1	375	375	---Na---	124	No Blast Hit				
Pe93M4.13		-	1	1548	1548	RNA recognition motif [Medicago truncatula]	515	gi|1012324306|gb|KYP36320.1|Transposon TX1 uncharacterized [Cajanus cajan]	KYP36320	1.62E-07	325	133
Pe93M4.14	1 isoform	+	7	3063	1998	Cyclic nucleotide-gated ion channel 4	665	gi|1009110676|ref|XP_015896826.1|PREDICTED: cyclic nucleotide-gated ion channel 4 [Ziziphus jujuba]	XP_015896826	0.0	629	528
Pe93M4.15		-	2	1196	342	Dynein light chain family [Populus trichocarpa]	113	gi|566154178|ref|XP_006370344.1|dynein light chain family protein [Populus trichocarpa]gi|550349523|gb|ERP66913.1|dynein light chain family protein [Populus trichocarpa]	XP_006370344, ERP66913	1.50E-53	113	100
Pe93M4.16		+	2	2311	822	Membrane lipo lipid attachment site [Medicago truncatula]	273	gi|566195155|ref|XP_002316935.2|hypothetical protein POPTR_0011s12710g [Populus trichocarpa]gi|550328251|gb|EEE97547.2|hypothetical protein POPTR_0011s12710g [Populus trichocarpa]	XP_002316935, EEE97547	4.53E-155	273	237
Pe93N7.1	Incomplete 3'	-	3	1805	1062	Indole-3-pyruvate monooxygenase YUCCA6-like	354	gi|255551064|ref|XP_002516580.1|PREDICTED: indole-3-pyruvate monooxygenase YUCCA6 [Ricinus communis]gi|223544400|gb|EEF45921.1|monooxygenase, putative [Ricinus communis]	XP_002516580, EEF45921	0.0	355	324
Pe93N7.2	1 isoform	+	3	2324	1125	Dehydration-responsive RD22-like	372	gi|743841896|ref|XP_011026571.1|PREDICTED: dehydration-responsive protein RD22-like [Populus euphratica]	XP_011026571	2.36E-175	402	301
Pe93N7.3	1 isoform	+	10	7866	2133	PREDICTED: uncharacterized protein LOC105638281	710	gi|802633411|ref|XP_012077461.1|PREDICTED: uncharacterized protein LOC105638281 [Jatropha curcas]	XP_012077461	0.0	725	478
Pe93N7.4		+	4	3925	2277	Golgin subfamily A member 4 [Theobroma	758	gi|802633414|ref|XP_012077462.1|PREDICTED: uncharacterized protein LOC105638282	XP_012077462, KDP34224	0.0	763	625

						cacao]		[Jatropha curcas]gi|643725023|gb|KDP34224.1|hypothetical protein JCGZ_07795 [Jatropha curcas]				
Pe93N7.5		-	1	960	960	Hypothetical protein AMTR_s00071p00160560	319	gi|548862650|gb|ERN20008.1|hypothetical protein AMTR_s00071p00160560 [Amborella trichopoda]	ERN20008	1.64E-49	321	188
Pe93N7.6		+	1	921	921	ORFIII-like partial [Melicytus alpinus]	306	gi|548862647|gb|ERN20005.1|hypothetical protein AMTR_s00071p00160140 [Amborella trichopoda]	ERN20005	5.51E-120	242	205
Pe93N7.7		-	2	1434	861	Zinc finger FYVE domain-containing 26 isoform 1 [Theobroma cacao]	286	gi|802633422|ref|XP_012077466.1|PREDICTED: uncharacterized protein LOC105638286 [Jatropha curcas]gi|643725029|gb|KDP34230.1|hypothetical protein JCGZ_07801 [Jatropha curcas]	XP_012077466, KDP34230	9.88E-64	293	188
Pe93N7.8		+	3	2034	1710	F-box LRR-repeat At5g38386	569	gi|743814327|ref|XP_011019688.1|PREDICTED: putative F-box/LRR-repeat protein At5g38386 [Populus euphratica]	XP_011019688	9.82E-126	565	326
Pe93N7.9		+	2	1947	1245	Hypothetical protein POPTR_0006s26160g [Populus trichocarpa]	434	gi|566178219|ref|XP_002308629.2|hypothetical protein POPTR_0006s26160g [Populus trichocarpa]gi|550337113|gb|EEE92152.2|hypothetical protein POPTR_0006s26160g [Populus trichocarpa]	XP_002308629, EEE92152	6.35E-159	415	299
Pe93N7.10	1 isoform	-	10	5402	942	CHY-type CTCHY-type RING-type Zinc finger isoform 1 [Theobroma cacao]	313	gi|802633436|ref|XP_012077474.1|PREDICTED: RING finger and CHY zinc finger domain-containing protein 1 isoform X1 [Jatropha curcas]gi|643725037|gb|KDP34238.1|hypothetical protein JCGZ_07809 [Jatropha curcas]	XP_012077474, KDP34238	0.0	313	275
Pe93N7.11		+	1	2607	1926	Leucine-rich repeat extensin 4	641	gi|743841750|ref|XP_011026538.1|PREDICTED: leucine-rich repeat extensin-like protein 4 isoform X1 [Populus euphratica]	XP_011026538	8.83E-169	403	316
Pe93N7.12	3 isoforms	-	6	5524	2082	SWIM zinc finger family isoform 2 [Theobroma cacao]	693	gi|743814398|ref|XP_011019704.1|PREDICTED: uncharacterized protein LOC105122342 isoform X1 [Populus euphratica]	XP_011019704	0.0	697	631
Pe93N7.13		+	1	1512	1512	Molybdate transporter 1-like	503	gi|566178232|ref|XP_002308631.2|hypothetical protein POPTR_0006s26220g [Populus trichocarpa]gi|550337119|gb|EEE92154.2|hypothetical protein POPTR_0006s26220g [Populus trichocarpa]	XP_002308631, EEE92154	0.0	460	407
Pe93N7.14		-	4	8889	5085	Enhancer of polycomb-like transcription factor isoform 1 [Theobroma cacao]	1694	gi|802639947|ref|XP_012078606.1|PREDICTED: uncharacterized protein LOC105639237 [Jatropha curcas]gi|643722525|gb|KDP32275.1|hypothetical protein JCGZ_13200 [Jatropha curcas]	XP_012078606, KDP32275	0.0	1727	1228
Pe93N7.15	1 isoform	-	11	2952	987	Psbp domain-containing chloroplastic isoform X1	328	gi|743814466|ref|XP_011019720.1|PREDICTED: psbP domain-containing protein 5, chloroplastic [Populus euphratica]	XP_011019720	9.82E-141	296	245
Pe93N7.16	5 isoforms	+	2	2734	387	Polyadenylate-binding -interacting 5-like [Gossypium hirsutum]	128	gi|802639959|ref|XP_012078612.1|PREDICTED: polyadenylate-binding protein-interacting protein 6 [Jatropha curcas]gi|802639963|ref|XP_012078613.1|PREDICTED: polyadenylate-binding protein-interacting protein 6 [Jatropha curcas]gi|643722531|gb|KDP32281.1|hypothetical protein JCGZ_13206 [Jatropha curcas]	XP_012078612, XP_012078613, KDP32281	7.23E-32	112	86
Pe93N7.17		-	4	2923	1941	SPOC domain Transcription elongation factor S-II isoform 1 [Theobroma cacao]	646	gi|1000941299|ref|XP_002532142.2|PREDICTED: uncharacterized protein LOC8271812 [Ricinus communis]	XP_002532142	0.0	711	461

Pe93N7.18		-	1	1374	1374	SPOC domain Transcription elongation factor S-II isoform 2 [Theobroma cacao]	457	gi|566178247|ref|XP_002309587.2|hypothetical protein POPTR_0006s26300g [Populus trichocarpa]gi|550337126|gb|EEE93110.2|hypothetical protein POPTR_0006s26300g [Populus trichocarpa]	XP_002309587, EEE93110	6.10E-113	470	305
Pe93N7.19	2 isoforms	-	4	2980	1305	E3 ubiquitin- ligase At1g12760-like isoform X1 [Gossypium hirsutum]	434	gi|224143048|ref|XP_002324833.1|zinc finger family protein [Populus trichocarpa]gi|222866267|gb|EEF03398.1|zinc finger family protein [Populus trichocarpa]	XP_002324833, EEF03398	0.0	437	371
Pe93N7.20		+	2	2826	1497	Serine threonine phosphatase 2A 57 kda regulatory subunit B iota isoform-like	498	gi|802639992|ref|XP_012078625.1|PREDICTED: serine/threonine protein phosphatase 2A 57 kDa regulatory subunit B' iota isoform [Jatropha curcas]gi|643722538|gb|KDP32288.1|hypothetical protein JCGZ_13213 [Jatropha curcas]	XP_012078625, KDP32288	0.0	498	464
Pe93N7.21	1 isoform	-	6	3327	666	Ferredoxin-related family [Populus trichocarpa]	221	gi|356563826|ref|XP_003550159.1|PREDICTED: photosynthetic NDH subunit of subcomplex B 3, chloroplastic-like isoform X1 [Glycine max]gi|947055616|gb|KRH05069.1|hypothetical protein GLYMA_17G205000 [Glycine max]	XP_003550159, KRH05069	4.40E-61	135	113
Pe93N7.22		+	2	2128	1794	Pentatricopeptide repeat superfamily isoform 1 [Theobroma cacao]	597	gi|224089100|ref|XP_002308636.1|hypothetical protein POPTR_0006s26360g [Populus trichocarpa]gi|222854612|gb|EEE92159.1|hypothetical protein POPTR_0006s26360g [Populus trichocarpa]	XP_002308636, EEE92159	0.0	599	496
Pe93N7.23		+	2	1414	1056	Gibberellin 3-beta-dioxygenase 1-like	351	gi|743814549|ref|XP_011019738.1|PREDICTED: gibberellin 3-beta-dioxygenase 1-like [Populus euphratica]	XP_011019738	3.23E-157	332	265
Pe93N7.24	1 isoform	-	9	5094	834	Coiled-coil domain-containing 93	277	gi|224092398|ref|XP_002309591.1|paramyosin-related family protein [Populus trichocarpa]gi|222855567|gb|EEE93114.1|paramyosin-related family protein [Populus trichocarpa]	XP_002309591, EEE93114	3.43E-96	254	197
Pe93O18.1	Incomplete 3'	-	5	1070	575	Crossover junction endonuclease MUS81 isoform X2	191	gi|743897682|ref|XP_011042136.1|PREDICTED: crossover junction endonuclease MUS81 isoform X2 [Populus euphratica]	XP_011042136	3.14E-94	191	166
Pe93O18.2	1 isoform	+	6	3100	411	PREDICTED: uncharacterized protein LOC8276531 [Ricinus communis]	136	gi|255568962|ref|XP_002525451.1|PREDICTED: uncharacterized protein LOC8276531 [Ricinus communis]gi|223535264|gb|EEF36941.1|conserved hypothetical protein [Ricinus communis]	XP_002525451, EEF36941	7.14E-71	141	124
Pe93O18.3		+	2	1313	1215	Myosin heavy chain kinase B-like	404	gi|802573991|ref|XP_012068600.1|PREDICTED: F-box/WD repeat-containing protein sel-10 [Jatropha curcas]gi|643733649|gb|KDP40492.1|hypothetical protein JCGZ_24491 [Jatropha curcas]	XP_012068600, KDP40492	0.0	407	333
Pe93O18.4		-	7	4165	1587	Ubiquitin carboxyl-terminal hydrolase 24 isoform X2	569	gi|1000979789|ref|XP_015570827.1|PREDICTED: ubiquitin carboxyl-terminal hydrolase 24 isoform X2 [Ricinus communis]	XP_015570827	0.0	547	404
Pe93O18.5		-	4	2650	1068	Gag protease poly [Theobroma cacao]	355	gi|590689992|ref|XP_007043384.1|Gag protease polyprotein [Theobroma cacao]gi|508707319|gb|EOX99215.1|Gag protease polyprotein [Theobroma cacao]	XP_007043384, EOX99215	3.15E-15	181	84
Pe93O18.6		+	1	387	387	Probable pectinesterase pectinesterase inhibitor 13	128	gi|951038589|ref|XP_014517179.1|PREDICTED: uncharacterized protein LOC106774649 [Vigna radiata var. radiata]	XP_014517179	6.92E-32	122	90
Pe93O18.7		+	1	810	810	Hypothetical protein PHAVU_003G065500g [Phaseolus vulgaris]	269	gi|593781517|ref|XP_007153799.1|hypothetical protein PHAVU_003G065500g [Phaseolus vulgaris]gi|561027153|gb|ESW25793.1|hypothetical protein PHAVU_003G065500g [Phaseolus	XP_007153799, ESW25793	3.81E-07	262	113

								vulgaris]				
Pe93O18.8		+	11	5251	1611	Glycosyltransferase-like KOBITO 1	536	gi|802573987|ref|XP_012068598.1|PREDICTED: glycosyltransferase-like KOBITO 1 [Jatropha curcas]gi|643733647|gb|KDP40490.1|hypothetical protein JCGZ_24489 [Jatropha curcas]	XP_012068598, KDP40490	0.0	531	492
Pe93O18.9		+	1	1632	1632	Scopoletin glucosyltransferase-like [Populus euphratica]	543	gi|743847809|ref|XP_011028074.1|PREDICTED: UDP-glycosyltransferase 73B4-like isoform X2 [Populus euphratica]	XP_011028074	0.0	535	400
Pe93O18.10	1 isoform	-	2	7643	1164	Bifunctional endo-1,4-beta-xylanase -like	387	gi|743886229|ref|XP_011037794.1|PREDICTED: uncharacterized protein LOC105134906 isoform X1 [Populus euphratica]	XP_011037794	3.24E-82	405	224
Pe93O18.11		+	5	1839	1422	Iq-domain isoform 1 [Theobroma cacao]	473	gi|223548128|gb|EEF49620.1|conserved hypothetical protein [Ricinus communis]	EEF49620	0.0	471	374
Pe99P16.1		-	1	279	279	---Na---	92	No Blast Hit				
Pe99P16.2		-	1	548	216	---Na---	71	No Blast Hit				
Pe99P16.3		-	1	330	330	PREDICTED: uncharacterized protein LOC104605763	109	gi|720041566|ref|XP_010268950.1|PREDICTED: uncharacterized protein LOC104605763 [Nelumbo nucifera]	XP_010268950	1.69E-22	114	70
Pe99P16.4		+	1	366	366	---Na---	121	No Blast Hit				
Pe99P16.5		-	1	498	498	Gag protease poly [Theobroma cacao]	165	gi|590691529|ref|XP_007043808.1|Gag protease polyprotein [Theobroma cacao]gi|508707743|gb|EOX99639.1|Gag protease polyprotein [Theobroma cacao]	XP_007043808, EOX99639	6.45E-03	154	69
Pe99P16.6		-	1	360	360	Gag protease poly [Theobroma cacao]	119	gi|590580527|ref|XP_007014093.1|Gag protease polyprotein [Theobroma cacao]gi|508784456|gb|EOY31712.1|Gag protease polyprotein [Theobroma cacao]	XP_007014093, EOY31712	3.68E-01	102	50
Pe99P16.7		-	1	312	312	---Na---	103	No Blast Hit				
Pe99P16.8		+	1	327	327	---Na---	108	No Blast Hit				
Pe99P16.9		+	2	1722	987	---Na---	328	No Blast Hit				
Pe99P16.10	1 isoform	-	33	15022	4605	Lysine-specific demethylase 5B isoform X2	1534	gi|743826047|ref|XP_011022706.1|PREDICTED: lysine-specific demethylase 5B isoform X2 [Populus euphratica]	XP_011022706	0.0	1533	1201
Pe99P16.11		+	10	5008	1467	Probable serine threonine-kinase At5g41260 isoform X1 [Gossypium hirsutum]	488	gi|743852460|ref|XP_011029267.1|PREDICTED: probable serine/threonine-protein kinase At5g41260 [Populus euphratica]	XP_011029267	0.0	488	464
Pe99P16.12		-	3	2473	1332	Tubulin beta chain-like	443	gi|460240224|gb|AGH08228.1|beta-tubulin 10 [Salix arbutifolia]	AGH08228	0.0	439	438
Pe99P16.13	1 isoform	-	15	8332	1581	Glutamate-cysteine ligase isoform 1 [Theobroma cacao]	526	gi|743826024|ref|XP_011022699.1|PREDICTED: glutamate--cysteine ligase, chloroplastic-like [Populus euphratica]	XP_011022699	0.0	526	482
Pe99P16.14		+	1	249	249	---Na---	82	No Blast Hit				
Pe99P16.15		-	2	801	717	PREDICTED: uncharacterized protein LOC107405433	238	gi|1009173738|ref|XP_015867972.1|PREDICTED: uncharacterized protein LOC107405433 [Ziziphus jujuba]	XP_015867972	5.93E-32	264	137
Pe101F21.1		-	7	4835	1806	Remorin family isoform 2 [Theobroma cacao]	601	gi|802592553|ref|XP_012071648.1|PREDICTED: uncharacterized protein LOC105633635 [Jatropha curcas]gi|802592555|ref|XP_012071649.1|PREDICTED: uncharacterized protein LOC105633635 [Jatropha curcas]gi|643730989|gb|KDP38327.1|hypothetical protein JCGZ_04252 [Jatropha curcas]	XP_012071648, XP_012071649, KDP38327	0.0	611	504

Pe101F21.2	+	2	1258	663	---Na---	220	No Blast Hit				
Pe101F21.3	+	1	703	585	---Na---	194	No Blast Hit				
Pe101F21.4	+	1	243	243	---Na---	80	No Blast Hit				
Pe101F21.5	+	1	255	255	Gag protease poly [Theobroma cacao]	84	gi|590691529|ref|XP_007043808.1|Gag protease polyprotein [Theobroma cacao]gi|508707743|gb|EOX99639.1|Gag protease polyprotein [Theobroma cacao]	XP_007043808, EOX99639	4.78E-09	82	50
Pe101F21.6	-	1	423	423	---Na---	140	No Blast Hit				
Pe101F21.7	-	1	387	387	---Na---	128	No Blast Hit				
Pe101F21.8	+	1	543	543	Gag protease poly [Theobroma cacao]	180	gi|590600561|ref|XP_007019489.1|Gag protease polyprotein [Theobroma cacao]gi|508724817|gb|EOY16714.1|Gag protease polyprotein [Theobroma cacao]	XP_007019489, EOY16714	7.43E-20	183	98
Pe101F21.9	+	1	357	357	Gag protease poly [Theobroma cacao]	118	gi|590693137|ref|XP_007044250.1|DNA/RNA polymerases superfamily protein [Theobroma cacao]gi|508708185|gb|EOY00082.1|DNA/RNA polymerases superfamily protein [Theobroma cacao]	XP_007044250, EOY00082	3.31E-04	120	49
Pe101F21.10	+	2	1101	414	---Na---	137	No Blast Hit				
Pe101F21.11	+	2	322	198	---Na---	65	No Blast Hit				
Pe101H15.1	+	5	1052	453	Two-component response regulator-like APRR2 isoform X4	150	gi|823213267|ref|XP_012439375.1|PREDICTED: two-component response regulator-like APRR2 isoform X3 [Gossypium raimondii]	XP_012439375	7.03E-51	149	126
Pe101H15.2	+	2	1037	588	---Na---	195	No Blast Hit				
Pe101H15.3	-	1	225	225	---Na---	74	No Blast Hit				
Pe101H15.4	+	1	285	285	---Na---	94	No Blast Hit				
Pe101H15.5	+	2	1515	1008	Gag protease poly [Theobroma cacao]	335	gi|590689992|ref|XP_007043384.1|Gag protease polyprotein [Theobroma cacao]gi|508707319|gb|EOX99215.1|Gag protease polyprotein [Theobroma cacao]	XP_007043384, EOX99215	1.07E-31	206	116
Pe101H15.6	+	4	24687	582	---Na---	232	No Blast Hit				
Pe101H15.7	+	1	462	462	---Na---	153	No Blast Hit				
Pe101H15.8	-	1	288	288	---Na---	95	No Blast Hit				
Pe101H15.9	-	1	276	276	Retrovirus-related Pol poly from transposon TNT 1-94	91	gi|1012357422|gb|KYP68607.1|Retrovirus-related Pol polyprotein from transposon TNT 1-94 [Cajanus cajan]	KYP68607	2.18E-21	74	59
Pe101H15.10	+	1	621	621	1510387Aretrotransposon del1-46	206	gi|226407|prf||1510387Aretrotransposon del1-46	1510387A	4.49E-02	82	48
Pe101H15.11	+	1	270	270	---Na---	89	No Blast Hit				
Pe101H15.12	-	1	330	330	---Na---	109	No Blast Hit				
Pe101H15.13	-	1	267	267	Transcription factor LUX [Populus euphratica]	88	gi|743876717|ref|XP_011035272.1|PREDICTED: transcription factor PCL1-like [Populus euphratica]gi|743876719|ref|XP_011035273.1|PREDICTED: transcription factor PCL1-like [Populus euphratica]	XP_011035272, XP_011035273	1.08E-16	81	59
Pe101H15.14	+	1	288	288	General transcription factor IIE subunit 2-like [Nelumbo nucifera]	95	gi|802740833|ref|XP_012086956.1|PREDICTED: general transcription factor IIE subunit 2-like [Jatropha curcas]gi|643712045|gb|KDP25473.1|hypothetica	XP_012086956, KDP25473	1.63E-31	89	72

								l protein JCGZ_20629 [Jatropha curcas]				
Pe101K14+ 141H13.1	Incomplete 3'	-	6	4395	2309	Autophagy-related 18g-like isoform X1	769	gi|802794236|ref|XP_012092325.1|PREDICTED: autophagy-related protein 18g [Jatropha curcas]gi|643704464|gb|KDP21528.1|hypothetical protein JCGZ_21999 [Jatropha curcas]	XP_012092325, KDP21528	0.0	777	670
Pe101K14+ 141H13.2		-	7	2525	1245	Receptor-like cytosolic serine threonine- kinase RBK2	414	gi|743870713|ref|XP_011033653.1|PREDICTED: receptor-like cytosolic serine/threonine-protein kinase RBK2 isoform X1 [Populus euphratica]gi|743870717|ref|XP_011033654.1|PREDICTED: receptor-like cytosolic serine/threonine-protein kinase RBK2 isoform X1 [Populus euphratica]	XP_011033653, XP_011033654	0.0	421	374
Pe101K14+ 141H13.3		+	17	4136	1524	Major facilitator superfamily isoform 1 [Theobroma cacao]	507	gi|566159508|ref|XP_002302812.2|sugar transporter family protein [Populus trichocarpa]gi|550345631|gb|EEE82085.2|sugar transporter family protein [Populus trichocarpa]	XP_002302812, EEE82085	0.0	510	440
Pe101K14+ 141H13.4		+	1	456	456	[Arabidopsis thaliana]	151	gi|643704459|gb|KDP21523.1|hypothetical protein JCGZ_21994 [Jatropha curcas]	KDP21523	2.94E-10	152	82
Pe101K14+ 141H13.5		-	4	3516	1515	DNase I-like superfamily [Theobroma cacao]	504	gi|802794201|ref|XP_012092317.1|PREDICTED: uncharacterized protein LOC105650052 [Jatropha curcas]gi|643704458|gb|KDP21522.1|hypothetical protein JCGZ_21993 [Jatropha curcas]	XP_012092317, KDP21522	0.0	502	432
Pe101K14+ 141H13.6		-	1	1650	1476	E3 ubiquitin- ligase RF298 [Brassica napus]	491	gi|923804551|ref|XP_013688540.1|PREDICTED: putative E3 ubiquitin-protein ligase RF298 [Brassica napus]gi|923804553|ref|XP_013688541.1|PREDICTED: putative E3 ubiquitin-protein ligase RF298 [Brassica napus]	XP_013688540, XP_013688541	5.82E-62	524	264
Pe101K14+ 141H13.7		-	15	11778	6576	Piezo-type mechanosensitive ion channel homolog isoform X2	2191	gi|1000954391|ref|XP_015578214.1|PREDICTED: piezo-type mechanosensitive ion channel homolog isoform X2 [Ricinus communis]	XP_015578214	0.0	2152	1910
Pe101K14+ 141H13.8		-	7	3235	960	Exosome complex component RRP4	319	gi|802793619|ref|XP_012092306.1|PREDICTED: exosome complex component RRP4 [Jatropha curcas]gi|643704451|gb|KDP21515.1|hypothetical protein JCGZ_21986 [Jatropha curcas]	XP_012092306, KDP21515	0.0	320	285
Pe101K14+ 141H13.9		+	1	1050	1050	Probable galacturonosyltransferase-like 4	349	gi|743831941|ref|XP_011024154.1|PREDICTED: probable galacturonosyltransferase-like 4 [Populus euphratica]	XP_011024154	0.0	342	318
Pe101K14+ 141H13.10		-	1	1909	1341	BSD domain-containing 1	446	gi|255567648|ref|XP_002524803.1|PREDICTED: BSD domain-containing protein 1 [Ricinus communis]gi|223535987|gb|EEF37646.1|synapse-associated protein, putative [Ricinus communis]	XP_002524803, EEF37646	7.73E-133	456	310
Pe101K14+ 141H13.11		+	1	1282	705	Hypothetical protein POPTR_0008s01890g [Populus trichocarpa]	234	gi|566181836|ref|XP_002310997.2|hypothetical protein POPTR_0008s01890g [Populus trichocarpa]gi|550332198|gb|EEE88364.2|hypothetical protein POPTR_0008s01890g [Populus trichocarpa]	XP_002310997, EEE88364	2.81E-27	294	136
Pe101K14+ 141H13.12	1 isoform	-	7	4627	1647	Oxidoreductase family [Populus trichocarpa]	548	gi|1000954433|ref|XP_015578228.1|PREDICTED: uncharacterized protein LOC8285863 isoform X1 [Ricinus communis]	XP_015578228	0.0	574	445
Pe101K14+ 141H13.13		-	1	890	471	Hypothetical protein POPTR_0001s29430g [Populus trichocarpa]	156	gi|224056319|ref|XP_002298800.1|hypothetical protein POPTR_0001s29430g [Populus trichocarpa]gi|222846058|gb|EEE83605.1|hypothetical protein POPTR_0001s29430g [Populus trichocarpa]	XP_002298800, EEE83605	2.08E-04	201	83

Pe101K14+ 141H13.14		+	12	4596	1356	Elongation factor mitochondrial	451	gi|731395250|ref|XP_010652106.1|PREDICTED : elongation factor Tu, mitochondrial [Vitis vinifera]gi|147784261|emb|CAN61809.1|hypothetical protein VITISV_014296 [Vitis vinifera]gi|296086691|emb|CBI32326.3|unnamed protein product [Vitis vinifera]	XP_010652106, CAN61809, CBI32326	0.0	453	417
Pe101K14+ 141H13.15	1 isoform	-	5	4658	1236	Hypothetical protein POPTR_0014s13430g [Populus trichocarpa]	411	gi|566204099|ref|XP_002321059.2|hypothetical protein POPTR_0014s13430g [Populus trichocarpa]gi|550324122|gb|EEE99374.2|hypothetical protein POPTR_0014s13430g [Populus trichocarpa]	XP_002321059, EEE99374	9.07E-154	420	304
Pe101K14+ 141H13.16		+	1	303	303	BTB POZ and MATH domain-containing 2-like	100	gi|802793583|ref|XP_012092297.1|PREDICTED : BTB/POZ and MATH domain-containing protein 2-like [Jatropha curcas]gi|643704439|gb|KDP21503.1|hypothetical protein JCGZ_21974 [Jatropha curcas]	XP_012092297, KDP21503	1.23E-33	99	78
Pe101K14+ 141H13.17		+	4	3425	1206	BTB POZ and MATH domain-containing 2-like	401	gi|731395256|ref|XP_010652109.1|PREDICTED : BTB/POZ and MATH domain-containing protein 2-like [Vitis vinifera]gi|296086694|emb|CBI32329.3|unnamed protein product [Vitis vinifera]	XP_010652109, CBI32329	0.0	402	378
Pe101K14+ 141H13.18		+	1	264	264	---Na---	87	No Blast Hit				
Pe101K14+ 141H13.19		-	7	2442	756	Endo-1,3 1,4-beta-D-glucanase-like	251	gi|255567666|ref|XP_002524812.1|PREDICTED : endo-1,3;1,4-beta-D-glucanase isoform X2 [Ricinus communis]gi|223535996|gb|EEF37655.1|endo-1,3-1,4-beta-d-glucanase, putative [Ricinus communis]	XP_002524812, EEF37655	1.20E-101	239	190
Pe101K14+ 141H13.20		-	7	2043	756	Endo-1,3 1,4-beta-D-glucanase-like	251	gi|225436938|ref|XP_002275501.1|PREDICTED : endo-1,3;1,4-beta-D-glucanase [Vitis vinifera]gi|147767003|emb|CAN67688.1|hypothetical protein VITISV_036609 [Vitis vinifera]	XP_002275501, CAN67688	1.79E-109	239	189
Pe101K14+ 141H13.21		-	6	1618	531	Endo-1,3 1,4-beta-D-glucanase-like	176	gi|629119820|gb|KCW84310.1|hypothetical protein EUGRSUZ_B01156 [Eucalyptus grandis]	KCW84310	8.01E-74	184	142
Pe101K14+ 141H13.22		-	7	2465	756	Endo-1,3 1,4-beta-D-glucanase-like	251	gi|565385032|ref|XP_006358424.1|PREDICTED : endo-1,3;1,4-beta-D-glucanase-like [Solanum tuberosum]	XP_006358424	2.77E-110	240	200
Pe101K14+ 141H13.23		-	7	5394	759	Endo-1,3 1,4-beta-D-glucanase-like	252	gi|255567666|ref|XP_002524812.1|PREDICTED : endo-1,3;1,4-beta-D-glucanase isoform X2 [Ricinus communis]gi|223535996|gb|EEF37655.1|endo-1,3-1,4-beta-d-glucanase, putative [Ricinus communis]	XP_002524812, EEF37655	6.09E-109	237	190
Pe101K14+ 141H13.24		+	1	931	609	Heavy-metal-associated domain [Medicago truncatula]	202	gi|743796278|ref|XP_011004702.1|PREDICTED : uncharacterized protein LOC105111125 [Populus euphratica]	XP_011004702	5.00E-18	238	114
Pe101K14+ 141H13.25		+	1	363	363	---Na---	120	No Blast Hit				
Pe101K14+ 141H13.26		-	1	2658	2658	Isoamylase chloroplastic	885	gi|802793555|ref|XP_012092290.1|PREDICTED : isoamylase 2, chloroplastic [Jatropha curcas]gi|643704433|gb|KDP21497.1|hypothetical protein JCGZ_21968 [Jatropha curcas]	XP_012092290, KDP21497	0.0	882	699
Pe101K14+ 141H13.27		-	1	1249	894	EID1-like F-box 3	297	gi|731395314|ref|XP_002276328.2|PREDICTED : EID1-like F-box protein 3 [Vitis vinifera]	XP_002276328	1.41E-119	245	203
Pe101K14+ 141H13.28		+	10	11646	4296	Tudor PWWP MBT domain-containing isoform	1431	gi|802793215|ref|XP_012092286.1|PREDICTED : HUA2-like protein 3 isoform X1 [Jatropha	XP_012092286, KDP21494	0.0	1449	995

						2 [Theobroma cacao]		curcas]gi|643704430|gb|KDP21494.1|hypothetical protein JCGZ_21965 [Jatropha curcas]				
Pe101K14+141H13.29		-	3	1115	474	---Na---	157	No Blast Hit				
Pe101K14+141H13.30		-	2	1315	513	---Na---	170	No Blast Hit				
Pe101K14+141H13.31	1 isoform	+	1	4655	303	---Na---	100	No Blast Hit				
Pe101K14+141H13.32		+	7	2533	1011	Transcription factor bhlh69-like isoform X2	336	gi|802792911|ref|XP_012092278.1|PREDICTED: uncharacterized protein LOC105650022 [Jatropha curcas]gi|802793185|ref|XP_012092279.1|PREDICTED: uncharacterized protein LOC105650022 [Jatropha curcas]gi|643704422|gb|KDP21486.1|hypothetical protein JCGZ_21957 [Jatropha curcas]	XP_012092278, XP_012092279, KDP21486	2.43E-74	342	200
Pe101K14+141H13.33		+	1	1355	967	Adenylate isopentenyltransferase chloroplastic	320	gi|223527128|gb|EEF29303.1|ATP binding protein, putative, partial [Ricinus communis]	EEF29303	4.53E-162	325	266
Pe101K14+141H13.34		-	1	477	477	Tetratricopeptide repeat-like superfamily [Theobroma cacao]	158	gi|743926739|ref|XP_011007542.1|PREDICTED: uncharacterized protein LOC105113180 [Populus euphratica]	XP_011007542	1.12E-29	157	91
Pe101K14+141H13.35		+	1	540	540	Ribonuclease H At1g65750 family	179	gi|720039522|ref|XP_010268332.1|PREDICTED: uncharacterized protein LOC104605308 [Nelumbo nucifera]	XP_010268332	1.44E-08	114	61
Pe101K14+141H13.36		-	1	456	456	Transcription cofactor vestigial 3	151	gi|593798734|ref|XP_007162405.1|hypothetical protein PHAVU_001G149300g [Phaseolus vulgaris]gi|561035869|gb|ESW34399.1|hypothetical protein PHAVU_001G149300g [Phaseolus vulgaris]	XP_007162405, ESW34399	2.52E-27	144	82
Pe101O4.1	Incomplete 5'	+	10	2590	1073	Kinesin KIF22 isoform X1 [Jatropha curcas]	356	gi|743907274|ref|XP_011047063.1|PREDICTED: kinesin heavy chain isoform X3 [Populus euphratica]	XP_011047063	7.28E-56	409	213
Pe101O4.2		-	19	8386	2235	DEAD-box ATP-dependent RNA helicase 28	744	gi|641866207|gb|KDO84892.1|hypothetical protein CISIN_1g004808mg [Citrus sinensis]	KDO84892	0.0	748	667
Pe101O4.3	1 isoform	-	6	1645	699	Pre-16S rRNA nuclease	232	gi|566168242|ref|XP_006385047.1|hypothetical protein POPTR_0004s23400g [Populus trichocarpa]gi|550341815|gb|ERP62844.1|hypothetical protein POPTR_0004s23400g [Populus trichocarpa]	XP_006385047, ERP62844	2.66E-101	223	184
Pe101O4.4		-	6	2413	933	UPF0307 ECA0281-like isoform X3	310	gi|224083048|ref|XP_002306937.1|hypothetical protein POPTR_0005s26230g [Populus trichocarpa]gi|222856386|gb|EEE93933.1|hypothetical protein POPTR_0005s26230g [Populus trichocarpa]	XP_002306937, EEE93933	4.88E-102	307	230
Pe101O4.5		-	6	2750	834	CMSS1 [Jatropha curcas]	277	gi|743912240|ref|XP_010999994.1|PREDICTED: protein CMSS1 [Populus euphratica]	XP_010999994	3.13E-113	269	202
Pe101O4.6		-	3	2402	309	Prolyl 4-hydroxylase 1	102	gi|596210596|ref|XP_007223857.1|hypothetical protein PRUPE_ppa010789mg [Prunus persica]gi|462420793|gb|EMJ25056.1|hypothetical protein PRUPE_ppa010789mg [Prunus persica]	XP_007223857, EMJ25056	1.25E-30	72	66
Pe101O4.7		-	2	1754	678	Transposon Tf2-1 poly isoform X1	225	gi|674233051|gb|KFK25816.1|hypothetical protein AALP_AA8G165200 [Arabis alpina]	KFK25816	1.27E-19	144	88
Pe101O4.8		-	6	3440	546	Prolyl 4-hydroxylase 1 isoform X3	181	gi|1000945741|ref|XP_015581184.1|PREDICTED: prolyl 4-hydroxylase 1 isoform X3 [Ricinus communis]	XP_015581184	1.60E-66	121	112

Pe101O4.9		-	6	1636	300	Prolyl 4-hydroxylase 1	99	gi|641831476|gb|KDO50532.1|hypothetical protein CISIN_1g027393mg [Citrus sinensis]	KDO50532	2.88E-41	97	83
Pe101O4.10		+	1	624	624	---Na---	207	No Blast Hit				
Pe101O4.11		+	1	2586	1233	Ribonuclease H At1g65750	411	gi|1021535283|ref|XP_016164673.1|PREDICTED: uncharacterized protein LOC107607211 [Arachis ipaensis]	XP_016164673	1.07E-58	403	210
Pe101O4.12		+	14	9702	1632	Signal peptide peptidase-like 3	543	gi|743898977|ref|XP_011042780.1|PREDICTED: signal peptide peptidase-like 3 isoform X3 [Populus euphratica]	XP_011042780	0.0	531	457
Pe101O4.13		-	1	948	318	Lipid-transfer DIR1	105	gi|255577622|ref|XP_002529688.1|PREDICTED: putative lipid-transfer protein DIR1 [Ricinus communis]gi|223530836|gb|EEF32699.1|lipid binding protein, putative [Ricinus communis]	XP_002529688, EEF32699	2.91E-38	107	89
Pe101O4.14		+	1	1230	1230	F-box At5g49610	409	gi|566260203|ref|XP_006389655.1|hypothetical protein POPTR_0021s01040g [Populus trichocarpa]gi|550312484|gb|ERP48569.1|hypothetical protein POPTR_0021s01040g [Populus trichocarpa]	XP_006389655, ERP48569	4.94E-26	432	184
Pe101O4.15	1 isoform	-	8	5272	1200	SMG9-like	399	gi|802777537|ref|XP_012090912.1|PREDICTED: protein SMG9-like [Jatropha curcas]gi|643705154|gb|KDP21771.1|hypothetical protein JCGZ_00558 [Jatropha curcas]	XP_012090912, KDP21771	0.0	412	336
Pe101O4.16		-	1	910	507	Transcription factor IBH1-like	168	gi|566204322|ref|XP_002320449.2|hypothetical protein POPTR_0014s14730g [Populus trichocarpa]gi|550324217|gb|EEE98764.2|hypothetical protein POPTR_0014s14730g [Populus trichocarpa]	XP_002320449, EEE98764	3.77E-48	173	123
Pe101O4.17		-	1	2098	1479	UDP-glycosyltransferase 92A1-like	492	gi|743918365|ref|XP_011003186.1|PREDICTED: UDP-glycosyltransferase 92A1-like [Populus euphratica]	XP_011003186	0.0	504	404
Pe101O4.18		-	3	1854	477	CRIB domain-containing RIC4-like	166	gi|566159597|ref|XP_002301646.2|ROP-INTERACTIVE CRIB MOTIF-CONTAINING protein 2 [Populus trichocarpa]gi|550345671|gb|EEE80919.2|ROP-INTERACTIVE CRIB MOTIF-CONTAINING protein 2 [Populus trichocarpa]	XP_002301646, EEE80919	2.09E-48	177	124
Pe101O4.19		+	2	1106	813	Hypothetical protein DCAR_017700	270	gi|1021036674|gb|KZM94457.1|hypothetical protein DCAR_017700 [Daucus carota subsp. sativus]	KZM94457	1.09E-40	220	124
Pe101O4.20		-	3	1142	822	Nuclease HARBI1	273	gi|590622104|ref|XP_007024956.1|Uncharacterized protein TCM_029406 [Theobroma cacao]gi|508780322|gb|EOY27578.1|Uncharacterized protein TCM_029406 [Theobroma cacao]	XP_007024956, EOY27578	2.25E-81	230	163
Pe101O4.21		+	4	2103	1122	15-cis-zeta-carotene chloroplastic	373	gi|823165010|ref|XP_012482446.1|PREDICTED: 15-cis-zeta-carotene isomerase, chloroplastic [Gossypium raimondii]gi|763761786|gb|KJB29040.1|hypothetical protein B456_005G080900 [Gossypium raimondii]	XP_012482446, KJB29040	0.0	379	310
Pe101O4.22		+	5	2325	393	Eukaryotic translation initiation factor 3 subunit H	177	gi|743940375|ref|XP_011014658.1|PREDICTED: eukaryotic translation initiation factor 3 subunit H-like [Populus euphratica]	XP_011014658	5.04E-73	131	123
Pe101P7.1	Incomplete 3'	-	11	4952	1452	Nuclear control of atpase 2	484	gi|641847537|gb|KDO66417.1|hypothetical protein CISIN_1g007748mg [Citrus sinensis]	KDO66417	0.0	484	381
Pe101P7.2	2 isoforms	-	14	4522	1275	Tryptophan tyrosine permease isoform 1 [Theobroma cacao]	489	gi|743875800|ref|XP_011035035.1|PREDICTED: uncharacterized protein LOC105132981 isoform X1 [Populus euphratica]	XP_011035035	0.0	484	413

Pe101P7.3		+	11	4304	1170	4-diphosphocytidyl-2-C-methyl-D-erythritol kinase	389	gi|164604994|dbj|BAF98293.1|4-(cytidine 5'-diphospho)-2-C-methyl-D-erythritol kinase [Hevea brasiliensis]gi|208429108|gb|ACI26723.1|4-diphosphocytidyl-2C-methyl-D-erythritol kinase [Hevea brasiliensis]	BAF98293, ACI26723	0.0	386	330
Pe101P7.4-5		-	2	1961	1938	Ubiquitin-associated domain-containing family [Populus trichocarpa]	319	gi|743897449|ref|XP_011042014.1|PREDICTED: cyclin-dependent kinase 12-like [Populus euphratica]	XP_011042014	2.16E-103	321	243
Pe101P7.6	1 isoform	+	6	3324	678	Elongation factor 1-beta 2-like	225	gi|593691235|ref|XP_007146187.1|hypothetical protein PHAVU_006G019800g [Phaseolus vulgaris]gi|561019410|gb|ESW18181.1|hypothetical protein PHAVU_006G019800g [Phaseolus vulgaris]	XP_007146187, ESW18181	1.25E-107	225	195
Pe101P7.7		+	1	1455	1455	Zinc finger family [Populus trichocarpa]	484	gi|743927265|ref|XP_011007811.1|PREDICTED: uncharacterized protein LOC105113357 [Populus euphratica]	XP_011007811	1.19E-36	484	217
Pe101P7.8		-	6	2323	1347	Kinase chloroplastic-like	448	gi|645218601|ref|XP_008231474.1|PREDICTED: protein kinase 2A, chloroplastic-like [Prunus mume]	XP_008231474	0.0	451	327
Pe101P7.9		+	16	4798	1704	CBL-interacting serine threonine- kinase 3 [Jatropha curcas]	444	gi|223527690|gb|EEF29798.1|CBL-interacting serine/threonine-protein kinase, putative [Ricinus communis]	EEF29798	0.0	450	428
Pe101P7.10		-	6	1603	633	Oligoribonuclease isoform X2	210	gi|802616771|ref|XP_012075386.1|PREDICTED: oligoribonuclease [Jatropha curcas]gi|643726429|gb|KDP35136.1|hypothetical protein JCGZ_10670 [Jatropha curcas]	XP_012075386, KDP35136	4.96E-82	188	151
Pe101P7.11		+	2	3240	450	Calmodulin-7 [Camelina sativa]	149	gi|359479174|ref|XP_003632230.1|PREDICTED: calmodulin-7 isoform X1 [Vitis vinifera]	XP_003632230	2.58E-98	149	149
Pe101P7.12		-	3	3358	792	ABA-responsive element binding 3 isoform 1 [Theobroma cacao]	268	gi|743919650|ref|XP_011003851.1|PREDICTED: ABSCISIC ACID-INSENSITIVE 5-like protein 2 isoform X1 [Populus euphratica]gi|743919652|ref|XP_011003852.1|PREDICTED: ABSCISIC ACID-INSENSITIVE 5-like protein 2 isoform X1 [Populus euphratica]gi|743919654|ref|XP_011003853.1|PREDICTED: ABSCISIC ACID-INSENSITIVE 5-like protein 2 isoform X2 [Populus euphratica]	XP_011003851, XP_011003852, XP_011003853	6.39E-130	276	223
Pe101P7.13		-	1	234	234	---Na---	77	No Blast Hit				
Pe101P7.14		-	3	744	351	---Na---	116	No Blast Hit				
Pe101P7.15	1 isoform	+	7	4989	732	Probable ATP synthase 24 kda mitochondrial	243	gi|694416489|ref|XP_009336369.1|PREDICTED: probable ATP synthase 24 kDa subunit, mitochondrial [Pyrus x bretschneideri]	XP_009336369	1.07E-117	243	213
Pe101P7.16		-	7	5296	1758	Pentatricopeptide repeat-containing At3g59040	585	gi|743923882|ref|XP_011006049.1|PREDICTED: pentatricopeptide repeat-containing protein At3g59040 [Populus euphratica]	XP_011006049	0.0	599	501
Pe101P7.17	2 isoforms	+	1	4500	1434	UBP1-associated 2B-like [Gossypium hirsutum]	477	gi|1028991299|ref|XP_016750489.1|PREDICTED: UBP1-associated protein 2B-like [Gossypium hirsutum]gi|1028991302|ref|XP_016750490.1|PREDICTED: UBP1-associated protein 2B-like [Gossypium hirsutum]gi|1028991305|ref|XP_016750491.1|PREDICTED: UBP1-associated protein 2B-like [Gossypium hirsutum]	XP_016750489, XP_016750490, XP_016750491	2.13E-161	399	314
Pe101P7.18		+	15	6158	1380	DNA primase small subunit	450	gi|590679147|ref|XP_007040498.1|DNA primase isoform 1 [Theobroma cacao]gi|508777743|gb|EOY24999.1|DNA	XP_007040498, EOY24999	0.0	453	403

								primase isoform 1 [Theobroma cacao]				
Pe101P7.19	1 isoform	+	15	8484	2712	La-related 1A	903	gi|566185915|ref|XP_002313496.2|hypothetical protein POPTR_0009s02330g [Populus trichocarpa]gi|550330877|gb|EEE87451.2|hypothetical protein POPTR_0009s02330g [Populus trichocarpa]	XP_002313496, EEE87451	0.0	935	711
Pe101P7.20		-	20	4855	2208	Argonaute 4-like [Pyrus x bretschneideri]	735	gi|743923892|ref|XP_011006053.1|PREDICTED: protein argonaute 4-like [Populus euphratica]gi|743923894|ref|XP_011006054.1|PREDICTED: protein argonaute 4-like [Populus euphratica]	XP_011006053, XP_011006054	0.0	734	702
Pe101P13.1		-	9	8367	1656	Ferredoxin-dependent glutamate chloroplastic	507	gi|802688163|ref|XP_012082579.1|PREDICTED: ferredoxin-dependent glutamate synthase, chloroplastic isoform X1 [Jatropha curcas]gi|643717826|gb|KDP29269.1|hypothetical protein JCGZ_16658 [Jatropha curcas]	XP_012082579, KDP29269	0.0	465	437
Pe101P13.2		-	2	1593	1458	Ribonuclease H At1g65750 family	485	gi|1012365680|gb|KYP76862.1|Putative ribonuclease H protein At1g65750, partial [Cajanus cajan]	KYP76862	9.53E-81	480	254
Pe101P13.3		-	8	3102	2307	Phosphatidylinositol 4-phosphate 5-kinase 4	768	gi|255571949|ref|XP_002526916.1|PREDICTED: phosphatidylinositol 4-phosphate 5-kinase 4 [Ricinus communis]gi|223533735|gb|EEF35469.1|phosphatidylinositol-4-phosphate 5-kinase, putative [Ricinus communis]	XP_002526916, EEF35469	0.0	770	699
Pe101P13.4	1 isoform	-	1	3198	1680	Phosphatidylinositol 4-kinase gamma 8 [Ricinus communis]	559	gi|223537068|gb|EEF38703.1|inositol or phosphatidylinositol kinase, putative [Ricinus communis]	EEF38703	0.0	576	419
Pe101P13.5	2 isoforms	-	3	1537	447	Heavy metal-associated isoprenylated plant 26	148	gi|802754289|ref|XP_012088680.1|PREDICTED: heavy metal-associated isoprenylated plant protein 26 [Jatropha curcas]	XP_012088680	4.41E-85	148	135
Pe101P13.6	6 isoforms	-	12	10555	1245	Lysosomal amino acid transporter 1 [Populus euphratica]	414	gi|743894794|ref|XP_011040657.1|PREDICTED: lysosomal amino acid transporter 1 [Populus euphratica]gi|743894796|ref|XP_011040658.1|PREDICTED: lysosomal amino acid transporter 1 [Populus euphratica]gi|743894798|ref|XP_011040659.1|PREDICTED: lysosomal amino acid transporter 1 [Populus euphratica]gi|743894800|ref|XP_011040660.1|PREDICTED: lysosomal amino acid transporter 1 [Populus euphratica]	XP_011040657, XP_011040658, XP_011040659, XP_011040660	0.0	416	318
Pe101P13.7		+	5	2125	720	Rho GDP-dissociation inhibitor 1-like	239	gi|743914156|ref|XP_011000997.1|PREDICTED: rho GDP-dissociation inhibitor 1-like [Populus euphratica]	XP_011000997	2.04E-121	245	210
Pe101P13.8	1 isoform	-	12	7602	1362	Polyglutamine-binding 1 [Citrus sinensis]	453	gi|566174209|ref|XP_002308857.2|hypothetical protein POPTR_0006s03080g [Populus trichocarpa]gi|550335346|gb|EEE92380.2|hypothetical protein POPTR_0006s03080g [Populus trichocarpa]	XP_002308857, EEE92380	0.0	440	357
Pe101P13.9	1 isoform	-	21	10658	2187	DNA gyrase subunit chloroplastic mitochondrial-like	728	gi|802688098|ref|XP_012082559.1|PREDICTED: DNA gyrase subunit B, chloroplastic/mitochondrial-like [Jatropha curcas]	XP_012082559	0.0	737	653
Pe101P13.10		-	2	971	336	60S ribosomal L36-3-like [Elaeis guineensis]	111	gi|514784185|ref|XP_004970529.1|PREDICTED: 60S ribosomal protein L36-3-like [Setaria italica]gi|944243354|gb|KQL07662.1|hypothetica	XP_004970529, KQL07662, KQL07663	3.45E-51	111	99

								l protein SETIT_003417mg [Setaria italica]gi|944243355|gb|KQL07663.1|hypothetical protein SETIT_003417mg [Setaria italica]				
Pe101P13.11		-	5	3245	1677	DNA binding [Ricinus communis]	558	gi|743913450|ref|XP_011000637.1|PREDICTED: uncharacterized protein LOC105108141 [Populus euphratica]gi|743913452|ref|XP_011000638.1|PREDICTED: uncharacterized protein LOC105108141 [Populus euphratica]	XP_011000637, XP_011000638	8.68E-103	228	194
Pe101P13.12		+	8	3442	1032	Choline-phosphate cytidylyltransferase 1	343	gi|743914233|ref|XP_011001038.1|PREDICTED: choline-phosphate cytidylyltransferase 1 [Populus euphratica]	XP_011001038	8.20E-174	285	257
Pe101P13.13-14		-	15	13552	1833	Cytochrome P450 chloroplastic	427	gi|255559122|ref|XP_002520583.1|PREDICTED: cytochrome P450 97B2, chloroplastic [Ricinus communis]gi|223540243|gb|EEF41816.1|cytochrome P450, putative [Ricinus communis]	XP_002520583, EEF41816	0.0	426	400
Pe101P13.15		-	1	1361	1116	F-box CPR30-like	371	gi|566173882|ref|XP_002307880.2|hypothetical protein POPTR_0006s01300g [Populus trichocarpa]gi|550335210|gb|EEE91403.2|hypothetical protein POPTR_0006s01300g [Populus trichocarpa]	XP_002307880, EEE91403	3.07E-146	363	278
Pe101P13.16		-	1	2046	753	Proteasome subunit alpha type-4-like [Ziziphus jujuba]	250	gi|823185034|ref|XP_012489395.1|PREDICTED: proteasome subunit alpha type-4 [Gossypium raimondii]gi|823185037|ref|XP_012489396.1|PREDICTED: proteasome subunit alpha type-4 [Gossypium raimondii]gi|1029069721|ref|XP_016695077.1|PREDICTED: proteasome subunit alpha type-4 [Gossypium hirsutum]gi|1029101721|ref|XP_016710225.1|PREDICTED: proteasome subunit alpha type-4 [Gossypium hirsutum]gi|763773407|gb|KJB40530.1|hypothetical protein B456_007G068200 [Gossypium raimondii]	XP_012489395, XP_012489396, XP_016695077, XP_016710225, KJB40530	6.58E-174	250	247
Pe101P13.17		+	2	666	210	Hypothetical protein PHAVU_011G024400g [Phaseolus vulgaris]	69	gi|593115821|ref|XP_007131573.1|hypothetical protein PHAVU_011G024400g [Phaseolus vulgaris]gi|561004573|gb|ESW03567.1|hypothetical protein PHAVU_011G024400g [Phaseolus vulgaris]	XP_007131573, ESW03567	5.85E-22	66	59
Pe101P13.18	Incomplete 3'	+	1	1423	1423	Ubiquitin- ligase [Populus trichocarpa]	474	gi|802708653|ref|XP_012084519.1|PREDICTED: E3 ubiquitin-protein ligase UPL5 [Jatropha curcas]gi|643715412|gb|KDP27496.1|hypothetical protein JCGZ_20228 [Jatropha curcas]	XP_012084519, KDP27496	0.0	474	366
Pe103M2.1		-	3	1413	1233	Nuclease HARBI1	410	gi|590668541|ref|XP_007037522.1|Uncharacterized protein TCM_014176 [Theobroma cacao]gi|508774767|gb|EOY22023.1|Uncharacterized protein TCM_014176 [Theobroma cacao]	XP_007037522, EOY22023	2.51E-127	379	261
Pe103M2.2		+	17	6962	2010	Alpha-L-arabinofuranosidase 1-like [Gossypium hirsutum]	669	gi|566208326|ref|XP_002322584.2|hypothetical protein POPTR_0016s02620g [Populus trichocarpa]gi|550320669|gb|EEF04345.2|hypothetical protein POPTR_0016s02620g [Populus trichocarpa]	XP_002322584, EEF04345	0.0	675	597
Pe103M2.3		-	2	1861	444	50S ribosomal chloroplastic-like	147	gi|449453131|ref|XP_004144312.1|PREDICTED: 50S ribosomal protein 5, chloroplastic [Cucumis sativus]gi|700199508|gb|KGN54666.1|hypothetical protein Csa_4G419580 [Cucumis sativus]	XP_004144312, KGN54666	6.63E-25	157	100

Pe103M2.4		-	2	549	429	50S ribosomal chloroplastic-like [Jatropha curcas]	142	gi|224089881|ref|XP_002308849.1|hypothetical protein POPTR_0006s02920g [Populus trichocarpa]gi|222854825|gb|EEE92372.1|hypothetical protein POPTR_0006s02920g [Populus trichocarpa]	XP_002308849, EEE92372	2.59E-07	111	62
Pe103M2.5	1 isoform	-	14	12656	1330	Aladin isoform X2	442	gi|118486469|gb|ABK95074.1|unknown [Populus trichocarpa]	ABK95074	0.0	449	390
Pe103M2.7		+	4	1011	486	---Na---	161	No Blast Hit				
Pe103M2.8		+	1	222	222	---Na---	73	No Blast Hit				
Pe103M2.9		-	1	303	303	---Na---	100	No Blast Hit				
Pe108C16.1		+	3	2520	1104	Uncharacterized LOC8278573 precursor [Ricinus communis]	367	gi|1021311980|ref|NP_001310676.1|uncharacterized LOC8278573 precursor [Ricinus communis]gi|1621268|emb|CAB02653.1|unknown [Ricinus communis]gi|223545501|gb|EEF47006.1|conserved hypothetical protein [Ricinus communis]	NP_001310676, CAB02653, EEF47006	0.0	364	334
Pe108C16.2		+	5	1502	597	RAN2 small Ras GTP-binding nuclear	149	gi|296081996|emb|CBI21001.3|unnamed protein product [Vitis vinifera]	CBI21001	4.18E-50	151	108
Pe108C16.3		-	4	1111	471	---Na---	156	No Blast Hit				
Pe108C16.4		-	1	2568	2568	Pentatricopeptide repeat-containing mitochondrial-like isoform X1 [Vitis vinifera]	855	gi|566178307|ref|XP_002309609.2|pentatricopeptide repeat-containing family protein [Populus trichocarpa]gi|550337148|gb|EEE93132.2|pentatricopeptide repeat-containing family protein [Populus trichocarpa]	XP_002309609, EEE93132	0.0	852	666
Pe108C16.5	1 isoform	-	10	3991	1161	tRNA (cytosine(38)-C(5))-methyltransferase-like	386	gi|802627241|ref|XP_012076609.1|PREDICTED: tRNA (cytosine-5-)-methyltransferase isoform X1 [Jatropha curcas]	XP_012076609	0.0	385	319
Pe108C16.6		+	1	1141	930	U-box domain-containing 12-like isoform X1 [Nicotiana sylvestris]	309	gi|743814916|ref|XP_011019814.1|PREDICTED: U-box domain-containing protein 10-like [Populus euphratica]	XP_011019814	1.17E-166	309	279
Pe108C16.7		+	9	3643	771	Acyl- thioesterase 2-like [Populus euphratica]	256	gi|743841444|ref|XP_011026469.1|PREDICTED: acyl-protein thioesterase 2-like [Populus euphratica]gi|743841448|ref|XP_011026470.1|PREDICTED: acyl-protein thioesterase 2-like [Populus euphratica]gi|743841452|ref|XP_011026471.1|PREDICTED: acyl-protein thioesterase 2-like [Populus euphratica]gi|743841458|ref|XP_011026472.1|PREDICTED: acyl-protein thioesterase 2-like [Populus euphratica]	XP_011026469, XP_011026470, XP_011026471, XP_011026472	2.14E-159	256	235
Pe108C16.8	1 isoform	-	8	4808	2013	Tesmin TSO1-like CXC domain-containing isoform 1 [Theobroma cacao]	670	gi|802627270|ref|XP_012076620.1|PREDICTED: CRC domain-containing protein TSO1-like isoform X1 [Jatropha curcas]gi|643724425|gb|KDP33626.1|hypothetical protein JCGZ_07197 [Jatropha curcas]	XP_012076620, KDP33626	1.18E-140	650	371
Pe108C16.9		+	2	967	468	Zinc finger family [Populus trichocarpa]	170	gi|802627276|ref|XP_012076622.1|PREDICTED: zinc finger Ran-binding domain-containing protein 2 [Jatropha curcas]gi|643724426|gb|KDP33627.1|hypothetical protein JCGZ_07198 [Jatropha curcas]	XP_012076622, KDP33627	8.61E-74	151	133
Pe108C16.10		-	3	829	648	PREDICTED: uncharacterized protein LOC105637663 [Jatropha curcas]	215	gi|802627228|ref|XP_012076604.1|PREDICTED: uncharacterized protein LOC105637663 [Jatropha curcas]gi|643724415|gb|KDP33616.1|hypothetica	XP_012076604, KDP33616	6.69E-122	216	200

								l protein JCGZ_07187 [Jatropha curcas]				
Pe108C16.11		+	4	2188	1026	Overexpressor of cationic peroxidase 3	341	gi|566178324|ref|XP_002308655.2|hypothetical protein POPTR_0006s26790g [Populus trichocarpa]gi|550337156|gb|EEE92178.2|hypothetical protein POPTR_0006s26790g [Populus trichocarpa]	XP_002308655, EEE92178	1.73E-103	328	243
Pe108C16.12		+	1	652	285	Overexpressor of cationic peroxidase [Theobroma cacao]	102	gi|566213666|ref|XP_002324288.2|hypothetical protein POPTR_0018s01520g [Populus trichocarpa]gi|550317796|gb|EEF02853.2|hypothetical protein POPTR_0018s01520g [Populus trichocarpa]	XP_002324288, EEF02853	8.93E-20	97	65
Pe108C16.13		+	1	1767	1767	Pentatricopeptide repeat-containing At3g56550 [Cucumis melo]	588	gi|566208377|ref|XP_002323212.2|pentatricopeptide repeat-containing family protein [Populus trichocarpa]gi|550320693|gb|EEF04973.2|pentatricopeptide repeat-containing family protein [Populus trichocarpa]	XP_002323212, EEF04973	0.0	590	483
Pe108C16.14		+	4	1935	513	bZIP with a Ring-finger motif family [Populus trichocarpa]	170	gi|224141881|ref|XP_002324289.1|bZIP with a Ring-finger motif family protein [Populus trichocarpa]gi|222865723|gb|EEF02854.1|bZIP with a Ring-finger motif family protein [Populus trichocarpa]	XP_002324289, EEF02854	1.22E-74	168	149
Pe108C16.15		+	14	6553	3252	Cellulose synthase A catalytic subunit 1 [UDP-forming]	1083	gi|802627222|ref|XP_012076601.1|PREDICTED: cellulose synthase A catalytic subunit 1 [UDP-forming] [Jatropha curcas]gi|643724411|gb|KDP33612.1|hypothetical protein JCGZ_07183 [Jatropha curcas]	XP_012076601, KDP33612	0.0	1084	1055
Pe108C16.16	1 isoform	+	5	1890	405	Alba DNA RNA-binding [Theobroma cacao]	134	gi|802627219|ref|XP_012076600.1|PREDICTED: uncharacterized protein At2g34160-like [Jatropha curcas]gi|643724410|gb|KDP33611.1|hypothetical protein JCGZ_07182 [Jatropha curcas]	XP_012076600, KDP33611	7.07E-70	134	122
Pe108C16.17		+	3	789	399	EG45-like domain containing	132	gi|743841383|ref|XP_011026456.1|PREDICTED: EG45-like domain containing protein 2 [Populus euphratica]	XP_011026456	2.52E-65	132	115
Pe108C16.18		-	5	2184	1047	Lactation elevated 1 isoform X1	348	gi|1000974524|ref|XP_015572524.1|PREDICTED: putative ATPase N2B [Ricinus communis]	XP_015572524	1.66E-26	81	72
Pe108C16.19		+	1	234	234	---Na---	77	No Blast Hit				
Pe108C16.20		+	1	282	282	---Na---	93	No Blast Hit				
Pe108C16.21		+	2	1949	1896	Gag protease poly [Theobroma cacao]	631	gi|590728434|ref|XP_007099662.1|Gag protease polyprotein-like protein [Theobroma cacao]gi|508728474|gb|EOY20371.1|Gag protease polyprotein-like protein [Theobroma cacao]	XP_007099662, EOY20371	3.61E-88	482	258
Pe108C16.22		+	1	384	384	Gag protease poly [Theobroma cacao]	127	gi|658064690|ref|XP_008368288.1|PREDICTED: uncharacterized protein LOC103431892 [Malus domestica]	XP_008368288	2.85E-26	88	69
Pe108C16.23		+	1	666	666	Gag protease poly [Theobroma cacao]	221	gi|590600561|ref|XP_007019489.1|Gag protease polyprotein [Theobroma cacao]gi|508724817|gb|EOY16714.1|Gag protease polyprotein [Theobroma cacao]	XP_007019489, EOY16714	9.61E-13	151	83
Pe108C16.24		+	2	867	510	---Na---	169	No Blast Hit				
Pe113A7.1		+	1	2075	630	YLS9-like [Populus euphratica]	209	gi|802611158|ref|XP_012074337.1|PREDICTED: protein YLS9-like [Jatropha curcas]gi|643727834|gb|KDP36127.1|hypothetical protein JCGZ_08771 [Jatropha curcas]	XP_012074337, KDP36127	1.90E-118	209	187

Pe113A7.2		+	2	2254	678	Retrotransposon Ty1-copia subclass	225	gi|823155331|ref|XP_012477568.1|PREDICTED : uncharacterized protein LOC105793188 [Gossypium raimondii]	XP_012477568	3.16E-52	200	139
Pe113A7.4		-	2	470	372	---Na---	123	No Blast Hit				
Pe113A7.5		+	1	1079	681	Syringolide-induced B13-1-9 [Populus trichocarpa]	226	gi|590632796|ref|XP_007027942.1|Late embryogenesis abundant (LEA) hydroxyproline-rich glycoprotein family, putative [Theobroma cacao]gi|508716547|gb|EOY08444.1|Late embryogenesis abundant (LEA) hydroxyproline-rich glycoprotein family, putative [Theobroma cacao]	XP_007027942, EOY08444	7.33E-97	228	191
Pe113A7.3		-	1	156	156	---Na---	51	No Blast Hit				
Pe117C17.1		+	2	422	333	---Na---	110	No Blast Hit				
Pe117C17.2		-	1	402	402	---Na---	133	No Blast Hit				
Pe117C17.3		+	1	279	279	---Na---	92	No Blast Hit				
Pe117C17.4		-	1	294	294	Gag protease poly [Theobroma cacao]	97	gi|590693615|ref|XP_007044383.1|DNA/RNA polymerases superfamily protein [Theobroma cacao]gi|508708318|gb|EOY00215.1|DNA/RNA polymerases superfamily protein [Theobroma cacao]	XP_007044383, EOY00215	1.12E+00	84	38
Pe117C17.5		-	1	225	225	---Na---	74	No Blast Hit				
Pe117C17.6		-	1	228	228	---Na---	75	No Blast Hit				
Pe117C17.7		-	1	291	291	---Na---	96	No Blast Hit				
Pe117C17.8		-	2	431	348	---Na---	115	No Blast Hit				
Pe117C17.9		+	1	495	495	Gag protease poly [Theobroma cacao]	164	gi|590691787|ref|XP_007043880.1|Gag protease polyprotein [Theobroma cacao]gi|508707815|gb|EOX99711.1|Gag protease polyprotein [Theobroma cacao]	XP_007043880, EOX99711	4.00E-01	107	55
Pe117C17.10		+	1	153	153	---Na---	50	No Blast Hit				
Pe117C17.11		-	5	2489	849	---Na---	282	No Blast Hit				
Pe117C17.12		+	1	264	264	---Na---	87	No Blast Hit				
Pe117C17.13		-	12	6852	1188	RNA-binding Luc7-like 2	363	gi|593332569|ref|XP_007139710.1|hypothetical protein PHAVU_008G052700g [Phaseolus vulgaris]gi|561012843|gb|ESW11704.1|hypothetical protein PHAVU_008G052700g [Phaseolus vulgaris]	XP_007139710, ESW11704	0.0	364	331
Pe117C17.14		-	2	2344	774	Retrotransposon Ty1-copia subclass	257	gi|923839654|ref|XP_013700463.1|PREDICTED : uncharacterized protein LOC106404280 [Brassica napus]	XP_013700463	1.57E-49	223	146
Pe117C17.15		+	1	183	183	---Na---	60	No Blast Hit				
Pe117C17.16		-	1	321	321	---Na---	106	No Blast Hit				
Pe123N8.1	1 isoform	+	12	4941	819	Secretory carrier membrane family [Populus trichocarpa]	272	gi|743809187|ref|XP_011018450.1|PREDICTED : secretory carrier-associated membrane protein 4-like [Populus euphratica]	XP_011018450	3.97E-174	272	256
Pe123N8.2		+	5	3093	1230	Phosphoribulokinase family [Populus trichocarpa]	409	gi|743809191|ref|XP_011018451.1|PREDICTED : phosphoribulokinase, chloroplastic-like [Populus euphratica]	XP_011018451	0.0	408	394
Pe123N8.3		-	8	4049	1560	Plastidal glycolate glycerate translocator	519	gi|255555937|ref|XP_002519004.1|PREDICTED : plastidal glycolate/glycerate translocator 1,	XP_002519004, EEF43537	0.0	517	449

						chloroplastic		chloroplastic [Ricinus communis]gi|223541991|gb|EEF43537.1|conserved hypothetical protein [Ricinus communis]				
Pe123N8.4	3 isoforms	-	6	3559	1674	4-coumarate-- ligase-like 6	557	gi|1000966856|ref|XP_015574447.1|PREDICTED: LOW QUALITY PROTEIN: 4-coumarate--CoA ligase-like 6 [Ricinus communis]	XP_015574447	0.0	560	461
Pe123N8.5	1 isoform	-	10	4655	1095	SWR1 complex subunit 2 [Populus euphratica]	364	gi|1009123617|ref|XP_015878633.1|PREDICTED: SWR1 complex subunit 2 isoform X3 [Ziziphus jujuba]gi|1009123619|ref|XP_015878634.1|PREDICTED: SWR1 complex subunit 2 isoform X3 [Ziziphus jujuba]	XP_015878633, XP_015878634	4.14E-149	348	288
Pe123N8.6		-	11	5485	2397	Csc1 at1g32090	798	gi|802537967|ref|XP_012066469.1|PREDICTED: CSC1-like protein At1g32090 [Jatropha curcas]gi|643741153|gb|KDP46674.1|hypothetical protein JCGZ_12198 [Jatropha curcas]	XP_012066469, KDP46674	0.0	798	755
Pe123N8.7		+	2	2227	1677	PREDICTED: uncharacterized protein LOC105121672	558	gi|743810259|ref|XP_011018700.1|PREDICTED: uncharacterized protein LOC105121672 [Populus euphratica]	XP_011018700	0.0	551	417
Pe123N8.8		+	4	2826	522	DNA (cytosine-5)-methyltransferase CMT2-like	173	gi|566161862|ref|XP_002303456.2|hypothetical protein POPTR_0003s09910g [Populus trichocarpa]gi|550342851|gb|EEE78435.2|hypothetical protein POPTR_0003s09910g [Populus trichocarpa]	XP_002303456, EEE78435	1.94E-62	173	132
Pe123N8.9		-	22	10051	2214	ENHANCED DISEASE RESISTANCE 2 isoform X1	737	gi|255557651|ref|XP_002519855.1|PREDICTED: protein ENHANCED DISEASE RESISTANCE 2 [Ricinus communis]gi|223540901|gb|EEF42459.1|lipid binding protein, putative [Ricinus communis]	XP_002519855, EEF42459	0.0	727	699
Pe123N8.10		-	1	253	195	---Na---	75	No Blast Hit				
Pe123N8.11		+	1	893	252	---Na---	83	No Blast Hit				
Pe123N8.12		-	1	375	375	---Na---	124	No Blast Hit				
Pe123N8.13		+	1	267	267	---Na---	88	No Blast Hit				
Pe123N8.14		+	2	1245	990	DNA RNA polymerases superfamily [Theobroma cacao]	329	gi|590580431|ref|XP_007014066.1|Uncharacterized protein TCM_038736 [Theobroma cacao]gi|508784429|gb|EOY31685.1|Uncharacterized protein TCM_038736 [Theobroma cacao]	XP_007014066, EOY31685	8.20E-34	329	154
Pe123N8.15	Incomplete 3'	+	1	163	163	---Na---	54	No Blast Hit				
Pe125I23.1	3 isoforms	+	8	7993	1776	WVD2-like 7 isoform X1 [Gossypium hirsutum]	537	gi|1000964317|ref|XP_015575288.1|PREDICTED: uncharacterized protein LOC8270369 [Ricinus communis]gi|1000964320|ref|XP_015575289.1|PREDICTED: uncharacterized protein LOC8270369 [Ricinus communis]	XP_015575288, XP_015575289	1.88E-154	542	354
Pe125I23.2		-	2	1456	1359	Formin 4	452	gi|566184928|ref|XP_002312698.2|formin homology 2 domain-containing family protein [Populus trichocarpa]gi|550333485|gb|EEE90065.2|formin homology 2 domain-containing family protein [Populus trichocarpa]	XP_002312698, EEE90065	0.0	452	375
Pe125I23.3		-	1	1089	1089	Formin homology 2 domain-containing family [Populus trichocarpa]	362	gi|566184928|ref|XP_002312698.2|formin homology 2 domain-containing family protein [Populus trichocarpa]gi|550333485|gb|EEE90065.2|formin homology 2 domain-containing family protein	XP_002312698, EEE90065	4.89E-38	276	164

								[Populus trichocarpa]				
Pe125I23.4		-	4	2173	786	Two-component response regulator ARR5-like	261	gi|255558348|ref|XP_002520201.1|PREDICTED : two-component response regulator ARR5 [Ricinus communis]gi|223540693|gb|EEF42256.1|two-component sensor protein histidine protein kinase, putative [Ricinus communis]	XP_002520201, EEF42256	7.53E-99	267	202
Pe125I23.5		-	1	414	414	Zinc finger A20 and AN1 domain-containing stress-associated 8	137	gi|1000984672|ref|XP_015579171.1|PREDICTED: putative zinc finger A20 and AN1 domain-containing stress-associated protein 8 [Ricinus communis]	XP_015579171	2.32E-36	144	96
Pe125I23.6		+	7	3435	1599	Zinc finger MYND domain-containing 15 isoform 1 [Theobroma cacao]	532	gi|802787836|ref|XP_012092023.1|PREDICTED : uncharacterized protein LOC105649827 [Jatropha curcas]gi|643704222|gb|KDP21286.1|hypothetical protein JCGZ_21757 [Jatropha curcas]	XP_012092023, KDP21286	0.0	527	488
Pe125I23.7		-	4	1561	975	Non-functional NADPH-dependent codeinone reductase 2-like	324	gi|743936700|ref|XP_011012738.1|PREDICTED : non-functional NADPH-dependent codeinone reductase 2-like [Populus euphratica]	XP_011012738	0.0	325	287
Pe125I23.8		+	1	2092	1116	Metalloendo ase 1-like	371	gi|255558368|ref|XP_002520211.1|PREDICTED : metalloendoproteinase 2-MMP [Ricinus communis]gi|223540703|gb|EEF42266.1|Metallo endoproteinase 1 precursor, putative [Ricinus communis]	XP_002520211, EEF42266	1.34E-166	366	290
Pe125I23.9		+	1	1254	1254	Vegetative incompatibility HET-E-1-like	417	gi|359473323|ref|XP_003631291.1|PREDICTED : vegetative incompatibility protein HET-E-1 [Vitis vinifera]	XP_003631291	0.0	431	330
Pe125I23.10		+	1	723	723	Zinc finger 5	240	gi|1000964177|ref|XP_015575264.1|PREDICTED: zinc finger protein 5 [Ricinus communis]	XP_015575264	1.07E-94	247	191
Pe125I23.11		+	5	2849	1329	IAA-amino acid hydrolase ILR1-like 4	442	gi|255558378|ref|XP_002520216.1|PREDICTED : IAA-amino acid hydrolase ILR1-like 1 [Ricinus communis]gi|223540708|gb|EEF42271.1|IAA-amino acid hydrolase ILR1 precursor, putative [Ricinus communis]	XP_002520216, EEF42271	0.0	421	371
Pe125I23.12	2 isoforms	+	14	4189	1608	DNA polymerase beta isoform X1 [Cucumis melo]	535	gi|802787894|ref|XP_012092039.1|PREDICTED : DNA polymerase beta [Jatropha curcas]gi|643704237|gb|KDP21301.1|hypothetical protein JCGZ_21772 [Jatropha curcas]	XP_012092039, KDP21301	0.0	538	457
Pe125I23.13		+	9	3444	1113	Rnase H family isoform 2 [Theobroma cacao]	370	gi|566190132|ref|XP_002314727.2|hypothetical protein POPTR_0010s10515g [Populus trichocarpa]gi|550329518|gb|EEF00898.2|hypothetical protein POPTR_0010s10515g [Populus trichocarpa]	XP_002314727, EEF00898	2.15E-149	378	279
Pe125I23.14	1 isoform	-	4	1895	1254	RNA-binding family isoform 1 [Theobroma cacao]	417	gi|1000942603|ref|XP_015582212.1|PREDICTED: uncharacterized protein LOC8281932 isoform X2 [Ricinus communis]	XP_015582212	6.00E-91	455	282
Pe125I23.15		+	6	3666	1002	Cyclin delta-1 family [Populus trichocarpa]	336	gi|359359230|gb|AEV41133.1|D1-type cyclin [Populus x canadensis]	AEV41133	2.68E-172	331	297
Pe125I23.16		-	3	1085	531	Serine threonine- kinase TAO3 [Theobroma cacao]	176	gi|590694181|ref|XP_007044537.1|Serine/threonine-protein kinase TAO3 [Theobroma cacao]gi|508708472|gb|EOY00369.1|Serine/threonine-protein kinase TAO3 [Theobroma cacao]	XP_007044537, EOY00369	2.75E-77	176	139
Pe125I23.17	2 isoforms	-	14	4579	1602	Nucleobase-ascorbate transporter 7	533	gi|802787942|ref|XP_012092049.1|PREDICTED : nucleobase-ascorbate transporter 7 [Jatropha curcas]gi|643704245|gb|KDP21309.1|hypothetical protein JCGZ_21780 [Jatropha curcas]	XP_012092049, KDP21309	0.0	523	508
Pe125I23.18		-	5	2957	492	LSM12 homolog A	180	gi|802787946|ref|XP_012092050.1|PREDICTED	XP_012092050,	8.77E-111	180	170

								: protein LSM12 homolog A-like [Jatropha curcas]gi|643704246|gb|KDP21310.1|hypothetical protein JCGZ_21781 [Jatropha curcas]	KDP21310			
Pe125I23.19		+	2	2173	1308	Altered xyloglucan 4	435	gi|743785603|ref|XP_011025578.1|PREDICTED: protein ALTERED XYLOGLUCAN 4 [Populus euphratica]	XP_011025578	0.0	436	374
Pe125I23.20		+	2	1772	894	Acyl- N-acyltransferases [Arabidopsis thaliana]	297	gi|255581178|ref|XP_002531402.1|PREDICTED: uncharacterized protein LOC8281921 [Ricinus communis]gi|1000942633|ref|XP_015582222.1|PREDICTED: uncharacterized protein LOC8281921 [Ricinus communis]gi|1000942635|ref|XP_015582223.1|PREDICTED: uncharacterized protein LOC8281921 [Ricinus communis]gi|1000942637|ref|XP_015582224.1|PREDICTED: uncharacterized protein LOC8281921 [Ricinus communis]gi|1000942639|ref|XP_015582225.1|PREDICTED: uncharacterized protein LOC8281921 [Ricinus communis]gi|223528995|gb|EEF30986.1|N-acetyltransferase, putative [Ricinus communis]	XP_002531402, XP_015582222, XP_015582223, XP_015582224, XP_015582225, EEF30986	3.80E-119	316	237
Pe125I23.21		-	8	2259	1002	Serine threonine- kinase PBS1-like [Nelumbo nucifera]	262	gi|802787957|ref|XP_012092052.1|PREDICTED: serine/threonine-protein kinase PBS1 [Jatropha curcas]gi|643704249|gb|KDP21313.1|hypothetical protein JCGZ_21784 [Jatropha curcas]	XP_012092052, KDP21313	3.25E-164	257	243
Pe134H15.1		-	1	616	312	---Na---	101	No Blast Hit				
Pe134H15.2		-	2	4158	1611	Serine threonine phosphatase 2A 59 kda regulatory subunit B gamma isoform-like [Nelumbo nucifera]	536	gi|255568687|ref|XP_002525315.1|PREDICTED: serine/threonine protein phosphatase 2A 59 kDa regulatory subunit B' gamma isoform [Ricinus communis]gi|223535374|gb|EEF37048.1|protein phosphatase 2a, regulatory subunit, putative [Ricinus communis]	XP_002525315, EEF37048	0.0	544	491
Pe134H15.3		-	5	3782	651	PHD finger family bromo-adjacent (BAH) domain-containing isoform 1 [Theobroma cacao]	216	gi|802658543|ref|XP_012080629.1|PREDICTED: BAH and coiled-coil domain-containing protein 1-like [Jatropha curcas]gi|643720413|gb|KDP30792.1|hypothetical protein JCGZ_13735 [Jatropha curcas]	XP_012080629, KDP30792	7.34E-150	216	211
Pe134H15.4		-	4	4553	1044	Homeobox SBH1	347	gi|255554765|ref|XP_002518420.1|PREDICTED: homeobox protein SBH1 [Ricinus communis]gi|223542265|gb|EEF43807.1|homeobox protein knotted-1, putative [Ricinus communis]	XP_002518420, EEF43807	2.60E-135	359	270
Pe134H15.5	1 isoform	+	2	3056	324	Ribosomal L30 [Populus trichocarpa]	107	gi|224068949|ref|XP_002302864.1|hypothetical protein POPTR_0002s21620g [Populus trichocarpa]gi|566159273|ref|XP_006386785.1|ribosomal protein L30 [Populus trichocarpa]gi|743923609|ref|XP_011005901.1|PREDICTED: uncharacterized protein LOC105112040 [Populus euphratica]gi|743923611|ref|XP_011005902.1|PREDICTED: uncharacterized protein LOC105112040 [Populus euphratica]gi|222844590|gb|EEE82137.1|hypothetical protein POPTR_0002s21620g [Populus trichocarpa]gi|550345532|gb|ERP64582.1|ribosomal protein L30 [Populus trichocarpa]	XP_002302864, XP_006386785, XP_011005901, XP_011005902, EEE82137, ERP64582	6.18E-55	106	98

Pe134H15.6		-	4	2312	897	Nitrate transporter [Morus notabilis]	298	gi|802658554|ref|XP_012080632.1|PREDICTED : protein NRT1/ PTR FAMILY 6.4 [Jatropha curcas]gi|643720418|gb|KDP30797.1|hypothetica l protein JCGZ_13740 [Jatropha curcas]	XP_012080632, KDP30797	1.11E-169	298	273
Pe134H15.7		-	5	4386	1578	Endo-1,4-beta-glucanase [Populus trichocarpa]	525	gi|802658558|ref|XP_012080633.1|PREDICTED : endoglucanase 11-like [Jatropha curcas]gi|643720421|gb|KDP30800.1|hypothetica l protein JCGZ_13743 [Jatropha curcas]	XP_012080633, KDP30800	0.0	528	474
Pe134H15.8	2 isoforms	+	10	7290	1197	Integral membrane family [Populus trichocarpa]	398	gi|566204468|ref|XP_002320480.2|integral membrane family protein [Populus trichocarpa]gi|550324280|gb|EEE98795.2|integra l membrane family protein [Populus trichocarpa]	XP_002320480, EEE98795	0.0	396	327
Pe134H15.9		+	6	4371	1524	PREDICTED: uncharacterized protein LOC8282559 [Ricinus communis]	507	gi|1000953354|ref|XP_015578580.1|PREDICTE D: uncharacterized protein LOC8282559 [Ricinus communis]gi|1000953358|ref|XP_015578581.1|P REDICTED: uncharacterized protein LOC8282559 [Ricinus communis]	XP_015578580, XP_015578581	0.0	515	398
Pe134H15.10		+	1	465	465	---Na---	154	No Blast Hit				
Pe134H15.11		-	1	1743	1743	Armadillo beta-catenin repeat family [Populus trichocarpa]	580	gi|590711688|ref|XP_007049178.1|ARM repeat superfamily protein [Theobroma cacao]gi|508701439|gb|EOX93335.1|ARM repeat superfamily protein [Theobroma cacao]	XP_007049178, EOX93335	0.0	559	500
Pe134H15.12	2 isoforms	+	11	6597	1899	LRR and PYD domains-containing 10	632	gi|743929384|ref|XP_011008925.1|PREDICTED : uncharacterized protein LOC105114162 [Populus euphratica]gi|743929386|ref|XP_011008926.1|P REDICTED: uncharacterized protein LOC105114162 [Populus euphratica]gi|743929388|ref|XP_011008928.1|P REDICTED: uncharacterized protein LOC105114162 [Populus euphratica]gi|743929390|ref|XP_011008929.1|P REDICTED: uncharacterized protein LOC105114162 [Populus euphratica]gi|743929392|ref|XP_011008930.1|P REDICTED: uncharacterized protein LOC105114162 [Populus euphratica]gi|743929394|ref|XP_011008931.1|P REDICTED: uncharacterized protein LOC105114162 [Populus euphratica]gi|743929396|ref|XP_011008932.1|P REDICTED: uncharacterized protein LOC105114162 [Populus euphratica]	XP_011008925, XP_011008926, XP_011008928, XP_011008929, XP_011008930, XP_011008931, XP_011008932	0.0	570	514
Pe134H15.13	1 isoform	+	1	1756	594	Zinc finger family [Populus trichocarpa]	197	gi|802658605|ref|XP_012080646.1|PREDICTED : LON peptidase N-terminal domain and RING finger protein 1-like [Jatropha curcas]gi|643720432|gb|KDP30811.1|hypothetica l protein JCGZ_13754 [Jatropha curcas]	XP_012080646, KDP30811	1.01E-57	200	143
Pe134H15.14		+	2	651	333	1-aminocyclopropane-1-carboxylate oxidase family [Populus trichocarpa]	110	gi|743907526|ref|XP_011047191.1|PREDICTED : 1-aminocyclopropane-1-carboxylate oxidase [Populus euphratica]	XP_011047191	2.79E-61	110	105
Pe134H15.15		+	1	597	300	1-aminocyclopropane-1-carboxylate partial	99	gi|802658609|ref|XP_012080647.1|PREDICTED : 1-aminocyclopropane-1-carboxylate oxidase [Jatropha curcas]gi|643720433|gb|KDP30812.1|hypothetica l protein JCGZ_13755 [Jatropha curcas]	XP_012080647, KDP30812	1.10E-52	99	94

Pe134H15.16		+	2	295	219	---Na---	72	No Blast Hit				
Pe134H15.17		+	1	396	396	Probable pectinesterase pectinesterase inhibitor 13	131	gi|720087678|ref|XP_010244226.1|PREDICTED : uncharacterized protein LOC104588104 [Nelumbo nucifera]	XP_010244226	4.24E-24	102	71
Pe134H15.18		+	1	519	519	PREDICTED: uncharacterized protein LOC107880735	172	gi|1027097397|ref|XP_016648321.1|PREDICTED: uncharacterized protein LOC107880735 [Prunus mume]	XP_016648321	4.35E-18	164	88
Pe134H15.19		+	1	470	447	---Na---	148	No Blast Hit				
Pe135J12.1	Incomplete 3'	-	9	8520	4433	Methyl- -binding domain-containing 9	1477	gi|1000969843|ref|XP_015573615.1|PREDICTED: methyl-CpG-binding domain-containing protein 9 [Ricinus communis]	XP_015573615	0.0	1508	1084
Pe135J12.2		+	5	3534	990	FAM179B-like isoform X2 [Nelumbo nucifera]	329	gi|802543021|ref|XP_012081820.1|PREDICTED : uncharacterized protein LOC105641815 [Jatropha curcas]gi|643739631|gb|KDP45369.1|hypothetical protein JCGZ_09618 [Jatropha curcas]	XP_012081820. KDP45369	5.53E-170	329	291
Pe135J12.3		-	1	288	288	---Na---	95	No Blast Hit				
Pe135J12.4		+	1	1069	603	UDP-glycosyltransferase 90A1-like	200	gi|255552620|ref|XP_002517353.1|PREDICTED : UDP-glycosyltransferase 90A1 [Ricinus communis]gi|223543364|gb|EEF44895.1|UDP-glucosyltransferase, putative [Ricinus communis]	XP_002517353, EEF44895	1.80E-75	194	148
Pe135J12.5		+	1	162	162	UDP-glycosyltransferase 90A1-like	53	gi|743907784|ref|XP_011047327.1|PREDICTED : UDP-glycosyltransferase 90A1 [Populus euphratica]	XP_011047327	3.54E-09	48	38
Pe135J12.6		-	6	3390	2142	Cyclic nucleotide-gated ion channel 18	713	gi|802543152|ref|XP_012081898.1|PREDICTED : putative cyclic nucleotide-gated ion channel 18 [Jatropha curcas]	XP_012081898	0.0	711	600
Pe135J12.7		+	8	5774	2331	Potassium transporter 8-like	776	gi|802543156|ref|XP_012081921.1|PREDICTED : potassium transporter 8 [Jatropha curcas]gi|643739642|gb|KDP45380.1|hypothetical protein JCGZ_09629 [Jatropha curcas]	XP_012081921, KDP45380	0.0	776	724
Pe135J12.8		+	7	3608	1572	PREDICTED: uncharacterized protein LOC105641890 isoform X1	523	gi|802543176|ref|XP_012081932.1|PREDICTED : uncharacterized protein LOC105641890 isoform X1 [Jatropha curcas]	XP_012081932	0.0	523	451
Pe135J12.9		+	1	545	492	NHL domain-containing [Theobroma cacao]	163	gi|255552644|ref|XP_002517365.1|PREDICTED : uncharacterized protein LOC8280547 [Ricinus communis]gi|223543376|gb|EEF44907.1|conserved hypothetical protein [Ricinus communis]	XP_002517365, EEF44907	1.74E-47	158	112
Pe135J12.10		-	1	3011	1167	RING U-box superfamily isoform 1 [Theobroma cacao]	399	gi|590645683|ref|XP_007031412.1|RING/U-box superfamily protein isoform 1 [Theobroma cacao]gi|590645686|ref|XP_007031413.1|RING/U-box superfamily protein isoform 1 [Theobroma cacao]gi|508710441|gb|EOY02338.1|RING/U-box superfamily protein isoform 1 [Theobroma cacao]gi|508710442|gb|EOY02339.1|RING/U-box superfamily protein isoform 1 [Theobroma cacao]	XP_007031412, XP_007031413, EOY02338, EOY02339	0.0	403	341
Pe135J12.11		-	2	2480	1860	Probable inactive receptor kinase At2g26730	619	gi|223543378|gb|EEF44909.1|serine-threonine protein kinase, plant-type, putative [Ricinus communis]	EEF44909	0.0	596	460
Pe135J12.12	1 isoform	-	6	3408	564	Ubiquitin-conjugating enzyme E2 5 isoform 1 [Theobroma cacao]	187	gi|802543221|ref|XP_012081963.1|PREDICTED : ubiquitin-conjugating enzyme E2-23 kDa-like [Jatropha curcas]gi|643739649|gb|KDP45387.1|hypothetica	XP_012081963, KDP45387	3.46E-113	187	177

								l protein JCGZ_09636 [Jatropha curcas]				
Pe135J12.13		+	15	9543	3579	DNA repair UVH3 isoform X2	1192	gi|223543381|gb|EEF44912.1|DNA-repair protein UVH3, putative [Ricinus communis]	EEF44912	0.0	1267	874
Pe135J12.14	2 isoforms	+	7	2209	1074	Nodulin 21 -like transporter family isoform 1 [Theobroma cacao]	357	gi|590645647|ref|XP_007031402.1|Nodulin MtN21 /EamA-like transporter family protein, putative isoform 1 [Theobroma cacao]gi|508710431|gb|EOY02328.1|Nodulin MtN21 /EamA-like transporter family protein, putative isoform 1 [Theobroma cacao]	XP_007031402, EOY02328	1.93E-124	345	257
Pe135J12.15		+	3	589	402	Nodulin 21 -like transporter family [Arabidopsis thaliana]	133	gi|565443962|ref|XP_006283966.1|hypothetical protein CARUB_v10005089mg [Capsella rubella]gi|482552671|gb|EOA16864.1|hypothetical protein CARUB_v10005089mg [Capsella rubella]	XP_006283966, EOA16864	4.62E-36	124	94
Pe135J12.16		+	3	621	435	WAT1-related At5g40240-like	144	gi|1029012591|ref|XP_016670187.1|PREDICTED: WAT1-related protein At5g40240-like [Gossypium hirsutum]	XP_016670187	1.89E-43	146	109
Pe135J12.17		+	7	1940	1077	Nodulin 21 -like transporter family [Arabidopsis thaliana]	358	gi|590645647|ref|XP_007031402.1|Nodulin MtN21 /EamA-like transporter family protein, putative isoform 1 [Theobroma cacao]gi|508710431|gb|EOY02328.1|Nodulin MtN21 /EamA-like transporter family protein, putative isoform 1 [Theobroma cacao]	XP_007031402, EOY02328	1.56E-133	358	268
Pe135J12.18		+	6	1830	1089	WAT1-related At5g40240-like [Camelina sativa]	362	gi|674965205|emb|CDX67448.1|BnaA07g14880D [Brassica napus]	CDX67448	2.62E-68	337	206
Pe135J12.19		+	4	1007	621	WAT1-related At5g40240-like [Camelina sativa]	206	gi|694434655|ref|XP_009344538.1|PREDICTED: WAT1-related protein At5g40230-like [Pyrus x bretschneideri]gi|694434703|ref|XP_009344561.1|PREDICTED: WAT1-related protein At5g40230-like [Pyrus x bretschneideri]	XP_009344538, XP_009344561	1.42E-72	206	153
Pe135J12.20	1 isoform	+	7	2786	1074	WAT1-related At3g28050-like	357	gi|255552664|ref|XP_002517375.1|PREDICTED: WAT1-related protein At3g28050 [Ricinus communis]gi|223543386|gb|EEF44917.1|Auxin-induced protein 5NG4, putative [Ricinus communis]	XP_002517375, EEF44917	0.0	359	315
Pe135J12.21		+	1	2036	552	Calmodulin binding [Ricinus communis]	183	gi|1000969899|ref|XP_015573627.1|PREDICTED: uncharacterized protein LOC8280560 [Ricinus communis]gi|223543389|gb|EEF44920.1|calmodulin binding protein, putative [Ricinus communis]	XP_015573627, EEF44920	4.32E-108	183	172
Pe135J12.22		-	3	2639	1242	ALTERED XYLOGLUCAN 4-like	413	gi|255552672|ref|XP_002517379.1|PREDICTED: protein ALTERED XYLOGLUCAN 4-like [Ricinus communis]gi|223543390|gb|EEF44921.1|conserved hypothetical protein [Ricinus communis]	XP_002517379, EEF44921	0.0	404	339
Pe135J12.23	Incomplete 3'	+	3	1448	1233	Xyloglucan glycosyltransferase 4	411	gi|255552680|ref|XP_002517383.1|PREDICTED: xyloglucan glycosyltransferase 4 [Ricinus communis]gi|223543394|gb|EEF44925.1|transferase, transferring glycosyl groups, putative [Ricinus communis]	XP_002517383, EEF44925	0.0	411	379
Pe138G17.1	Incomplete 3'	-	1	178	178	---Na---	59	No Blast Hit				
Pe138G17.2	2 isoforms	-	9	5161	870	Probable ribose-5-phosphate isomerase chloroplastic isoform X1	290	gi|1000945452|ref|XP_015581287.1|PREDICTED: probable ribose-5-phosphate isomerase 4, chloroplastic [Ricinus communis]	XP_015581287	1.96E-138	291	246
Pe138G17.3		-	1	294	294	---Na---	97	No Blast Hit				

Pe138G17.4		+	1	354	354	---Na---	117	No Blast Hit				
Pe138G17.5		-	2	1098	531	---Na---	176	No Blast Hit				
Pe138G17.6		-	8	4284	2934	Ribonuclease H At1g65750 family	977	gi|1012354778|gb|KYP65965.1|Putative ribonuclease H protein At1g65750 family [Cajanus cajan]	KYP65965	9.88E-133	627	352
Pe138G17.7		-	1	1242	1242	Hypothetical protein CISIN_1g046520mg	413	gi|641817638|gb|KDO38897.1|hypothetical protein CISIN_1g046520mg [Citrus sinensis]	KDO38897	9.98E-43	233	134
Pe138G17.8		-	1	353	207	---Na---	68	No Blast Hit				
Pe138G17.9	1 isoform	+	10	6113	2532	Subtilisin-like protease	843	gi|802620382|ref|XP_012075543.1|PREDICTED : subtilisin-like protease SBT2.5 [Jatropha curcas]gi|643726069|gb|KDP34877.1|hypothetica l protein JCGZ_09165 [Jatropha curcas]	XP_012075543, KDP34877	0.0	844	746
Pe138G17.10		-	1	671	279	---Na---	92	No Blast Hit				
Pe138G17.11		-	1	201	201	Gag protease poly [Theobroma cacao]	66	gi|658064690|ref|XP_008368288.1|PREDICTED : uncharacterized protein LOC103431892 [Malus domestica]	XP_008368288	2.93E-12	59	46
Pe138G17.12		+	1	195	195	---Na---	64	No Blast Hit				
Pe138G17.13		-	2	698	486	---Na---	161	No Blast Hit				
Pe138G17.14		-	1	240	240	---Na---	79	No Blast Hit				
Pe138G17.15		+	1	438	438	---Na---	145	No Blast Hit				
Pe138G17.16		-	2	809	720	Gag protease poly [Theobroma cacao]	239	gi|590689992|ref|XP_007043384.1|Gag protease polyprotein [Theobroma cacao]gi|508707319|gb|EOX99215.1|Gag protease polyprotein [Theobroma cacao]	XP_007043384, EOX99215	1.50E-09	90	53
Pe141B12.1	Incomplete 3'	-	12	6666	1641	Cleavage and polyadenylation specificity factor subunit 1	547	gi|566196509|ref|XP_002318462.2|cleavage and polyadenylation specificity factor family protein [Populus trichocarpa]gi|550326263|gb|EEE96682.2|cleava ge and polyadenylation specificity factor family protein [Populus trichocarpa]	XP_002318462, EEE96682	0.0	556	484
Pe141B12.2		+	2	4111	3417	Receptor kinase	1138	gi|802536920|ref|XP_012086888.1|PREDICTED : leucine-rich repeat receptor-like protein kinase PEPR1 [Jatropha curcas]	XP_012086888	0.0	1126	961
Pe141B12.3		+	3	3087	1593	Gag protease poly [Theobroma cacao]	530	gi|590728434|ref|XP_007099662.1|Gag protease polyprotein-like protein [Theobroma cacao]gi|508728474|gb|EOY20371.1|Gag protease polyprotein-like protein [Theobroma cacao]	XP_007099662, EOY20371	2.68E-64	415	209
Pe141B12.4		-	2	1243	420	---Na---	139	No Blast Hit				
Pe141B12.5		+	1	288	288	---Na---	95	No Blast Hit				
Pe141B12.6		-	1	525	525	---Na---	174	No Blast Hit				
Pe141B12.7		-	15	6769	2454	PREDICTED: uncharacterized protein LOC105139805 isoform X2	817	gi|743902739|ref|XP_011044715.1|PREDICTED : uncharacterized protein LOC105139805 isoform X2 [Populus euphratica]	XP_011044715	0.0	827	671
Pe141B12.8		-	1	1069	399	Auxin-responsive SAUR32	132	gi|590674460|ref|XP_007039174.1|SAUR family protein [Theobroma cacao]gi|508776419|gb|EOY23675.1|SAUR family protein [Theobroma cacao]	XP_007039174, EOY23675	1.44E-49	129	112
Pe141B12.9		-	1	1199	951	Transmembrane [Medicago truncatula]	316	gi|224120976|ref|XP_002318466.1|hypothetical protein POPTR_0012s03060g [Populus	XP_002318466, EEE96686	1.66E-100	300	217

							trichocarpa]gi|222859139|gb|EEE96686.1|hypothetical protein POPTR_0012s03060g [Populus trichocarpa]				
Pe141B12.10	-	4	658	282	---Na---	93	No Blast Hit				
Pe141B12.11	+	3	925	593	Pyridoxal chloroplastic	156	gi|743902730|ref|XP_011044711.1|PREDICTED: LOW QUALITY PROTEIN: pyridoxal reductase, chloroplastic [Populus euphratica]	XP_011044711	6.92E-65	121	113
Pe141J23.1	+	2	682	579	Maternal effect embryoarrest 60	192	gi|802546376|ref|XP_012084606.1|PREDICTED: uncharacterized protein LOC105643970 isoform X1 [Jatropha curcas]gi|643739461|gb|KDP45215.1|hypothetical protein JCGZ_15080 [Jatropha curcas]	XP_012084606, KDP45215	4.57E-89	196	175
Pe141J23.2	-	3	1738	1578	Probable disease resistance At4g27220 [Populus euphratica]	525	gi|743898427|ref|XP_011042504.1|PREDICTED: probable disease resistance protein At4g27220 [Populus euphratica]gi|743898429|ref|XP_011042505.1|PREDICTED: probable disease resistance protein At4g27220 [Populus euphratica]gi|743898431|ref|XP_011042506.1|PREDICTED: probable disease resistance protein At4g27220 [Populus euphratica]	XP_011042504, XP_011042505, XP_011042506	7.52E-86	596	296
Pe141J23.3	+	2	580	399	---Na---	132	No Blast Hit				
Pe141J23.4	+	1	225	225	---Na---	74	No Blast Hit				
Pe141J23.5	-	1	1464	1464	Gag protease poly [Theobroma cacao]	487	gi|590633038|ref|XP_007028010.1|Gag protease polyprotein [Theobroma cacao]gi|508716615|gb|EOY08512.1|Gag protease polyprotein [Theobroma cacao]	XP_007028010, EOY08512	2.42E-44	307	158
Pe141J23.6	-	1	189	189	---Na---	62	No Blast Hit				
Pe141J23.7	+	1	417	417	---Na---	138	No Blast Hit				
Pe141J23.8	-	4	3814	1158	Calmodulin 3	385	gi|643739481|gb|KDP45235.1|hypothetical protein JCGZ_15100 [Jatropha curcas]	KDP45235	1.06E-84	150	145
Pe141J23.9	-	2	1316	531	FAR1-RELATED SEQUENCE 5-like isoform X2 [Pyrus x bretschneideri]	176	gi|802546406|ref|XP_012084725.1|PREDICTED: protein FAR1-RELATED SEQUENCE 5 [Jatropha curcas]gi|802546408|ref|XP_012084733.1|PREDICTED: protein FAR1-RELATED SEQUENCE 5 [Jatropha curcas]gi|802546410|ref|XP_012084741.1|PREDICTED: protein FAR1-RELATED SEQUENCE 5 [Jatropha curcas]gi|643739478|gb|KDP45232.1|hypothetical protein JCGZ_15097 [Jatropha curcas]	XP_012084725, XP_012084733, XP_012084741, KDP45232	2.06E-107	176	163
Pe141J23.10	+	4	2487	888	Hypothetical protein DCAR_017700	295	gi|1021036674|gb|KZM94457.1|hypothetical protein DCAR_017700 [Daucus carota subsp. sativus]	KZM94457	1.60E-22	204	103
Pe141J23.11	-	2	346	264	Calmodulin 11	87	gi|743774547|ref|XP_010917630.1|PREDICTED: calmodulin-like protein 11 [Elaeis guineensis]	XP_010917630	3.11E-23	66	59
Pe141J23.12	-	6	3110	432	Nuclease HARBI1	143	gi|702274813|ref|XP_010044172.1|PREDICTED: putative nuclease HARBI1 isoform X2 [Eucalyptus grandis]	XP_010044172	5.31E-10	103	55
Pe141J23.13	-	1	1870	477	Adrenodoxin mitochondrial	158	gi|719963350|ref|XP_010249447.1|PREDICTED: adrenodoxin-like protein, mitochondrial [Nelumbo nucifera]	XP_010249447	3.37E-82	163	142
Pe141J23.14	+	9	4303	2550	Linoleate 9S-lipoxygenase 6-like	849	gi|802546423|ref|XP_012084809.1|PREDICTED: linoleate 9S-lipoxygenase 6-like [Jatropha	XP_012084809	0.0	857	704

								curcas]				
Pe141J23.15	1 isoform	-	9	9258	1455	Glycine oxidase isoform X1 [Ricinus communis]	489	gi|566201903|ref|XP_006374834.1|hypothetical protein POPTR_0014s01860g [Populus trichocarpa]gi|550323140|gb|ERP52631.1|hypothetical protein POPTR_0014s01860g [Populus trichocarpa]	XP_006374834, ERP52631	0.0	490	383
Pe141J23.16	1 isoform	+	2	3289	591	PREDICTED: uncharacterized protein LOC105114482	196	gi|743930181|ref|XP_011009336.1|PREDICTED: uncharacterized protein LOC105114482 [Populus euphratica]	XP_011009336	6.17E-87	165	146
Pe141J23.17		-	14	5226	1980	Actin binding Calponin (CH) domain-containing isoform 1 [Theobroma cacao]	659	gi|224065468|ref|XP_002301825.1|hypothetical protein POPTR_0002s25290g [Populus trichocarpa]gi|222843551|gb|EEE81098.1|hypothetical protein POPTR_0002s25290g [Populus trichocarpa]	XP_002301825, EEE81098	0.0	660	640
Pe141J23.18	2 isoforms	-	9	4428	1200	At5g48470 [Arabidopsis thaliana]	409	gi|802546445|ref|XP_012084899.1|PREDICTED: uncharacterized protein LOC105644228 isoform X1 [Jatropha curcas]gi|643739494|gb|KDP45248.1|hypothetical protein JCGZ_15113 [Jatropha curcas]	XP_012084899, KDP45248	0.0	401	348
Pe141J23.19		+	2	1241	495	Lactoylglutathione lyase glyoxalase I family [Theobroma cacao]	164	gi|1029102091|ref|XP_016710421.1|PREDICTED: uncharacterized protein At5g48480-like [Gossypium hirsutum]gi|728832363|gb|KHG11806.1|hypothetical protein F383_11017 [Gossypium arboreum]	XP_016710421, KHG11806	1.60E-70	164	136
Pe141J23.20		-	1	539	318	Lipid-transfer DIR1	105	gi|1026022387|ref|XP_016557586.1|PREDICTED: putative lipid-transfer protein DIR1 [Capsicum annuum]	XP_016557586	1.56E-33	100	79
Pe141J23.21	1 isoform	+	4	2432	444	ATP-dependent rna helicase dbp7	147	gi|1000950046|ref|XP_015579709.1|PREDICTED: uncharacterized protein LOC8259691 [Ricinus communis]	XP_015579709	2.40E-80	148	132
Pe141J23.22		-	15	7531	1527	Gb: isoform 1 [Theobroma cacao]	508	gi|802546458|ref|XP_012084969.1|PREDICTED: HAUS augmin-like complex subunit 3 [Jatropha curcas]gi|643739499|gb|KDP45253.1|hypothetical protein JCGZ_15118 [Jatropha curcas]	XP_012084969, KDP45253	0.0	508	476
Pe141K8.1		-	2	2017	1533	Cytochrome P450 71A1-like	510	gi|224070800|ref|XP_002303241.1|hypothetical protein POPTR_0003s06460g [Populus trichocarpa]gi|222840673|gb|EEE78220.1|hypothetical protein POPTR_0003s06460g [Populus trichocarpa]	XP_002303241, EEE78220	0.0	512	476
Pe141K8.2		-	2	2315	1536	Cytochrome P450 71A1-like	511	gi|566149458|ref|XP_006369134.1|hypothetical protein POPTR_0001s16790g [Populus trichocarpa]gi|550347495|gb|ERP65703.1|hypothetical protein POPTR_0001s16790g [Populus trichocarpa]	XP_006369134, ERP65703	0.0	505	452
Pe141K8.3		+	1	1053	411	PREDICTED: uncharacterized protein LOC105631382 [Jatropha curcas]	184	gi|802576719|ref|XP_012068870.1|PREDICTED: uncharacterized protein LOC105631382 [Jatropha curcas]gi|643733845|gb|KDP40688.1|hypothetical protein JCGZ_24687 [Jatropha curcas]	XP_012068870, KDP40688	1.13E-37	123	105
Pe141K8.4		+	8	5119	2769	Linoleate 13S-lipoxygenase 3-chloroplastic-like	922	gi|566149454|ref|XP_006369132.1|hypothetical protein POPTR_0001s16780g [Populus trichocarpa]gi|550347493|gb|ERP65701.1|hypothetical protein POPTR_0001s16780g [Populus trichocarpa]	XP_006369132, ERP65701	0.0	925	833
Pe141K8.5		-	1	2619	1950	Cation calcium exchanger 4-like	649	gi|566161201|ref|XP_002304130.2|hypothetical protein POPTR_0003s06520g [Populus	XP_002304130, EEE79109	0.0	648	564

Pe141K8.6	2 isoforms	+	14	5586	1527	Aldehyde dehydrogenase family 7 member a1 turgor responsive family [Populus trichocarpa]	508	trichocarpa]gi|550342555|gb|EEE79109.2|hypothetical protein POPTR_0003s06520g [Populus trichocarpa] gi|743881366|ref|XP_011036450.1|PREDICTED: aldehyde dehydrogenase family 7 member B4-like [Populus euphratica]gi|743881370|ref|XP_011036452.1|PREDICTED: aldehyde dehydrogenase family 7 member B4-like [Populus euphratica]gi|743881374|ref|XP_011036453.1|PREDICTED: aldehyde dehydrogenase family 7 member B4-like [Populus euphratica]	XP_011036450, XP_011036452, XP_011036453	0.0	508	492
Pe141K8.7	1 isoform	-	1	2512	486	Uncharacterized protein TCM_029159 [Theobroma cacao]	161	gi|590620896|ref|XP_007024658.1|Uncharacterized protein TCM_029159 [Theobroma cacao]gi|508780024|gb|EOY27280.1|Uncharacterized protein TCM_029159 [Theobroma cacao]	XP_007024658, EOY27280	1.01E-85	163	143
Pe141K8.8		-	7	4896	1968	Lipase class 3 family [Populus trichocarpa]	655	gi|743881386|ref|XP_011036456.1|PREDICTED: uncharacterized protein LOC105133971 [Populus euphratica]gi|743881388|ref|XP_011036457.1|PREDICTED: uncharacterized protein LOC105133971 [Populus euphratica]	XP_011036456, XP_011036457	0.0	645	551
Pe141K8.9		-	9	3577	741	Proteasome subunit alpha type-6	246	gi|1011993226|ref|XP_015940788.1|PREDICTED: proteasome subunit alpha type-6 [Arachis duranensis]gi|1021468759|ref|XP_016181042.1|PREDICTED: proteasome subunit alpha type-6 [Arachis ipaensis]	XP_015940788, XP_016181042	5.72E-172	246	244
Pe141K8.10		+	13	4468	2280	Inter-alpha-trypsin inhibitor heavy chain H3-like isoform X1	759	gi|743878811|ref|XP_011035743.1|PREDICTED: inter-alpha-trypsin inhibitor heavy chain H3-like isoform X1 [Populus euphratica]	XP_011035743	0.0	758	635
Pe141K8.11	2 isoforms	+	5	2786	537	Probable nucleoside diphosphate kinase 5 isoform X1 [Populus euphratica]	178	gi|743881409|ref|XP_011036462.1|PREDICTED: probable nucleoside diphosphate kinase 5 isoform X1 [Populus euphratica]gi|743881413|ref|XP_011036463.1|PREDICTED: probable nucleoside diphosphate kinase 5 isoform X1 [Populus euphratica]gi|743881415|ref|XP_011036464.1|PREDICTED: probable nucleoside diphosphate kinase 5 isoform X1 [Populus euphratica]gi|743881429|ref|XP_011036465.1|PREDICTED: probable nucleoside diphosphate kinase 5 isoform X1 [Populus euphratica]gi|743881433|ref|XP_011036466.1|PREDICTED: probable nucleoside diphosphate kinase 5 isoform X1 [Populus euphratica]gi|743881437|ref|XP_011036467.1|PREDICTED: probable nucleoside diphosphate kinase 5 isoform X1 [Populus euphratica]gi|743881439|ref|XP_011036468.1|PREDICTED: probable nucleoside diphosphate kinase 5 isoform X1 [Populus euphratica]gi|743881443|ref|XP_011036469.1|PREDICTED: probable nucleoside diphosphate kinase 5 isoform X1 [Populus euphratica]gi|743881447|ref|XP_011036470.1|PREDICTED: probable nucleoside diphosphate kinase 5 isoform X1 [Populus euphratica]	XP_011036462, XP_011036463, XP_011036464, XP_011036465, XP_011036466, XP_011036467, XP_011036468, XP_011036469, XP_011036470	1.83E-78	168	142

Pe141K8.12		+	5	3247	387	Small nuclear ribonucleo [Populus trichocarpa]	128	gi|255583960|ref|XP_002532727.1|PREDICTED: sm-like protein LSM1B [Ricinus communis]gi|223527535|gb|EEF29658.1|lsm1, putative [Ricinus communis]	XP_002532727, EEF29658	2.81E-80	128	126
Pe141K8.13		-	1	1881	1881	Exocyst complex component EXO70A1-like	626	gi|743935308|ref|XP_011012017.1|PREDICTED: exocyst complex component EXO70A1-like [Populus euphratica]	XP_011012017	0.0	636	578
Pe141K8.14		+	12	4093	1269	Nucleolysin TIAR	422	gi|595933803|ref|XP_007215433.1|hypothetical protein PRUPE_ppa006170mg [Prunus persica]gi|645243848|ref|XP_008228163.1|PREDICTED: oligouridylate-binding protein 1B [Prunus mume]gi|462411583|gb|EMJ16632.1|hypothetical protein PRUPE_ppa006170mg [Prunus persica]	XP_007215433, XP_008228163, EMJ16632	0.0	401	383
Pe141K8.15		+	24	11592	3213	Nuclear pore complex NUP107	1070	gi|802576348|ref|XP_012068848.1|PREDICTED: nuclear pore complex protein NUP107 [Jatropha curcas]gi|643733827|gb|KDP40670.1|hypothetical protein JCGZ_24669 [Jatropha curcas]	XP_012068848, KDP40670	0.0	1087	967
Pe141K8.16		+	12	7209	3975	Mediator of RNA polymerase II transcription subunit 15a isoform X2	1324	gi|802576346|ref|XP_012068847.1|PREDICTED: uncharacterized protein LOC105631363 [Jatropha curcas]gi|643733826|gb|KDP40669.1|hypothetical protein JCGZ_24668 [Jatropha curcas]	XP_012068847, KDP40669	0.0	1380	1069
Pe164A12.1	Incomplete 3'	-	1	1017	1017	tRNA rRNA methyltransferase family [Populus trichocarpa]	339	gi|743943514|ref|XP_011016266.1|PREDICTED: uncharacterized protein LOC105119782 isoform X1 [Populus euphratica]	XP_011016266	5.65E-88	379	226
Pe164A12.2	1 isoform	+	1	2497	693	Mitochondrial import inner membrane translocase subunit TIM17-2-like	230	gi|802627893|ref|XP_012076870.1|PREDICTED: mitochondrial import inner membrane translocase subunit TIM17-2-like [Jatropha curcas]gi|643724594|gb|KDP33795.1|hypothetical protein JCGZ_07366 [Jatropha curcas]	XP_012076870, KDP33795	1.42E-119	230	199
Pe164A12.3		-	9	5640	1497	Hexokinase-1-like	498	gi|802627906|ref|XP_012076877.1|PREDICTED: hexokinase-1-like [Jatropha curcas]gi|643724600|gb|KDP33801.1|hypothetical protein JCGZ_07372 [Jatropha curcas]	XP_012076877, KDP33801	0.0	498	471
Pe164A12.4		+	1	1593	1593	DETOXIFICATION 51-like	530	gi|255546327|ref|XP_002514223.1|PREDICTED: protein DETOXIFICATION 52 [Ricinus communis]gi|223546679|gb|EEF48177.1|multidrug resistance pump, putative [Ricinus communis]	XP_002514223, EEF48177	0.0	534	451
Pe164A12.5		-	1	384	384	---Na---	127	No Blast Hit				
Pe164A12.6		+	1	447	447	PREDICTED: uncharacterized protein LOC107820043	148	gi|1025310513|ref|XP_016501732.1|PREDICTED: uncharacterized protein LOC107820043 [Nicotiana tabacum]	XP_016501732	1.14E-24	143	86
Pe164A12.7		+	3	1919	1290	IQ-DOMAIN 14 [Vitis vinifera]	429	gi|223546680|gb|EEF48178.1|conserved hypothetical protein [Ricinus communis]	EEF48178	0.0	429	345
Pe164A12.8	1 isoform	-	5	2346	1155	Probable pectinesterase 53	384	gi|1000977251|ref|XP_002514210.2|PREDICTED: probable pectinesterase 53 [Ricinus communis]	XP_002514210	0.0	382	339
Pe164A12.9		-	11	7964	4503	Phosphoinositide 3-kinase regulatory subunit 4	1500	gi|802627834|ref|XP_012076849.1|PREDICTED: probable serine/threonine-protein kinase vps15 isoform X1 [Jatropha curcas]gi|643724584|gb|KDP33785.1|hypothetical protein JCGZ_07356 [Jatropha curcas]	XP_012076849, KDP33785	0.0	1548	1343
Pe164A12.10		-	1	1061	228	Programmed cell death 4-like	75	gi|313586565|gb|ADR71293.1|hypothetical protein 17 [Hevea brasiliensis]	ADR71293	2.89E-29	76	66
Pe164A12.11		+	5	1076	396	Arginine--tRNA	131	gi|590576591|ref|XP_007012999.1|Arginyl-	XP_007012999,	2.19E-35	127	97

						cytoplasmic-like isoform X2		tRNA synthetase, class Ic isoform 1 [Theobroma cacao]gi|508783362|gb|EOY30618.1|Arginyl-tRNA synthetase, class Ic isoform 1 [Theobroma cacao]	EOY30618			
Pe164A12.12		+	9	4753	900	Arginyl-tRNA class Ic isoform 4 [Theobroma cacao]	299	gi|1021029800|gb|KZM87585.1|hypothetical protein DCAR_031954 [Daucus carota subsp. sativus]	KZM87585	6.59E-147	312	250
Pe164A12.13		-	2	1455	582	Oxidative stress isoform 2 [Theobroma cacao]	193	gi|225445063|ref|XP_002280262.1|PREDICTED: uncharacterized protein LOC100249186 [Vitis vinifera]	XP_002280262	3.60E-44	192	130
Pe164A12.14	1 isoform	+	4	3414	1509	Glucan endo-1,3-beta-glucosidase 13	502	gi|802627817|ref|XP_012076842.1|PREDICTED: glucan endo-1,3-beta-glucosidase 13-like [Jatropha curcas]gi|643724578|gb|KDP33779.1|hypothetical protein JCGZ_07350 [Jatropha curcas]	XP_012076842, KDP33779	0.0	492	453
Pe164A12.15		-	3	2306	396	Profilin 1 isoform 1 [Theobroma cacao]	131	gi|728849826|gb|KHG29269.1|Profilin-1 [Gossypium arboreum]	KHG29269	1.28E-80	131	123
Pe164A12.16		-	3	730	399	Profilin 5 [Theobroma cacao]	132	gi|1012350201|gb|KYP61390.1|Profilin-6 [Cajanus cajan]	KYP61390	6.57E-76	133	125
Pe164A12.17		+	2	2467	1257	PREDICTED: uncharacterized protein LOC105637821 [Jatropha curcas]	418	gi|802627802|ref|XP_012076837.1|PREDICTED: uncharacterized protein LOC105637821 [Jatropha curcas]gi|643724574|gb|KDP33775.1|hypothetical protein JCGZ_07346 [Jatropha curcas]	XP_012076837, KDP33775	0.0	416	386
Pe164A12.18		+	3	2436	771	Acid phosphatase 1	256	gi|255546271|ref|XP_002514195.1|PREDICTED: acid phosphatase 1 [Ricinus communis]gi|223546651|gb|EEF48149.1|Acid phosphatase 1 precursor, putative [Ricinus communis]	XP_002514195, EEF48149	2.07E-114	252	205
Pe164A12.19		-	1	1272	1272	Leucine-rich repeat extensin 4	423	gi|802627797|ref|XP_012076834.1|PREDICTED: LOW QUALITY PROTEIN: leucine-rich repeat extensin-like protein 4 [Jatropha curcas]	XP_012076834	0.0	414	338
Pe164A12.20	5 isoforms	+	6	2194	855	Dual specificity phosphatase DSP8	284	gi|255546267|ref|XP_002514193.1|PREDICTED: putative dual specificity protein phosphatase DSP8 [Ricinus communis]gi|223546649|gb|EEF48147.1|Protein-tyrosine phosphatase mitochondrial 1, mitochondrial precursor, putative [Ricinus communis]	XP_002514193, EEF48147	1.76E-170	285	258
Pe164A12.21		-	3	2474	921	NAC domain containing 75 isoform 1 [Theobroma cacao]	306	gi|643724570|gb|KDP33771.1|hypothetical protein JCGZ_07342 [Jatropha curcas]	KDP33771	3.00E-138	306	254
Pe164B18.1	Incomplete 5'	+	8	1982	918	OPEN STOMATA 1 family [Populus trichocarpa]	305	gi|545715729|gb|AGW51607.1|serine/threonine protein kinase [Populus tremula]gi|545715731|gb|AGW51608.1|serine/threonine protein kinase [Populus tremula]	AGW51607, AGW51608	0.0	305	295
Pe164B18.2		+	1	564	564	---Na---	187	No Blast Hit				
Pe164B18.3		-	6	1550	720	Far upstream element-binding 1-like	239	gi|255561653|ref|XP_002521836.1|PREDICTED: far upstream element-binding protein 2 [Ricinus communis]gi|223538874|gb|EEF40472.1|RNA-binding protein Nova-1, putative [Ricinus communis]	XP_002521836, EEF40472	3.53E-37	246	148
Pe164B18.4		-	2	791	579	PREDICTED: uncharacterized protein LOC104901011	192	gi|731351096|ref|XP_010686855.1|PREDICTED: uncharacterized protein LOC104901011 [Beta vulgaris subsp. vulgaris]	XP_010686855	1.42E-28	144	83
Pe164B18.5		-	3	2532	1608	PREDICTED: uncharacterized protein	535	gi|731346747|ref|XP_010684619.1|PREDICTED: uncharacterized protein LOC104899180 [Beta	XP_010684619	2.26E-41	392	185

						LOC104899180		vulgaris subsp. vulgaris]				
Pe164B18.6		+	1	723	723	---Na---	240	No Blast Hit				
Pe164B18.7		-	6	1752	708	Far upstream element-binding 1-like	235	gi|255561653|ref|XP_002521836.1|PREDICTED: far upstream element-binding protein 2 [Ricinus communis]gi|223538874|gb|EEF40472.1|RNA-binding protein Nova-1, putative [Ricinus communis]	XP_002521836, EEF40472	2.58E-32	244	140
Pe164B18.8		-	13	3421	1212	Probable N-acetyl-gamma-glutamyl-phosphate chloroplastic	403	gi|255561649|ref|XP_002521834.1|PREDICTED: probable N-acetyl-gamma-glutamyl-phosphate reductase, chloroplastic [Ricinus communis]gi|223538872|gb|EEF40470.1|N-acetyl-gamma-glutamyl-phosphate reductase, putative [Ricinus communis]	XP_002521834, EEF40470	0.0	401	359
Pe164B18.9		-	3	1854	825	Salicylic acid-binding 2-like	274	gi|566187404|ref|XP_002313085.2|hypothetical protein POPTR_0009s11070g [Populus trichocarpa]gi|550331488|gb|EEE87040.2|hypothetical protein POPTR_0009s11070g [Populus trichocarpa]	XP_002313085, EEE87040	2.58E-107	264	201
Pe164B18.10		-	3	1903	876	Methyl esterase isoform 1 [Theobroma cacao]	291	gi|566187404|ref|XP_002313085.2|hypothetical protein POPTR_0009s11070g [Populus trichocarpa]gi|550331488|gb|EEE87040.2|hypothetical protein POPTR_0009s11070g [Populus trichocarpa]	XP_002313085, EEE87040	1.68E-108	285	213
Pe164B18.11		-	3	1853	888	Salicylic acid-binding 2-like	303	gi|566187404|ref|XP_002313085.2|hypothetical protein POPTR_0009s11070g [Populus trichocarpa]gi|550331488|gb|EEE87040.2|hypothetical protein POPTR_0009s11070g [Populus trichocarpa]	XP_002313085, EEE87040	3.90E-109	285	214
Pe164B18.12		-	3	1328	846	Salicylic acid-binding 2-like	281	gi|566187404|ref|XP_002313085.2|hypothetical protein POPTR_0009s11070g [Populus trichocarpa]gi|550331488|gb|EEE87040.2|hypothetical protein POPTR_0009s11070g [Populus trichocarpa]	XP_002313085, EEE87040	1.33E-110	256	198
Pe164B18.13	1 isoform	-	9	3851	1557	BAT2 domain-containing 1 [Theobroma cacao]	518	gi|255556237|ref|XP_002519153.1|PREDICTED: uncharacterized protein LOC8275563 [Ricinus communis]gi|223541816|gb|EEF43364.1|conserved hypothetical protein [Ricinus communis]	XP_002519153, EEF43364	0.0	518	435
Pe164B18.14		-	1	2362	1863	Pollen-specific leucine-rich repeat extensin 3	620	gi|743923186|ref|XP_011005677.1|PREDICTED: pollen-specific leucine-rich repeat extensin-like protein 3 [Populus euphratica]	XP_011005677	1.38E-167	490	326
Pe164B18.15	3 isoforms	+	4	2571	1152	B3 domain-containing transcription factor VRN1-like isoform X1	383	gi|743923174|ref|XP_011005670.1|PREDICTED: B3 domain-containing transcription factor VRN1-like isoform X1 [Populus euphratica]	XP_011005670	0.0	381	324
Pe164B18.16	1 isoform	+	5	2866	1260	B3 domain-containing transcription factor VRN1-like [Populus euphratica]	419	gi|743879614|ref|XP_011035988.1|PREDICTED: B3 domain-containing transcription factor VRN1-like [Populus euphratica]gi|743879618|ref|XP_011035989.1|PREDICTED: B3 domain-containing transcription factor VRN1-like [Populus euphratica]	XP_011035988, XP_011035989	3.68E-58	358	195
Pe164B18.17		+	2	2071	1593	Cytochrome P450 CYP82D47-like [Citrus sinensis]	530	gi|743923158|ref|XP_011005663.1|PREDICTED: cytochrome P450 CYP82D47-like [Populus euphratica]	XP_011005663	0.0	526	424
Pe164B18.18		+	2	466	375	Target of rapamycin [Theobroma cacao]	124	gi|743923148|ref|XP_011005657.1|PREDICTED: uncharacterized protein LOC105111884 isoform X2 [Populus euphratica]	XP_011005657	1.30E-29	89	75
Pe164B18.19		+	2	1120	1005	Cytochrome P450 82C4-like	334	gi|566166707|ref|XP_006384482.1|hypothetical protein POPTR_0004s15470g [Populus	XP_006384482, ERP62279	5.34E-167	334	276

								trichocarpa]gi|550341100|gb|ERP62279.1|hypothetical protein POPTR_0004s15470g [Populus trichocarpa]					
Pe164B18.20		+	2	1829	1608	Cytochrome P450 CYP82D47-like [Citrus sinensis]	535	gi|743923158|ref|XP_011005663.1|PREDICTED: cytochrome P450 CYP82D47-like [Populus euphratica]	XP_011005663	0.0	531	426
Pe164B18.21		+	15	16279	11409	Serine threonine- kinase SMG1-like	3802	gi|743923146|ref|XP_011005656.1|PREDICTED: uncharacterized protein LOC105111884 isoform X1 [Populus euphratica]	XP_011005656	0.0	3761	3183
Pe164B18.22		+	1	243	243	---Na---	80	No Blast Hit				
Pe164B18.23		-	1	1881	1344	Myosin heavy chain kinase C-like	447	gi|566187442|ref|XP_002313076.2|hypothetical protein POPTR_0009s11270g [Populus trichocarpa]gi|550331505|gb|EEE87031.2|hypothetical protein POPTR_0009s11270g [Populus trichocarpa]	XP_002313076, EEE87031	0.0	432	367
Pe164B18.24		+	1	1922	1458	Allene oxide synthase-like	485	gi|743885582|ref|XP_011037601.1|PREDICTED: allene oxide synthase-like [Populus euphratica]	XP_011037601	0.0	470	397
Pe164B18.25		-	19	4819	1977	Long chain acyl-synthetase 2	658	gi|743794195|ref|XP_010999571.1|PREDICTED: long chain acyl-CoA synthetase 2 isoform X2 [Populus euphratica]	XP_010999571	0.0	658	602
Pe164B18.26		+	1	2558	1353	RING-H2 finger ATL65	494	gi|743794182|ref|XP_010999537.1|PREDICTED: RING-H2 finger protein ATL65 [Populus euphratica]	XP_010999537	1.38E-167	451	349
Pe164B18.27		+	3	738	462	Casp os03g0767900	153	gi|566166742|ref|XP_002305408.2|hypothetical protein POPTR_0004s15650g [Populus trichocarpa]gi|550341116|gb|EEE85919.2|hypothetical protein POPTR_0004s15650g [Populus trichocarpa]	XP_002305408, EEE85919	9.72E-87	151	144
Pe164B18.28		+	1	720	720	DC1 domain-containing family [Populus trichocarpa]	239	gi|224077790|ref|XP_002305409.1|DC1 domain-containing family protein [Populus trichocarpa]gi|222848373|gb|EEE85920.1|DC1 domain-containing family protein [Populus trichocarpa]	XP_002305409, EEE85920	1.35E-137	237	211
Pe164B18.29		-	1	555	555	Plastid division PDV2	184	gi|1000966554|ref|XP_015574550.1|PREDICTED: plastid division protein PDV2 [Ricinus communis]	XP_015574550	1.27E-72	186	146
Pe164D9.1	Incomplete 3'	-	1	1075	1075	Pentatricopeptide repeat-containing At3g02330	358	gi|566166021|ref|XP_006384245.1|hypothetical protein POPTR_0004s11010g [Populus trichocarpa]gi|550340791|gb|ERP62042.1|hypothetical protein POPTR_0004s11010g [Populus trichocarpa]	XP_006384245, ERP62042	1.75E-160	357	279
Pe164D9.2		+	1	519	447	Conserved hypothetical protein	148	gi|223526655|gb|EEF28897.1|conserved hypothetical protein [Ricinus communis]	EEF28897	1.11E-30	89	74
Pe164D9.3		+	1	1199	582	Basic leucine zipper 43-like	193	gi|802751716|ref|XP_012088083.1|PREDICTED: basic leucine zipper 43-like [Jatropha curcas]gi|643709985|gb|KDP24311.1|hypothetical protein JCGZ_25607 [Jatropha curcas]	XP_012088083, KDP24311	6.52E-94	195	167
Pe164D9.4		+	3	2603	1680	Probable galacturonosyltransferase 9	559	gi|802751706|ref|XP_012088081.1|PREDICTED: probable galacturonosyltransferase 9 [Jatropha curcas]gi|643709983|gb|KDP24309.1|hypothetical protein JCGZ_25605 [Jatropha curcas]	XP_012088081, KDP24309	0.0	545	501
Pe164D9.5	2 isoforms	+	1	3564	1467	6-phosphogluconate decarboxylating 3	488	gi|802751701|ref|XP_012088080.1|PREDICTED: 6-phosphogluconate dehydrogenase, decarboxylating 3 [Jatropha curcas]gi|643709982|gb|KDP24308.1|hypothetical protein JCGZ_25604 [Jatropha curcas]	XP_012088080, KDP24308	0.0	487	482
Pe164D9.6		+	1	1896	312	GLUTAMINE DUMPER	103	gi|658025697|ref|XP_008348261.1|PREDICTED	XP_008348261	9.15E-23	107	75

					6-like		: protein GLUTAMINE DUMPER 6-like [Malus domestica]				
Pe164D9.7	-	2	742	357	---Na---	118	No Blast Hit				
Pe164D9.8	-	1	658	342	GLUTAMINE DUMPER 3-like	113	gi|566213374|ref|XP_006373515.1|hypothetical protein POPTR_0017s14430g [Populus trichocarpa]gi|550320337|gb|ERP51312.1|hypothetical protein POPTR_0017s14430g [Populus trichocarpa]	XP_006373515, ERP51312	1.81E-33	105	84
Pe164D9.9	-	1	548	321	Transmembrane [Medicago truncatula]	106	gi|590651722|ref|XP_007032963.1|Glutamine dumper 2, putative [Theobroma cacao]gi|508711992|gb|EOY03889.1|Glutamine dumper 2, putative [Theobroma cacao]	XP_007032963, EOY03889	2.09E-31	106	80
Pe164D9.10	-	2	1567	1122	CONSTANS-like 1 [Gossypium hirsutum]	373	gi|41323976|gb|AAS00054.1|CONSTANS-like protein CO1 [Populus deltoides]	AAS00054	0.0	377	310
Pe164D9.11	+	3	1532	1296	F-box FBD LRR-repeat At1g13570-like [Malus domestica]	431	gi|802751866|ref|XP_012088117.1|PREDICTED: F-box/FBD/LRR-repeat protein At1g13570-like [Jatropha curcas]gi|802751871|ref|XP_012088118.1|PREDICTED: F-box/FBD/LRR-repeat protein At1g13570-like [Jatropha curcas]gi|802751876|ref|XP_012088119.1|PREDICTED: F-box/FBD/LRR-repeat protein At1g13570-like [Jatropha curcas]	XP_012088117, XP_012088118, XP_012088119	0.0	430	337
Pe164D9.12	-	11	2830	1872	NSP-interacting kinase 1	623	gi|566165977|ref|XP_006384231.1|hypothetical protein POPTR_0004s10790g [Populus trichocarpa]gi|550340770|gb|ERP62028.1|hypothetical protein POPTR_0004s10790g [Populus trichocarpa]	XP_006384231, ERP62028	0.0	623	578
Pe164D9.13	-	1	3276	1968	Pentatricopeptide repeat-containing mitochondrial-like	655	gi|802751886|ref|XP_012088121.1|PREDICTED: pentatricopeptide repeat-containing protein At3g02490, mitochondrial [Jatropha curcas]gi|643710015|gb|KDP24341.1|hypothetical protein JCGZ_25637 [Jatropha curcas]	XP_012088121, KDP24341	0.0	646	514
Pe164D9.14	-	3	435	204	Stress-induced KIN2-like	67	gi|659119050|ref|XP_008459448.1|PREDICTED: stress-induced protein KIN2-like [Cucumis melo]	XP_008459448	8.84E-24	67	64
Pe164D9.15	+	8	3627	939	Eukaryotic translation initiation factor 5B-like isoform X1	312	gi|566213340|ref|XP_006373498.1|hypothetical protein POPTR_0017s14290g [Populus trichocarpa]gi|550320320|gb|ERP51295.1|hypothetical protein POPTR_0017s14290g [Populus trichocarpa]	XP_006373498, ERP51295	7.19E-82	246	184
Pe164D9.16	-	1	1086	1086	S-adenosylmethionine decarboxylase family [Populus trichocarpa]	361	gi|743820479|ref|XP_011021158.1|PREDICTED: S-adenosylmethionine decarboxylase proenzyme-like [Populus euphratica]	XP_011021158	0.0	350	302
Pe164D9.17	-	2	294	156	S-adenosylmethionine decarboxylase proenzyme-like	51	gi|148807144|gb|ABR13282.1|putative S-adenosylmethionine decarboxylase [Prunus dulcis]	ABR13282	2.29E-23	51	51
Pe164D9.18	-	8	5817	1062	EVI5 [Jatropha curcas]	353	gi|1000952279|ref|XP_015578952.1|PREDICTED: EVI5-like protein [Ricinus communis]gi|1000952281|ref|XP_015578953.1|PREDICTED: EVI5-like protein [Ricinus communis]gi|1000952283|ref|XP_015578954.1|PREDICTED: EVI5-like protein [Ricinus communis]	XP_015578952, XP_015578953, XP_015578954	0.0	353	337
Pe164D9.19	+	4	4159	1986	ATP-dependent zinc metalloprotease -like [Pyrus x bretschneideri]	661	gi|743906670|ref|XP_011046761.1|PREDICTED: ATP-dependent zinc metalloprotease FtsH isoform X1 [Populus	XP_011046761, XP_011046762	0.0	662	554

							euphratica]gi|743906672|ref|XP_011046762.1|PREDICTED: ATP-dependent zinc metalloprotease FtsH isoform X1 [Populus euphratica]				
Pe164D9.20		-	4	2422	1458	Proline dehydrogenase mitochondrial-like	487	gi|566213328|ref|XP_006373492.1|hypothetical protein POPTR_0017s14250g [Populus trichocarpa]gi|550320314|gb|ERP51289.1|hypothetical protein POPTR_0017s14250g [Populus trichocarpa]	XP_006373492, ERP51289	0.0	500	383
Pe164D9.21		+	2	1285	1173	[Theobroma cacao]	390	gi|224079914|ref|XP_002305971.1|hypothetical protein POPTR_0004s10570g [Populus trichocarpa]gi|118484298|gb|ABK94028.1|unknown [Populus trichocarpa]gi|222848935|gb|EEE86482.1|hypothetical protein POPTR_0004s10570g [Populus trichocarpa]	XP_002305971, ABK94028, EEE86482	0.0	392	323
Pe164D9.22		+	6	2249	1494	Tryptophan synthase beta chain 2	497	gi|566213322|ref|XP_006373489.1|hypothetical protein POPTR_0017s14220g [Populus trichocarpa]gi|550320311|gb|ERP51286.1|hypothetical protein POPTR_0017s14220g [Populus trichocarpa]	XP_006373489, ERP51286	0.0	487	448
Pe164D9.23		-	1	198	198	Hypothetical protein POPTR_0017s14200g [Populus trichocarpa]	65	gi|566213314|ref|XP_006373485.1|hypothetical protein POPTR_0017s14200g [Populus trichocarpa]gi|566213316|ref|XP_006373486.1|hypothetical protein POPTR_0017s14200g [Populus trichocarpa]gi|550320307|gb|ERP51282.1|hypothetical protein POPTR_0017s14200g [Populus trichocarpa]gi|550320308|gb|ERP51283.1|hypothetical protein POPTR_0017s14200g [Populus trichocarpa]	XP_006373485, XP_006373486, ERP51282, ERP51283	6.94E-27	65	62
Pe164D9.24		+	1	1422	1422	Ribonuclease H At1g65750 family	473	gi|590608283|ref|XP_007021221.1|Uncharacterized protein TCM_031281 [Theobroma cacao]gi|508720849|gb|EOY12746.1|Uncharacterized protein TCM_031281 [Theobroma cacao]	XP_007021221, EOY12746	1.18E-74	445	231
Pe164D9.25		+	2	1949	1341	Trichome birefringence-like 19	446	gi|566165934|ref|XP_002305969.2|hypothetical protein POPTR_0004s10540g [Populus trichocarpa]gi|550340752|gb|EEE86480.2|hypothetical protein POPTR_0004s10540g [Populus trichocarpa]	XP_002305969, EEE86480	0.0	452	389
Pe164D9.26		+	2	1459	1278	Trichome birefringence-like 19	425	gi|566165931|ref|XP_002305967.2|hypothetical protein POPTR_0004s10520g [Populus trichocarpa]gi|550340751|gb|EEE86478.2|hypothetical protein POPTR_0004s10520g [Populus trichocarpa]	XP_002305967, EEE86478	0.0	425	352
Pe164D9.27		+	1	2889	2202	Endo-1,3(4)-beta-glucanase 2	733	gi|743906701|ref|XP_011046776.1|PREDICTED: putative endo-1,3(4)-beta-glucanase 2 [Populus euphratica]	XP_011046776	0.0	738	659
Pe164D9.28	Incomplete 3'	+	1	1022	1022	Proline-rich receptor kinase PERK8	340	gi|1000938780|ref|XP_015583515.1|PREDICTED: proline-rich receptor-like protein kinase PERK8 isoform X1 [Ricinus communis]	XP_015583515	1.67E-18	156	108
Pe164K17.1		+	4	1374	585	DNA repair RAD51 homolog 3 isoform X2	194	gi|802700078|ref|XP_012083596.1|PREDICTED: DNA repair protein RAD51 homolog 3 [Jatropha curcas]	XP_012083596	2.22E-106	194	177
Pe164K17.2		+	7	3497	2244	Transketolase [Theobroma cacao]	747	gi|802700075|ref|XP_012083595.1|PREDICTED: transketolase, chloroplastic [Jatropha curcas]gi|643717143|gb|KDP28769.1|hypothetical protein JCGZ_14540 [Jatropha curcas]	XP_012083595, KDP28769	0.0	753	697

Pe164K17.3		+	8	4575	1551	3-phosphoshikimate 1-carboxyvinyltransferase 2	516	gi|224063774|ref|XP_002301279.1|3-phosphoshikimate 1-carboxyvinyltransferase family protein [Populus trichocarpa]gi|222843005|gb|EEE80552.1|3-phosphoshikimate 1-carboxyvinyltransferase family protein [Populus trichocarpa]	XP_002301279, EEE80552	0.0	518	476
Pe164K17.4		+	1	1769	1329	UDP-glucuronate 4-epimerase 3	442	gi|802700069|ref|XP_012083592.1|PREDICTED: UDP-glucuronate 4-epimerase 3 [Jatropha curcas]	XP_012083592	0.0	445	415
Pe164K17.5		+	4	3232	2091	Leucine-rich repeat receptor kinase [Theobroma cacao]	696	gi|1001910103|gb|AMM43004.1|LRR-RLK [Vernicia montana]	AMM43004	0.0	646	556
Pe164K17.6		+	2	1057	663	Hypothetical protein POPTR_0002s14810g [Populus trichocarpa]	220	gi|224063798|ref|XP_002301283.1|hypothetical protein POPTR_0002s14810g [Populus trichocarpa]gi|222843009|gb|EEE80556.1|hypothetical protein POPTR_0002s14810g [Populus trichocarpa]	XP_002301283, EEE80556	3.07E-101	219	185
Pe164K17.7		-	7	3302	924	Probable S-acyltransferase 14	307	gi|743788719|ref|XP_011034295.1|PREDICTED: probable protein S-acyltransferase 14 [Populus euphratica]	XP_011034295	0.0	306	293
Pe164K17.8		+	7	2300	654	DUF1499 family [Medicago truncatula]	217	gi|255541316|ref|XP_002511722.1|PREDICTED: uncharacterized protein LOC8270642 [Ricinus communis]gi|223548902|gb|EEF50391.1|conserved hypothetical protein [Ricinus communis]	XP_002511722, EEF50391	5.87E-112	222	188
Pe164K17.9		-	7	1758	672	Proteasome subunit beta type-1-like	223	gi|697164738|ref|XP_009591182.1|PREDICTED: proteasome subunit beta type-1-like [Nicotiana tomentosiformis]gi|1025260797|ref|XP_016491078.1|PREDICTED: proteasome subunit beta type-1-like [Nicotiana tabacum]	XP_009591182, XP_016491078	1.81E-144	223	214
Pe164K17.10		+	4	1381	411	Cornichon homolog 4-like	136	gi|976902078|gb|KVH90036.1|Cornichon [Cynara cardunculus var. scolymus]	KVH90036	3.01E-37	136	96
Pe164K17.11	2 isoforms	-	3	1896	468	AT-hook motif nuclear-localized 7-like [Arachis ipaensis]	155	gi|224130006|ref|XP_002320727.1|hypothetical protein POPTR_0014s06550g [Populus trichocarpa]gi|222861500|gb|EEE99042.1|hypothetical protein POPTR_0014s06550g [Populus trichocarpa]	XP_002320727, EEE99042	1.06E-43	119	100
Pe164K17.12		+	1	1890	1890	PREDICTED: uncharacterized protein LOC105643110 [Jatropha curcas]	629	gi|802699728|ref|XP_012083563.1|PREDICTED: uncharacterized protein LOC105643110 [Jatropha curcas]gi|643717120|gb|KDP28746.1|hypothetical protein JCGZ_14517 [Jatropha curcas]	XP_012083563, KDP28746	0.0	638	555
Pe164K17.13		-	2	5789	576	LOB domain-containing 19-like	191	gi|802699725|ref|XP_012083562.1|PREDICTED: LOB domain-containing protein 19 [Jatropha curcas]	XP_012083562	8.71E-76	190	150
Pe164K17.14		+	2	1989	693	LOB domain-containing 18	230	gi|566158111|ref|XP_002301295.2|hypothetical protein POPTR_0002s15000g [Populus trichocarpa]gi|550345048|gb|EEE80568.2|hypothetical protein POPTR_0002s15000g [Populus trichocarpa]	XP_002301295, EEE80568	5.05E-103	214	186
Pe164K17.15		+	11	9502	5334	Brefeldin A-inhibited guanine nucleotide-exchange 2-like	1777	gi|802699495|ref|XP_012083558.1|PREDICTED: brefeldin A-inhibited guanine nucleotide-exchange protein 2-like [Jatropha curcas]gi|802699498|ref|XP_012083559.1|PREDICTED: brefeldin A-inhibited guanine nucleotide-exchange protein 2-like [Jatropha curcas]gi|643717116|gb|KDP28742.1|hypothetical protein JCGZ_14513 [Jatropha curcas]	XP_012083558, XP_012083559, KDP28742	0.0	1781	1681

Pe164K17.16	1 isoform	+	1	3023	915	DNA-binding family [Populus trichocarpa]	304	gi|743788812|ref|XP_011034536.1|PREDICTED: putative DNA-binding protein ESCAROLA [Populus euphratica]	XP_011034536	3.81E-117	307	235
Pe164K17.17	2 isoforms	+	3	2604	204	---Na---	67	No Blast Hit				
Pe164K17.18		-	2	436	336	Little zipper 2	111	gi|566158124|ref|XP_002302541.2|hypothetical protein POPTR_0002s15060g [Populus trichocarpa]gi|550345053|gb|EEE81814.2|hypothetical protein POPTR_0002s15060g [Populus trichocarpa]	XP_002302541, EEE81814	2.56E-25	106	77
Pe164K17.19		-	13	5922	2643	SMAD FHA domain-containing isoform 2 [Theobroma cacao]	880	gi|802699480|ref|XP_012083553.1|PREDICTED: hyaluronan-mediated motility receptor isoform X3 [Jatropha curcas]gi|643717111|gb|KDP28737.1|hypothetical protein JCGZ_14508 [Jatropha curcas]	XP_012083553, KDP28737	0.0	889	721
Pe164K17.20		-	7	3883	2025	Ankyrin repeat and zinc finger domain-containing 1	674	gi|743804997|ref|XP_011017509.1|PREDICTED: ankyrin repeat and zinc finger domain-containing protein 1 isoform X1 [Populus euphratica]gi|743805001|ref|XP_011017510.1|PREDICTED: ankyrin repeat and zinc finger domain-containing protein 1 isoform X1 [Populus euphratica]	XP_011017509, XP_011017510	0.0	667	514
Pe164K17.21		-	1	2505	1281	Fasciclin-like arabinogalactan 10	496	gi|743788844|ref|XP_011034618.1|PREDICTED: fasciclin-like arabinogalactan protein 10 [Populus euphratica]	XP_011034618	0.0	409	371
Pe164K17.22		+	4	2601	1266	Growth-regulating factor 9 isoform X1	465	gi|743788848|ref|XP_011034630.1|PREDICTED: growth-regulating factor 9 [Populus euphratica]gi|743788852|ref|XP_011034638.1|PREDICTED: growth-regulating factor 9 [Populus euphratica]	XP_011034630, XP_011034638	2.36E-100	429	250
Pe164K17.23		-	13	5476	1476	Spastin [Vitis vinifera]	491	gi|567905468|ref|XP_006445222.1|hypothetical protein CICLE_v10019901mg [Citrus clementina]gi|568875742|ref|XP_006490949.1|PREDICTED: spastin isoform X1 [Citrus sinensis]gi|557547484|gb|ESR58462.1|hypothetical protein CICLE_v10019901mg [Citrus clementina]gi|641867192|gb|KDO85876.1|hypothetical protein CISIN_1g011393mg [Citrus sinensis]	XP_006445222, XP_006490949, ESR58462, KDO85876	0.0	491	444
Pe164K17.24		+	5	2386	1533	Cytochrome P450 704C1-like	510	gi|743788878|ref|XP_011034716.1|PREDICTED: cytochrome P450 704C1 isoform X2 [Populus euphratica]	XP_011034716	0.0	510	441
Pe164K17.25		+	6	2959	1530	Cytochrome P450 704C1-like	509	gi|224127430|ref|XP_002320072.1|hypothetical protein POPTR_0014s06790g [Populus trichocarpa]gi|222860845|gb|EEE98387.1|hypothetical protein POPTR_0014s06790g [Populus trichocarpa]	XP_002320072, EEE98387	0.0	502	447
Pe164K17.26	2 isoforms / Incomplete 5'	-	3	1862	786	BEACH domain-containing C2-like	261	gi|823186269|ref|XP_012489801.1|PREDICTED: BEACH domain-containing protein lvsC [Gossypium raimondii]gi|763774021|gb|KJB41144.1|hypothetical protein B456_007G092900 [Gossypium raimondii]	XP_012489801, KJB41144	5.38E-95	239	196
Pe168B17.1	Incomplete 5'	+	3	603	365	---Na---	120	No Blast Hit				
Pe168B17.2		-	1	867	867	---Na---	288	No Blast Hit				
Pe168B17.3		+	3	1552	1014	BOI-related E3 ubiquitin-ligase 1-like isoform X1	337	gi|1000953298|ref|XP_015578652.1|PREDICTED: E3 ubiquitin-protein ligase BOI [Ricinus	XP_015578652, XP_015578653	3.57E-174	339	285

						[Populus euphratica]		communis]gi|1000953300|ref|XP_015578653.1|PREDICTED: E3 ubiquitin-protein ligase BOI [Ricinus communis]				
Pe168B17.4	1 isoform	-	8	2147	756	RNA-binding 48-like	251	gi|224104821|ref|XP_002313579.1|hypothetical protein POPTR_0009s16880g [Populus trichocarpa]gi|222849987|gb|EEE87534.1|hypothetical protein POPTR_0009s16880g [Populus trichocarpa]	XP_002313579, EEE87534	6.54E-112	239	196
Pe168B17.5	2 isoforms	+	4	1794	657	Pyroglutamyl-peptidase 1 [Nelumbo nucifera]	218	gi|645230232|ref|XP_008221840.1|PREDICTED: pyrrolidone-carboxylate peptidase [Prunus mume]	XP_008221840	9.73E-118	218	188
Pe168B17.6		+	9	2762	1413	Probable polygalacturonase At1g80170	483	gi|1000953188|ref|XP_015578604.1|PREDICTED: probable polygalacturonase At1g80170 [Ricinus communis]	XP_015578604	0.0	483	423
Pe168B17.7		+	7	3655	1500	Polygalacturonase At1g48100-like	499	gi|743938733|ref|XP_011013806.1|PREDICTED: polygalacturonase At1g48100-like [Populus euphratica]	XP_011013806	0.0	505	435
Pe168B17.8	2 isoforms	+	6	3005	546	ADP-ribosylation factor [Nelumbo nucifera]	181	gi|661882967|emb|CDP13175.1|unnamed protein product [Coffea canephora]	CDP13175	1.01E-126	181	181
Pe168B17.9	1 isoform	-	11	6457	1398	Regulator of G- signaling 1	465	gi|1000975769|ref|XP_015572282.1|PREDICTED: regulator of G-protein signaling 1 isoform X2 [Ricinus communis]	XP_015572282	0.0	461	407
Pe168B17.10		-	1	375	375	Omega-6 fatty acid endoplasmic reticulum isozyme 1-like	124	gi|45272283|gb|AAS57577.1|delta12-oleic acid desaturase [Euphorbia lagascae]	AAS57577	9.94E-26	97	71
Pe168B17.11		+	1	1104	1104	Omega-6 fatty acid desaturase [Rhus chinensis]	367	gi|661878762|emb|CDP17521.1|unnamed protein product [Coffea canephora]	CDP17521	0.0	360	304
Pe168B17.12	3 isoforms	+	7	6861	4140	COP1-interacting [Populus trichocarpa]	1379	gi|1000975573|ref|XP_015572220.1|PREDICTED: uncharacterized protein LOC8285446 isoform X1 [Ricinus communis]	XP_015572220	3.22E-56	782	397
Pe168B17.13		-	4	2419	1767	NRT1 PTR FAMILY -like	588	gi|802787577|ref|XP_012091963.1|PREDICTED: protein NRT1/ PTR FAMILY 2.13-like [Jatropha curcas]gi|643704177|gb|KDP21241.1|hypothetical protein JCGZ_21712 [Jatropha curcas]	XP_012091963, KDP21241	5.01E-142	582	348
Pe168B17.14		+	8	3306	1995	Proline-rich receptor kinase PERK13	664	gi|743872399|ref|XP_011034126.1|PREDICTED: proline-rich receptor-like protein kinase PERK13 [Populus euphratica]	XP_011034126	0.0	546	404
Pe168B17.15		-	7	2159	1497	Transcription factor bhlh90	498	gi|743872407|ref|XP_011034128.1|PREDICTED: transcription factor bHLH90 isoform X2 [Populus euphratica]	XP_011034128	5.80E-114	491	305
Pe168B17.16		+	4	2714	1704	Probable nucleoredoxin 1	567	gi|223535332|gb|EEF37007.1|nucleoredoxin, putative [Ricinus communis]	EEF37007	0.0	574	481
Pe168B17.17		+	4	2057	1701	Probable nucleoredoxin 1	566	gi|1000953211|ref|XP_002525368.2|PREDICTED: LOW QUALITY PROTEIN: uncharacterized protein LOC8282593 [Ricinus communis]	XP_002525368	0.0	525	432
Pe168B17.18		+	2	674	606	---Na---	201	No Blast Hit				
Pe168B17.19		+	3	1080	174	---Na---	57	No Blast Hit				
Pe168B17.20		+	2	321	249	---Na---	82	No Blast Hit				
Pe168B17.21		+	5	6792	2094	Gag protease poly [Theobroma cacao]	697	gi|590728434|ref|XP_007099662.1|Gag protease polyprotein-like protein [Theobroma cacao]gi|508728474|gb|EOY20371.1|Gag protease polyprotein-like protein [Theobroma cacao]	XP_007099662, EOY20371	1.10E-47	372	184

Pe171P13.1		+	2	1704	1083	Probable pectinesterase pectinesterase inhibitor 51	360	gi|743827206|ref|XP_011022961.1|PREDICTED: probable pectinesterase/pectinesterase inhibitor 51 [Populus euphratica]	XP_011022961	0.0	363	326
Pe171P13.2	1 isoform	-	4	4069	1908	UDP-glucuronate:xylan alpha-glucuronosyltransferase 1-like [Gossypium raimondii]	635	gi|60657592|gb|AAX33317.1|secondary cell wall-related glycosyltransferase family 8 [Populus tremula x Populus tremuloides]	AAX33317	0.0	630	579
Pe171P13.3		-	3	1190	780	Fidgetin 1	259	gi|255552382|ref|XP_002517235.1|PREDICTED: uncharacterized protein LOC8259126 [Ricinus communis]gi|223543606|gb|EEF45135.1|conserved hypothetical protein [Ricinus communis]	XP_002517235, EEF45135	7.65E-110	263	203
Pe171P13.4	2 isoforms	-	6	5015	1125	Probable Histone-lysine N-methyltransferase ATXR5	374	gi|567870359|ref|XP_006427801.1|hypothetical protein CICLE_v10025879mg [Citrus clementina]gi|568820204|ref|XP_006464618.1|PREDICTED: probable Histone-lysine N-methyltransferase ATXR5 [Citrus sinensis]gi|557529791|gb|ESR41041.1|hypothetical protein CICLE_v10025879mg [Citrus clementina]	XP_006427801, XP_006464618, ESR41041	0.0	374	321
Pe171P13.5	1 isoform	-	10	5935	1425	Bifunctional aspartate aminotransferase and glutamate aspartate-prephenate aminotransferase-like	474	gi|590707491|ref|XP_007048020.1|Aspartate aminotransferase isoform 1 [Theobroma cacao]gi|590707497|ref|XP_007048022.1|Aspartate aminotransferase isoform 1 [Theobroma cacao]gi|508700281|gb|EOX92177.1|Aspartate aminotransferase isoform 1 [Theobroma cacao]gi|508700283|gb|EOX92179.1|Aspartate aminotransferase isoform 1 [Theobroma cacao]	XP_007048020, XP_007048022, EOX92177, EOX92179	0.0	481	427
Pe171P13.6		+	1	663	663	MKS1 [Medicago truncatula]	220	gi|566169739|ref|XP_006382836.1|hypothetical protein POPTR_0005s05910g [Populus trichocarpa]gi|550338206|gb|ERP60633.1|hypothetical protein POPTR_0005s05910g [Populus trichocarpa]	XP_006382836, ERP60633	1.70E-67	195	149
Pe171P13.7		-	7	3333	957	Uridylate kinase	318	gi|255552374|ref|XP_002517231.1|PREDICTED: uridylate kinase isoform X2 [Ricinus communis]gi|223543602|gb|EEF45131.1|Uridylate kinase, putative [Ricinus communis]	XP_002517231, EEF45131	0.0	321	284
Pe171P13.8	1 isoform	+	2	5283	819	Tropinone reductase-like 1	272	gi|590632579|ref|XP_007027880.1|Short chain alcohol dehydrogenase [Theobroma cacao]gi|508716485|gb|EOY08382.1|Short chain alcohol dehydrogenase [Theobroma cacao]	XP_007027880, EOY08382	1.46E-118	257	208
Pe171P13.9		-	7	2541	1968	WD repeat-containing 44-like [Populus euphratica]	655	gi|566169735|ref|XP_006382834.1|WD-40 repeat family protein [Populus trichocarpa]gi|550338204|gb|ERP60631.1|WD-40 repeat family protein [Populus trichocarpa]	XP_006382834, ERP60631	0.0	673	466
Pe171P13.10		-	20	9727	4035	TONSOKU isoform X1	1344	gi|743827382|ref|XP_011023008.1|PREDICTED: protein TONSOKU isoform X1 [Populus euphratica]	XP_011023008	0.0	1359	1094
Pe171P13.11		-	2	1227	1146	Uncharacterized acetyltransferase At3g50280-like	381	gi|255585099|ref|XP_002533255.1|PREDICTED: uncharacterized acetyltransferase At3g50280 [Ricinus communis]gi|223526911|gb|EEF29117.1|Anthranilate N-benzoyltransferase protein, putative [Ricinus communis]	XP_002533255, EEF29117	1.96E-149	409	289
Pe171P13.12		-	2	461	348	TONSOKU isoform X2	115	gi|641839447|gb|KDO58377.1|hypothetical protein CISIN_1g0007421mg, partial [Citrus sinensis]	KDO58377	4.43E-45	108	95

Pe171P13.13		-	1	638	330	Uncharacterized acetyltransferase At3g50280-like	109	gi|255585099|ref|XP_002533255.1|PREDICTED: uncharacterized acetyltransferase At3g50280 [Ricinus communis]gi|223526911|gb|EEF29117.1|Anthranilate N-benzoyltransferase protein, putative [Ricinus communis]	XP_002533255, EEF29117	5.79E-40	109	88
Pe171P13.14		-	1	1332	1332	Uncharacterized acetyltransferase At3g50280-like	443	gi|566161010|ref|XP_002304178.2|anthranilate N-hydroxycinnamoyl/benzoyltransferase family protein [Populus trichocarpa]gi|550342474|gb|EEE79157.2|anthranilate N-hydroxycinnamoyl/benzoyltransferase family protein [Populus trichocarpa]	XP_002304178, EEE79157	0.0	445	338
Pe171P13.15		-	1	1562	1332	Uncharacterized acetyltransferase At3g50280-like	443	gi|566161010|ref|XP_002304178.2|anthranilate N-hydroxycinnamoyl/benzoyltransferase family protein [Populus trichocarpa]gi|550342474|gb|EEE79157.2|anthranilate N-hydroxycinnamoyl/benzoyltransferase family protein [Populus trichocarpa]	XP_002304178, EEE79157	0.0	445	339
Pe171P13.16		-	1	1332	1332	Uncharacterized acetyltransferase At3g50280-like	443	gi|566161010|ref|XP_002304178.2|anthranilate N-hydroxycinnamoyl/benzoyltransferase family protein [Populus trichocarpa]gi|550342474|gb|EEE79157.2|anthranilate N-hydroxycinnamoyl/benzoyltransferase family protein [Populus trichocarpa]	XP_002304178, EEE79157	0.0	445	336
Pe171P13.17	3 isoforms	-	3	1766	405	UPF0235 c15orf40 homolog isoform X2 [Ziziphus jujuba]	121	gi|225431557|ref|XP_002282176.1|PREDICTED: UPF0235 protein C15orf40 [Vitis vinifera]gi|147866968|emb|CAN83055.1|hypothetical protein VITISV_009894 [Vitis vinifera]	XP_002282176, CAN83055	1.88E-31	92	76
Pe171P13.18		-	1	1117	612	Truncated Kunitz trypsin inhibitor family [Populus trichocarpa]	203	gi|743935298|ref|XP_011012012.1|PREDICTED: alpha-amylase/subtilisin inhibitor-like [Populus euphratica]	XP_011012012	9.78E-84	202	153
Pe171P13.19		-	2	617	501	Truncated Kunitz trypsin inhibitor family [Populus trichocarpa]	166	gi|743935298|ref|XP_011012012.1|PREDICTED: alpha-amylase/subtilisin inhibitor-like [Populus euphratica]	XP_011012012	6.99E-49	204	118
Pe171P13.20		+	1	872	675	RING-H2 finger ATL78	224	gi|802725453|ref|XP_012086051.1|PREDICTED: RING-H2 finger protein ATL78-like [Jatropha curcas]gi|643713146|gb|KDP26113.1|hypothetical protein JCGZ_22214 [Jatropha curcas]	XP_012086051, KDP26113	1.85E-110	222	188
Pe171P13.21		+	6	2342	1416	Actin-7 [Ricinus communis]	471	gi|976924565|gb|KVI08516.1|Actin/actin-like conserved site-containing protein [Cynara cardunculus var. scolymus]	KVI08516	0.0	381	379
Pe171P13.22		-	1	471	471	Kd heat shock family [Populus trichocarpa]	156	gi|802538756|ref|XP_012068597.1|PREDICTED: 17.3 kDa class I heat shock protein-like [Jatropha curcas]gi|802563491|ref|XP_012066923.1|PREDICTED: 17.3 kDa class I heat shock protein-like [Jatropha curcas]gi|643735633|gb|KDP42161.1|hypothetical protein JCGZ_02891 [Jatropha curcas]gi|643741012|gb|KDP46582.1|hypothetical protein JCGZ_08554 [Jatropha curcas]	XP_012068597, XP_012066923, KDP42161, KDP46582	1.38E-88	157	146
Pe171P13.23		+	2	2761	1512	Trihelix transcription factor GT-2-like	503	gi|743904660|ref|XP_011045710.1|PREDICTED: trihelix transcription factor GT-2-like [Populus euphratica]	XP_011045710	0.0	509	386
Pe171P13.24	1 isoform	-	7	7273	798	DNAJ heat shock N-terminal domain-containing family [Populus trichocarpa]	265	gi|590708280|ref|XP_007048232.1|Chaperone DNAJ-domain superfamily protein, putative [Theobroma cacao]gi|508700493|gb|EOX92389.1|Chaperone	XP_007048232, EOX92389	2.44E-108	278	205

								DNAJ-domain superfamily protein, putative [Theobroma cacao]				
Pe171P13.25		+	10	5762	2859	Eukaryotic translation initiation factor 3 subunit I-like	952	gi|802597734|ref|XP_012072406.1|PREDICTED : eukaryotic translation initiation factor 3 subunit I-like [Jatropha curcas]gi|643730769|gb|KDP38201.1|hypothetical protein JCGZ_04844 [Jatropha curcas]	XP_012072406, KDP38201	0.0	303	293
Pe173B16.1	Incomplete 5'	+	1	2747	2296	Translocase of chloroplast chloroplastic-like	768	gi|965603883|dbj|BAT91060.1|hypothetical protein VIGAN_06236600 [Vigna angularis var. angularis]	BAT91060	0.0	769	711
Pe173B16.2		-	1	839	570	Dirigent 4-like	189	gi|566182627|ref|XP_002312128.2|disease resistance-responsive family protein [Populus trichocarpa]gi|550332532|gb|EEE89495.2|disease resistance-responsive family protein [Populus trichocarpa]	XP_002312128, EEE89495	1.59E-88	184	154
Pe173B16.3		-	5	1931	1566	Amidase	521	gi|743884472|ref|XP_011037287.1|PREDICTED : putative amidase C869.01 [Populus euphratica]	XP_011037287	0.0	511	460
Pe173B16.4		-	3	1183	1005	Protodermal factor 1	334	gi|802707714|ref|XP_012084370.1|PREDICTED : protodermal factor 1 [Jatropha curcas]	XP_012084370	1.71E-135	311	242
Pe173B16.5		-	9	2982	1008	Probable magnesium transporter NIPA6 [Gossypium hirsutum]	335	gi|802707726|ref|XP_012084372.1|PREDICTED : probable magnesium transporter NIPA6 [Jatropha curcas]gi|643715637|gb|KDP27578.1|hypothetical protein JCGZ_19583 [Jatropha curcas]	XP_012084372, KDP27578	0.0	331	312
Pe173B16.6		-	1	519	519	Peptidyl-prolyl cis-trans isomerase family [Populus trichocarpa]	172	gi|224105235|ref|XP_002313736.1|peptidyl-prolyl cis-trans isomerase family protein [Populus trichocarpa]gi|566187778|ref|XP_006379277.1|hypothetical protein POPTR_0009s13270g [Populus trichocarpa]gi|118481352|gb|ABK92619.1|unknown [Populus trichocarpa]gi|118483196|gb|ABK93502.1|unknown [Populus trichocarpa]gi|222850144|gb|EEE87691.1|peptidyl-prolyl cis-trans isomerase family protein [Populus trichocarpa]gi|550331639|gb|ERP57074.1|hypothetical protein POPTR_0009s13270g [Populus trichocarpa]	XP_002313736, XP_006379277, ABK92619, ABK93502, EEE87691, ERP57074	2.28E-111	172	166
Pe173B16.7		-	32	15390	5916	Nucleoporin NUP188 homolog isoform X1	1971	gi|802707738|ref|XP_012084375.1|PREDICTED : uncharacterized protein LOC105643779 [Jatropha curcas]	XP_012084375	0.0	1973	1608
Pe173B16.8		-	2	2307	1530	Proline-rich 4-like	509	gi|566187774|ref|XP_006379276.1|hypothetical protein POPTR_0009s13250g [Populus trichocarpa]gi|550331637|gb|ERP57073.1|hypothetical protein POPTR_0009s13250g [Populus trichocarpa]	XP_006379276, ERP57073	5.64E-88	204	167
Pe173B16.9		-	8	3030	771	Translocon-associated subunit alpha-like	256	gi|643715642|gb|KDP27583.1|hypothetical protein JCGZ_19588 [Jatropha curcas]	KDP27583	2.33E-138	248	224
Pe173B16.10		-	11	2730	1014	Xap5 circadian timekeeper	337	gi|802707750|ref|XP_012084377.1|PREDICTED : protein XAP5 CIRCADIAN TIMEKEEPER isoform X1 [Jatropha curcas]gi|643715643|gb|KDP27584.1|hypothetical protein JCGZ_19589 [Jatropha curcas]	XP_012084377, KDP27584	0.0	337	322
Pe173B16.11	2 isoforms	+	5	2260	1020	Transcription factor bhlh121-like [Populus euphratica]	339	gi|590684731|ref|XP_007041927.1|Basic helix-loop-helix DNA-binding superfamily protein [Theobroma	XP_007041927, EOX97758	3.41E-130	336	263

							cacao]gi|508705862|gb|EOX97758.1|Basic helix-loop-helix DNA-binding superfamily protein [Theobroma cacao]				
Pe173B16.12	+	9	3076	963	Triosephosphate chloroplastic	320	gi|743884381|ref|XP_011037267.1|PREDICTED: triosephosphate isomerase, chloroplastic-like [Populus euphratica]	XP_011037267	0.0	320	284
Pe173B16.13	-	11	9849	6375	Urb2 isoform 2 [Theobroma cacao]	2124	gi|566167032|ref|XP_002305483.2|hypothetical protein POPTR_0004s17490g [Populus trichocarpa]gi|550341234|gb|EEE85994.2|hypothetical protein POPTR_0004s17490g [Populus trichocarpa]	XP_002305483, EEE85994	0.0	2115	1500
Pe173B16.14	+	4	2387	1725	Plant neutral invertase family isoform 1 [Theobroma cacao]	574	gi|590684809|ref|XP_007041939.1|Plant neutral invertase family protein isoform 1 [Theobroma cacao]gi|590684812|ref|XP_007041940.1|Plant neutral invertase family protein isoform 1 [Theobroma cacao]gi|590684816|ref|XP_007041941.1|Plant neutral invertase family protein isoform 1 [Theobroma cacao]gi|508705874|gb|EOX97770.1|Plant neutral invertase family protein isoform 1 [Theobroma cacao]gi|508705875|gb|EOX97771.1|Plant neutral invertase family protein isoform 1 [Theobroma cacao]gi|508705876|gb|EOX97772.1|Plant neutral invertase family protein isoform 1 [Theobroma cacao]	XP_007041939, XP_007041940, XP_007041941, EOX97770, EOX97771, EOX97772	0.0	566	517
Pe173B16.15	+	6	1853	819	ER lumen -retaining receptor	272	gi|802707828|ref|XP_012084391.1|PREDICTED: putative ER lumen protein-retaining receptor C28H8.4 [Jatropha curcas]gi|643715652|gb|KDP27593.1|hypothetical protein JCGZ_19598 [Jatropha curcas]	XP_012084391, KDP27593	0.0	272	267
Pe173B16.16	-	2	1342	1176	Type III polyketide synthase B-like	391	gi|590684827|ref|XP_007041944.1|Chalcone and stilbene synthase family protein [Theobroma cacao]gi|508705879|gb|EOX97775.1|Chalcone and stilbene synthase family protein [Theobroma cacao]	XP_007041944, EOX97775	0.0	388	363
Pe173B16.17	-	8	2781	795	5 -methylthioadenosine S-adenosylhomocysteine nucleosidase 2-like	264	gi|224078057|ref|XP_002305481.1|phosphorylase family protein [Populus trichocarpa]gi|118481001|gb|ABK92454.1|unknown [Populus trichocarpa]gi|222848445|gb|EEE85992.1|phosphorylase family protein [Populus trichocarpa]	XP_002305481, ABK92454, EEE85992	3.53E-149	266	233
Pe173B16.18	-	6	2139	1344	Cobra 1	447	gi|223531297|gb|EEF33139.1|Protein COBRA precursor, putative [Ricinus communis]	EEF33139	0.0	417	345
Pe173B16.19	+	2	991	885	Aquaporin TIP1-2-like	294	gi|854958783|gb|AKN79687.1|X-intrisic protein subfamily member 1;1 [Hevea brasiliensis]	AKN79687	2.51E-129	282	231
Pe173B16.20	-	5	3153	1098	Calcium-binding EF hand family [Populus trichocarpa]	365	gi|566167023|ref|XP_002305479.2|hypothetical protein POPTR_0004s17420g [Populus trichocarpa]gi|550341231|gb|EEE85990.2|hypothetical protein POPTR_0004s17420g [Populus trichocarpa]	XP_002305479, EEE85990	0.0	358	327
Pe173B16.21	+	19	8013	3219	Pentatricopeptide repeat-containing chloroplastic isoform X2	1072	gi|566167019|ref|XP_002306163.2|hypothetical protein POPTR_0004s17400g [Populus trichocarpa]gi|550341229|gb|EEE86674.2|hypothetical protein POPTR_0004s17400g [Populus trichocarpa]	XP_002306163, EEE86674	0.0	1108	834

Pe173B16.22		-	1	883	279	Auxin-induced 15A-like	92	gi|224105265|ref|XP_002313747.1|auxin-responsive family protein [Populus trichocarpa]gi|222850155|gb|EEE87702.1|auxin-responsive family protein [Populus trichocarpa]	XP_002313747, EEE87702	1.68E-36	99	81
Pe173B16.23		+	1	475	372	Auxin-induced 15A-like	123	gi|224103265|ref|XP_002312989.1|hypothetical protein POPTR_0009s13020g [Populus trichocarpa]gi|222849397|gb|EEE86944.1|hypothetical protein POPTR_0009s13020g [Populus trichocarpa]	XP_002312989, EEE86944	2.27E-34	99	76
Pe173B16.24		-	1	1041	294	Auxin-induced 15A-like [Brassica napus]	101	gi|565439268|ref|XP_006282478.1|hypothetical protein CARUB_v10006095mg [Capsella rubella]gi|482551183|gb|EOA15376.1|hypothetical protein CARUB_v10006095mg [Capsella rubella]	XP_006282478, EOA15376	3.15E-49	104	93
Pe175N8.1		+	1	537	537	TMV resistance N [Morus notabilis]	178	gi|118489985|gb|ABK96789.1|unknown [Populus trichocarpa x Populus deltoides]	ABK96789	1.17E-78	151	131
Pe175N8.2	1 isoform	+	3	3292	2316	TMV resistance N-like	771	gi|566205729|ref|XP_002321495.2|hypothetical protein POPTR_0015s04000g [Populus trichocarpa]gi|550321898|gb|EEF05622.2|hypothetical protein POPTR_0015s04000g [Populus trichocarpa]	XP_002321495, EEF05622	0.0	771	610
Pe175N8.3		-	27	14245	3663	Phospholipid-transporting atpase 3	1220	gi|802787684|ref|XP_012091990.1|PREDICTED: phospholipid-transporting ATPase 3 isoform X1 [Jatropha curcas]	XP_012091990	0.0	1219	1150
Pe175N8.4		-	6	2806	807	Transcription factor bhlh79	268	gi|255558300|ref|XP_002520177.1|PREDICTED: transcription factor bHLH79 isoform X1 [Ricinus communis]gi|223540669|gb|EEF42232.1|DNA binding protein, putative [Ricinus communis]	XP_002520177, EEF42232	4.32E-109	274	211
Pe175N8.5	1 isoform	-	9	5378	1605	CW14 isoform 1 [Theobroma cacao]	534	gi|566189114|ref|XP_002315697.2|hypothetical protein POPTR_0010s04930g [Populus trichocarpa]gi|550329086|gb|EEF01868.2|hypothetical protein POPTR_0010s04930g [Populus trichocarpa]	XP_002315697, EEF01868	0.0	549	468
Pe175N8.6	3 isoforms	+	7	6144	1974	Probable beta-1,3-galactosyltransferase 19	657	gi|1000964246|ref|XP_002520170.2|PREDICTED: probable beta-1,3-galactosyltransferase 17 [Ricinus communis]	XP_002520170	0.0	663	566
Pe175N8.7		-	1	915	915	Wall-associated receptor kinase-like 15	304	gi|255558284|ref|XP_002520169.1|PREDICTED: uncharacterized protein LOC8287048 [Ricinus communis]gi|223540661|gb|EEF42224.1|conserved hypothetical protein [Ricinus communis]	XP_002520169, EEF42224	1.48E-165	285	260
Pe175N8.8		+	1	2253	2253	Pentatricopeptide repeat-containing mitochondrial [Vitis vinifera]	750	gi|1000964164|ref|XP_002520166.2|PREDICTED: pentatricopeptide repeat-containing protein At1g05670, mitochondrial [Ricinus communis]	XP_002520166	0.0	749	631
Pe175N8.9		+	2	996	699	Glutathione S-transferase U17-like	232	gi|327342604|gb|AEA50895.1|glutathione S-transferase [Populus alba x Populus glandulosa]	AEA50895	2.26E-112	226	196
Pe175N8.10		-	1	1905	1905	Probable serine threonine-kinase At1g18390	634	gi|743935009|ref|XP_011011859.1|PREDICTED: probable serine/threonine-protein kinase At1g18390 [Populus euphratica]	XP_011011859	0.0	633	498
Pe175N8.11		+	4	5361	1593	Actin cross-linking [Theobroma cacao]	530	gi|802787608|ref|XP_012091970.1|PREDICTED: uncharacterized protein LOC105649794 isoform X2 [Jatropha curcas]gi|643704183|gb|KDP21247.1|hypothetical protein JCGZ_21718 [Jatropha curcas]	XP_012091970, KDP21247	0.0	528	471
Pe175N8.12	1 isoform	+	2	2794	792	DNAJ heat shock family [Populus trichocarpa]	263	gi|566184951|ref|XP_002311798.2|DNAJ heat shock family protein [Populus trichocarpa]gi|550333496|gb|EEE89165.2|DNAJ heat shock family protein [Populus trichocarpa]	XP_002311798, EEE89165	1.19E-123	312	223

Pe175N8.13		-	4	3845	1830	NRT1 PTR FAMILY -like	619	gi|255558256|ref|XP_002520155.1|PREDICTED : protein NRT1/ PTR FAMILY 2.13 [Ricinus communis]gi|223540647|gb|EEF42210.1|nitrate transporter, putative [Ricinus communis]	XP_002520155, EEF42210	0.0	611	515
Pe175N8.14		-	2	1981	969	PREDICTED: uncharacterized protein LOC8287027 isoform X1	322	gi|1000964189|ref|XP_015575266.1|PREDICTE D: uncharacterized protein LOC8287027 isoform X1 [Ricinus communis]	XP_015575266	1.83E-143	323	269
Pe175N8.15	5 isoforms	+	8	3189	1224	Ultraviolet-B receptor UVR8 isoform X2	407	gi|1000964268|ref|XP_015575281.1|PREDICTE D: ultraviolet-B receptor UVR8 [Ricinus communis]gi|1000964271|ref|XP_015575282.1|P REDICTED: ultraviolet-B receptor UVR8 [Ricinus communis]gi|1000964274|ref|XP_015575283.1|P REDICTED: ultraviolet-B receptor UVR8 [Ricinus communis]	XP_015575281, XP_015575282, XP_015575283	5.33E-174	399	302
Pe175N8.16		-	2	1812	1524	Ribonuclease h at1g65750	507	gi|727649960|ref|XP_010495633.1|PREDICTED : uncharacterized protein LOC104772751 [Camelina sativa]	XP_010495633	3.53E-73	486	240
Pe175N8.17		-	1	249	249	---Na---	82	No Blast Hit				
Pe175N8.18		-	1	324	324	---Na---	107	No Blast Hit				
Pe175N8.19		+	1	219	219	---Na---	72	No Blast Hit				
Pe175N8.20		+	1	588	588	---Na---	195	No Blast Hit				
Pe185D11.1		+	3	575	399	MATE efflux family [Medicago truncatula]	132	gi|566204385|ref|XP_006375528.1|hypothetical protein POPTR_0014s15130g, partial [Populus trichocarpa]gi|550324245|gb|ERP53325.1|hypoth etical protein POPTR_0014s15130g, partial [Populus trichocarpa]	XP_006375528, ERP53325	1.61E-60	129	113
Pe185D11.2		+	2	710	309	---Na---	102	No Blast Hit				
Pe185D11.3		+	4	1231	447	ATP-dependent rna helicase dbp7	148	gi|1000950046|ref|XP_015579709.1|PREDICTE D: uncharacterized protein LOC8259691 [Ricinus communis]	XP_015579709	3.59E-74	148	131
Pe185D11.4	1 isoform	-	5	2375	699	PREDICTED: uncharacterized protein LOC105125699	232	gi|743833569|ref|XP_011024565.1|PREDICTED : uncharacterized protein LOC105125699 [Populus euphratica]	XP_011024565	5.67E-130	232	207
Pe185D11.5		+	8	3912	1098	SOS response-associated peptidase	365	gi|743833573|ref|XP_011024566.1|PREDICTED : embryonic stem cell-specific 5-hydroxymethylcytosine-binding protein-like [Populus euphratica]	XP_011024566	2.62E-165	370	291
Pe185D11.6		+	1	231	231	---Na---	76	No Blast Hit				
Pe185D11.7		+	1	288	288	---Na---	95	No Blast Hit				
Pe185D11.8		-	1	297	297	PREDICTED: uncharacterized protein LOC105644870	98	gi|802546600|ref|XP_012085759.1|PREDICTED : uncharacterized protein LOC105644870 [Jatropha curcas]	XP_012085759	3.01E-37	98	84
Pe185D11.9		-	2	1880	393	DCTP pyrophosphatase 1-like	130	gi|703115081|ref|XP_010100812.1|hypothetical protein L484_015842 [Morus notabilis]gi|587896019|gb|EXB84510.1|hypotheti cal protein L484_015842 [Morus notabilis]	XP_010100812, EXB84510	3.91E-67	132	115
Pe185D11.10		-	3	702	327	Epidermal patterning factor 9	108	gi|823189691|ref|XP_012490914.1|PREDICTED : EPIDERMAL PATTERNING FACTOR-like protein 9 isoform X2 [Gossypium raimondii]gi|763775461|gb|KJB42584.1|hypothet ical protein B456_007G158800 [Gossypium raimondii]	XP_012490914, KJB42584	6.51E-37	109	82

Pe185D11.11		-	1	783	783	Cysteine-rich repeat secretory 55-like	260	gi|1029136145|ref|XP_016746151.1|PREDICTED: cysteine-rich repeat secretory protein 55-like [Gossypium hirsutum]	XP_016746151	1.09E-121	243	209
Pe185D11.12		+	2	2293	651	Ras-related RABA5b	216	gi|802546474|ref|XP_012085038.1|PREDICTED: ras-related protein RABA5b [Jatropha curcas]gi|643739507|gb|KDP45261.1|hypothetical protein JCGZ_15126 [Jatropha curcas]	XP_012085038, KDP45261	4.05E-127	216	200
Pe185D11.13		+	1	537	537	PREDICTED: uncharacterized protein LOC104220956	178	gi|698554473|ref|XP_009770224.1|PREDICTED: uncharacterized protein LOC104220956 [Nicotiana sylvestris]	XP_009770224	1.44E-13	148	76
Pe185D11.14		+	1	624	624	Hypothetical protein GLYMA_06G246200	207	gi|947106940|gb|KRH55323.1|hypothetical protein GLYMA_06G246200 [Glycine max]	KRH55323	1.42E-21	122	74
Pe185D11.15		-	1	201	201	---Na---	66	No Blast Hit				
Pe185D11.16		+	1	315	315	---Na---	104	No Blast Hit				
Pe185D11.17		-	3	1919	1209	Cytochrome b561 and DOMON domain-containing At3g25290-like	402	gi|743833617|ref|XP_011024578.1|PREDICTED: cytochrome b561 and DOMON domain-containing protein At3g25290 [Populus euphratica]	XP_011024578	0.0	397	320
Pe185D11.18		-	1	1292	720	Auxin-induced in root cultures 12	239	gi|743833623|ref|XP_011024579.1|PREDICTED: auxin-induced in root cultures protein 12 [Populus euphratica]	XP_011024579	7.45E-85	258	176
Pe185D11.19		+	2	812	240	60S ribosomal L32-1-like	79	gi|255585616|ref|XP_002533495.1|PREDICTED: 60S ribosomal protein L32-1 [Ricinus communis]gi|223526639|gb|EEF28882.1|60S ribosomal protein L32, putative [Ricinus communis]	XP_002533495, EEF28882	3.74E-03	30	29
Pe185D11.20		+	1	315	315	---Na---	104	No Blast Hit				
Pe185D11.21		+	2	360	246	Ribosomal L32e isoform 1 [Theobroma cacao]	81	gi|313586537|gb|ADR71279.1|60S ribosomal protein L32A [Hevea brasiliensis]gi|313586539|gb|ADR71280.1|60S ribosomal protein L32B [Hevea brasiliensis]	ADR71279, ADR71280	1.34E-44	81	78
Pe185D11.22		-	2	2045	1299	Serine threonine- kinase OXI1-like	432	gi|255585618|ref|XP_002533496.1|PREDICTED: serine/threonine-protein kinase OXI1 [Ricinus communis]gi|223526640|gb|EEF28883.1|serine/threonine protein kinase, putative [Ricinus communis]	XP_002533496, EEF28883	1.20E-169	441	321
Pe185D11.23		+	3	610	405	---Na---	134	No Blast Hit				
Pe185D11.24		+	7	3253	588	Uncharacterized HIT MT1300	195	gi|802546486|ref|XP_012085108.1|PREDICTED: hit family protein 1 isoform X1 [Jatropha curcas]gi|643739514|gb|KDP45268.1|hypothetical protein JCGZ_15133 [Jatropha curcas]	XP_012085108, KDP45268	2.41E-92	194	164
Pe185D11.25		+	8	1774	858	E3 ubiquitin- ligase CHIP	285	gi|802546490|ref|XP_012085129.1|PREDICTED: E3 ubiquitin-protein ligase CHIP [Jatropha curcas]gi|643739515|gb|KDP45269.1|hypothetical protein JCGZ_15134 [Jatropha curcas]	XP_012085129, KDP45269	1.19E-149	282	239
Pe185D11.26		-	2	2304	1374	U-box domain-containing 9	457	gi|703115023|ref|XP_010100798.1|U-box domain-containing protein 9 [Morus notabilis]gi|587896005|gb|EXB84496.1|U-box domain-containing protein 9 [Morus notabilis]	XP_010100798, EXB84496	0.0	457	417
Pe185D11.27		-	1	1733	885	Sulfate thiosulfate import ATP-binding [Theobroma cacao]	294	gi|568862695|ref|XP_006484811.1|PREDICTED: uncharacterized protein LOC102616317 [Citrus sinensis]gi|641841966|gb|KDO60875.1|hypothetical protein CISIN_1g022485mg [Citrus sinensis]	XP_006484811, KDO60875	5.98E-139	299	241
Pe185D11.28	2 isoforms	+	8	3554	1725	Transcription factor bhlh62	574	gi|566159858|ref|XP_002303073.2|basic helix-loop-helix family protein [Populus	XP_002303073, EEE82346	0.0	579	452

								trichocarpa]gi|550345773|gb|EEE82346.2|basic helix-loop-helix family protein [Populus trichocarpa]				
Pe185D11.29		-	5	1879	663	40S ribosomal S8-like	220	gi|823147362|ref|XP_012473594.1|PREDICTED: 40S ribosomal protein S8-like [Gossypium raimondii]gi|1029106052|ref|XP_016703938.1|PREDICTED: 40S ribosomal protein S8-like [Gossypium hirsutum]gi|763755328|gb|KJB22659.1|hypothetical protein B456_004G059300 [Gossypium raimondii]	XP_012473594, XP_016703938, KJB22659	1.84E-130	204	195
Pe185D11.30		-	12	4778	1722	Peptidyl-prolyl cis-trans isomerase FKBP62-like	573	gi|224065421|ref|XP_002301809.1|hypothetical protein POPTR_0002s24970g [Populus trichocarpa]gi|222843535|gb|EEE81082.1|hypothetical protein POPTR_0002s24970g [Populus trichocarpa]	XP_002301809, EEE81082	0.0	575	549
Pe185D11.31		-	6	2510	462	Peptidyl-prolyl cis-trans isomerase FKBP15-1-like	153	gi|802546504|ref|XP_012085211.1|PREDICTED: peptidyl-prolyl cis-trans isomerase FKBP15-1 [Jatropha curcas]gi|643739522|gb|KDP45276.1|hypothetical protein JCGZ_15141 [Jatropha curcas]	XP_012085211, KDP45276	4.83E-84	142	133
Pe185D11.32		+	3	2673	1410	Probable glucan endo-1,3-beta-glucosidase A6	469	gi|566159849|ref|XP_006386885.1|3-glucanase family protein [Populus trichocarpa]gi|550345770|gb|ERP64682.1|3-glucanase family protein [Populus trichocarpa]	XP_006386885, ERP64682	0.0	462	413
Pe185D11.33		+	2	2125	1188	UV-B-induced chloroplastic-like	395	gi|802546518|ref|XP_012085283.1|PREDICTED: uncharacterized protein LOC105644513 [Jatropha curcas]gi|643739526|gb|KDP45280.1|hypothetical protein JCGZ_15145 [Jatropha curcas]	XP_012085283, KDP45280	1.98E-156	336	271
Pe185D11.34		+	1	450	450	Hypothetical protein JCGZ_15146	149	gi|643739527|gb|KDP45281.1|hypothetical protein JCGZ_15146 [Jatropha curcas]	KDP45281	9.72E-47	149	115
Pe185D11.35		+	4	2758	990	Quinone-oxidoreductase chloroplastic	329	gi|802546524|ref|XP_012085318.1|PREDICTED: putative quinone-oxidoreductase homolog, chloroplastic [Jatropha curcas]	XP_012085318	0.0	329	301
Pe185D11.36	Incomplete 3'	+	4	1639	462	Quinone-oxidoreductase chloroplastic	154	gi|802546524|ref|XP_012085318.1|PREDICTED: putative quinone-oxidoreductase homolog, chloroplastic [Jatropha curcas]	XP_012085318	4.29E-79	150	137
Pe185J16.1	Incomplete3'	-	2	595	296	Myb domain [Theobroma cacao]	98	gi|590693272|ref|XP_007044287.1|Myb domain protein 62, putative [Theobroma cacao]gi|508708222|gb|EOY00119.1|Myb domain protein 62, putative [Theobroma cacao]	XP_007044287, EOY00119	1.15E-46	98	88
Pe185J16.2	2 isoforms	+	6	2643	825	Basic helix-loop-helix family [Populus trichocarpa]	274	gi|743904927|ref|XP_011045850.1|PREDICTED: transcription factor BEE 1 isoform X2 [Populus euphratica]	XP_011045850	1.35E-124	273	227
Pe185J16.3	2 isoforms	+	9	5775	2091	Gb: [Theobroma cacao]	696	gi|802797420|ref|XP_012093017.1|PREDICTED: uncharacterized protein LOC105650685 [Jatropha curcas]gi|802797424|ref|XP_012093018.1|PREDICTED: uncharacterized protein LOC105650685 [Jatropha curcas]gi|643686964|gb|KDP20129.1|hypothetical protein JCGZ_05898 [Jatropha curcas]	XP_012093017, XP_012093018, KDP20129	0.0	683	574
Pe185J16.4		+	7	4234	2283	Chloroplastic group IIA intron splicing facilitator chloroplastic-like	760	gi|743904944|ref|XP_011045859.1|PREDICTED: chloroplastic group IIA intron splicing facilitator CRS1, chloroplastic isoform X1 [Populus euphratica]	XP_011045859	0.0	759	623

Pe185J16.5		-	7	2759	1299	Hydroxyproline-rich glyco [Populus trichocarpa]	432	gi|224121298|ref|XP_002318548.1|hydroxyproline-rich glycoprotein [Populus trichocarpa]gi|222859221|gb|EEE96768.1|hydroxyproline-rich glycoprotein [Populus trichocarpa]	XP_002318548, EEE96768	8.87E-146	437	306
Pe185J16.6		-	3	1648	951	Cup-shaped cotyledon 2	316	gi|743930144|ref|XP_011009316.1|PREDICTED: NAC domain-containing protein 100-like [Populus euphratica]	XP_011009316	2.13E-172	321	279
Pe185J16.7		+	6	2461	1764	Checkpoint serine threonine- kinase isoform 1 [Theobroma cacao]	587	gi|566196903|ref|XP_002317914.2|hypothetical protein POPTR_0012s05310g [Populus trichocarpa]gi|550326429|gb|EEE96134.2|hypothetical protein POPTR_0012s05310g [Populus trichocarpa]	XP_002317914, EEE96134	0.0	592	504
Pe185J16.8	1 isoform	+	3	2959	516	PREDICTED: uncharacterized protein LOC105650678 [Jatropha curcas]	171	gi|802797381|ref|XP_012093007.1|PREDICTED: uncharacterized protein LOC105650678 [Jatropha curcas]gi|643686956|gb|KDP20121.1|hypothetical protein JCGZ_05890 [Jatropha curcas]	XP_012093007, KDP20121	1.77E-69	175	145
Pe185J16.9	1 isoform	-	4	3325	585	Ribulose-1,5-bisphosphate carboxylase oxygenase small subunit (chloroplast) [Gossypium hirsutum]	194	gi|255582745|ref|XP_002532149.1|PREDICTED: ribulose bisphosphate carboxylase small chain, chloroplastic [Ricinus communis]gi|223528159|gb|EEF30223.1|Ribulose bisphosphate carboxylase small chain, chloroplast precursor, putative [Ricinus communis]	XP_002532149, EEF30223	6.96E-32	80	69
Pe185J16.10		+	2	1456	687	LOB domain-containing 41-like	228	gi|223526744|gb|EEF28973.1|LOB domain-containing protein, putative [Ricinus communis]	EEF28973	7.92E-85	241	179
Pe185J16.11		+	2	1766	318	NADH dehydrogenase [ubiquinone] 1 beta subcomplex subunit 10-B-like [Gossypium hirsutum]	105	gi|802797369|ref|XP_012093003.1|PREDICTED: NADH dehydrogenase [ubiquinone] 1 beta subcomplex subunit 10-B [Jatropha curcas]	XP_012093003	6.03E-58	106	98
Pe185J16.12	1 isoform	+	4	1258	912	Ribonuclease 3 2	303	gi|1021024978|gb|KZM82766.1|hypothetical protein DCAR_030335 [Daucus carota subsp. sativus]	KZM82766	6.10E-31	289	147
Pe185J16.13		-	5	2881	1710	FAR1-RELATED SEQUENCE 5-like	569	gi|698560284|ref|XP_009771804.1|PREDICTED: putative protein FAR1-RELATED SEQUENCE 10 [Nicotiana sylvestris]	XP_009771804	6.17E-49	178	120
Pe185J16.14		-	1	966	966	Ethylene-responsive transcription factor ERF118 [Vitis vinifera]	321	gi|566206211|ref|XP_006374367.1|hypothetical protein POPTR_0015s06470g [Populus trichocarpa]gi|550322126|gb|ERP52164.1|hypothetical protein POPTR_0015s06470g [Populus trichocarpa]	XP_006374367, ERP52164	7.08E-76	337	213
Pe185J16.15		+	1	1055	402	Nim1-interacting 2	133	gi|802797349|ref|XP_012092998.1|PREDICTED: uncharacterized protein LOC105650670 [Jatropha curcas]gi|643686947|gb|KDP20112.1|hypothetical protein JCGZ_05881 [Jatropha curcas]	XP_012092998, KDP20112	7.52E-03	123	75
Pe185J16.16		+	1	762	762	Dentin sialophospho -like	253	gi|802797345|ref|XP_012092997.1|PREDICTED: uncharacterized protein LOC105650669 [Jatropha curcas]gi|643686946|gb|KDP20111.1|hypothetical protein JCGZ_05880 [Jatropha curcas]	XP_012092997, KDP20111	2.30E-57	255	166
Pe185J16.17		+	2	1346	615	AT-rich interactive domain-containing [Theobroma cacao]	204	gi|802797341|ref|XP_012092996.1|PREDICTED: uncharacterized protein LOC105650668 [Jatropha curcas]gi|643686945|gb|KDP20110.1|hypothetical protein JCGZ_05879 [Jatropha curcas]	XP_012092996, KDP20110	1.68E-80	214	160
Pe185J16.18		+	3	961	639	YLS3	212	gi|566206187|ref|XP_006374355.1|hypothetical protein POPTR_0015s06360g [Populus	XP_006374355, XP_006374359,	1.83E-57	199	142

								trichocarpa]gi|566206195|ref|XP_006374359.1|protease inhibitor/seed storage/lipid transfer family protein [Populus trichocarpa]gi|550322114|gb|ERP52152.1|hypothetical protein POPTR_0015s06360g [Populus trichocarpa]gi|550322118|gb|ERP52156.1|protease inhibitor/seed storage/lipid transfer family protein [Populus trichocarpa]	ERP52152, ERP52156			
Pe185J16.19		+	3	447	237	Unnamed protein product	78	gi|297734194|emb|CBI15441.3|unnamed protein product [Vitis vinifera]	CBI15441	5.61E-08	85	55
Pe185J16.20		+	3	462	252	Heavy metal-associated isoprenylated plant 25-like	83	gi|1028969875|ref|XP_016728446.1|PREDICTED: heavy metal-associated isoprenylated plant protein 25-like [Gossypium hirsutum]	XP_016728446	1.76E-35	80	73
Pe185J16.21		+	6	4519	1755	Aluminum-activated malate transporter 9	584	gi|566206183|ref|XP_006374353.1|hypothetical protein POPTR_0015s06340g [Populus trichocarpa]gi|550322112|gb|ERP52150.1|hypothetical protein POPTR_0015s06340g [Populus trichocarpa]	XP_006374353, ERP52150	0.0	587	499
Pe185J16.22		+	1	3239	1500	ASPARTIC PROTEASE IN GUARD CELL 1-like	499	gi|802797309|ref|XP_012092987.1|PREDICTED: protein ASPARTIC PROTEASE IN GUARD CELL 1 [Jatropha curcas]gi|643686935|gb|KDP20100.1|hypothetical protein JCGZ_05869 [Jatropha curcas]	XP_012092987, KDP20100	0.0	493	419
Pe185J16.23	1 isoform	+	4	2100	1122	PLAC8 family [Theobroma cacao]	373	gi|743931453|ref|XP_011009997.1|PREDICTED: protein PLANT CADMIUM RESISTANCE 6-like isoform X1 [Populus euphratica]	XP_011009997	6.90E-114	404	258
Pe185J16.24		-	21	8773	2367	STOMATAL CYTOKINESIS-DEFECTIVE 1 family [Populus trichocarpa]	788	gi|1000982347|ref|XP_015584441.1|PREDICTED: DENN domain and WD repeat-containing protein SCD1 [Ricinus communis]	XP_015584441	0.0	789	750
Pe186E19.1-2		+	14	7450	2091	Copper-transporting atpase chloroplastic	347	gi|743921834|ref|XP_011004985.1|PREDICTED: copper-transporting ATPase PAA1, chloroplastic-like [Populus euphratica]	XP_011004985	0.0	332	313
Pe186E19.3		+	5	2922	2199	Pumilio homolog chloroplastic	732	gi|1009137772|ref|XP_015886236.1|PREDICTED: uncharacterized protein LOC107421499 [Ziziphus jujuba]	XP_015886236	0.0	779	518
Pe186E19.4		-	4	1370	978	Peroxidase 43	325	gi|1028936978|ref|XP_016711666.1|PREDICTED: peroxidase 43-like [Gossypium hirsutum]	XP_016711666	0.0	317	278
Pe186E19.5	3 isoforms	+	10	6043	1548	Dentin sialophospho isoform X1 [Cucumis melo]	527	gi|1029121789|ref|XP_016739342.1|PREDICTED: protein LNK1-like isoform X3 [Gossypium hirsutum]	XP_016739342	2.97E-123	565	320
Pe186E19.6		+	2	1363	357	Lipid transfer	118	gi|823243397|ref|XP_012454350.1|PREDICTED: non-specific lipid-transfer protein-like [Gossypium raimondii]gi|1029077556|ref|XP_016698971.1|PREDICTED: non-specific lipid-transfer protein-like [Gossypium hirsutum]gi|7012719|gb|AAF35184.1|AF195863_1lipid transfer protein precursor [Gossypium hirsutum]gi|208427039|gb|ACI26701.1|lipid transfer protein [Gossypium hirsutum]gi|403391431|gb|AFR43273.1|lipid transfer protein precursor [Gossypium raimondii]gi|403391433|gb|AFR43274.1|lipid transfer protein precursor [Gossypium hirsutum]gi|403391435|gb|AFR43275.1|lipid transfer protein precursor [Gossypium barbadense]gi|763804574|gb|KJB71512.1|hypoth	XP_012454350, XP_016698971, AAF35184, ACI26701, AFR43273, AFR43274, AFR43275, KJB71512	2.08E-41	120	96

								etical protein B456_011G128200 [Gossypium raimondii]				
Pe186E19.7	6 isoforms	+	7	3237	759	Endoplasmic reticulum-Golgi intermediate compartment 3-like	252	gi|590688586|ref|XP_007042992.1|Endoplasmic reticulum vesicle transporter protein [Theobroma cacao]gi|508706927|gb|EOX98823.1|Endoplasmic reticulum vesicle transporter protein [Theobroma cacao]	XP_007042992, EOX98823	6.35E-146	248	229
Pe186E19.8		+	5	2486	1350	5 -3 exoribonuclease-like [Gossypium hirsutum]	449	gi|802711239|ref|XP_012084609.1|PREDICTED: uncharacterized protein LOC105643976 [Jatropha curcas]gi|643715123|gb|KDP27373.1|hypothetical protein JCGZ_20197 [Jatropha curcas]	XP_012084609, KDP27373	0.0	453	390
Pe186E19.9		-	2	2464	1629	Probable inactive receptor kinase At2g26730	542	gi|223530209|gb|EEF32117.1|serine-threonine protein kinase, plant-type, putative [Ricinus communis]	EEF32117	0.0	499	391
Pe186E19.10		-	3	1359	987	Peroxidase N1-like	328	gi|225626273|gb|ACN97186.1|peroxidase [Populus trichocarpa]	ACN97186	0.0	323	284
Pe186E19.11		+	1	1979	252	Lipid transfer	83	gi|345104195|gb|AEN70919.1|lipid transfer protein [Gossypium mustelinum]gi|345104197|gb|AEN70920.1|lipid transfer protein [Gossypium mustelinum]	AEN70919, AEN70920	1.80E-09	48	42
Pe186E19.12	2 isoforms	-	1	3704	1362	MTL1-like [Jatropha curcas]	453	gi|1000950698|ref|XP_002526903.2|PREDICTED: uncharacterized protein LOC8270482, partial [Ricinus communis]	XP_002526903	1.12E-164	411	312
Pe186E19.13		+	4	2091	702	3-hydroxyacyl-[acyl-carrier-] dehydratase -like	233	gi|924434384|gb|ALB76802.1|hydroxyacyl-ACP dehydratase [Jatropha curcas]	ALB76802	1.92E-118	244	206
Pe186E19.14		-	2	879	780	Chlorophyll a-b binding CP24 chloroplastic	259	gi|743922235|ref|XP_011005195.1|PREDICTED: chlorophyll a-b binding protein CP24 10A, chloroplastic-like [Populus euphratica]gi|743941455|ref|XP_011015216.1|PREDICTED: chlorophyll a-b binding protein CP24 10A, chloroplastic-like [Populus euphratica]	XP_011005195, XP_011015216	1.10E-156	259	242
Pe186E19.15		+	3	770	540	Non-specific lipid-transfer At5g64080	179	gi|118485342|gb|ABK94530.1|unknown [Populus trichocarpa]	ABK94530	3.48E-48	184	135
Pe186E19.16		+	5	1216	774	20 kda chloroplastic-like isoform X1 [Gossypium hirsutum]	257	gi|802628447|ref|XP_012077092.1|PREDICTED: 20 kDa chaperonin, chloroplastic [Jatropha curcas]gi|643724747|gb|KDP33948.1|hypothetical protein JCGZ_07519 [Jatropha curcas]	XP_012077092, KDP33948	1.36E-147	257	240
Pe186E19.17		-	3	833	543	Zinc finger zfs1-like	180	gi|720016204|ref|XP_010261083.1|PREDICTED: uncharacterized protein LOC104599994 [Nelumbo nucifera]gi|720016208|ref|XP_010261084.1|PREDICTED: uncharacterized protein LOC104599994 [Nelumbo nucifera]	XP_010261083, XP_010261084	2.77E-05	127	57
Pe186E19.18		-	11	3181	1794	Sulfate transporter -like [Populus euphratica]	597	gi|1000937733|ref|XP_015583853.1|PREDICTED: sulfate transporter 1.3 [Ricinus communis]	XP_015583853	0.0	628	529
Pe186E19.19		-	3	2542	516	Chloroplast-targeted copper chaperone [Theobroma cacao]	171	gi|731410542|ref|XP_010657601.1|PREDICTED: uncharacterized protein LOC100243595 [Vitis vinifera]	XP_010657601	1.02E-60	174	130
Pe186E19.20		+	1	1434	1434	F-box kelch-repeat At1g22040-like [Gossypium hirsutum]	477	gi|823160236|ref|XP_012479960.1|PREDICTED: F-box/kelch-repeat protein At1g22040-like [Gossypium raimondii]gi|823160238|ref|XP_012479961.1|PREDICTED: F-box/kelch-repeat protein At1g22040-like [Gossypium raimondii]gi|823160240|ref|XP_012479962.1|PREDICTED: F-box/kelch-repeat protein	XP_012479960, XP_012479961, XP_012479962, XP_012479963, KJB32005, KJB32006, KJB32007	0.0	478	382

							At1g22040-like [Gossypium raimondii]gi|823160242|ref|XP_012479963.1|PREDICTED: F-box/kelch-repeat protein At1g22040-like [Gossypium raimondii]gi|763764751|gb|KJB32005.1|hypothetical protein B456_005G218400 [Gossypium raimondii]gi|763764752|gb|KJB32006.1|hypothetical protein B456_005G218400 [Gossypium raimondii]gi|763764753|gb|KJB32007.1|hypothetical protein B456_005G218400 [Gossypium raimondii]				
Pe186E19.21	-	10	2851	1248	Probable LL-diaminopimelate chloroplastic [Populus euphratica]	415	gi|743899848|ref|XP_011043216.1|PREDICTED: probable LL-diaminopimelate aminotransferase, chloroplastic [Populus euphratica]gi|743899850|ref|XP_011043217.1|PREDICTED: probable LL-diaminopimelate aminotransferase, chloroplastic [Populus euphratica]gi|743899852|ref|XP_011043218.1|PREDICTED: probable LL-diaminopimelate aminotransferase, chloroplastic [Populus euphratica]gi|743899854|ref|XP_011043219.1|PREDICTED: probable LL-diaminopimelate aminotransferase, chloroplastic [Populus euphratica]gi|743899856|ref|XP_011043220.1|PREDICTED: probable LL-diaminopimelate aminotransferase, chloroplastic [Populus euphratica]	XP_011043216, XP_011043217, XP_011043218, XP_011043219, XP_011043220	0.0	414	361
Pe186E19.22	-	11	5100	1827	BRASSINOSTEROID INSENSITIVE 1-associated receptor kinase 1-like	611	gi|743938672|ref|XP_011013774.1|PREDICTED: BRASSINOSTEROID INSENSITIVE 1-associated receptor kinase 1-like [Populus euphratica]	XP_011013774	0.0	601	563
Pe186E19.23-24-25-26	-	9	3502	2307	Beta-glucosidase family [Populus trichocarpa]/beta-xylosidase alpha-L-arabinofuranosidase 1-like	154	gi|641837580|gb|KDO56532.1|hypothetical protein CISIN_1g0476211mg, partial [Citrus sinensis]	KDO56532	9.35E-58	121	110
Pe186E19.27	+	1	4219	1935	Hornerin-like [Pyrus x bretschneideri]	644	gi|566160321|ref|XP_002304079.2|hypothetical protein POPTR_0003s01750g [Populus trichocarpa]gi|550342152|gb|EEE79058.2|hypothetical protein POPTR_0003s01750g [Populus trichocarpa]	XP_002304079, EEE79058	0.0	649	539
Pe186E19.28	+	3	2327	900	TIS11-like [Gossypium raimondii]	299	gi|743911037|ref|XP_010999374.1|PREDICTED: uncharacterized protein LOC105107224 [Populus euphratica]gi|743911039|ref|XP_010999375.1|PREDICTED: uncharacterized protein LOC105107224 [Populus euphratica]gi|743911041|ref|XP_010999376.1|PREDICTED: uncharacterized protein LOC105107224 [Populus euphratica]gi|743911043|ref|XP_010999377.1|PREDICTED: uncharacterized protein LOC105107224 [Populus euphratica]	XP_010999374, XP_010999375, XP_010999376, XP_010999377	6.23E-51	291	174
Pe186E19.29	+	4	1831	372	PREDICTED: uncharacterized protein LOC105639838 [Jatropha curcas]	123	gi|802645882|ref|XP_012079394.1|PREDICTED: uncharacterized protein LOC105639838 [Jatropha curcas]gi|643722186|gb|KDP32065.1|hypothetical protein JCGZ_12526 [Jatropha curcas]	XP_012079394, KDP32065	1.49E-33	104	81

Pe186E19.30		+	2	1216	210	---Na---	69	No Blast Hit				
Pe186E19.31	1 isoform	+	3	1949	570	NAC domain IPR003441 isoform 1 [Theobroma cacao]	189	gi|802536203|ref|XP_012090474.1|PREDICTED: NAC transcription factor NAM-B2 [Jatropha curcas]gi|495025487|gb|AGL39676.1|NAC transcription factor 020 [Jatropha curcas]gi|643741804|gb|KDP47166.1|hypothetical protein JCGZ_00057 [Jatropha curcas]	XP_012090474, AGL39676, KDP47166	9.61E-96	190	164
Pe186E19.32		-	9	3930	801	Phosphatidylserine decarboxylase proenzyme 2-like	266	gi|596121642|ref|XP_007221805.1|hypothetical protein PRUPE_ppa006115mg [Prunus persica]gi|462418741|gb|EMJ23004.1|hypothetical protein PRUPE_ppa006115mg [Prunus persica]	XP_007221805, EMJ23004	2.78E-151	266	240
Pe195F4.1		+	1	681	177	[Physcomitrella patens]	58	gi|590684475|ref|XP_007041864.1|20S proteasome alpha subunit PAD1 [Theobroma cacao]gi|508705799|gb|EOX97695.1|20S proteasome alpha subunit PAD1 [Theobroma cacao]	XP_007041864, EOX97695	4.93E-27	58	58
Pe195F4.2		-	2	5781	759	Myb-related 308-like	252	gi|449458606|ref|XP_004147038.1|PREDICTED: myb-related protein 308-like [Cucumis sativus]	XP_004147038	4.73E-130	259	222
Pe195F4.3		-	12	3762	1620	Synaptotagmin A isoform 1 [Theobroma cacao]	539	gi|743793114|ref|XP_011046292.1|PREDICTED: synaptotagmin-2 [Populus euphratica]	XP_011046292	0.0	539	506
Pe195F4.4		+	20	8364	5442	E3 ubiquitin- ligase UPL3-like	1813	gi|590684421|ref|XP_007041850.1|HEAT repeat,HECT-domain isoform 1 [Theobroma cacao]gi|508705785|gb|EOX97681.1|HEAT repeat,HECT-domain isoform 1 [Theobroma cacao]	XP_007041850, EOX97681	0.0	1748	1501
Pe195F4.5		-	14	3925	1935	Beta-galactosidase 13-like	644	gi|224080622|ref|XP_002306183.1|glycosyl hydrolase family 35 family protein [Populus trichocarpa]gi|222849147|gb|EEE86694.1|glycosyl hydrolase family 35 family protein [Populus trichocarpa]	XP_002306183, EEE86694	0.0	676	525
Pe195F4.6		-	1	261	261	---Na---	86	No Blast Hit				
Pe195F4.7		+	19	4316	2505	Beta-galactosidase 13-like	834	gi|224080622|ref|XP_002306183.1|glycosyl hydrolase family 35 family protein [Populus trichocarpa]gi|222849147|gb|EEE86694.1|glycosyl hydrolase family 35 family protein [Populus trichocarpa]	XP_002306183, EEE86694	0.0	810	670
Pe195F4.8	2 isoforms	+	11	3020	1230	MCM10 homolog	409	gi|566187848|ref|XP_002313710.2|hypothetical protein POPTR_0009s13690g [Populus trichocarpa]gi|550331664|gb|EEE87665.2|hypothetical protein POPTR_0009s13690g [Populus trichocarpa]	XP_002313710, EEE87665	0.0	415	342
Pe195F4.9		+	7	4959	597	Rac-like GTP-binding 5	198	gi|225428023|ref|XP_002278595.1|PREDICTED: rac-like GTP-binding protein 5 [Vitis vinifera]gi|147768427|emb|CAN73627.1|hypothetical protein VITISV_026639 [Vitis vinifera]gi|297744613|emb|CBI37875.3|unnamed protein product [Vitis vinifera]	XP_002278595, CAN73627, CBI37875	1.62E-138	198	197
Pe195F4.10	1 isoform	+	4	1229	447	Ubiquitin-conjugating enzyme E2 28-like	148	gi|645260849|ref|XP_008236012.1|PREDICTED: ubiquitin-conjugating enzyme E2 28-like [Prunus mume]	XP_008236012	1.29E-100	148	148
Pe195F4.11		+	10	3705	2043	Kinase with adenine nucleotide alpha hydrolases-like isoform 1 [Theobroma cacao]	680	gi|224078315|ref|XP_002305520.1|kinase family protein [Populus trichocarpa]gi|222848484|gb|EEE86031.1|kinase family protein [Populus trichocarpa]	XP_002305520, EEE86031	0.0	683	575
Pe195F4.12	2 isoforms	+	2	2704	834	Basic-leucine zipper transcription factor family	277	gi|1000937742|ref|XP_015583847.1|PREDICTED: uncharacterized protein LOC8266428 isoform	XP_015583847	2.40E-146	277	234

					isoform 1 [Theobroma cacao]		X2 [Ricinus communis]				
Pe195F4.13	-	6	2653	1203	WD-40 repeat-containing MSI2-like	400	gi|743793054|ref|XP_011046141.1|PREDICTED : WD-40 repeat-containing protein MSI2-like [Populus euphratica]	XP_011046141	0.0	400	353
Pe195F4.14	-	2	1904	465	Heavy metal-associated isoprenylated plant 26-like	154	gi|224080626|ref|XP_002306185.1|GMFP7 family protein [Populus trichocarpa]gi|222849149|gb|EEE86696.1|GMFP 7 family protein [Populus trichocarpa]	XP_002306185, EEE86696	3.69E-92	154	146
Pe195F4.15	+	2	1395	858	Probable BOI-related E3 ubiquitin- ligase 2	285	gi|743906511|ref|XP_011046677.1|PREDICTED : probable BOI-related E3 ubiquitin-protein ligase 2 [Populus euphratica]	XP_011046677	1.93E-100	289	219
Pe195F4.16	+	9	4234	687	Probable CDP-diacylglycerol--inositol 3-phosphatidyltransferase 2 [Jatropha curcas]	228	gi|802674798|ref|XP_012081784.1|PREDICTED : probable CDP-diacylglycerol--inositol 3-phosphatidyltransferase 2 [Jatropha curcas]gi|802674811|ref|XP_012081785.1|PRED ICTED: probable CDP-diacylglycerol--inositol 3-phosphatidyltransferase 2 [Jatropha curcas]gi|802674820|ref|XP_012081786.1|PRED ICTED: probable CDP-diacylglycerol--inositol 3-phosphatidyltransferase 2 [Jatropha curcas]gi|802674827|ref|XP_012081787.1|PRED ICTED: probable CDP-diacylglycerol--inositol 3-phosphatidyltransferase 2 [Jatropha curcas]gi|802674830|ref|XP_012081788.1|PRED ICTED: probable CDP-diacylglycerol--inositol 3-phosphatidyltransferase 2 [Jatropha curcas]gi|802674838|ref|XP_012081790.1|PRED ICTED: probable CDP-diacylglycerol--inositol 3-phosphatidyltransferase 2 [Jatropha curcas]gi|802674848|ref|XP_012081791.1|PRED ICTED: probable CDP-diacylglycerol--inositol 3-phosphatidyltransferase 2 [Jatropha curcas]gi|643718452|gb|KDP29667.1|hypothetica l protein JCGZ_18829 [Jatropha curcas]	XP_012081784, XP_012081785, XP_012081786, XP_012081787, XP_012081788, XP_012081790, XP_012081791, KDP29667	3.60E-134	228	207
Pe195F4.17	-	2	1842	1134	High-affinity nickel-transport family [Theobroma cacao]	377	gi|802674857|ref|XP_012081792.1|PREDICTED : uncharacterized protein LOC105641795 [Jatropha curcas]	XP_012081792	0.0	378	326
Pe195F4.18	+	13	2378	1161	Glycerate dehydrogenase	386	gi|595792351|ref|XP_007199924.1|hypothetical protein PRUPE_ppa006993mg [Prunus persica]gi|462395324|gb|EMJ01123.1|hypothetic al protein PRUPE_ppa006993mg [Prunus persica]	XP_007199924, EMJ01123	0.0	386	379
Pe195F4.19	+	2	544	465	SCF ubiquitin SKP1 component [Medicago truncatula]	154	gi|1012122743|ref|XP_015962809.1|PREDICTE D: SKP1-like protein 1B [Arachis duranensis]	XP_015962809	4.99E-51	155	108
Pe195F4.20	+	13	3984	1254	Heparan-alpha-glucosaminide N-acetyltransferase-like	417	gi|1009150695|ref|XP_015893157.1|PREDICTE D: heparan-alpha-glucosaminide N-acetyltransferase-like [Ziziphus jujuba]	XP_015893157	0.0	423	360
Pe195F4.21	+	4	1350	837	Plasma membrane intrinsic 2,8 isoform 1 [Theobroma cacao]	278	gi|590684272|ref|XP_007041804.1|Plasma membrane intrinsic protein 2,8 isoform 1 [Theobroma cacao]gi|508705739|gb|EOX97635.1|Plasma membrane intrinsic protein 2,8 isoform 1 [Theobroma cacao]	XP_007041804, EOX97635	0.0	278	266
Pe195F4.22	-	3	1618	1215	Phospholipase isoform 2 [Theobroma cacao]	404	gi|743906501|ref|XP_011046671.1|PREDICTED : uncharacterized protein LOC105141207 isoform X3 [Populus euphratica]	XP_011046671	1.10E-147	408	300

Pe195F4.23		-	2	794	306	PREDICTED: uncharacterized protein LOC105141207 isoform X3	101	gi|743906501|ref|XP_011046671.1|PREDICTED : uncharacterized protein LOC105141207 isoform X3 [Populus euphratica]	XP_011046671	3.02E-19	71	55
Pe198H23.1		+	1	503	503	PREDICTED: extensin-2 [Ricinus communis]	166	gi|1000974909|ref|XP_015572337.1|PREDICTE D: extensin-2 [Ricinus communis]	XP_015572337	6.60E-21	162	105
Pe198H23.2		+	2	1159	441	Mitochondrial carrier family [Populus trichocarpa]	146	gi|1009148412|ref|XP_015891923.1|PREDICTE D: uncharacterized protein LOC107426304 [Ziziphus jujuba]	XP_015891923	7.38E-42	138	101
Pe198H23.3		-	1	722	471	---Na---	156	No Blast Hit				
Pe198H23.4		+	1	2107	1098	Probable galacturonosyltransferase-like 7	365	gi|802689173|ref|XP_012082838.1|PREDICTED : probable galacturonosyltransferase-like 7 [Jatropha curcas]gi|802689180|ref|XP_012082840.1|PRED ICTED: probable galacturonosyltransferase-like 7 [Jatropha curcas]gi|802689191|ref|XP_012082841.1|PRED ICTED: probable galacturonosyltransferase-like 7 [Jatropha curcas]gi|802689200|ref|XP_012082842.1|PRED ICTED: probable galacturonosyltransferase-like 7 [Jatropha curcas]	XP_012082838, XP_012082840, XP_012082841, XP_012082842	0.0	362	318
Pe198H23.5		-	3	4579	3510	Cellulose synthase D5	1169	gi|590719376|ref|XP_007051035.1|Cellulose synthase-like D5 [Theobroma cacao]gi|508703296|gb|EOX95192.1|Cellulose synthase-like D5 [Theobroma cacao]	XP_007051035, EOX95192	0.0	1184	1092
Pe198H23.6		-	6	2091	1413	Two-component response regulator ARR1-like isoform X2	470	gi|255564288|ref|XP_002523141.1|PREDICTED : two-component response regulator ARR2 [Ricinus communis]gi|223537703|gb|EEF39326.1|two-component sensor histidine kinase bacteria, putative [Ricinus communis]	XP_002523141, EEF39326	5.11E-76	442	257
Pe198H23.7		+	5	3552	750	Drought-responsive family [Populus trichocarpa]	220	gi|1000957222|ref|XP_015577201.1|PREDICTE D: protein DEHYDRATION-INDUCED 19 homolog 6 [Ricinus communis]	XP_015577201	2.37E-56	229	147
Pe198H23.8		-	6	5279	1215	Hypothetical protein L484_007474 [Morus notabilis]	404	gi|703125832|ref|XP_010103405.1|hypothetical protein L484_007474 [Morus notabilis]gi|587907734|gb|EXB95724.1|hypotheti cal protein L484_007474 [Morus notabilis]	XP_010103405, EXB95724	0.0	416	335
Pe198H23.9		-	6	3202	1428	Probable polygalacturonase isoform X2	475	gi|731395561|ref|XP_010652214.1|PREDICTED : probable polygalacturonase [Vitis vinifera]	XP_010652214	0.0	413	355
Pe198H23.10	2 isoforms	+	5	4267	1914	Ethylene receptor family [Populus trichocarpa]	637	gi|4164161|dbj|BAA37137.1|ethylene response sensor [Passiflora edulis]	BAA37137	0.0	637	634
Pe198H23.11		-	2	1504	861	Ethylene-responsive transcription factor RAP2-12-like isoform X2	286	gi|802689061|ref|XP_012082821.1|PREDICTED : ethylene-responsive transcription factor ERF073 [Jatropha curcas]gi|643716573|gb|KDP28199.1|hypothetica l protein JCGZ_13970 [Jatropha curcas]	XP_012082821, KDP28199	1.24E-91	316	210
Pe198H23.12		-	1	866	705	---Na---	234	No Blast Hit				
Pe198H23.13		-	5	1698	1245	Exopolygalacturonase clone GBGE184	414	gi|224064464|ref|XP_002301489.1|Exopolygalac turonase clone GBGE184 precursor family protein [Populus trichocarpa]gi|222843215|gb|EEE80762.1|Exopol ygalacturonase clone GBGE184 precursor family protein [Populus trichocarpa]	XP_002301489, EEE80762	0.0	409	333

Pe198H23.14	-	3	2136	1686	Pectinesterase-like	561	gi|566159086|ref|XP_002301486.2|hypothetical protein POPTR_0002s20370g [Populus trichocarpa]gi|550345454|gb|EEE80759.2|hypothetical protein POPTR_0002s20370g [Populus trichocarpa]	XP_002301486, EEE80759	0.0	547	477
Pe198H23.15	+	2	1813	1686	Probable pectinesterase inhibitor 7	561	gi|224128446|ref|XP_002320332.1|pectinesterase family protein [Populus trichocarpa]gi|222861105|gb|EEE98647.1|pectinesterase family protein [Populus trichocarpa]	XP_002320332, EEE98647	0.0	561	472
Pe198H23.16	-	1	540	540	Pectinesterase inhibitor-like	179	gi|743885806|ref|XP_011037660.1|PREDICTED: pectinesterase inhibitor-like [Populus euphratica]	XP_011037660	3.92E-42	183	120
Pe198H23.17	-	1	851	645	RING-H2 finger ATL2	214	gi|802688990|ref|XP_012082808.1|PREDICTED: RING-H2 finger protein ATL5-like [Jatropha curcas]gi|643716561|gb|KDP28187.1|hypothetical protein JCGZ_13958 [Jatropha curcas]	XP_012082808, KDP28187	3.94E-37	196	123
Pe198H23.18	+	3	1857	948	Bifunctional epoxide hydrolase 2-like	454	gi|567903814|ref|XP_006444395.1|hypothetical protein CICLE_v10024303mg, partial [Citrus clementina]gi|557546657|gb|ESR57635.1|hypothetical protein CICLE_v10024303mg, partial [Citrus clementina]	XP_006444395, ESR57635	2.86E-121	261	212
Pe198H23.19	+	2	1602	1497	Gag protease poly [Theobroma cacao]	498	gi|590728434|ref|XP_007099662.1|Gag protease polyprotein-like protein [Theobroma cacao]gi|508728474|gb|EOY20371.1|Gag protease polyprotein-like protein [Theobroma cacao]	XP_007099662, EOY20371	2.96E-67	473	237
Pe198H23.20	+	3	1512	672	---Na---	249	No Blast Hit				
Pe198H23.21	-	1	180	180	---Na---	59	No Blast Hit				
Pe198H23.22	+	1	684	684	---Na---	227	No Blast Hit				
Pe198H23.23	+	2	548	348	---Na---	115	No Blast Hit				
Pe198H23.24	+	4	3399	3003	Unnamed protein product	1000	gi|87116463|dbj|BAE79384.1|unnamed protein product [Ipomoea batatas]	BAE79384	1.51E-63	792	339
Pe201C11.1	-	5	2820	711	Transcription factor ILR3-like	236	gi|502143701|ref|XP_004505433.1|PREDICTED: transcription factor ILR3-like [Cicer arietinum]	XP_004505433	1.11E-120	242	208
Pe201C11.2	-	3	1443	384	Thioredoxin h [Vitis vinifera]	127	gi|1009110861|ref|XP_015897996.1|PREDICTED: thioredoxin-like protein CXXS1 [Ziziphus jujuba]	XP_015897996	3.21E-56	127	112
Pe201C11.3	-	17	5434	2400	Kinesin-3 isoform X2	799	gi|802587202|ref|XP_012070721.1|PREDICTED: kinesin-3 isoform X1 [Jatropha curcas]gi|643731848|gb|KDP39040.1|hypothetical protein JCGZ_00797 [Jatropha curcas]	XP_012070721, KDP39040	0.0	802	721
Pe201C11.4	+	1	1485	408	60S ribosomal L27-3-like [Gossypium raimondii]	135	gi|802774424|ref|XP_012090796.1|PREDICTED: 60S ribosomal protein L27-3 [Jatropha curcas]gi|643705874|gb|KDP22049.1|hypothetical protein JCGZ_02471 [Jatropha curcas]	XP_012090796, KDP22049	9.61E-83	135	133
Pe201C11.5	-	1	318	318	---Na---	105	No Blast Hit				
Pe201C11.6	+	4	2977	618	Hypothetical protein B456_006G213400	205	gi|763770414|gb|KJB37629.1|hypothetical protein B456_006G213400 [Gossypium raimondii]	KJB37629	6.03E-15	108	63
Pe201C11.7	-	3	1210	912	Nuclease HARBI1	303	gi|702274810|ref|XP_010044171.1|PREDICTED: putative nuclease HARBI1 isoform X1 [Eucalyptus grandis]	XP_010044171	2.17E-104	292	204
Pe201C11.8	-	1	195	195	---Na---	64	No Blast Hit				
Pe201C11.9	-	1	720	720	---Na---	239	No Blast Hit				

Pe201C11.10		-	1	438	438	---Na---	145	No Blast Hit				
Pe201C11.11		+	5	1847	1338	Ribonuclease h at1g65750	445	gi|4544460|gb|AAD22368.1|putative non-LTR retroelement reverse transcriptase [Arabidopsis thaliana]	AAD22368	4.48E-33	277	135
Pe201C11.12		+	1	1510	234	Mitochondrial import receptor subunit TOM7 family [Populus trichocarpa]	77	gi|566215807|ref|XP_006372198.1|Mitochondrial import receptor subunit TOM7 family protein [Populus trichocarpa]gi|550318729|gb|ERP49995.1|Mitochondrial import receptor subunit TOM7 family protein [Populus trichocarpa]	XP_006372198, ERP49995	2.15E-27	76	65
Pe201C11.13-14		+	9	5199	2634	Formin 5	525	gi|1000957728|ref|XP_015577081.1|PREDICTED: formin-like protein 5 isoform X2 [Ricinus communis]	XP_015577081	4.24E-97	598	327
Pe201C11.15		+	11	5452	2298	CWF19 2 [Jatropha curcas]	765	gi|802540628|ref|XP_012077540.1|PREDICTED: CWF19-like protein 2 [Jatropha curcas]gi|802540630|ref|XP_012077547.1|PREDICTED: CWF19-like protein 2 [Jatropha curcas]	XP_012077540, XP_012077547	0.0	797	605
Pe201C11.16		-	2	2059	402	Histone H2A family [Populus trichocarpa]	133	gi|703083793|ref|XP_010092305.1|putative histone H2A.1 [Morus notabilis]gi|587861075|gb|EXB50939.1|putative histone H2A.1 [Morus notabilis]	XP_010092305, EXB50939	1.84E-81	132	131
Pe201C11.17		+	2	2730	1290	Zinc finger family [Populus trichocarpa]	429	gi|566154756|ref|XP_006370600.1|hypothetical protein POPTR_0001s44130g [Populus trichocarpa]gi|550349806|gb|ERP67169.1|hypothetical protein POPTR_0001s44130g [Populus trichocarpa]	XP_006370600, ERP67169	0.0	440	387
Pe201C11.18		-	1	573	573	Ribonuclease h at1g65750	190	gi|590647573|ref|XP_007031938.1|Ribonuclease H protein [Theobroma cacao]gi|508710967|gb|EOY02864.1|Ribonuclease H protein [Theobroma cacao]	XP_007031938, EOY02864	3.70E-17	160	81
Pe201C11.19		+	1	408	408	---Na---	135	No Blast Hit				
Pe201C11.20		+	1	606	606	---Na---	201	No Blast Hit				
Pe201C11.21		+	1	264	264	---Na---	87	No Blast Hit				
Pe201C11.22		+	4	2639	237	---Na---	78	No Blast Hit				
Pe201C11.23		+	1	708	708	---Na---	235	No Blast Hit				
Pe207D11.1	Incomplete 5'	+	2	510	399	Pentatricopeptide repeat-containing mitochondrial-like	132	gi|743909614|ref|XP_011048290.1|PREDICTED: pentatricopeptide repeat-containing protein At4g04790, mitochondrial-like [Populus euphratica]	XP_011048290	4.51E-52	131	106
Pe207D11.2		-	1	675	675	Potassium sodium hyperpolarization-activated cyclic nucleotide-gated channel 1 [Theobroma cacao]	224	gi|743944359|ref|XP_011016700.1|PREDICTED: uncharacterized protein LOC105120210 [Populus euphratica]	XP_011016700	5.15E-95	231	183
Pe207D11.3		+	13	3430	1119	Polycomb group FERTILIZATION-INDEPENDENT ENDOSPERM-like [Gossypium hirsutum]	372	gi|802587398|ref|XP_012070730.1|PREDICTED: polycomb group protein FERTILIZATION-INDEPENDENT ENDOSPERM [Jatropha curcas]gi|643731856|gb|KDP39048.1|hypothetical protein JCGZ_00805 [Jatropha curcas]	XP_012070730, KDP39048	0.0	371	360
Pe207D11.4	1 isoform	+	7	6756	2670	Autophagy-related 18f-like	889	gi|802587292|ref|XP_012070728.1|PREDICTED: autophagy-related protein 18f [Jatropha curcas]gi|643731854|gb|KDP39046.1|hypothetical protein JCGZ_00803 [Jatropha curcas]	XP_012070728, KDP39046	0.0	899	717
Pe207D11.5		+	3	5083	1218	Probable	405	gi|802587290|ref|XP_012070727.1|PREDICTED	XP_012070727,	0.0	392	382

					galacturonosyltransferase 12		: probable galacturonosyltransferase 12 [Jatropha curcas]gi|643731853|gb|KDP39045.1|hypothetical protein JCGZ_00802 [Jatropha curcas]	KDP39045			
Pe207D11.6	-	1	1014	1014	PREDICTED: uncharacterized protein LOC105780163	337	gi|823131826|ref|XP_012459787.1|PREDICTED: uncharacterized protein LOC105780163 [Gossypium raimondii]	XP_012459787	6.22E-126	338	258
Pe207D11.7	-	5	2806	711	Transcription factor ILR3-like	236	gi|502143701|ref|XP_004505433.1|PREDICTED: transcription factor ILR3-like [Cicer arietinum]	XP_004505433	1.11E-120	242	208
Pe207D11.8	-	3	1461	384	Thioredoxin h [Vitis vinifera]	127	gi|1009110861|ref|XP_015897996.1|PREDICTED: thioredoxin-like protein CXXS1 [Ziziphus jujuba]	XP_015897996	3.21E-56	127	112
Pe207D11.9	-	17	5435	2400	Kinesin-3 isoform X2	799	gi|802587202|ref|XP_012070721.1|PREDICTED: kinesin-3 isoform X1 [Jatropha curcas]gi|643731848|gb|KDP39040.1|hypothetical protein JCGZ_00797 [Jatropha curcas]	XP_012070721, KDP39040	0.0	802	721
Pe207D11.10	+	1	1491	408	60S ribosomal L27-3-like [Gossypium raimondii]	135	gi|802774424|ref|XP_012090796.1|PREDICTED: 60S ribosomal protein L27-3 [Jatropha curcas]gi|643705874|gb|KDP22049.1|hypothetical protein JCGZ_02471 [Jatropha curcas]	XP_012090796, KDP22049	9.61E-83	135	133
Pe207D11.11	-	1	270	270	---Na---	89	No Blast Hit				
Pe207D11.12	+	3	3271	1062	PREDICTED: uncharacterized protein At2g29880-like isoform X1 [Gossypium hirsutum]	353	gi|1029056112|ref|XP_016668589.1|PREDICTED: uncharacterized protein At2g29880-like isoform X1 [Gossypium hirsutum]gi|1029056114|ref|XP_016668595.1|PREDICTED: uncharacterized protein At2g29880-like isoform X1 [Gossypium hirsutum]gi|1029056116|ref|XP_016668602.1|PREDICTED: uncharacterized protein At2g29880-like isoform X1 [Gossypium hirsutum]gi|1029056118|ref|XP_016668607.1|PREDICTED: uncharacterized protein At2g29880-like isoform X1 [Gossypium hirsutum]gi|1029056120|ref|XP_016668613.1|PREDICTED: uncharacterized protein At2g29880-like isoform X1 [Gossypium hirsutum]gi|1029056122|ref|XP_016668622.1|PREDICTED: uncharacterized protein At2g29880-like isoform X1 [Gossypium hirsutum]	XP_016668589, XP_016668595, XP_016668602, XP_016668607, XP_016668613, XP_016668622	5.06E-43	308	162
Pe207D11.13	+	1	297	297	---Na---	98	No Blast Hit				
Pe207D11.14	+	4	1405	1134	Gag protease poly [Theobroma cacao]	377	gi|590689992|ref|XP_007043384.1|Gag protease polyprotein [Theobroma cacao]gi|508707319|gb|EOX99215.1|Gag protease polyprotein [Theobroma cacao]	XP_007043384, EOX99215	1.52E-23	215	113
Pe207D11.15	-	1	420	420	---Na---	139	No Blast Hit				
Pe207D11.16	-	3	1818	1230	Nuclease HARBI1	409	gi|702274810|ref|XP_010044171.1|PREDICTED: putative nuclease HARBI1 isoform X1 [Eucalyptus grandis]	XP_010044171	2.94E-129	342	249
Pe207D11.17	+	1	1026	306	---Na---	101	No Blast Hit				
Pe207D11.18	+	1	213	213	---Na---	70	No Blast Hit				
Pe207D11.19	-	1	276	276	---Na---	91	No Blast Hit				
Pe207D11.20	-	1	420	420	---Na---	139	No Blast Hit				
Pe207D11.21	+	1	339	339	---Na---	112	No Blast Hit				

Pe207D11.22		+	5	1844	1398	Ribonuclease h at1g65750	465	gi|4544460|gb|AAD22368.1|putative non-LTR retroelement reverse transcriptase [Arabidopsis thaliana]	AAD22368	7.17E-35	275	135
Pe207D11.23		+	1	234	234	Mitochondrial import receptor subunit TOM7 family [Populus trichocarpa]	77	gi|566215807|ref|XP_006372198.1|Mitochondrial import receptor subunit TOM7 family protein [Populus trichocarpa]gi|550318729|gb|ERP49995.1|Mitochondrial import receptor subunit TOM7 family protein [Populus trichocarpa]	XP_006372198, ERP49995	2.15E-27	76	65
Pe207D11.24		+	7	5200	2730	Formin 5 isoform 1 [Theobroma cacao]	909	gi|255563641|ref|XP_002522822.1|PREDICTED: formin-like protein 5 isoform X1 [Ricinus communis]gi|223537906|gb|EEF39520.1|actin binding protein, putative [Ricinus communis]	XP_002522822, EEF39520	0.0	981	671
Pe207D11.25	Incomplete3'	+	6	1709	1099	Cwf19 2	366	gi|567854079|ref|XP_006420159.1|hypothetical protein CICLE_v10004361mg [Citrus clementina]gi|557522032|gb|ESR33399.1|hypothetical protein CICLE_v10004361mg [Citrus clementina]	XP_006420159, ESR33399	6.84E-96	373	257
Pe209G15.1		-	9	4652	3084	C2 and GRAM domain-containing At1g03370-like	1027	gi|1000953987|ref|XP_015578356.1|PREDICTED: C2 and GRAM domain-containing protein At1g03370 [Ricinus communis]	XP_015578356	0.0	1028	898
Pe209G15.2		-	9	3919	2751	Linoleate 13S-lipoxygenase 2-chloroplastic-like	916	gi|566185986|ref|XP_002314229.2|hypothetical protein POPTR_0009s02750g [Populus trichocarpa]gi|550330904|gb|EEE88184.2|hypothetical protein POPTR_0009s02750g [Populus trichocarpa]	XP_002314229, EEE88184	0.0	918	723
Pe209G15.3		-	7	3447	2238	Oligopeptide transporter 1-like	745	gi|1021025474|gb|KZM83262.1|hypothetical protein DCAR_030831 [Daucus carota subsp. sativus]	KZM83262	0.0	742	666
Pe209G15.4	2 isoforms	+	2	5039	1833	Serine threonine- kinase D6PKL2-like [Populus euphratica]	610	gi|566207955|ref|XP_002322497.2|hypothetical protein POPTR_0016s00660g [Populus trichocarpa]gi|550320499|gb|EEF04258.2|hypothetical protein POPTR_0016s00660g [Populus trichocarpa]	XP_002322497, EEF04258	0.0	613	553
Pe209G15.5		-	4	1434	915	U-box domain-containing 35-like	304	gi|224089553|ref|XP_002308753.1|hypothetical protein POPTR_0006s00580g [Populus trichocarpa]gi|222854729|gb|EEE92276.1|hypothetical protein POPTR_0006s00580g [Populus trichocarpa]	XP_002308753, EEE92276	5.23E-106	295	217
Pe209G15.6		-	2	1853	1671	WEB family At1g12150-like	556	gi|224139444|ref|XP_002323115.1|hypothetical protein POPTR_0016s00690g [Populus trichocarpa]gi|222867745|gb|EEF04876.1|hypothetical protein POPTR_0016s00690g [Populus trichocarpa]	XP_002323115, EEF04876	1.38E-169	541	409
Pe209G15.7	2 isoforms	-	14	8353	1242	Thiolase family isoform 1 [Theobroma cacao]	413	gi|1000957655|ref|XP_015577103.1|PREDICTED: acetyl-CoA acetyltransferase, cytosolic 1 isoform X1 [Ricinus communis]	XP_015577103	0.0	410	379
Pe209G15.8		-	2	1542	840	2OG-Fe(II) oxygenase family oxidoreductase [Medicago truncatula]	279	gi|743916755|ref|XP_011002353.1|PREDICTED: alpha-ketoglutarate-dependent dioxygenase alkB homolog 2 [Populus euphratica]	XP_011002353	5.75E-102	255	188
Pe209G15.9		-	1	650	606	Nuclear transcription factor Y subunit B-6	201	gi|359497402|ref|XP_003635503.1|PREDICTED: nuclear transcription factor Y subunit B-6 [Vitis vinifera]gi|296083539|emb|CBI23532.3|unnamed protein product [Vitis vinifera]	XP_003635503, CBI23532	1.95E-87	189	160
Pe209G15.10		+	4	4477	912	SNF1-related kinase regulatory subunit beta-2-like	303	gi|566207979|ref|XP_002322502.2|hypothetical protein POPTR_0016s00810g [Populus trichocarpa]gi|550320509|gb|EEF04263.2|hypothetical protein POPTR_0016s00810g [Populus	XP_002322502, EEF04263	1.11E-161	303	261

								trichocarpa]				
Pe209G15.11		-	6	3911	2220	Oligopeptide transporter 3	739	gi|802614695|ref|XP_012074788.1|PREDICTED: oligopeptide transporter 3 [Jatropha curcas]gi|643726940|gb|KDP35505.1|hypothetical protein JCGZ_08943 [Jatropha curcas]	XP_012074788, KDP35505	0.0	737	699
Pe209G15.12		-	5	2570	960	Copper-binding family [Populus trichocarpa]	319	gi|743916795|ref|XP_011002375.1|PREDICTED: heavy metal-associated isoprenylated plant protein 26-like [Populus euphratica]	XP_011002375	3.95E-111	238	214
Pe209G15.13		+	1	219	219	---Na---	72	No Blast Hit				
Pe209G15.14		+	2	415	342	---Na---	113	No Blast Hit				
Pe209G15.15		-	3	615	453	Beta-glucosidase 46-like isoform X1	150	gi|1021508176|ref|XP_016197440.1|PREDICTED: beta-glucosidase 46 isoform X2 [Arachis ipaensis]	XP_016197440	2.96E-36	136	90
Pe209G15.16		+	1	2115	2115	Pentatricopeptide repeat-containing chloroplastic	704	gi|566207985|ref|XP_002322504.2|chloroplastic RNA-binding protein P67 [Populus trichocarpa]gi|550320511|gb|EEF04265.2|chloroplastic RNA-binding protein P67 [Populus trichocarpa]	XP_002322504, EEF04265	0.0	705	560
Pe209G15.17		-	10	7638	1674	Microtubule-associated 70-5	557	gi|657958883|ref|XP_008370982.1|PREDICTED: microtubule-associated protein 70-5 [Malus domestica]	XP_008370982	0.0	561	448
Pe212D7.1		+	1	309	309	Gag protease poly [Theobroma cacao]	102	gi|590581221|ref|XP_007014288.1|Uncharacterized protein TCM_039264 [Theobroma cacao]gi|508784651|gb|EOY31907.1|Uncharacterized protein TCM_039264 [Theobroma cacao]	XP_007014288, EOY31907	6.44E-25	99	69
Pe212D7.2		-	1	288	288	---Na---	95	No Blast Hit				
Pe212D7.3		-	2	568	408	---Na---	135	No Blast Hit				
Pe212D7.4		-	2	973	714	Nuclease HARBI1 isoform X1	237	gi|985448414|ref|XP_015385689.1|PREDICTED: uncharacterized protein LOC107177004 [Citrus sinensis]	XP_015385689	2.59E-31	125	82
Pe212D7.5		-	2	721	372	---Na---	123	No Blast Hit				
Pe212D7.6		-	1	171	171	---Na---	56	No Blast Hit				
Pe212D7.7		-	1	321	321	---Na---	106	No Blast Hit				
Pe212D7.8		+	3	1835	180	---Na---	59	No Blast Hit				
Pe212D7.9	1 isoform	+	12	5554	1977	G2484-1 isoform 1 [Theobroma cacao]	658	gi|255562773|ref|XP_002522392.1|PREDICTED: uncharacterized protein LOC8260588 isoform X1 [Ricinus communis]gi|1000958683|ref|XP_015576768.1|PREDICTED: uncharacterized protein LOC8260588 isoform X1 [Ricinus communis]gi|1000958685|ref|XP_015576769.1|PREDICTED: uncharacterized protein LOC8260588 isoform X1 [Ricinus communis]gi|1000958687|ref|XP_015576770.1|PREDICTED: uncharacterized protein LOC8260588 isoform X1 [Ricinus communis]gi|1000958689|ref|XP_015576771.1|PREDICTED: uncharacterized protein LOC8260588 isoform X1 [Ricinus communis]gi|1000958691|ref|XP_015576772.1|PREDICTED: uncharacterized protein LOC8260588 isoform X1 [Ricinus	XP_002522392, XP_015576768, XP_015576769, XP_015576770, XP_015576771, XP_015576772, XP_015576773, EEF40076	0.0	659	530

								communis]gi|1000958693|ref|XP_015576773.1|PREDICTED: uncharacterized protein LOC8260588 isoform X1 [Ricinus communis]gi|223538470|gb|EEF40076.1|electron transporter, putative [Ricinus communis]				
Pe212D7.10		-	1	935	396	PREDICTED: uncharacterized protein LOC105803851 [Gossypium raimondii]	131	gi|823192296|ref|XP_012491711.1|PREDICTED: uncharacterized protein LOC105803851 [Gossypium raimondii]gi|763775306|gb|KJB42429.1|hypothetical protein B456_007G207600 [Gossypium raimondii]	XP_012491711, KJB42429	9.81E-36	133	89
Pe212D7.11		-	1	1082	471	HTH-type transcriptional regulator ptxe [Theobroma cacao]	156	gi|802641037|ref|XP_012079119.1|PREDICTED: uncharacterized protein LOC105639617 [Jatropha curcas]gi|643721951|gb|KDP31830.1|hypothetical protein JCGZ_12291 [Jatropha curcas]	XP_012079119, KDP31830	4.37E-69	167	136
Pe212D7.12		-	2	1377	999	Oxygen-evolving enhancer chloroplastic	332	gi|1009145009|ref|XP_015890101.1|PREDICTED: oxygen-evolving enhancer protein 1, chloroplastic [Ziziphus jujuba]	XP_015890101	0.0	332	313
Pe212D7.13		-	4	2951	612	Cold-regulated 413 plasma membrane 2	203	gi|802641055|ref|XP_012079124.1|PREDICTED: cold-regulated 413 plasma membrane protein 2-like [Jatropha curcas]gi|802641060|ref|XP_012079125.1|PREDICTED: cold-regulated 413 plasma membrane protein 2-like [Jatropha curcas]gi|643721955|gb|KDP31834.1|hypothetical protein JCGZ_12295 [Jatropha curcas]	XP_012079124, XP_012079125, KDP31834	1.04E-120	203	190
Pe212D7.14		-	6	2992	1452	HGV2-like [Populus euphratica]	483	gi|802641071|ref|XP_012079127.1|PREDICTED: NASP-related protein sim3 [Jatropha curcas]gi|643721958|gb|KDP31837.1|hypothetical protein JCGZ_12298 [Jatropha curcas]	XP_012079127, KDP31837	0.0	474	391
Pe212D7.15		-	3	3331	2100	Wall-associated receptor kinase-like 14	699	gi|802641083|ref|XP_012079130.1|PREDICTED: wall-associated receptor kinase-like 14 [Jatropha curcas]gi|643721961|gb|KDP31840.1|hypothetical protein JCGZ_12301 [Jatropha curcas]	XP_012079130, KDP31840	0.0	684	570
Pe212D7.16		-	1	531	273	At5g66985 [Arabidopsis thaliana]	90	gi|643721963|gb|KDP31842.1|hypothetical protein JCGZ_12303 [Jatropha curcas]	KDP31842	1.56E-14	95	64
Pe212D7.17		-	36	21131	9330	Vacuolar sorting-associated [Theobroma cacao]	3109	gi|743838225|ref|XP_011025659.1|PREDICTED: uncharacterized protein LOC105126489 isoform X3 [Populus euphratica]	XP_011025659	0.0	3171	2339
Pe212D7.18		+	5	2702	1759	Extra-large guanine nucleotide-binding 1	617	gi|802641094|ref|XP_012079134.1|PREDICTED: extra-large guanine nucleotide-binding protein 1 [Jatropha curcas]gi|643721966|gb|KDP31845.1|hypothetical protein JCGZ_12306 [Jatropha curcas]	XP_012079134, KDP31845	0.0	613	510
Pe212I1.1		+	2	845	729	Type I inositol 1,4,5-trisphosphate 5-phosphatase 12-like isoform X2	242	gi|223531453|gb|EEF33286.1|type II inositol 5-phosphatase, putative [Ricinus communis]	EEF33286	4.59E-104	236	199
Pe212I1.2	2 isoforms	+	4	1988	285	Cold-inducible RNA-binding -like	94	gi|802553453|ref|XP_012064989.1|PREDICTED: glycine-rich RNA-binding, abscisic acid-inducible protein isoform X2 [Jatropha curcas]	XP_012064989	1.44E-37	94	81
Pe212I1.3		+	1	489	489	Pathogenesis-related 1-like	162	gi|566187029|ref|XP_006379156.1|pathogenesis-related family protein [Populus trichocarpa]gi|550331336|gb|ERP56953.1|pathogenesis-related family protein [Populus trichocarpa]	XP_006379156, ERP56953	2.17E-62	160	126

Pe212I1.4		+	3	2902	783	---Na---	220	No Blast Hit				
Pe212I1.5		+	1	489	489	Pathogenesis-related 1-like	162	gi|566187029|ref|XP_006379156.1|pathogenesis-related family protein [Populus trichocarpa]gi|550331336|gb|ERP56953.1|pathogenesis-related family protein [Populus trichocarpa]	XP_006379156, ERP56953	7.01E-63	160	125
Pe212I1.6	1 isoform	-	5	1973	468	Glycine-rich RNA-binding mitochondrial-like [Pyrus x bretschneideri]	130	gi|641854127|gb|KDO72935.1|hypothetical protein CISIN_1g034017mg [Citrus sinensis]gi|641854128|gb|KDO72936.1|hypothetical protein CISIN_1g034017mg [Citrus sinensis]gi|641854129|gb|KDO72937.1|hypothetical protein CISIN_1g034017mg [Citrus sinensis]gi|641854130|gb|KDO72938.1|hypothetical protein CISIN_1g034017mg [Citrus sinensis]	KDO72935, KDO72936, KDO72937, KDO72938	2.60E-38	104	84
Pe212I1.7		+	5	5189	2163	ATP-dependent zinc metalloprotease chloroplastic	720	gi|802606394|ref|XP_012073680.1|PREDICTED: ATP-dependent zinc metalloprotease FTSH, chloroplastic [Jatropha curcas]gi|643728895|gb|KDP36832.1|hypothetical protein JCGZ_08123 [Jatropha curcas]	XP_012073680, KDP36832	0.0	721	686
Pe212I1.8		-	1	234	234	[Theobroma cacao]	77	gi|743875328|ref|XP_011034898.1|PREDICTED: uncharacterized protein LOC105132865 [Populus euphratica]	XP_011034898	2.49E-20	64	53
Pe212I1.9		-	1	387	387	Transmembrane [Medicago truncatula]	128	gi|658023878|ref|XP_008347340.1|PREDICTED: uncharacterized protein LOC103410415 [Malus domestica]	XP_008347340	4.53E-44	101	89
Pe212I1.10		-	2	590	489	PREDICTED: uncharacterized protein LOC105635249 [Jatropha curcas]	162	gi|802606386|ref|XP_012073675.1|PREDICTED: uncharacterized protein LOC105635249 [Jatropha curcas]gi|643728890|gb|KDP36827.1|hypothetical protein JCGZ_08118 [Jatropha curcas]	XP_012073675, KDP36827	2.06E-42	146	109
Pe212I1.11	3 isoforms	-	7	3679	1056	PREDICTED: uncharacterized protein LOC107917966 isoform X1 [Gossypium hirsutum]	351	gi|1029085395|ref|XP_016702909.1|PREDICTED: uncharacterized protein LOC107917966 isoform X1 [Gossypium hirsutum]gi|1029085397|ref|XP_016702910.1|PREDICTED: uncharacterized protein LOC107917966 isoform X2 [Gossypium hirsutum]	XP_016702909, XP_016702910	2.20E-169	355	284
Pe212I1.12	1 isoform	-	11	3798	1272	Metallocarboxypeptidase A MCYG_01475 isoform X1	423	gi|743895932|ref|XP_011041244.1|PREDICTED: carboxypeptidase A6-like isoform X1 [Populus euphratica]	XP_011041244	0.0	420	365
Pe212I1.13		-	5	2846	1566	Probable flavin-containing monooxygenase 1	521	gi|743895938|ref|XP_011041247.1|PREDICTED: probable flavin-containing monooxygenase 1 isoform X1 [Populus euphratica]	XP_011041247	0.0	541	421
Pe212I1.14		+	2	977	909	---Na---	302	No Blast Hit				
Pe212I1.15		+	1	396	396	Retrovirus-related Pol poly LINE-1	131	gi|985452739|ref|XP_015386704.1|PREDICTED: uncharacterized protein LOC107177445 [Citrus sinensis]	XP_015386704	1.52E-24	129	81
Pe212I1.16		+	3	1396	933	Ribonuclease H At1g65750 family	310	gi|1029025189|ref|XP_016676456.1|PREDICTED: uncharacterized protein LOC107895735 [Gossypium hirsutum]	XP_016676456	5.36E-22	265	113
Pe212I1.17		+	2	1351	666	PREDICTED: uncharacterized protein LOC8273606	224	gi|1000986445|ref|XP_002510670.2|PREDICTED: uncharacterized protein LOC8273606 [Ricinus communis]	XP_002510670	3.02E-78	220	165
Pe212I1.18		-	8	2622	720	Dolichyl-phosphate beta-D-mannosyltransferase family [Populus trichocarpa]	239	gi|802605979|ref|XP_012073661.1|PREDICTED: dolichol-phosphate mannosyltransferase subunit 1 [Jatropha curcas]gi|643728860|gb|KDP36797.1|hypothetica	XP_012073661, KDP36797	7.90E-159	236	232

								l protein JCGZ_08088 [Jatropha curcas]				
Pe212I1.19		+	5	2489	396	Small nuclear ribonucleo Sm D3	131	gi|351721470|ref|NP_001237722.1|uncharacterized protein LOC100305856 [Glycine max]gi|356525557|ref|XP_003531391.1|PREDICTED: small nuclear ribonucleoprotein SmD3b-like [Glycine max]gi|593793490|ref|XP_007159784.1|hypothetical protein PHAVU_002G266900g [Phaseolus vulgaris]gi|950930956|ref|XP_014505795.1|PREDICTED: small nuclear ribonucleoprotein SmD3b-like [Vigna radiata var. radiata]gi|255626793|gb|ACU13741.1|unknown [Glycine max]gi|561033199|gb|ESW31778.1|hypothetical protein PHAVU_002G266900g [Phaseolus vulgaris]gi|734395773|gb|KHN29131.1|Small nuclear ribonucleoprotein Sm D3 [Glycine soja]gi|920686654|gb|KOM30637.1|hypothetical protein LR48_Vigan01g019100 [Vigna angularis]gi|947094762|gb|KRH43347.1|hypothetical protein GLYMA_08G143900 [Glycine max]gi|947111162|gb|KRH59488.1|hypothetical protein GLYMA_05G185800 [Glycine max]gi|965656894|dbj|BAT73313.1|hypothetical protein VIGAN_01078600 [Vigna angularis var. angularis]	NP_001237722, XP_003531391, XP_007159784, XP_014505795, ACU13741, ESW31778, KHN29131, KOM30637, KRH43347, KRH59488, BAT73313	4.08E-79	131	126
Pe212I1.20	1 isoform	-	12	6141	2415	BTB POZ domain-containing family [Populus trichocarpa]	804	gi|802605639|ref|XP_012073654.1|PREDICTED: BTB/POZ domain-containing protein At2g30600 isoform X1 [Jatropha curcas]gi|802605641|ref|XP_012073655.1|PREDICTED: BTB/POZ domain-containing protein At2g30600 isoform X1 [Jatropha curcas]	XP_012073654, XP_012073655	0.0	804	714
Pe212I1.21	1 isoform	-	11	3986	1293	Cyclin-B2-4-like isoform X1 [Populus euphratica]	430	gi|255539220|ref|XP_002510675.1|PREDICTED: cyclin-B2-3 [Ricinus communis]gi|1000986050|ref|XP_015573607.1|PREDICTED: cyclin-B2-3 [Ricinus communis]gi|223551376|gb|EEF52862.1|cyclin B, putative [Ricinus communis]	XP_002510675, XP_015573607, EEF52862	0.0	436	364
Pe212I1.22		+	4	2839	1416	Catalase isozyme 1	471	gi|823226893|ref|XP_012446280.1|PREDICTED: catalase isozyme 1 [Gossypium raimondii]gi|763790967|gb|KJB57963.1|hypothetical protein B456_009G187600 [Gossypium raimondii]	XP_012446280, KJB57963	0.0	450	416
Pe212I1.23		+	4	4877	2814	NLP4-like [Populus euphratica]	937	gi|1000986724|ref|XP_015575768.1|PREDICTED: protein NLP4 [Ricinus communis]gi|1000986726|ref|XP_015575774.1|PREDICTED: protein NLP4 [Ricinus communis]	XP_015575768, XP_015575774	0.0	941	735
Pe212I1.24		-	35	12694	3567	Niemann-Pick C1 -like	1188	gi|802605623|ref|XP_012073645.1|PREDICTED: Niemann-Pick C1 protein-like [Jatropha curcas]	XP_012073645	0.0	1186	1123
Pe212J12.1	Incomplete 3'	-	2	487	381	Pollenless 3-like 2	127	gi|1009174088|ref|XP_015868163.1|PREDICTED: protein POLLENLESS 3-LIKE 2-like, partial [Ziziphus jujuba]	XP_015868163	5.04E-76	127	121
Pe212J12.2		+	2	1266	678	Myb-related 308-like	225	gi|566170785|ref|XP_006383085.1|hypothetical protein POPTR_0005s11410g [Populus trichocarpa]gi|550338662|gb|ERP60882.1|hypothetical protein POPTR_0005s11410g [Populus trichocarpa]	XP_006383085, ERP60882	1.61E-111	217	188

Pe212J12.3		-	15	4357	1644	MLO 4	547	gi|566170771|ref|XP_006383078.1|hypothetical protein POPTR_0005s11350g [Populus trichocarpa]gi|550338655|gb|ERP60875.1|hypothetical protein POPTR_0005s11350g [Populus trichocarpa]	XP_006383078, ERP60875	0.0	546	434
Pe212J12.4		-	1	636	636	---Na---	211	No Blast Hit				
Pe212J12.5		+	1	405	405	---Na---	134	No Blast Hit				
Pe212J12.6		-	6	1111	411	Thiosulfate sulfurtransferase 18-like isoform X2	136	gi|590702599|ref|XP_007046665.1|Rhodanese/Cell cycle control phosphatase superfamily protein [Theobroma cacao]gi|508698926|gb|EOX90822.1|Rhodanese/Cell cycle control phosphatase superfamily protein [Theobroma cacao]	XP_007046665, EOX90822	1.16E-55	129	107
Pe212M5.1	Incomplete 3'	-	2	1636	1526	Gag protease poly [Theobroma cacao]	508	gi|590728434|ref|XP_007099662.1|Gag protease polyprotein-like protein [Theobroma cacao]gi|508728474|gb|EOY20371.1|Gag protease polyprotein-like protein [Theobroma cacao]	XP_007099662, EOY20371	9.72E-59	405	202
Pe212M5.2		+	1	399	399	---Na---	132	No Blast Hit				
Pe212M5.3		-	1	267	267	---Na---	88	No Blast Hit				
Pe212M5.4		-	2	425	276	---Na---	91	No Blast Hit				
Pe212M5.5		+	1	351	351	---Na---	116	No Blast Hit				
Pe212M5.6		+	4	873	549	PREDICTED: uncharacterized protein LOC107775794	182	gi|1025067023|ref|XP_016451062.1|PREDICTED: uncharacterized protein LOC107775794 [Nicotiana tabacum]	XP_016451062	5.02E-14	228	95
Pe212M5.7		+	2	1592	1566	Ribonuclease h at1g65750	521	gi|659121154|ref|XP_008460525.1|PREDICTED: LOW QUALITY PROTEIN: putative ribonuclease H protein At1g65750 [Cucumis melo]	XP_008460525	4.52E-75	463	246
Pe212M5.8		-	6	3170	1143	Mitochondrial arginine transporter BAC2	380	gi|566150464|ref|XP_006369411.1|hypothetical protein POPTR_0001s23010g [Populus trichocarpa]gi|550347944|gb|ERP65980.1|hypothetical protein POPTR_0001s23010g [Populus trichocarpa]	XP_006369411, ERP65980	0.0	380	331
Pe212M5.9		-	1	471	471	Caffeoylshikimate esterase-like	156	gi|948288846|gb|ALM55688.1|caffeoyl shikimate esterase 14, partial [Populus tomentosa]	ALM55688	9.74E-75	156	133
Pe213C9.1		+	1	3324	2016	Scarecrow 28	671	gi|224058599|ref|XP_002299559.1|hypothetical protein POPTR_0001s08850g [Populus trichocarpa]gi|222846817|gb|EEE84364.1|hypothetical protein POPTR_0001s08850g [Populus trichocarpa]	XP_002299559, EEE84364	0.0	664	589
Pe213C9.2		-	2	1646	1503	Probable (S)-N-methylcoclaurine 3 -hydroxylase isozyme 2	500	gi|224053959|ref|XP_002298058.1|hypothetical protein POPTR_0001s08860g [Populus trichocarpa]gi|222845316|gb|EEE82863.1|hypothetical protein POPTR_0001s08860g [Populus trichocarpa]	XP_002298058, EEE82863	0.0	507	444
Pe213C9.3		-	1	581	234	---Na---	77	No Blast Hit				
Pe213C9.4		+	4	2187	843	F-box PP2-A12-like	280	gi|224074873|ref|XP_002304470.1|F-box family protein [Populus trichocarpa]gi|222841902|gb|EEE79449.1|F-box family protein [Populus trichocarpa]	XP_002304470, EEE79449	1.96E-144	301	237
Pe213C9.5		-	3	728	240	rRNA biogenesis rrp36 isoform X2 [Citrus	79	gi|590677830|ref|XP_007040128.1|Uncharacterized protein TCM_016182 [Theobroma	XP_007040128, EOY24629	1.77E-13	51	45

						sinensis]		cacao]gi|508777373|gb|EOY24629.1|Uncharacterized protein TCM_016182 [Theobroma cacao]				
Pe213C9.6		-	1	351	351	Gag protease poly [Theobroma cacao]	116	gi|590691529|ref|XP_007043808.1|Gag protease polyprotein [Theobroma cacao]gi|508707743|gb|EOX99639.1|Gag protease polyprotein [Theobroma cacao]	XP_007043808, EOX99639	7.17E-04	34	26
Pe213C9.7		+	1	537	537	Retroelement poly -like	178	gi|828297604|ref|XP_012568871.1|PREDICTED: uncharacterized protein LOC105851739 [Cicer arietinum]	XP_012568871	1.13E-20	94	68
Pe213C9.8		+	1	327	327	---Na---	108	No Blast Hit				
Pe213C9.9		+	1	375	375	---Na---	124	No Blast Hit				
Pe213C9.10		-	1	498	498	---Na---	165	No Blast Hit				
Pe213C9.11		+	5	3599	1590	Sugar transport 1-like	529	gi|57283530|emb|CAG27605.1|monosaccharide transporter [Populus tremula x Populus tremuloides]	CAG27605	0.0	507	411
Pe213C9.12		+	1	808	480	---Na---	159	No Blast Hit				
Pe214A18.1	Incomplete 3'	-	8	2431	1053	NAP1 isoform X1	351	gi|743856487|ref|XP_011029922.1|PREDICTED: protein NAP1-like isoform X2 [Populus euphratica]	XP_011029922	0.0	353	342
Pe214A18.2		-	5	1549	378	Chloroplastic mitochondrial	125	gi|743856495|ref|XP_011029924.1|PREDICTED: sulfiredoxin, chloroplastic/mitochondrial [Populus euphratica]	XP_011029924	3.66E-70	125	116
Pe214A18.3		-	4	1898	1251	Probable arabinosyltransferase ARAD1	416	gi|590681398|ref|XP_007041077.1|Exostosin family protein isoform 1 [Theobroma cacao]gi|508705012|gb|EOX96908.1|Exostosin family protein isoform 1 [Theobroma cacao]	XP_007041077, EOX96908	3.77E-156	462	307
Pe214A18.4		+	2	676	582	Membrane steroid-binding 2-like	193	gi|566207660|ref|XP_002321821.2|cytochrome b5 domain-containing family protein [Populus trichocarpa]gi|550322745|gb|EEF05948.2|cytochrome b5 domain-containing family protein [Populus trichocarpa]	XP_002321821, EEF05948	2.76E-89	190	158
Pe214A18.5	1 isoform	-	11	6314	1593	Coatomer subunit delta-like [Malus domestica]	530	gi|802613420|ref|XP_012074712.1|PREDICTED: coatomer subunit delta [Jatropha curcas]gi|802613422|ref|XP_012074713.1|PREDICTED: coatomer subunit delta [Jatropha curcas]gi|643727186|gb|KDP35720.1|hypothetical protein JCGZ_10492 [Jatropha curcas]	XP_012074712, XP_012074713, KDP35720	0.0	529	499
Pe214A18.6		+	1	966	966	Polyadenylate-binding 1-B-binding [Theobroma cacao]	321	gi|802659893|ref|XP_012080898.1|PREDICTED: uncharacterized protein LOC105641058 [Jatropha curcas]gi|802659909|ref|XP_012080899.1|PREDICTED: uncharacterized protein LOC105641058 [Jatropha curcas]gi|643719952|gb|KDP30542.1|hypothetical protein JCGZ_15251 [Jatropha curcas]	XP_012080898, XP_012080899, KDP30542	0.0	321	294
Pe214A18.7		-	5	2990	897	Peroxisomal 2,4-dienoyl-reductase	298	gi|743856547|ref|XP_011029941.1|PREDICTED: peroxisomal 2,4-dienoyl-CoA reductase-like [Populus euphratica]gi|743856549|ref|XP_011029942.1|PREDICTED: peroxisomal 2,4-dienoyl-CoA reductase-like [Populus euphratica]	XP_011029941, XP_011029942	3.30E-167	299	268
Pe214A18.8	1 isoform	+	3	2863	1230	BAG family molecular chaperone regulator 7	409	gi|255539763|ref|XP_002510946.1|PREDICTED: BAG family molecular chaperone regulator 7 [Ricinus communis]gi|223550061|gb|EEF51548.1|conserv	XP_002510946, EEF51548	3.15E-164	406	317

								ed hypothetical protein [Ricinus communis]				
Pe214A18.9		+	11	5500	1158	Trehalose-phosphate phosphatase A-like [Glycine max]	396	gi|255539761|ref|XP_002510945.1|PREDICTED: trehalose-phosphate phosphatase A [Ricinus communis]gi|1000985472|ref|XP_015581627.1|PREDICTED: trehalose-phosphate phosphatase A [Ricinus communis]gi|1000985474|ref|XP_015581628.1|PREDICTED: trehalose-phosphate phosphatase A [Ricinus communis]gi|1000985476|ref|XP_015581633.1|PREDICTED: trehalose-phosphate phosphatase A [Ricinus communis]gi|223550060|gb|EEF51547.1|trehalose-6-phosphate synthase, putative [Ricinus communis]	XP_002510945, XP_015581627, XP_015581628, XP_015581633, EEF51547	0.0	386	347
Pe214A18.10		+	2	852	582	Ribonuclease h at1g65750	193	gi|702284285|ref|XP_010046135.1|PREDICTED: uncharacterized protein LOC104434998 [Eucalyptus grandis]	XP_010046135	1.19E-24	164	96
Pe214A18.11		+	1	348	348	---Na---	115	No Blast Hit				
Pe214A18.12		+	3	4230	1956	Gag protease poly [Theobroma cacao]	671	gi|590728434|ref|XP_007099662.1|Gag protease polyprotein-like protein [Theobroma cacao]gi|508728474|gb|EOY20371.1|Gag protease polyprotein-like protein [Theobroma cacao]	XP_007099662, EOY20371	1.04E-51	371	186
Pe214A18.13		-	1	276	276	---Na---	91	No Blast Hit				
Pe214A18.14		+	2	806	408	---Na---	135	No Blast Hit				
Pe214A18.15		+	1	243	243	---Na---	80	No Blast Hit				
Pe214A18.16		+	1	810	810	Gag protease poly [Theobroma cacao]	269	gi|823238574|ref|XP_012451939.1|PREDICTED: uncharacterized protein LOC105774125 [Gossypium raimondii]	XP_012451939	1.98E-15	240	113
Pe214A18.17		+	2	806	408	---Na---	135	No Blast Hit				
Pe214A18.18		+	1	249	249	---Na---	82	No Blast Hit				
Pe214A18.19		+	1	2184	2184	Ribonuclease H At1g65750	727	gi|985449423|ref|XP_015385965.1|PREDICTED: uncharacterized protein LOC107177137 [Citrus sinensis]	XP_015385965	6.48E-145	729	384
Pe214A18.20		+	9	3590	1773	Monocopper oxidase SKS1	590	gi|255539757|ref|XP_002510943.1|PREDICTED: monocopper oxidase-like protein SKS1 [Ricinus communis]gi|223550058|gb|EEF51545.1|multicopper oxidase, putative [Ricinus communis]	XP_002510943, EEF51545	0.0	582	523
Pe214A18.21		-	3	1256	1071	NAC domain-containing 7	356	gi|743856571|ref|XP_011029951.1|PREDICTED: NAC domain-containing protein 7-like [Populus euphratica]	XP_011029951	0.0	363	314
Pe214H11.1	Incomplete 3'	-	1	1268	1169	Pentatricopeptide repeat-containing mitochondrial	389	gi|743895123|ref|XP_011040829.1|PREDICTED: pentatricopeptide repeat-containing protein At2g03880, mitochondrial [Populus euphratica]	XP_011040829	0.0	358	304
Pe214H11.2		+	10	3444	1104	PREDICTED: uncharacterized protein At2g17340 isoform X1 [Gossypium hirsutum]	367	gi|1029090389|ref|XP_016705273.1|PREDICTED: uncharacterized protein At2g17340 isoform X1 [Gossypium hirsutum]gi|1029090391|ref|XP_016705274.1|PREDICTED: uncharacterized protein At2g17340 isoform X1 [Gossypium hirsutum]	XP_016705273, XP_016705274	0.0	367	346
Pe214H11.3		+	1	1574	441	PREDICTED: uncharacterized protein	162	gi|1009141847|ref|XP_015888405.1|PREDICTED: uncharacterized protein LOC107423372	XP_015888405	2.71E-75	167	143

						LOC107423372		[Ziziphus jujuba]				
Pe214H11.4	1 isoform	-	7	5770	1686	Calcium-dependent kinase 26-like [Populus euphratica]	561	gi|743913884|ref|XP_011000864.1|PREDICTED: calcium-dependent protein kinase 26 [Populus euphratica]gi|743913886|ref|XP_011000866.1|PREDICTED: calcium-dependent protein kinase 26 [Populus euphratica]gi|743913888|ref|XP_011000867.1|PREDICTED: calcium-dependent protein kinase 26 [Populus euphratica]gi|743913890|ref|XP_011000868.1|PREDICTED: calcium-dependent protein kinase 26 [Populus euphratica]	XP_011000864, XP_011000866, XP_011000867, XP_011000868	0.0	564	530
Pe214H11.5	2 isoforms	+	9	5452	1590	Bromodomain and PHD finger-containing 3 [Morus notabilis]	529	gi|802607766|ref|XP_012073976.1|PREDICTED: uncharacterized protein LOC105635519 [Jatropha curcas]gi|643728488|gb|KDP36533.1|hypothetical protein JCGZ_08870 [Jatropha curcas]	XP_012073976, KDP36533	0.0	548	401
Pe214H11.6		+	6	1830	1197	Probable serine threonine-kinase chloroplastic [Gossypium hirsutum]	398	gi|566188383|ref|XP_002312803.2|hypothetical protein POPTR_0009s16860g [Populus trichocarpa]gi|550331888|gb|EEE86758.2|hypothetical protein POPTR_0009s16860g [Populus trichocarpa]	XP_002312803, EEE86758	0.0	406	338
Pe214H11.7	1 isoform	+	39	13956	3837	Niemann-pick c1	1278	gi|1000950294|ref|XP_015579647.1|PREDICTED: Niemann-Pick C1 protein isoform X2 [Ricinus communis]	XP_015579647	0.0	1278	1138
Pe214H11.8	2 isoforms	+	2	4272	2460	Pentatricopeptide repeat-containing At2g17140-like [Ziziphus jujuba]	871	gi|802563966|ref|XP_012067167.1|PREDICTED: pentatricopeptide repeat-containing protein At2g17140 [Jatropha curcas]	XP_012067167	0.0	871	756
Pe214H11.9		-	4	3936	2721	NLP1 [Ricinus communis]	906	gi|743913928|ref|XP_011000886.1|PREDICTED: protein NLP2-like [Populus euphratica]gi|743913930|ref|XP_011000887.1|PREDICTED: protein NLP2-like [Populus euphratica]gi|743913932|ref|XP_011000889.1|PREDICTED: protein NLP2-like [Populus euphratica]	XP_011000886, XP_011000887, XP_011000889	0.0	950	682
Pe214H11.10		-	3	2290	174	---Na---	57	No Blast Hit				
Pe214H11.11-12		-	7	2767	1197	Ubiquitin domain-containing DSK2a-like	122	gi|763780695|gb|KJB47766.1|hypothetical protein B456_008G041000 [Gossypium raimondii]	KJB47766	1.35E-41	126	102
Pe214H11.13		-	4	2789	1929	U-box domain-containing 15-like	642	gi|590683533|ref|XP_007041623.1|U-box domain-containing protein 15 isoform 1 [Theobroma cacao]gi|508705558|gb|EOX97454.1|U-box domain-containing protein 15 isoform 1 [Theobroma cacao]	XP_007041623, EOX97454	0.0	626	553
Pe214H11.14		+	2	2006	1872	SEC1 family transport SLY1-like	623	gi|743895028|ref|XP_011040777.1|PREDICTED: SEC1 family transport protein SLY1-like [Populus euphratica]	XP_011040777	0.0	621	600
Pe214H11.15		-	19	13757	3246	MATE efflux family chloroplastic-like [Populus euphratica]	1081	gi|566167377|ref|XP_002305555.2|hypothetical protein POPTR_0004s19430g [Populus trichocarpa]gi|550341386|gb|EEE86066.2|hypothetical protein POPTR_0004s19430g [Populus trichocarpa]	XP_002305555, EEE86066	0.0	588	497
Pe214H11.16		+	9	2885	1392	O-fucosyltransferase family [Theobroma cacao]	463	gi|224080776|ref|XP_002306226.1|hypothetical protein POPTR_0004s19390g [Populus trichocarpa]gi|222849190|gb|EEE86737.1|hypothetical protein POPTR_0004s19390g [Populus	XP_002306226, EEE86737	0.0	437	381

								trichocarpa]				
Pe214H11.17	1 isoform	+	4	5650	2487	DNA-directed RNA polymerase subunit beta	828	gi|743792708|ref|XP_011045186.1|PREDICTED : uncharacterized protein LOC105140165 isoform X1 [Populus euphratica]	XP_011045186	0.0	850	650
Pe214H11.18		+	9	3662	1512	Probable serine threonine-kinase At4g35230 isoform X1	503	gi|224080762|ref|XP_002306223.1|kinase family protein [Populus trichocarpa]gi|222849187|gb|EEE86734.1|kinase family protein [Populus trichocarpa]	XP_002306223, EEE86734	0.0	505	477
Pe214H11.19		-	3	1053	852	No apical meristem family [Populus trichocarpa]	283	gi|925170183|gb|ALC79008.1|NAC transcription factors 31 [Manihot esculenta]	ALC79008	6.00E-114	281	217
Pe214H11.20	1 isoform	-	5	2781	786	Expansin-like A2	261	gi|743792752|ref|XP_011045305.1|PREDICTED : expansin-like A2 [Populus euphratica]	XP_011045305	6.07E-163	261	241
Pe214H11.21	3 isoforms	-	13	6479	2958	Probable alanine--tRNA chloroplastic isoform X1	985	gi|224105089|ref|XP_002313683.1|hypothetical protein POPTR_0009s14340g [Populus trichocarpa]gi|313471500|sp|B9HQZ6.1|SYAP_POPTRRecName: Full=Alanine--tRNA ligase, chloroplastic/mitochondrial; AltName: Full=Alanyl-tRNA synthetase; Short=AlaRS; Flags: Precursorgi|222850091|gb|EEE87638.1|hypothetical protein POPTR_0009s14340g [Populus trichocarpa]	XP_002313683, B9HQZ6, EEE87638	0.0	999	900
Pe214H11.22		+	2	799	222	Lysine histidine transporter-like 8	73	gi|255575021|ref|XP_002528416.1|PREDICTED : lysine histidine transporter-like 8 [Ricinus communis]gi|223532152|gb|EEF33958.1|amino acid transporter, putative [Ricinus communis]	XP_002528416, EEF33958	1.39E-10	40	37
Pe214H11.23		+	1	1247	450	SAUR-like auxin-responsive [Theobroma cacao]	149	gi|743792777|ref|XP_011045381.1|PREDICTED : uncharacterized protein LOC105140298 [Populus euphratica]	XP_011045381	4.02E-51	147	111
Pe214H11.24		+	5	1876	1524	Lysine histidine transporter-like 8	507	gi|743905803|ref|XP_011046310.1|PREDICTED : lysine histidine transporter-like 8 [Populus euphratica]	XP_011046310	0.0	511	386
Pe214H11.25		-	3	1896	912	Group 2 isoform 1 [Theobroma cacao]	303	gi|255575029|ref|XP_002528420.1|PREDICTED : uncharacterized protein LOC8265944 [Ricinus communis]gi|223532156|gb|EEF33962.1|hypothetical protein RCOM_0615680 [Ricinus communis]	XP_002528420, EEF33962	4.90E-173	313	273
Pe214H11.26		-	1	2553	1182	Signal recognition particle 43 kda chloroplastic	393	gi|802756621|ref|XP_012089050.1|PREDICTED : signal recognition particle 43 kDa protein, chloroplastic [Jatropha curcas]	XP_012089050	0.0	384	331
Pe214H11.27		+	8	3145	1623	L-ascorbate oxidase homolog	540	gi|743905809|ref|XP_011046313.1|PREDICTED : L-ascorbate oxidase homolog [Populus euphratica]	XP_011046313	0.0	539	463
Pe214H11.28		-	3	2145	690	Coiled-coil domain-containing 124	229	gi|703125402|ref|XP_010103296.1|hypothetical protein L484_014336 [Morus notabilis]gi|587907354|gb|EXB95363.1|hypothetical protein L484_014336 [Morus notabilis]	XP_010103296, EXB95363	2.94E-120	229	217
Pe214H11.29	1 isoform	+	5	3678	1683	Rop guanine nucleotide exchange factor 1	560	gi|802716071|ref|XP_012084870.1|PREDICTED : rop guanine nucleotide exchange factor 1 [Jatropha curcas]gi|643714751|gb|KDP27131.1|hypothetical protein JCGZ_20943 [Jatropha curcas]	XP_012084870, KDP27131	0.0	576	513
Pe214H11.30		+	9	6899	4572	Transcriptional elongation regulator MINIYO	1523	gi|566187935|ref|XP_002312932.2|hypothetical protein POPTR_0009s14190g [Populus trichocarpa]gi|550331699|gb|EEE86887.2|hypothetical protein POPTR_0009s14190g [Populus trichocarpa]	XP_002312932, EEE86887	0.0	1529	1104

Pe214N19.1	Incomplete 3'	-	1	302	302	WPP domain-associated	100	gi|643739291|gb|KDP45087.1|hypothetical protein JCGZ_18529 [Jatropha curcas]	KDP45087	2.04E-37	100	80
Pe214N19.2		-	1	1011	1011	Caffeoylshikimate esterase-like	336	gi|743895458|ref|XP_011040996.1|PREDICTED : caffeoylshikimate esterase [Populus euphratica]	XP_011040996	0.0	322	303
Pe214N19.3		-	2	2285	654	Aquaporin PIP2-2	217	gi|1012247423|ref|XP_015942637.1|PREDICTED: aquaporin PIP2-2 [Arachis duranensis]	XP_015942637	4.96E-111	209	184
Pe214N19.4		+	3	970	456	---Na---	151	No Blast Hit				
Pe214N19.5		+	1	407	189	---Na---	62	No Blast Hit				
Pe214N19.6		+	1	234	234	---Na---	77	No Blast Hit				
Pe214N19.7		+	1	1779	1779	Gag protease poly [Theobroma cacao]	592	gi|590581218|ref|XP_007014287.1|Gag protease polyprotein [Theobroma cacao]gi|508784650|gb|EOY31906.1|Gag protease polyprotein [Theobroma cacao]	XP_007014287, EOY31906	6.17E-47	313	163
Pe214N19.8		+	2	2057	588	DNA RNA polymerases superfamily [Theobroma cacao]	195	gi|590566582|ref|XP_007010273.1|DNA/RNA polymerases superfamily protein [Theobroma cacao]gi|508727186|gb|EOY19083.1|DNA/RNA polymerases superfamily protein [Theobroma cacao]	XP_007010273, EOY19083	1.47E-34	189	110
Pe214N19.9		-	1	582	582	---Na---	193	No Blast Hit				
Pe214N19.10		-	2	938	789	---Na---	262	No Blast Hit				
Pe214N19.11		+	1	357	357	---Na---	118	No Blast Hit				
Pe214N19.12		+	1	375	201	---Na---	66	No Blast Hit				
Pe214N19.13		+	3	815	384	---Na---	127	No Blast Hit				
Pe214N19.14		+	1	1140	1140	Gag protease poly [Theobroma cacao]	379	gi|590581218|ref|XP_007014287.1|Gag protease polyprotein [Theobroma cacao]gi|508784650|gb|EOY31906.1|Gag protease polyprotein [Theobroma cacao]	XP_007014287, EOY31906	5.66E-46	333	163
Pe214N19.15		-	2	754	696	---Na---	231	No Blast Hit				
Pe214N19.16		-	2	5067	1992	Gag protease poly [Theobroma cacao]	663	gi|590728434|ref|XP_007099662.1|Gag protease polyprotein-like protein [Theobroma cacao]gi|508728474|gb|EOY20371.1|Gag protease polyprotein-like protein [Theobroma cacao]	XP_007099662, EOY20371	1.05E-84	490	257
Pe214N19.17		-	3	1047	396	---Na---	131	No Blast Hit				
Pe214N19.18		+	2	1705	576	Retrotransposon Ty1-copia subclass	191	gi|960485211|ref|XP_014757768.1|PREDICTED : uncharacterized protein LOC106866668, partial [Brachypodium distachyon]	XP_014757768	2.08E-14	106	60
Pe214N19.19		-	8	4161	969	mRNA-decapping enzyme subunit 2-like	322	gi|743895471|ref|XP_011041004.1|PREDICTED : mRNA-decapping enzyme subunit 2-like [Populus euphratica]	XP_011041004	0.0	322	302
Pe214N19.20	Incomplete 3'	+	13	5913	2470	Endoplasmic reticulum metallopeptidase 1 isoform X1	823	gi|802550944|ref|XP_012093256.1|PREDICTED : endoplasmic reticulum metallopeptidase 1 isoform X2 [Jatropha curcas]gi|643738412|gb|KDP44365.1|hypothetical protein JCGZ_20045 [Jatropha curcas]	XP_012093256, KDP44365	0.0	832	722
Pe215I8.1	Incomplete 5'	+	4	2203	1433	Mitochondrial transcription termination factor family isoform 1 [Theobroma cacao]	476	gi|566212535|ref|XP_006373250.1|hypothetical protein POPTR_0017s10070g [Populus trichocarpa]gi|550319956|gb|ERP51047.1|hypothetical protein POPTR_0017s10070g [Populus trichocarpa]	XP_006373250, ERP51047	4.82E-163	448	329
Pe215I8.2		-	1	894	894	Ribonuclease h at1g65750	297	gi|685385687|ref|XP_009124780.1|PREDICTED : putative ribonuclease H protein At1g65750	XP_009124780	4.21E-36	261	132

							[Brassica rapa]					
Pe215I8.3		+	2	489	183	---Na---	60	No Blast Hit				
Pe215I8.4		+	2	867	747	Mitochondrial transcription termination factor family isoform 2 [Theobroma cacao]	248	gi|590601537|ref|XP_007019634.1|Mitochondrial transcription termination factor family protein isoform 2 [Theobroma cacao]gi|508724962|gb|EOY16859.1|Mitochondrial transcription termination factor family protein isoform 2 [Theobroma cacao]	XP_007019634, EOY16859	6.79E-66	271	162
Pe215I8.5		-	4	2917	753	Phosducin 3	250	gi|595941067|ref|XP_007215853.1|hypothetical protein PRUPE_ppa010366mg [Prunus persica]gi|462412003|gb|EMJ17052.1|hypothetical protein PRUPE_ppa010366mg [Prunus persica]	XP_007215853, EMJ17052	1.03E-147	252	231
Pe215I8.6-7-8		+	12	3042	1017	COP9 signalosome complex subunit 3-like isoform X2	74	gi|255536979|ref|XP_002509556.1|PREDICTED: COP9 signalosome complex subunit 3 [Ricinus communis]gi|223549455|gb|EEF50943.1|26S proteasome regulatory subunit S3, putative [Ricinus communis]	XP_002509556, EEF50943	8.05E-10	56	42
Pe215I8.9		-	7	6278	3501	ARM repeat superfamily [Theobroma cacao]	1166	gi|802539411|ref|XP_012070910.1|PREDICTED: protein TPLATE [Jatropha curcas]gi|643740732|gb|KDP46322.1|hypothetical protein JCGZ_10162 [Jatropha curcas]	XP_012070910, KDP46322	0.0	1166	1114
Pe215I8.10		+	1	479	285	---Na---	94	No Blast Hit				
Pe215I8.11		+	1	312	312	Ribonuclease H At1g65750 family	103	gi|1012365003|gb|KYP76185.1|Putative ribonuclease H protein At1g65750 [Cajanus cajan]	KYP76185	6.40E-09	96	54
Pe215I8.12		+	6	2571	1668	4-coumarate-- ligase-like 9	555	gi|566211730|ref|XP_006372917.1|hypothetical protein POPTR_0017s06220g [Populus trichocarpa]gi|550319565|gb|ERP50714.1|hypothetical protein POPTR_0017s06220g [Populus trichocarpa]	XP_006372917, ERP50714	0.0	555	420
Pe215I8.13		-	6	2878	1635	4-coumarate-- ligase-like 9	544	gi|566211730|ref|XP_006372917.1|hypothetical protein POPTR_0017s06220g [Populus trichocarpa]gi|550319565|gb|ERP50714.1|hypothetical protein POPTR_0017s06220g [Populus trichocarpa]	XP_006372917, ERP50714	0.0	544	460
Pe215I8.14	2 isoforms	+	7	3633	933	Cytochrome c1- heme mitochondrial	310	gi|802539419|ref|XP_012070956.1|PREDICTED: cytochrome c1-2, heme protein, mitochondrial [Jatropha curcas]gi|802539421|ref|XP_012070965.1|PREDICTED: cytochrome c1-2, heme protein, mitochondrial [Jatropha curcas]gi|802539423|ref|XP_012070973.1|PREDICTED: cytochrome c1-2, heme protein, mitochondrial [Jatropha curcas]gi|643740736|gb|KDP46326.1|hypothetical protein JCGZ_10166 [Jatropha curcas]	XP_012070956, XP_012070965, XP_012070973, KDP46326	0.0	310	297
Pe215I8.15		+	1	651	180	---Na---	59	No Blast Hit				
Pe215I8.16		+	1	3998	978	Hypothetical protein POPTR_0001s34210g [Populus trichocarpa]	325	gi|224055683|ref|XP_002298601.1|hypothetical protein POPTR_0001s34210g [Populus trichocarpa]gi|222845859|gb|EEE83406.1|hypothetical protein POPTR_0001s34210g [Populus trichocarpa]	XP_002298601, EEE83406	8.80E-76	358	209
Pe215I8.17	3 isoforms	-	5	2534	657	Thymidylate kinase isoform X3 [Populus	258	gi|743903650|ref|XP_011045178.1|PREDICTED: thymidylate kinase isoform X3 [Populus	XP_011045178, XP_011045179,	3.41E-132	260	224

						euphratica]		euphratica]gi|743903652|ref|XP_011045179.1|PREDICTED: thymidylate kinase isoform X3 [Populus euphratica]gi|743903654|ref|XP_011045180.1|PREDICTED: thymidylate kinase isoform X3 [Populus euphratica]gi|743903656|ref|XP_011045181.1|PREDICTED: thymidylate kinase isoform X3 [Populus euphratica]	XP_011045180, XP_011045181			
Pe215I8.18		+	10	6901	2211	Transcription factor GTE8 [Prunus mume]	736	gi|802539435|ref|XP_012071037.1|PREDICTED: transcription factor GTE8-like [Jatropha curcas]gi|643740740|gb|KDP46330.1|hypothetical protein JCGZ_10170 [Jatropha curcas]	XP_012071037, KDP46330	0.0	743	562
Pe215I8.19		-	3	1025	654	Lysine histidine transporter 1-like	217	gi|802539439|ref|XP_012071062.1|PREDICTED: lysine histidine transporter 1-like [Jatropha curcas]gi|643740742|gb|KDP46332.1|hypothetical protein JCGZ_10172 [Jatropha curcas]	XP_012071062, KDP46332	4.25E-113	191	176
Pe215I8.20		-	2	3559	2049	Ankyrin repeat-containing isoform 1 [Theobroma cacao]	743	gi|743903680|ref|XP_011045195.1|PREDICTED: tankyrase-2 [Populus euphratica]	XP_011045195	0.0	681	600
Pe215I8.21		-	11	2886	1011	Glyceraldehyde-3-phosphate dehydrogenase	336	gi|802539456|ref|XP_012071155.1|PREDICTED: glyceraldehyde-3-phosphate dehydrogenase, cytosolic [Jatropha curcas]gi|643740748|gb|KDP46338.1|hypothetical protein JCGZ_10178 [Jatropha curcas]	XP_012071155, KDP46338	0.0	336	327
Pe215I8.22	1 isoform	-	15	5225	1548	Glucose-6-phosphate 1-cytoplasmic isoform [Vitis vinifera]	515	gi|743931921|ref|XP_011010244.1|PREDICTED: glucose-6-phosphate 1-dehydrogenase, cytoplasmic isoform 2 [Populus euphratica]gi|743935938|ref|XP_011012349.1|PREDICTED: glucose-6-phosphate 1-dehydrogenase, cytoplasmic isoform 2 [Populus euphratica]	XP_011010244, XP_011012349	0.0	515	492
Pe215I8.23		+	13	7172	2208	HAUS augmin-like complex subunit 6 [Theobroma cacao]	735	gi|802539470|ref|XP_012071208.1|PREDICTED: uncharacterized protein LOC105633240 [Jatropha curcas]	XP_012071208	0.0	736	662
Pe215I8.24	2 isoforms	+	2	3807	1563	Serine threonine phosphatase 2A 57 kda regulatory subunit B theta isoform-like [Gossypium hirsutum]	520	gi|1009124134|ref|XP_015878903.1|PREDICTED: serine/threonine protein phosphatase 2A 57 kDa regulatory subunit B' theta isoform-like [Ziziphus jujuba]	XP_015878903	0.0	526	470
Pe215I8.25		+	2	2833	1359	Probable carboxylesterase 11	452	gi|802539480|ref|XP_012071244.1|PREDICTED: probable carboxylesterase 11 [Jatropha curcas]gi|643740755|gb|KDP46345.1|hypothetical protein JCGZ_10185 [Jatropha curcas]	XP_012071244, KDP46345	0.0	477	407
Pe215I8.26		+	18	8238	3360	Acting on ester bonds isoform 2 [Theobroma cacao]	1119	gi|743903753|ref|XP_011045233.1|PREDICTED: uncharacterized protein LOC105140193 isoform X1 [Populus euphratica]	XP_011045233	0.0	1119	980
Pe215I8.27		-	6	2160	1185	Upf0392 rcom_0530710	394	gi|643740756|gb|KDP46346.1|hypothetical protein JCGZ_10186 [Jatropha curcas]	KDP46346	1.06E-153	409	298
Pe215I8.28	Incomplete 3'	+	2	668	263	Myb-related 315-like	103	gi|728820984|gb|KHG04077.1|Myb-related protein [Gossypium arboreum]	KHG04077	8.04E-64	103	102
Pe216B2.1	Incomplete 5'	+	1	218	218	Crocetin chloroplastic-like	218	gi|743817028|ref|XP_011020320.1|PREDICTED: crocetin glucosyltransferase, chloroplastic-like [Populus euphratica]	XP_011020320	4.12E-15	71	55
Pe216B2.2		-	3	1656	816	Brassinosteroid-regulated BRU1-like	816	gi|764573164|ref|XP_011462834.1|PREDICTED: xyloglucan endotransglucosylase/hydrolase 2-like [Fragaria vesca subsp. vesca]	XP_011462834	4.57E-150	268	245

Pe216B2.3		-	6	5248	2919	Dentin sialophospho	2919	gi|1000963013|ref|XP_015575602.1|PREDICTED: uncharacterized protein LOC8270577 isoform X1 [Ricinus communis]	XP_015575602	1.26E-161	1128	585
Pe216B2.4	1 isoform	-	24	15969	3819	Insulinase (Peptidase family M16) family isoform 2 [Theobroma cacao]	3819	gi|566159636|ref|XP_002301748.2|hypothetical protein POPTR_0002s23680g [Populus trichocarpa]gi|550345688|gb|EEE81021.2|hypothetical protein POPTR_0002s23680g [Populus trichocarpa]	XP_002301748, EEE81021	0.0	1282	1113
Pe216B2.5		-	10	6695	1143	30S ribosomal S1-like [Gossypium hirsutum]	1143	gi|590655984|ref|XP_007034140.1|Nucleic acid-binding proteins superfamily isoform 1 [Theobroma cacao]gi|508713169|gb|EOY05066.1|Nucleic acid-binding proteins superfamily isoform 1 [Theobroma cacao]	XP_007034140, EOY05066	1.68E-162	290	257
Pe216B2.6		-	2	798	717	PRD1	717	gi|802639763|ref|XP_012078523.1|PREDICTED: protein PRD1 [Jatropha curcas]	XP_012078523	6.45E-20	203	127
Pe216B22.1		-	2	4076	1044	DNAJ homolog subfamily B member 1	1044	gi|802652785|ref|XP_012080195.1|PREDICTED: DNAJ homolog subfamily B member 1 [Jatropha curcas]gi|643720930|gb|KDP31194.1|hypothetical protein JCGZ_11570 [Jatropha curcas]	XP_012080195, KDP31194	3.67E-172	350	310
Pe216B22.2		+	2	2256	1335	Endonuclease or glycosyl hydrolase with C2H2-type zinc finger isoform 1 [Theobroma cacao]	1335	gi|731416017|ref|XP_010659748.1|PREDICTED: uncharacterized protein LOC100242533 isoform X2 [Vitis vinifera]	XP_010659748	1.00E-94	411	250
Pe216B22.3	2 isoforms	+	1	4962	2703	Pentatricopeptide repeat-containing At1g74750 [Vitis vinifera]	2703	gi|743850307|ref|XP_011028698.1|PREDICTED: pentatricopeptide repeat-containing protein At1g18900-like [Populus euphratica]gi|743850311|ref|XP_011028699.1|PREDICTED: pentatricopeptide repeat-containing protein At1g18900-like [Populus euphratica]	XP_011028698, XP_011028699	0.0	906	772
Pe216B22.4		+	15	8815	3768	Ubiquitin carboxyl-terminal hydrolase-related isoform 1 [Theobroma cacao]	3768	gi|802652762|ref|XP_012080189.1|PREDICTED: uncharacterized protein LOC105640478 isoform X1 [Jatropha curcas]gi|643720927|gb|KDP31191.1|hypothetical protein JCGZ_11567 [Jatropha curcas]	XP_012080189, KDP31191	0.0	1278	1019
Pe216B22.5		+	2	1013	816	LOB domain 27 [Populus trichocarpa]	816	gi|743898653|ref|XP_011042621.1|PREDICTED: LOB domain-containing protein 27 [Populus euphratica]	XP_011042621	5.50E-128	274	233
Pe216B22.6	2 isoforms	+	9	5210	1596	WW domain-binding 11	1596	gi|645268718|ref|XP_008239663.1|PREDICTED: formin-like protein 20 [Prunus mume]	XP_008239663	4.32E-99	539	414
Pe216B22.8		+	7	3281	2088	HIPL1 -like	2088	gi|255540863|ref|XP_002511496.1|PREDICTED: HIPL1 protein isoform X1 [Ricinus communis]gi|1000984157|ref|XP_015577451.1|PREDICTED: HIPL1 protein isoform X1 [Ricinus communis]gi|223550611|gb|EEF52098.1|HIPL1 protein precursor, putative [Ricinus communis]	XP_002511496, XP_015577451, EEF52098	0.0	654	564
Pe216B22.9		+	12	7482	2229	DDT domain-containing DDR4-like isoform X2	2229	gi|566197249|ref|XP_002318002.2|hypothetical protein POPTR_0012s07420g [Populus trichocarpa]gi|550326583|gb|EEE96222.2|hypothetical protein POPTR_0012s07420g [Populus trichocarpa]	XP_002318002, EEE96222	0.0	731	626
Pe216B22.10		-	7	4567	1962	Probable beta-1,3-galactosyltransferase 19	1962	gi|802652705|ref|XP_012080173.1|PREDICTED: probable beta-1,3-galactosyltransferase 19 [Jatropha curcas]gi|643720917|gb|KDP31181.1|hypothetical protein JCGZ_11557 [Jatropha curcas]	XP_012080173, KDP31181	0.0	675	591

Pe216B22.11		+	2	1986	978	Dynein light chain type 1 family isoform 2 [Theobroma cacao]	978	gi|255561632|ref|XP_002521826.1|PREDICTED: uncharacterized protein LOC8283253 [Ricinus communis]gi|223539039|gb|EEF40636.1|axonemal dynein light chain, putative [Ricinus communis]	XP_002521826, EEF40636	5.40E-89	326	215
Pe216B22.12		+	6	3013	846	Transcription factor bhlh79 isoform X1	846	gi|568845700|ref|XP_006476705.1|PREDICTED: transcription factor bHLH79 [Citrus sinensis]	XP_006476705	1.44E-130	284	228
Pe216B22.13		-	7	3010	795	5-formyltetrahydrofolate cyclo- mitochondrial	795	gi|566197259|ref|XP_002318620.2|hypothetical protein POPTR_0012s07480g [Populus trichocarpa]gi|550326587|gb|EEE96840.2|hypothetical protein POPTR_0012s07480g [Populus trichocarpa]	XP_002318620, EEE96840	2.74E-120	257	217
Pe216B22.14		+	6	1862	921	Type 2A phosphatase-2 [Populus trichocarpa]	921	gi|823211886|ref|XP_012438703.1|PREDICTED: serine/threonine-protein phosphatase PP2A catalytic subunit-like [Gossypium raimondii]gi|1028966628|ref|XP_016726839.1|PREDICTED: serine/threonine-protein phosphatase PP2A catalytic subunit-like [Gossypium hirsutum]gi|728814029|gb|KHG01412.1|Serine/threonine-protein phosphatase PP2A catalytic subunit [Gossypium arboreum]gi|763783777|gb|KJB50848.1|hypothetical protein B456_008G189700 [Gossypium raimondii]gi|763783778|gb|KJB50849.1|hypothetical protein B456_008G189700 [Gossypium raimondii]	XP_012438703, XP_016726839, KHG01412, KJB50848, KJB50849	0.0	306	303
Pe216B22.15		-	2	2011	1719	Probable myosin-binding 5	1719	gi|255540837|ref|XP_002511483.1|PREDICTED: probable myosin-binding protein 5 isoform X2 [Ricinus communis]gi|223550598|gb|EEF52085.1|hypothetical protein RCOM_1512920 [Ricinus communis]	XP_002511483, EEF52085	0.0	618	420
Pe216B22.16		-	9	3169	1140	Polynucleotide 5 -hydroxyl-kinase NOL9-like	1140	gi|802652675|ref|XP_012080165.1|PREDICTED: polynucleotide 5'-hydroxyl-kinase NOL9 isoform X2 [Jatropha curcas]	XP_012080165	0.0	379	331
Pe216B22.17	Incomplete 3'	+	13	6190	1264	Transportin-3 isoform X1	1264	gi|566197271|ref|XP_002318009.2|hypothetical protein POPTR_0012s07540g [Populus trichocarpa]gi|550326592|gb|EEE96229.2|hypothetical protein POPTR_0012s07540g [Populus trichocarpa]	XP_002318009, EEE96229	0.0	410	380
Pe216F3.1		-	15	7746	3099	Phox-associated domain,Phox-like,Sorting C- isoform 1 [Theobroma cacao]	1032	gi|743794238|ref|XP_010999716.1|PREDICTED: uncharacterized protein LOC105107475 isoform X1 [Populus euphratica]	XP_010999716	0.0	1053	853
Pe216F3.2		+	3	1407	648	CCG-binding 1	215	gi|224103497|ref|XP_002313079.1|hypothetical protein POPTR_0009s11140g [Populus trichocarpa]gi|118483253|gb|ABK93529.1|unknown [Populus trichocarpa]gi|222849487|gb|EEE87034.1|hypothetical protein POPTR_0009s11140g [Populus trichocarpa]	XP_002313079, ABK93529, EEE87034	3.12E-52	207	137
Pe216F3.3		+	1	2109	2109	Pollen-specific leucine-rich repeat extensin 3	702	gi|743914492|ref|XP_011001177.1|PREDICTED: pollen-specific leucine-rich repeat extensin-like protein 3 [Populus euphratica]	XP_011001177	0.0	453	358
Pe216F3.4		-	2	1133	1047	Probable inactive receptor kinase At1g48480	348	gi|223541815|gb|EEF43363.1|serine-threonine protein kinase, plant-type, putative [Ricinus communis]	EEF43363	1.13E-171	339	287

Pe216F3.5	+	9	3908	1596	BAT2 domain-containing 1 [Theobroma cacao]	531	gi|224103501|ref|XP_002313081.1|hypothetical protein POPTR_0009s11110g [Populus trichocarpa]gi|222849489|gb|EEE87036.1|hypothetical protein POPTR_0009s11110g [Populus trichocarpa]	XP_002313081, EEE87036	0.0	498	420
Pe216F3.6	+	2	3527	738	2-oxoglutarate (2OG) and Fe (II)-dependent oxygenase superfamily [Theobroma cacao]	245	gi|566187408|ref|XP_002313083.2|oxidoreductase family protein [Populus trichocarpa]gi|550331490|gb|EEE87038.2|oxidoreductase family protein [Populus trichocarpa]	XP_002313083, EEE87038	1.23E-64	141	123
Pe216F3.7	+	3	1261	1107	Flavonol synthase flavanone 3-hydroxylase-like [Pyrus x bretschneideri]	368	gi|743914494|ref|XP_011001178.1|PREDICTED: protein SRG1-like [Populus euphratica]	XP_011001178	0.0	369	326
Pe216F3.8	+	3	1456	1131	Oxidoreductase-like family [Populus trichocarpa]	376	gi|743914494|ref|XP_011001178.1|PREDICTED: protein SRG1-like [Populus euphratica]	XP_011001178	0.0	373	316
Pe216F3.9	+	3	1392	876	Salicylic acid-binding 2-like	291	gi|567860850|ref|XP_006423079.1|hypothetical protein CICLE_v10028977mg [Citrus clementina]gi|557525013|gb|ESR36319.1|hypothetical protein CICLE_v10028977mg [Citrus clementina]	XP_006423079, ESR36319	3.61E-96	283	201
Pe216F3.10	+	3	1085	891	Salicylic acid-binding 2-like	296	gi|802599671|ref|XP_012072533.1|PREDICTED: salicylic acid-binding protein 2-like [Jatropha curcas]	XP_012072533	1.07E-109	279	208
Pe216F3.11	+	37	14151	8943	Phragmoplast orienting kinesin 2	2980	gi|802598832|ref|XP_012072464.1|PREDICTED: phragmoplast orienting kinesin 2 [Jatropha curcas]	XP_012072464	0.0	3102	2381
Pe216F3.12	+	7	3415	1620	Far upstream element-binding 1-like	539	gi|255561653|ref|XP_002521836.1|PREDICTED: far upstream element-binding protein 2 [Ricinus communis]gi|223538874|gb|EEF40472.1|RNA-binding protein Nova-1, putative [Ricinus communis]	XP_002521836, EEF40472	0.0	559	405
Pe216F3.13	+	7	1768	789	Exosome complex component MTR3-like	262	gi|802551429|ref|XP_012064793.1|PREDICTED: exosome complex component MTR3 [Jatropha curcas]gi|643738043|gb|KDP44031.1|hypothetical protein JCGZ_05498 [Jatropha curcas]	XP_012064793, KDP44031	1.49E-142	250	226
Pe216F3.14	-	9	2458	1092	OPEN STOMATA 1 family [Populus trichocarpa]	363	gi|255561655|ref|XP_002521837.1|PREDICTED: serine/threonine-protein kinase SRK2E isoform X1 [Ricinus communis]gi|223538875|gb|EEF40473.1|Serine/threonine-protein kinase SAPK10, putative [Ricinus communis]	XP_002521837, EEF40473	0.0	363	356
Pe216F3.15	+	9	5815	1410	SWIB complex BAF60b domain-containing [Theobroma cacao]	469	gi|590686340|ref|XP_007042350.1|SWIB complex BAF60b domain-containing protein, putative [Theobroma cacao]gi|508706285|gb|EOX98181.1|SWIB complex BAF60b domain-containing protein, putative [Theobroma cacao]	XP_007042350, EOX98181	0.0	483	378
Pe216F3.16	+	5	3273	1188	Two-component response regulator-like APRR2 [Populus euphratica]	395	gi|802598810|ref|XP_012072452.1|PREDICTED: probable transcription factor KAN3 [Jatropha curcas]gi|643730807|gb|KDP38239.1|hypothetical protein JCGZ_04882 [Jatropha curcas]	XP_012072452, KDP38239	2.69E-165	413	316
Pe216F3.17	+	10	2499	1272	La-related 6C	423	gi|802598806|ref|XP_012072450.1|PREDICTED: la-related protein 6C [Jatropha curcas]	XP_012072450	0.0	426	336
Pe216F3.18	+	4	1983	954	Peroxidase 10	342	gi|591403378|gb|AHL39161.1|class III peroxidase [Populus trichocarpa]	AHL39161	0.0	342	295
Pe216F3.19	+	1	393	393	Transcription factor RBF1-like	130	gi|255561671|ref|XP_002521845.1|PREDICTED: uncharacterized protein LOC8283273 [Ricinus communis]gi|223538883|gb|EEF40481.1|conserv	XP_002521845, EEF40481	1.97E-50	130	100

								ed hypothetical protein [Ricinus communis]				
Pe216F9.1	Incomplete 5'	+	1	264	264	ADP-ribosylation factor gtpase-activating AGD4-like isoform X3 [Gossypium hirsutum]	87	gi|1000967323|ref|XP_015574395.1|PREDICTED: ADP-ribosylation factor GTPase-activating protein AGD4 isoform X2 [Ricinus communis]	XP_015574395	3.13E-21	72	59
Pe216F9.2		+	1	207	207	---Na---	68	No Blast Hit				
Pe216F9.3		+	7	8737	1164	ADP-ribosylation factor gtpase-activating AGD4-like isoform X3 [Gossypium hirsutum]	387	gi|1000967327|ref|XP_015574397.1|PREDICTED: ADP-ribosylation factor GTPase-activating protein AGD4 isoform X4 [Ricinus communis]	XP_015574397	0.0	386	317
Pe216F9.4		-	3	2011	1035	Gibberellin 2-beta-dioxygenase 8-like	344	gi|566190158|ref|XP_002315815.2|hypothetical protein POPTR_0010s10700g [Populus trichocarpa]gi|550329528|gb|EEF01986.2|hypothetical protein POPTR_0010s10700g [Populus trichocarpa]	XP_002315815, EEF01986	1.15E-172	337	282
Pe216F9.5		-	8	8390	1113	ZZ-type zinc finger-containing isoform 1 [Theobroma cacao]	370	gi|1000967298|ref|XP_015574385.1|PREDICTED: uncharacterized protein LOC8275351 isoform X2 [Ricinus communis]gi|1000967300|ref|XP_015574386.1|PREDICTED: uncharacterized protein LOC8275351 isoform X2 [Ricinus communis]gi|223542195|gb|EEF43739.1|conserved hypothetical protein [Ricinus communis]	XP_015574385, XP_015574386, EEF43739	1.17E-117	275	214
Pe216F9.6		-	2	4204	2037	Inactive leucine-rich repeat receptor-like serine threonine- kinase At1g60630	678	gi|743822334|ref|XP_011021663.1|PREDICTED: inactive leucine-rich repeat receptor-like serine/threonine-protein kinase At1g60630 [Populus euphratica]	XP_011021663	0.0	671	546
Pe216F9.7	2 isoforms	-	5	4510	780	Zinc knuckle family [Populus trichocarpa]	259	gi|743822338|ref|XP_011021664.1|PREDICTED: TIMELESS-interacting protein [Populus euphratica]	XP_011021664	4.35E-91	267	186
Pe216F9.8		+	13	6810	2832	Nucleolar 14 [Ricinus communis]	943	gi|802543944|ref|XP_012082537.1|PREDICTED: nucleolar protein 14 isoform X1 [Jatropha curcas]gi|643739695|gb|KDP45433.1|hypothetical protein JCGZ_09682 [Jatropha curcas]	XP_012082537, KDP45433	0.0	944	759
Pe216F9.9		+	1	1618	951	BIG GRAIN 1-like E	316	gi|255541978|ref|XP_002512053.1|PREDICTED: protein BIG GRAIN 1-like E [Ricinus communis]gi|223549233|gb|EEF50722.1|conserved hypothetical protein [Ricinus communis]	XP_002512053, EEF50722	2.19E-138	323	271
Pe216F9.10	2 isoforms	+	4	4110	1398	Squamosa promoter-binding 6 [Populus euphratica]	465	gi|743798826|ref|XP_011011660.1|PREDICTED: squamosa promoter-binding-like protein 6 [Populus euphratica]gi|743798829|ref|XP_011011667.1|PREDICTED: squamosa promoter-binding-like protein 6 [Populus euphratica]gi|743798833|ref|XP_011011676.1|PREDICTED: squamosa promoter-binding-like protein 6 [Populus euphratica]	XP_011011660, XP_011011667, XP_011011676	5.11E-164	490	332
Pe216F9.11		+	7	1807	519	CRABS CLAW isoform X1	172	gi|802568623|ref|XP_012067815.1|PREDICTED: protein CRABS CLAW isoform X1 [Jatropha curcas]	XP_012067815	8.24E-95	171	160
Pe216F9.12	2 isoforms	+	13	9274	3417	PREDICTED: uncharacterized protein LOC8264610	1148	gi|1000981617|ref|XP_015583328.1|PREDICTED: uncharacterized protein LOC8264610 [Ricinus communis]	XP_015583328	0.0	1197	723
Pe216F9.13		+	9	4001	1542	Vacuolar fusion CCZ1 homolog isoform X1	513	gi|743785180|ref|XP_011024441.1|PREDICTED: vacuolar fusion protein CCZ1 homolog isoform X2 [Populus euphratica]	XP_011024441	0.0	514	451

Pe216F9.14	2 isoforms	+	4	2343	738	Vacuolar iron transporter 1-like	245	gi|118486116|gb|ABK94901.1|unknown [Populus trichocarpa]	ABK94901	4.53E-145	240	224
Pe216F9.15	1 isoform	-	2	3743	1212	Heat stress transcription factor A-8	403	gi|224099573|ref|XP_002311537.1|Heat shock factor protein 5 [Populus trichocarpa]gi|222851357|gb|EEE88904.1|Heat shock factor protein 5 [Populus trichocarpa]	XP_002311537, EEE88904	0.0	398	323
Pe216F9.16		+	5	1981	606	S-adenosyl-L-methionine-dependent methyltransferases superfamily [Arabidopsis thaliana]	201	gi|224101913|ref|XP_002312473.1|hypothetical protein POPTR_0008s13600g [Populus trichocarpa]gi|118484661|gb|ABK94201.1|unknown [Populus trichocarpa]gi|183585171|gb|ACC63877.1|trans-caffeoyl-CoA 3-O-methyltransferase [Populus trichocarpa]gi|222852293|gb|EEE89840.1|hypothetical protein POPTR_0008s13600g [Populus trichocarpa]	XP_002312473, ABK94201, ACC63877, EEE89840	7.96E-46	143	103
Pe216F9.17		+	4	1255	687	Caffeoyl- O-methyltransferase-like	228	gi|802596632|ref|XP_012072340.1|PREDICTED: putative caffeoyl-CoA O-methyltransferase At1g67980 [Jatropha curcas]gi|643730711|gb|KDP38143.1|hypothetical protein JCGZ_04786 [Jatropha curcas]	XP_012072340, KDP38143	2.56E-113	227	193
Pe216F9.18		+	1	425	210	---Na---	69	No Blast Hit				
Pe216F9.19	Incomplete 3'	+	1	1713	1713	UDP-Glycosyltransferase trehalose-phosphatase family isoform 1 [Theobroma cacao]	571	gi|566190284|ref|XP_002314777.2|hypothetical protein POPTR_0010s11510g [Populus trichocarpa]gi|550329577|gb|EEF00948.2|hypothetical protein POPTR_0010s11510g [Populus trichocarpa]	XP_002314777, EEF00948	0.0	571	554
Pe216I5.1		+	14	4390	1797	Uncharacterized aarf domain-containing kinase chloroplastic	598	gi|359475997|ref|XP_002280681.2|PREDICTED: uncharacterized aarF domain-containing protein kinase At4g31390, chloroplastic [Vitis vinifera]gi|296081769|emb|CBI20774.3|unnamed protein product [Vitis vinifera]	XP_002280681, CBI20774	0.0	597	572
Pe216I5.2		+	11	5192	2325	Elongation factor 1-alpha isoform X1 [Ziziphus jujuba]	774	gi|1009136103|ref|XP_015885353.1|PREDICTED: elongation factor 1-alpha isoform X1 [Ziziphus jujuba]gi|1009136105|ref|XP_015885354.1|PREDICTED: elongation factor 1-alpha isoform X1 [Ziziphus jujuba]gi|1009136107|ref|XP_015885355.1|PREDICTED: elongation factor 1-alpha isoform X1 [Ziziphus jujuba]gi|1009136109|ref|XP_015885357.1|PREDICTED: elongation factor 1-alpha isoform X1 [Ziziphus jujuba]	XP_015885353, XP_015885354, XP_015885355, XP_015885357	0.0	776	608
Pe216I5.3		+	1	597	597	Uncharacterized protein isoform 2 [Theobroma cacao]	198	gi|590571393|ref|XP_007011581.1|Uncharacterized protein isoform 2 [Theobroma cacao]gi|508781944|gb|EOY29200.1|Uncharacterized protein isoform 2 [Theobroma cacao]	XP_007011581, EOY29200	4.21E-80	198	152
Pe216I5.4		-	5	1988	1227	Zinc finger 622 isoform 1 [Theobroma cacao]	408	gi|255574095|ref|XP_002527963.1|PREDICTED: zinc finger protein 622 [Ricinus communis]gi|223532589|gb|EEF34375.1|transcription factor, putative [Ricinus communis]	XP_002527963, EEF34375	0.0	408	357
Pe216I5.5		+	13	5376	1809	KAKU4 isoform X4 [Citrus sinensis]	602	gi|743862313|ref|XP_011031358.1|PREDICTED: uncharacterized protein LOC105130507 isoform X2 [Populus euphratica]	XP_011031358	8.29E-152	639	401
Pe216I5.6		+	6	2625	1092	COP9 signalosome complex subunit 5b-like	363	gi|659088644|ref|XP_008445090.1|PREDICTED: COP9 signalosome complex subunit 5b-like [Cucumis melo]	XP_008445090	0.0	365	354

Pe216I5.7	1 isoform	+	16	5929	2862	Plasma membrane atpase 4-like	953	gi|694372604|ref|XP_009363612.1|PREDICTED: plasma membrane ATPase 4-like [Pyrus x bretschneideri]	XP_009363612	0.0	950	922
Pe216I5.8	2 isoforms	-	12	5201	1050	Centromere O isoform X2 [Gossypium raimondii]	349	gi|255548558|ref|XP_002515335.1|PREDICTED: uncharacterized protein LOC8265176 [Ricinus communis]gi|223545279|gb|EEF46784.1|conserved hypothetical protein [Ricinus communis]	XP_002515335, EEF46784	0.0	347	303
Pe216I5.9		+	1	2207	1257	Transcriptional adapter 1	418	gi|802624532|ref|XP_012076339.1|PREDICTED: uncharacterized protein LOC105637478 [Jatropha curcas]gi|643724246|gb|KDP33447.1|hypothetical protein JCGZ_07018 [Jatropha curcas]	XP_012076339, KDP33447	0.0	419	357
Pe216I5.10	5 isoforms	-	5	4741	1650	E3 ubiquitin- ligase MBR2 [Ricinus communis]	549	gi|802624535|ref|XP_012076340.1|PREDICTED: E3 ubiquitin-protein ligase RNF12-A-like [Jatropha curcas]gi|802624538|ref|XP_012076341.1|PREDICTED: E3 ubiquitin-protein ligase RNF12-A-like [Jatropha curcas]gi|643724247|gb|KDP33448.1|hypothetical protein JCGZ_07019 [Jatropha curcas]	XP_012076340, XP_012076341, KDP33448	0.0	541	405
Pe216I5.11		+	1	1953	1953	Calmodulin-binding family [Populus trichocarpa]	650	gi|566214079|ref|XP_006371799.1|calmodulin-binding family protein [Populus trichocarpa]gi|550317972|gb|ERP49596.1|calmodulin-binding family protein [Populus trichocarpa]	XP_006371799, ERP49596	1.99E-36	415	224
Pe216I5.12		-	1	558	372	PREDICTED: uncharacterized protein LOC105637482 [Jatropha curcas]	123	gi|802624544|ref|XP_012076343.1|PREDICTED: uncharacterized protein LOC105637482 [Jatropha curcas]gi|643724249|gb|KDP33450.1|hypothetical protein JCGZ_07021 [Jatropha curcas]	XP_012076343, KDP33450	6.59E-46	123	103
Pe216I5.13		-	1	1990	489	54S ribosomal mitochondrial [Cucumis melo]	163	gi|567916350|ref|XP_006450181.1|hypothetical protein CICLE_v10009422mg [Citrus clementina]gi|568860087|ref|XP_006483558.1|PREDICTED: 54S ribosomal protein L24, mitochondrial [Citrus sinensis]gi|557553407|gb|ESR63421.1|hypothetical protein CICLE_v10009422mg [Citrus clementina]gi|641848425|gb|KDO67302.1|hypothetical protein CISIN_1g039022mg [Citrus sinensis]	XP_006450181, XP_006483558, ESR63421, KDO67302	7.20E-69	152	130
Pe216I5.14		-	12	4544	1815	Pentatricopeptide repeat-containing At5g10690	604	gi|255548570|ref|XP_002515341.1|PREDICTED: pentatricopeptide repeat-containing protein At5g10690 [Ricinus communis]gi|223545285|gb|EEF46790.1|pentatricopeptide repeat-containing protein, putative [Ricinus communis]	XP_002515341, EEF46790	0.0	595	489
Pe216I5.15		+	1	201	201	Methionyl-tRNA synthetase [Theobroma cacao]	66	gi|720067067|ref|XP_010276698.1|PREDICTED: uncharacterized protein LOC104611379 [Nelumbo nucifera]	XP_010276698	1.20E-30	65	63
Pe216I5.16	2 isoforms	+	4	5246	2844	Coatomer subunit beta-2 [Populus euphratica]	948	gi|802624665|ref|XP_012076347.1|PREDICTED: coatomer subunit beta-1 [Jatropha curcas]gi|643724252|gb|KDP33453.1|hypothetical protein JCGZ_07024 [Jatropha curcas]	XP_012076347, KDP33453	0.0	948	918

Supplementary Table S4. Comparative genomic mapping between 18 *Passiflora edulis* BAC-insert sequences and the *Populus trichocarpa* genome (top) and between 15 sequences of *Passiflora edulis* BAC-inserts and the *Manihot esculenta* genome (below).

Passiflora edulis						*Populus trichocarpa*					
**BAC code**	**BAC length (bp)**	**Syntenic region length (bp)**	**Genes in the syntenic region**	**Gene average lenth (bp)**	**Intergenic space average length (bp)**	**Chr**	**Syntenic region length (bp)**	**Genes in the syntenic region**	**Gene average length (bp)**	**Intergenic space average length (bp)**	**Orthologous genes in the syntenic region**
Pe101K14+ 141H13	172,337	159,949	32	2,923	2,157	14	213,942	31	4,055	2,962	12
Pe108C16	96,753	68,880	18	2,280	1,640	6	137,749	23	3,747	5,188	16
		65,309	17	2,286	1,760	18	130,229	21	2,689	7,942	13
Pe164B18	104,102	103,945	29	2,313	1,317	4	369,800	45	2,996	6,058	20
		103,945	29	2,313	1,317	9	189,230	42	2,345	2,214	18
Pe164D9	93,527	80,789	24	1,955	1,559	4	430,901	38	2,442	9,139	27
		85,112	26	1,951	1,451	17	209,253	38	2,320	3,855	26
Pe164K17	113,504	113,313	26	3,037	1,762	14	332,637	46	3,834	3,584	23
		110,607	25	3,103	1,782	2	307,065	38	3,510	5,183	16
Pe171P13	111,123	85,809	19	2,606	2,365	7	340,005	47	3,169	34,428	12
Pe173B16	109,801	105,875	24	3,079	1,498	4	409,775	54	3,384	13,166	28
		105,875	24	3,079	1,498	9	166,729	37	2,809	3,315	29
Pe185D11	119,061	110,316	33	1,538	1,862	2	253,596	34	2,691	5,280	22
Pe185J16	103,095	47,587	11	2,693	2,149	12	231,419	14	3,736	21,564	10
Pe186E19	115,218	17,442	7	3,554	893	1	27,583	5	2,625	3,615	5
		92,977	20	2,853	1,939	1	268,117	16	5,753	12,808	8
Pe207D11	111,690	31,090	9	3,019	723	1	122,497	12	3,600	13,387	8
Pe212I1	121,384	85,114	18	3,138	1,882	2	162,212	25	4,547	11,759	14
		85,114	18	3,138	1,882	5	169,126	27	4,056	9,702	13
Pe214H11	142,456	79,416	16	3,781	1,361	9	221,003	36	2,571	3,834	17
		64,482	13	3,675	1,392	4	248,247	32	2,234	5,702	14
		60,720	12	3,950	968	9	202,191	38	3,039	2,650	13
		62,181	13	3,950	904	4	222,504	37	2,630	3,745	11
Pe215I8	129,737	79,415	15	2,774	2,950	1	166,694	18	3,190	6,428	12
Pe84I14	97,848	93,065	23	3,114	976	14	141,647	20	4,958	6,435	13

Pe84M23	93,217	92,795	25	2,777	975	2	171,100	23	3,093	4,545	15
		89,339	24	2,820	943	5	206,947	19	3,130	8,693	12
Pe93M2	100,436	98,828	25	2,245	5,735	12	199,350	38	2,536	9,887	17
		88,334	23	2,269	5,982	15	207,961	33	3,192	10,369	18
Pe93N7	106,968	105,007	24	3,121	1,423	6	340,655	46	3,745	11,582	23
		99,896	23	3,035	1,490	18	337,287	33	3,358	20,496	16
**Total**	**2,042,257**	**1,702,975***	**406***	**2,785***	**1,871***		**7,137,451**	**966**	**3,290**	**8,694**	**501**

		Passiflora edulis				*Maninhot esculenta*					
**BAC code**	**BAC length (bp)**	**Syntenic region length (bp)**	**Genes in the syntenic region**	**Gene average lenth (bp)**	**Intergenic space average length (bp)**	**Chr**	**Syntenic region length (bp)**	**Genes in the syntenic region**	**Gene average length (bp)**	**Intergenic space average length (bp)**	**Orthologous genes in the syntenic region**
Pe101K14+ 141H13	172,337	170,391	36	2,721	2,072	1	183,133	29	3,466	3,081	16
		164,887	35	2,673	2,100	5	259,161	33	4,516	3,884	14
Pe108C16	96,753	68,880	18	2,280	1,640	3	76,043	14	3,672	4,372	10
		63,474	16	2,379	1,696	16	88,458	15	3,432	6,018	10
Pe164B18	104,102	103,945	29	2,314	1,318	17	182,720	38	2,430	2,525	17
		103,945	29	2,314	1,318	15	345,243	35	4,197	5,835	12
Pe164D9	93,527	93,489	28	1,869	1,601	2	206,242	38	2,389	3,122	25
		85,112	26	1,951	1,452	1	118,187	21	2,636	3,144	15
Pe164K17	113,504	101,996	22	3,149	1,923	1	189,788	23	3,879	7,638	11
		110,607	25	3,104	1,782	5	393,258	38	4,822	6,328	17
Pe173B16	109,801	92,992	23	3,094	1,087	4	235,649	17	4,748	9,462	20
Pe185D11	119,061	110,279	33	1,613	1,785	12	317,886	43	2,362	5,330	27
		112,597	34	1,602	1,764	13	254,296	29	2,584	6,216	18
Pe185J16	103,095	88,563	21	2,351	2,040	1	308,705	27	6,736	20,718	12
Pe186E19	115,218	50,679	16	2,224	1,110	14	304,339	14	5,566	17,417	9
		50,679	16	2,224	1,110	6	101,361	12	4,021	4,828	8
Pe207D11	111,690	28,902	7	3,712	1,450	15	48,780	11	4,100	4,838	6
Pe212I1	121,384	85,114	19	3,138	1,882	18	172,143	23	4,370	11,733	14
Pe215I8	129,737	118,786	22	326	2,382	17	162,363	19	3,787	5,963	14
		124,698	26	3,008	2,169	15	193,725	24	4,418	3,815	14
Pe84I14	97,848	96,433	24	3,092	969	1	135,657	19	5,005	6,468	12

		94,441	24	3,033	944	5	211,686	26	4,890	9,873	14
Pe84M23	93,217	66,677	17	2,865	1,126	18	148,682	18	3,680	5,096	13
		53,520	15	2,678	956	2	137,511	19	2,670	5,214	8
Pe93M2	100,436	98,828	25	2,245	5,735	6	126,299	22	2,782	5,921	17
		78,587	19	2,256	2,198	14	151,939	26	3,883	7,350	12
**Total**	**1,681,710**	**1,392,795***	**348***	**2,641***	**1,850***		**5,053,254**	**633**	**3,886**	**6,777**	**365**

- Chr, chromosome
- *No redundancy
